# CSRP2 promotes the glioblastoma mesenchymal phenotype via p130Cas-mediated NF-κB and MAPK pathways

**DOI:** 10.1186/s13046-025-03484-7

**Published:** 2025-08-05

**Authors:** Jiawei He, Liang Zhang, Hao Xu, Chengtian Gao, Wentao Zhao, Bingchang Zhang, Wanhong Han, Wenpeng Zhao, Guowei Tan, Sifang Chen, Ping Zhong, Zhe Shen, Jian Meng, Ziqian Tang, Hanwen Lu, Xin Gao, Zhangyu Li, Wenhua Li, Jianyao Mao, Bosen Liu, Yun-wu Zhang, Zhanxiang Wang

**Affiliations:** 1 Department of Neurosurgery, Department of Neuroscience, School of Medicine, The First Affiliated Hospital of Xiamen University, Xiamen University, Xiamen, Fujian 361003 China; 2 Fujian Provincial Key Laboratory of Neurodegenerative Disease and Aging Research, Institute of Neuroscience, School of Medicine, Xiamen University, Xiamen, Fujian 361102 China; 3 Xiamen Neurosurgical Quality Control Center, Xiamen, Fujian 361003 China; 4 Department of Neurosurgery, The First Affiliated Hospital of USTC, Division of Life Sciences and Medicine, University of Science and Technology of China, Hefei, Anhui 230001 China; 5 Department of Neurosurgery, The First Affiliated Hospital of Henan, University of Science and Technology, Luoyang, Henan, 471000 China; 6 Clinical Research Institute of the First Affiliated Hospital of Xiamen University, Fujian Key Laboratory of Brain Tumors Diagnosis and Precision Treatment, Xiamen Key Laboratory of Brain Center, The First Affiliated Hospital of Xiamen University, School of Medicine, Xiamen University, Xiamen, 361102 China

**Keywords:** Cysteine-rich protein 2, Glioblastoma multiforme, Mitoxantrone, NF-κB, p130Cas

## Abstract

**Background:**

Cysteine-rich protein 2 (CSRP2) plays a role in a variety of biological processes including cell proliferation and differentiation. However, whether and how CSRP2 participates in the malignancy of glioblastoma multiforme (GBM), including its proneural-to-mesenchymal transition (PMT), remains unclear.

**Methods:**

CSRP2 expression in low-grade and high-grade gliomas was analyzed, and survival analyses were performed in patients with gliomas with high and low CSRP2 expression in various tumor databases. Quantitative real-time PCR (qRT–PCR) and western blotting (WB) were used to detect the expression of CSRP2 in GBM and control brain tissues. CSRP2 function in GBM was determined by a series of functional tests in *vitro* and in *vivo*. WB, co-immunoprecipitation (co-IP) and immunofluorescence were used to determine the relation between CSRP2 and p130Cas. Mechanisms of CSRP2 involvement in GBM progression were analyzed with gene set enrichment analysis and KEGG enrichment analysis in available databases. WB was used to determine the relation between CSRP2 and PMT markers, NF-κB and MAPK signaling-related proteins, and apoptosis-related proteins. Microscale thermophoresis assay was used to analyze whether mitoxantrone (MTO) and CSRP2 could bind. MTO function was determined by a series of functional tests in *vitro*, while the relation between MTO and PMT markers, NF-κB and MAPK signaling-related proteins, and apoptosis-related proteins was analyzed by WB in GBM cell lines stably overexpressing CSRP2.

**Results:**

We found that CSRP2 expression significantly increased in GBM, especially mesenchymal GBM, and that glioma patients with high CSRP2 expression possibly had poor prognosis. CSRP2 overexpression in GBM cells promoted proliferation, colony formation, migration, invasion, temozolomide resistance, and PMT in vitro and tumor formation in vivo. While knockdown of CSRP2 had the opposite effects. Mechanistically, we revealed that CSRP2 interacted with p130Cas, thereby regulating the NF-κB and the MAPK signaling pathways. CSRP2 overexpression and knockdown increased and decreased p130Cas levels and NF-κB and MAPK activities, respectively. Both p130Cas downregulation and NF-κB inhibition reversed the elevated PMT and NF-κB and MAPK activities resulted from CSRP2 overexpression. Finally, we identified that MTO bound CSRP2 and inhibited the malignant effects of CSRP2 overexpression on GBM cells.

**Conclusions:**

Our findings demonstrate that CSRP2 promotes GBM malignancy including PMT and temozolomide resistance through activating p130Cas-mediated NF-κB and MAPK signaling pathways. Inhibiting CSRP2 function, including using MTO, may become a novel therapeutic approach for GBM.

**Supplementary Information:**

The online version contains supplementary material available at 10.1186/s13046-025-03484-7.

## Introduction

Glioblastoma multiforme (GBM) is the most common primary malignant tumor of the central nervous system [1]. GBM grows infiltratively in brain tissue with high heterogeneity, is difficult to be completely resected by surgery, and has a low sensitivity to adjuvant chemotherapy and radiotherapy, all of which make GBM susceptible to resistance and recurrence. Conventional treatment including surgery, radiotherapy, and chemotherapy has not been observed to significantly improve survival outcomes in patients with GBM [2], and the current median survival is only about 15 months [3]. Gliomas are classified into four grades (I-IV) according to the World Health Organization (WHO) classification, and into proneural (PN), neural (NL), classical (CL), and mesenchymal (MES) according to molecular typing [4]. CL and MES are mainly found in GBM and have the worst clinical prognosis [5, 6]. It has been found that gliomas of the PN subtype transform into the MES subtype during progression and recurrence after treatment with radiotherapy, a process called proneural-to-mesenchymal transition (PMT) [7]. The molecular events that drive PMT are thought to be similar to those that drive epithelial-mesenchymal transition (EMT) in cancer cells. EMT enhances the invasive and metastatic ability of cancer cells and is associated with chemotherapy resistance [8–10]. PMT, which is similar to EMT, is also believed to participate in the poor prognosis of glioma. Therefore, in-depth studies to elucidate the molecular mechanisms underlying the malignant progression of gliomas and their inherent resistance to adjuvant chemotherapy and radiotherapy are of great importance in the search for effective new targets for glioma therapy and the development of new drugs for glioma treatment.

Cysteine-rich protein 2 (CSRP2) is a member of the LIM-only family of cysteine-rich proteins and contains two LIM zinc-finger domains [11]. CSRP2 is widely expressed in a variety of tissues [12] and plays a role in diverse biological processes such as signaling, cytoskeleton formation, cell proliferation, and differentiation [13]. Recent studies have revealed that CSRP2 also participates in a variety of tumors. However, the function of CSRP2 in different tumors is controversial, for example, CSRP2 was found to promote invasion and metastasis in breast cancer [14] and chemoresistance in leukemia [15], but inhibit proliferation, migration, and invasion in gastric and colon cancers [16, 17]. Two very recent studies reported that CSRP2 could maintain the malignant phenotypes of gliomas through the Notch signaling pathway and inhibit glioma necroptosis through activating the JAK-STAT1 signaling pathway [18, 19]. However, the detailed molecular functions of CSRP2 in GBM, especially its PMT process, required further elucidation.

In addition to the Notch signaling pathway and the JAK-STAT1 signaling pathway, several other signaling pathways, such as the NF-κB signaling pathway and the RAS/RAF/MEK/ERK signaling pathway (also known as the MAPK signaling pathway), are also linked to the malignancy of GBM. NF-κB is a family of five transcription factor proteins, including NF-κB1/p105/p50, NF-κB2/p100/p52, RelA/p65, RelB, and c-Rel that can form various heterodimers or homodimers. In the resting state, NF-κB proteins localize in the cytoplasm in combination with their repressor IκB (e.g., IκBα and IκBβ). Upon stimulation, IκB kinases (IKKα, IKKβ, and NEMO) are activated and phosphorylate IκB, leading to its ubiquitination and degradation, so that transcriptionally active NF-κB dimers can translocate to the nucleus and regulate the expression of downstream genes [20]. Enormous evidence has shown that aberrant NF-κB activation is crucial for the onset, progression, metastasis, and resistance of multiple cancers including GBM [21–23]. The activation of NF-κB also plays an important role in the transformation of MES. For example, NF-κB can be activated in mesenchymal GBM cells by a range of intrinsic and extrinsic signals that promote mesenchymal differentiation. NF-κB in the nucleus promotes mesenchymal differentiation by inducing the expression of mesenchymal genes such as CD44 and N-Cadherin [24]. In addition, NF-κB promotes mesenchymal changes in the tumor microenvironment by regulating the composition of secreted cytokines, extracellular matrix (ECM) proteins, and other enzymes, thereby promoting invasion, angiogenesis, and treatment resistance [24]. Radiotherapy-induced changes in intratumor PMT are also associated with NF-κB activation and macrophage/microglia involvement in GBM and lead to poor prognosis of GBM patients [25, 26].

Ras is a GTP-binding protein with GTPase activity. When activated by binding of receptor tyrosine kinase (RTK) to ligands on the membrane, Ras can cause a phosphorylation cascade of downstream protein kinases such as the RAF/MEK/ERK axis (i.e. the MAPK signaling), ultimately leading to transcription factor activation and the expression of genes involved in cell cycle and survival [27–31]. Ras is the most commonly mutated oncogene in all human cancers and overactive Ras/MAPK signaling has emerged as one of the most important oncogenic drivers [28, 29, 32]. In pediatric low grade gliomas (PLGG), most of the mutations identified locate in and activate the MAPK signaling pathway [33]. In addition, the MAPK signaling pathway is interconnected with the NF-κB signaling pathway in tumors. For example, the MAPK pathway can activate NF-κB transcription, thereby co-promoting human head and neck squamous cell carcinomas progression [34, 35]. Furthermore, in human prostate cancer, inhibition of the MAPK signaling suppresses the NF-κB signaling, thereby promoting apoptosis [36]. In human neuroblastoma cells, inhibition of the NF-κB activity further reduced the MAPK signaling, leading to apoptosis [37]. However, whether and how CSRP2 regulates NF-κB and MAPK signaling pathways in tumors, especially in GBM, remains unclear.

Mitoxantrone (MTO) is a topoisomerase inhibitor used in the treatment of breast cancer, hepatocellular carcinoma, ovarian cancer, non-Hodgkin’s lymphoma, and leukemia [38–40]. Intratumoral injection of MTO also showed efficacy in improving the survival of patients with GBM [38–40]. The antitumor effects of MTO have been associated with the NF-κB and the MAPK signaling pathways [41, 42]. However, the detailed molecular mechanism underlying MTO function has yet to be determined.

In this study, we found that CSRP2 expression was upregulated in GBM patients and that high expression of CSRP2 possibly associated with poor prognosis. In addition, CSRP2 overexpression promoted the malignancy of GBM cells and their tumorigenicity in an intracranial xenograft tumor model, whereas CSRP2 knockdown had the opposite effects. Furthermore, we showed that CSRP2 interacted with p130Cas to promote PMT in GBM by activating the NF-κB and the MAPK signaling pathways. Finally, we identified that mitoxantrone could bind CSRP2 and inhibit the malignant function of CSRP2 in GBM.

## Materials and methods

### Glioma databases and data analysis

RNA-seq expression data and clinical and molecular information of glioma patient samples were obtained from public cancer/glioma datasets, including the China Glioma Genome Atlas (CGGA, http://www.cgga.org.cn/), The Cancer Genome Atlas Databases (TCGA) in the UCSC Xena platform (https://xena.ucsc.edu/), Rembrandt in the GEO database (https://www.ncbi.nlm.nih.gov/gds/), Gene Expression Profiling Interaction Analysis (GEPIA, http://gepia.cancer-pku.cn/), and Gravendeel in the GlioVis database (http://gliovis.bioinfo.cnio.es/). Differences in overall survival between CSRP2-low and CSRP2-high groups were analyzed using Kaplan-Meier curves and the log-rank test. The median CSRP2 expression was used as the cutoff point. Correlation analysis was performed using Pearson analysis. Gene set enrichment analysis (GSEA) was performed using the CGGA data and Broad Institute GSEA version 4.1.0 software. For KEGG pathway enrichment analysis, data from TCGA were divided into CSRP2-high and CSRP2-low groups with the median CSRP2 expression as the cutoff point. Differentially expressed genes between the two groups were subjected to KEGG enrichment analysis using R language version 4.2.1 software.

### Clinical samples

Biopsy samples (WHO grade IV) from GBM patients and non-tumor brain biopsy samples from patients with traumatic brain injury or epilepsy were collected and freshly frozen at the Department of Neurosurgery, the First Affiliated Hospital of Xiamen University. All patients gave informed consent. The study protocol was approved by the Ethics Committee of the First Affiliated Hospital of Xiamen University (XMYY-2022KY076).

### Lentivirus production

CSRP2 and p130Cas shRNA sequences below were designed by the Thermo Scientific™ TRC Lentiviral™ shRNA Library and synthesized by TsingKe Biotech. shRNAs were inserted into the pLKO.1 vector (a kind gift from Dr. Qinxi Li).

shCSRP2#1 sense: 5′- CAGGCCTACAACAAATCCAAA-3′;

shCSRP2#1 antisense: 5′- TTTGGATTTGTTGTAGGCCTG-3′;

shCSRP2#2 sense: 5′- GTGAAATTCTACCAGCATTAA-3′;

shCSRP2#2 antisense: 5′- TTAATGCTGGTAGAATTTCAC-3′;

shp130Cas sense: 5′- GCTGAAGCAGTTTGAACGACT − 3′;

shp130Cas antisense: 5′- AGTCGTTCAAACTGCTTCAGC − 3′.

Full-length human CSRP2 cDNA was inserted into the lentivirus vector pBoBi (a kind gift from Dr. Qinxi Li).

For lentivirus packaging, HEK293T cells were co-transfected with constructed lentivirus vectors and packaging plasmid mixes (PSPAX and PMD2G plasmids) using the Hieff Trans Liposomal Transfection Reagent (YEA-SEN). Lentiviral particles in the media were harvested 48 h later.

### Cell culture and stable cell line construction

The human GBM cell lines (U87-MG and U251) and HEK293T and normal human astrocyte (NHA) cell lines were originally obtained from the American Typical Culture Collection Center (ATCC, USA) and are maintained in our laboratory. The temozolomide-resistant cell (U87-MG T3rd) is a kind gift from Dr. Yongping You. These cells were cultured in high-glucose DMEM (Thermo Fisher) containing 10% FBS (Excell, Shanghai, China) and antibiotics (1% penicillin and streptomycin, Gibco) at 37 °C with humidified air of 5% CO2. U87-MG, U251, and U87-MG T3rd cells were transduced with appropriate amounts of lentivirus expressing CSRP2 shRNAs or CSRP2 cDNA and corresponding controls in the presence of 10 µg/mL polyglutamine (YEA-SEN). Twenty-four hours after transduction, cells were selected with puromycin (Shanghai Sangyo Biotechnology) to obtain stable cell lines for subsequent experiments.

### Quantitative real time-PCR (qRT-PCR)

Total RNA was extracted using the TRIzol reagent (YEA-SEN) and cDNA was synthesized using the HiScript II Q RT SuperMix for qPCR Kit (Vazyme), following the manufacturers’ protocols. *CSRP2* mRNA levels were measured by qRT-PCR using 2X Universal SYBR Green Fast qPCR Mix (Abclonal) on a StepOnePlus real-time PCR system (LightCycle). β-actin mRNA levels were utilized as an internal control. Relative mRNA expression was determined using the 2 − ΔΔct method. The primer sequences are listed as the following:

CSRP2-F: 5′-TGGGAGGACCGTGTACCAC − 3′,

CSRP2-R: 5′- CCGTAGCCTTTTGGCCCATA − 3′;

β-actin-F: 5′-ATCAAGATCATTGCTCCTCCTGAG-3′,

β-actin-R: 5′-CTGCTTGCTGATCCACATCTG-3′.

### Cell proliferation assay

1000 cells/well were seeded in 96-well plates. Cell proliferation was studied every 24 h using a CCK8 Kit (US EVERBRIGHT, China), following the manufacturer’s protocol.

### Colony formation assay

1000 cells/well were seeded in 6-well plates. After 14 d of cultivation, cells were fixed with 4% paraformaldehyde for 30 min and stained with 0.1% crystal violet for 1 h. The numbers of colonies with a size of 2 mm or greater were counted.

### Cell migration assay

20,000 cells were seeded in the upper Transwell chamber (polycarbonate filter with 8-mm pores, Corning) containing 200 µL serum-free medium. The bottom chamber was supplemented with 600 µL complete medium as a chemo-attractant. After 48 h incubation, non-migrated cells on the upper surface of the filters were removed by a cotton swab. Migrated cells on the lower surface of the filters were fixed with 4% formaldehyde for 30 min and then stained with 0.1% crystal violet at room temperature for another 1 h. After being washed with 1×PBS, cells were photographed under inverted microscope.

### Cell invasion assay

50,000 cells were seeded in the upper Transwell chamber coated with 200 mg/mL Matrigel (Corning) and containing 200 µL serum-free medium. The bottom chamber was supplemented with 600 µL complete medium as a chemo-attractant. After 24 h incubation, noninvasive cells on the upper surface of the filters were removed by a cotton swab. Invasive cells on the lower surface of the filters were fixed with 4% formaldehyde for 30 min and then stained with 0.1% crystal violet for another 1 h. After being washed with 1×PBS, cells were photographed under inverted microscope.

### Cell apoptosis analysis by flow cytometry

Treated cells were harvested and incubated with an Annexin V-fluorescein isothiocyanate (FITC)/propidium iodide (PI) apoptosis detection kit (BD Biosciences), following the manufacturer’s protocol. Stained cells were analyzed with a flow cytometer.

### Western blotting (WB) and co-immunoprecipitation (co-IP)

Brain tissue samples or cells were lysed with 1% TNEN buffer. Equal amounts of protein lysates (20 µg) were separated by SDS-PAGE and transferred to PVDF membranes. The membranes were sequentially incubated with primary antibodies and appropriate HRP-conjugated secondary antibodies. Protein bands on the membranes were scanned with the Azure C300 Imaging System (USA) and band intensities were measured using ImageJ (National Institutes of Health). Original WB data are provided in Supplementary Fig. S1 and their quantification and statistical analysis data are presented in Supplementary Table S1.

For Co-IP assay, cell lysates were incubated with primary antibodies and protein A/G MagBeads at 4 °C overnight. Immunoprecipitated proteins were then subjected to western blotting analysis.

Antibodies used include: rabbit anti-CSRP2 (Cat# 10892-2-AP, 1:2000), rabbit anti-Flag (Cat# 20543-1-AP, 1:8000), rabbit anti-HA (Cat# 51064-2-AP, 1:2000), and rabbit anti-TAZ (Cat# 23306-1-AP, 1:4000) from Proteintech; rabbit anti-p-IκBα (Cat# AF1870, 1:1000), rabbit anti-p-NF-κB p65 (site 536, Cat# AF5881, 1:1000), and rabbit anti-NF-κB p65 (Cat# AF5243, 1:1000) from Beyotime; rabbit anti-Bcl-2 (Cat# 2870 S, 1:1000), rabbit anti-Bcl-xL (Cat# 2764 S, 1:1000), rabbit anti-β-Catenin (Cat# 9582 S, 1:1000), rabbit anti-CREB (Cat# 9197 S, 1:1000), rabbit anti-p-CREB (Cat# 9198 S, 1:1000), rabbit anti-cleaved Caspase-8 (Cat# 9496 S, 1:1000), rabbit anti-cleaved PARP (Cat# 5625 S, 1:1000), rabbit anti- C/EBPβ (Cat# 3087 S, 1:1000), rabbit anti- Erk1/2 (Cat# 9102 S, 1:1000), rabbit anti-p-Erk1/2 (Cat# 4370 S, 1:1000), rabbit anti- IRAK4 (Cat# 4363 S, 1:1000), rabbit anti-IκBα (Cat# 4812 S, 1:1000), rabbit anti-Met (Cat# 8198 S, 1:1000), rabbit anti-N-Cadherin (Cat# 13116 S, 1:1000), rabbit anti-Olig2 (Cat# 65915 S, 1:1000), rabbit anti-p130Cas (Cat# 13846 S, 1:1000), rabbit anti-Raf-1 (Cat# 9422 S, 1:1000), rabbit anti-p-Raf-1 (Cat# 9421 S, 1:1000), and rabbit anti-Rsk1/2/3 (Cat# 9355 S, 1:1000) from Cell Signaling Technology; mouse anti-E-Cadherin (Cat# 610404, 1:250) from BD Biosciences; and rabbit anti-GAPDH (Cat# AB0036, 1:10000) from Abways.

### Immunofluorescence (IF) assay

Treated cells were fixed with 4% paraformaldehyde for 20 min, permeabilized with 0.2% Triton X-100 for 10 min, blocked with 2% fetal bovine serum for 1.5 h, incubated with rabbit anti-CSRP2 (Cat# 10892-2-AP, 1:200) and mouse anti-p130Cas (Cat# 67215-1-Ig, 1:200) antibodies overnight, and then incubated with fluorescence-labeled secondary antibodies for 1 h. After stained with 4-amino-6-diamino-2-phenylindole (DAPI) for 10 min, cells were observed under an Olympus FV4000 confocal laser scanning microscope (Olympus USA, NY, USA).

### Focused ion beam scanning Electron microscope

Treated cells were fixed in 2.5% glutaraldehyde at 4 °C for 3 h and in 1% osmium tetroxide at 4 °C for 1 h. After being dehydrated with gradient alcohol, cells were resin-embedded and sliced with an ultra-thin sectioning machine (Leica, Germany). The ultra-thin Sects. (60–80 nm) were affixed to a copper mesh, stained with uranyl acetate and lead citrate, and visualized under a Focused Ion Beam Scanning Electron Microscope (Helios 5 UC, ThermoFisher).

### Virtual drug screening

A structure-based virtual screening strategy was applied to identify potential CSRP2-binding molecules from a library of FDA-approved drugs. The protein structure of CSRP2 was predicted by AlphaFold (Identifier ID: AF-Q16527-F1). The virtual screening was performed using the Schrödinger’s software.

### Microscale thermophoresis (MST) assay

CSRP2 recombinant protein was obtained from Wuhan Huamei and labeled with fluorescence using a Monolith™ RED-NHS Secondary Protein Labeling Kit (Nano Temper), following the manufacturer’s protocol. Mitoxantrone (MTO) was from MCE (Cat# HY-13502). For the binding study, 5 µL of fluorescence-labeled CSRP2 recombinant protein was mixed with 5 µL of different concentrations of MTO. After 5 min incubation at room temperature, samples were loaded into MST NT.115 standard glass capillaries and measured at 70–100% excitation power using the NanoTemper Monolith Pico. At least three independent experiments were repeated. The data were then imported into NanoTemper’s MO affinity analysis software (v.2.3) and the Kd value was calculated using the Kd model.

### In vivo intracranial xenograft tumor model

6-week-old female BALB/c nude mice were used for tumor cell xenograft. Mice were randomly separated into indicated groups (*n* = 11 per group). After anesthesia, 1 × 10^5^ cells were transplanted into the right frontal lobe of each mouse by a microinjector. Mouse body weight and living status were recorded. Four weeks after implantation, mice were anaesthetized and subjected to cerebral magnetic resonance imaging (MRI). At the end of the experiment, survival mice were sacrificed and brains were dissected for histopathologic and Ki-67 staining. All animal experiments were performed in a non-blinded manner. Animal procedures were in accordance with the guidelines of the National Institutional Animal Health Guide for the Care and Used of Laboratory Animals and approved by the Animal Ethics Committee of Xiamen University (XMULAC20230235).

### Immunohistochemistry

Tumor tissue immunohistochemistry was performed by Servicebio. Briefly, tumor tissues were fixed with 4% paraformaldehyde, paraffin-embedded, sectioned, and subjected to hematoxylin staining as previously described [43]. Tumor areas were analyzed with 3Dslicer. Paraffin-embedded tissues were immunostained with a Ki-67 antibody (Servicebio, Cat# GB121141) and HRP-labeled goat anti-mouse IgG (Servicebio, Cat# GB23301), and then stained with a DAB kit (Servicebio). The ratio of Ki-67-positive cells in tumor tissues was counted with Image J.

### Statistical analyses

Statistical analyses were performed using Graphpad Prism 8.0 software. No samples were excluded from analysis. All quantitative data were presented as the mean ± SEM. Variances were similar between groups for comparisons. Two-tailed unpaired t-test was used to compare the differences between two groups. One-way ANOVA and two-way ANOVA with post hoc tests were used for comparisons between multiple groups. *p* < 0.05 was considered as statistically significant.

## Results

### CSRP2 expression is upregulated in GBM and associated with tumor progression and possibly with poor prognosis

**Fig. 1:**
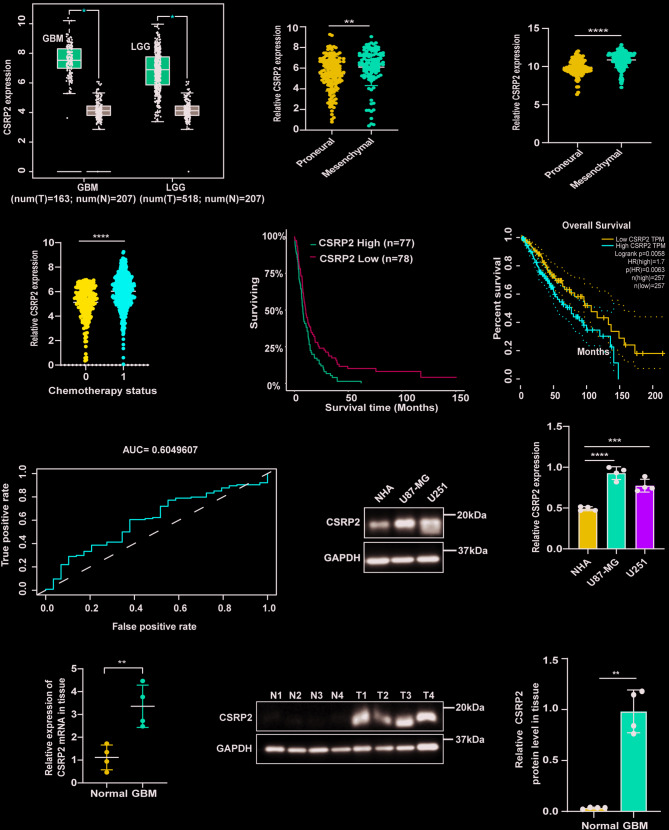
CSRP2 is upregulated in GBM and correlated with advanced progression and poor prognosis of GBM. (**A**) Comparison of CSRP2 expression between GBM and normal controls and between LGG and normal controls, using data from the GEPIA database. (**B**) Comparison of CSRP2 expression between GBM proneural and mesenchymal subtypes using the CGGA database. Unpaired t test, *n* = 157 for proneural and *n* = 116 for mesenchymal. (**C**) Comparison of CSRP2 expression between GBM proneural and mesenchymal subtypes using the TCGA database. Unpaired t test, *n* = 138 for proneural and *n* = 157 for mesenchymal. (**D**) Comparison of CSRP2 expression between different chemotherapy status (0 and 1) of GBM using the CGGA database. Unpaired t test, *n* = 275 for chemotherapy status 0 and *n* = 635 for chemotherapy status 1. (**E**, **F**) Kaplan‒Meier survival analysis of patients with low or high CSRP2 expression using data from the Gravendeel database (**E**) and the GEPIA database (**F**). Log-rank test. (**G**) Receiver Operating Characteristic curve (ROC curve) of CSRP2 predicting survival or death in GBM patients using the TCGA database. (**H**, **I**) Equal amounts of cell lysates of NHA and GBM cell lines (U87-MG and U251) were subjected to western blotting (**H**) and quantitative comparison (I) for CSRP2. GAPDH was used as an internal loading control. One-way ANOVA with Tukey’s post hoc test. *n* = 4 per group. (**J**) *CSRP2* mRNA levels in 4 non-tumor brain biopsies from traumatic brain injury or epilepsy patients (N1-N4) and 4 GBM samples (grades IV) were compared. Unpaired t test. (K, L) Equal amounts of protein lysates from the 4 GBM samples and the 4 non-tumor controls were subjected to western blotting (K) and quantitative comparison (**L**) for CSRP2. Unpaired t test. Data represent mean ± SEM, *p < 0.05, **p < 0.01, ***p < 0.001, ****p < 0.0001

To investigate whether CSRP2 is involved in the malignant progression of gliomas, we first analyzed CSRP2 expression using data from TCGA, GEPIA, Gravendeel, Rembrandt, and CGGA databases. We found that CSRP2 expression significantly increased in both low-grade glioma (LGG) and GBM tissues when compared to normal tissues (Fig. 1A and Supplementary Fig. S2A), and CSRP2 expression was higher in GBM with advanced pathological grades than those with low pathological grades (Supplementary Fig. S2B, C). Furthermore, we found elevated CSRP2 expression in the mesenchymal type of GBM compared to the proneural type (Fig. 1B, C), and in GBM patients who had undergone chemotherapy (status 1) compared to those without chemotherapy (status 0) (Fig. 1D). These results implicate that CSRP2 is involved in the PMT process in GBM. Moreover, we found that GBM and/or LGG patients with high CSRP2 expression had a worse prognosis than those with low CSRP2 expression using Gravendeel (Fig. 1E), GEPIA (Fig. 1F), and CGGA datasets (Supplementary Fig. S2D), though there was no significant correlation between CSRP2 expression and GBM prognosis in TCGA and Rembrandt datasets (Supplementary Fig. S2E, F). Inconsistency between the analysis results of a same glioma prognostic marker using different databases have been noticed in multiple literature reports [44–47]. Possible explanations for this are heterogenous patient compositions in different databases, and/or patients in different cohorts were subjected to heterogenous treatments. When we predicted the survival or death of GBM patients using CSRP2 as a Receiver Operating Characteristic curve (ROC curve), the AUC was 0.6049607 (Fig. 1G). Isocitrate dehydrogenase (IDH) mutations and co-deletion of chromosome arms 1p and 19q (1p/19q) favor longer survival of GBM patients and are important molecular biomarkers for the diagnosis and treatment of GBM [48, 49]. We also found that CSRP2 expression significantly decreased in patients with IDH mutations and chromosome arm 1p/19q co-deletions when compared to respective controls (Supplementary Fig. S2G, H). To further determine whether CSRP2 is involved in GBM, we compared CSRP2 protein in Normal Human Astrocytes (NHA) and GBM cell lines (U87-MG and U251) and found that CSRP2 levels in GBM cell lines were significantly higher than those in NHA (Fig. 1H, I). Finally, we collected clinical samples from 4 non-brain tumor patients and 4 GBM cases for analysis, found that both mRNA (Fig. 1J) and protein levels (Fig. 1K, L) of CSRP2 were significantly higher in GBM than controls.

### CSRP2 overexpression promotes GBM progression

**Fig. 2:**
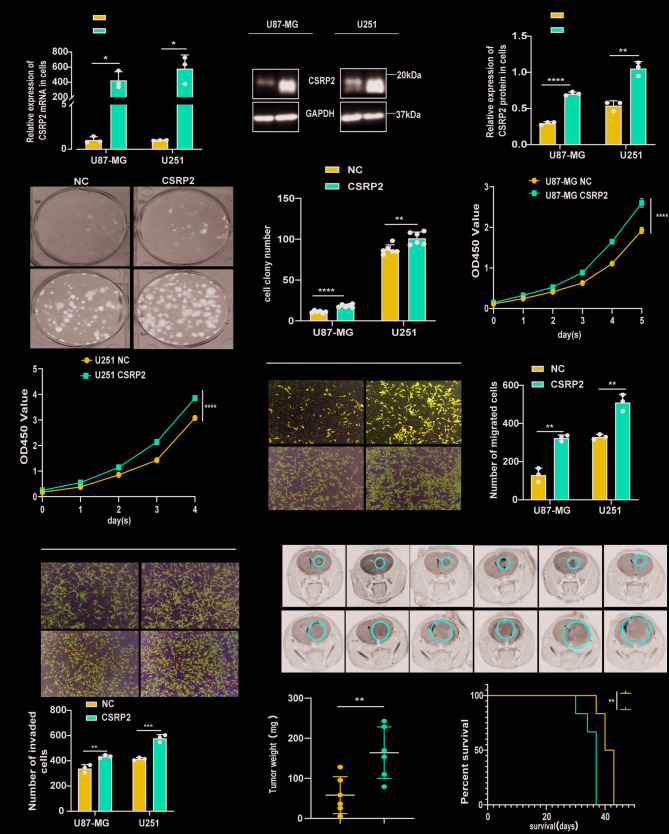
CSRP2 overexpression promotes the malignancy of GBM cells. (**A**) U87-MG and U251 cells were stably transduced with lentiviruses expressing CSRP2 or control (NC) and the mRNA levels of *CSRP2* were compared. Unpaired t test, *n* = 3 per group. (**B**, **C**) CSRP2 protein levels in U87-MG and U251 cells stably overexpressing CSRP2 and controls were analyzed by western blotting (**B**) and densitometry quantification for comparison (**C**). Unpaired t test, *n* = 3 per group. (**D**, **E**) U87-MG and U251 cells with stable CSRP2 overexpression and controls were assayed for colony formation (**D**) and the colony numbers were compared (**E**). Unpaired t test, *n* = 6 per group. (**F**, **G**) U87-MG (**F**) and U251 (**G**) cells with stable CSRP2 overexpression and controls were assayed for their proliferation. Two-way ANOVA with Sidak’s post hoc test, *n* = 6 per group for U87-MG and U251. (**H**-**K**) Cells with stable CSRP2 overexpression and controls were studied for their migration ability (**H**, **I**) and invasion ability (**J**, **K**). Unpaired t test, *n* = 3 per group. (**L**, **M**) U87-MG cells with stable CSRP2 expression and controls were xenografted into the mouse brain. Tumor formation was observed by MRI brain images (**L**) and tumor weights were compared (**M**). Red cycles indicate tumor areas. Unpaired t test, *n* = 6 per group. (**N**) Kaplan-Meier survival curves of xenografted mice. Log-rank test, *n* = 6 per group. Data represent mean ± SEM, *p < 0.05, **p < 0.01, ***p < 0.001, ****p < 0.0001

To investigate the function of CSRP2 in GBM, we infected two glioma cell lines (U87-MG and U251) with lentiviruses overexpressing CSRP2 or control, and obtained stable cell lines after puromycin selection. qRT-PCR and western blotting analyses confirmed the elevated expression of CSRP2 in these cells (Fig. 2A-C). Overexpression of CSRP2 significantly promoted colony formation (Fig. 2D, E), cell proliferation (Fig. 2F, G), cell migration (Fig. 2H, I), and cell invasion (Fig. 2J, K). We also found that cell apoptosis decreased after CSRP2 overexpression (Supplementary Fig. S3A, B). CSRP2 overexpression also increased the levels of two anti-apoptotic proteins, Bcl-2 and Bcl-xL, and decreased the levels of cleaved PARP and cleaved Caspase-8 that are indicative of apoptosis (Supplementary Fig. S3C-E).

To assess the effect of CSRP2 overexpression on GBM tumor formation in vivo, we transplanted U87-MG cells with stable CSRP2 overexpression and control cells into the brains of nude mice. Approximately one month later, mice were scanned by MRI to confirm the presence of tumors (Fig. 2L). We subsequently dissected tumors from these mice for comparison. We found that tumors derived from CSRP2-overexpressing cells were dramatically larger and heavier than those derived from control cells (Fig. 2M and Supplementary Fig. S3I), and this was confirmed by H&E staining (Supplementary Fig. S3F, G). We also found that the ratio of Ki-67 positive cells was higher in tumors derived from CSRP2-overexpressing cells than that in tumors derived from control cells (Supplementary Fig. S3J, K), confirming that CSRP2 overexpression also promotes cell proliferation in vivo. The survival period of mice xenografted with CSRP2-overexpressed cells (median survival of 37 days) was shorter than that of the control group (median survival of 41.5 days) (Fig. 2N). Body weight statistics of mice also revealed that compared to controls, the CSRP2-overexpressing group lost weight dramatically at the late stage (Supplementary Fig. S3H).

### CSRP2 knockdown attenuates GBM progression

**Fig. 3:**
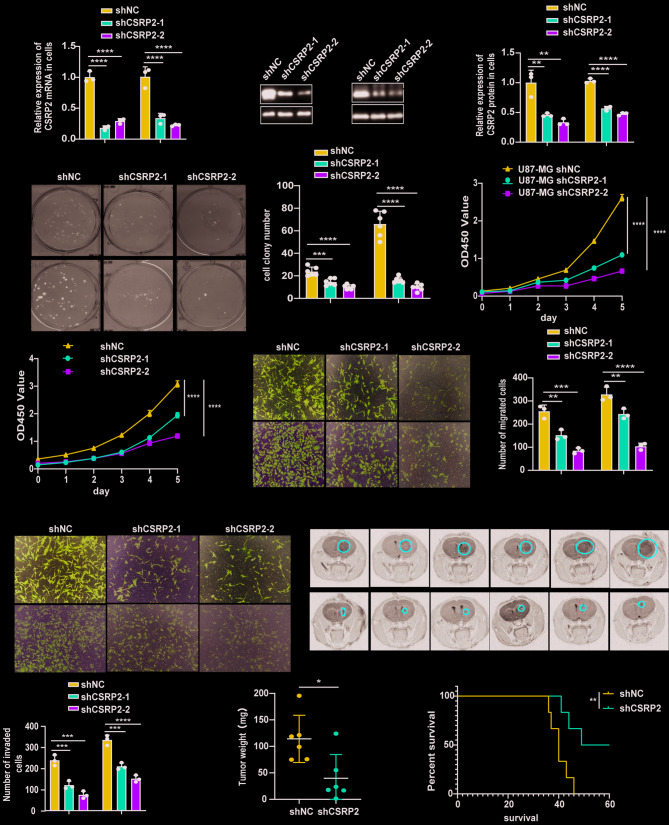
CSRP2 knockdown reduces the malignancy of GBM cells. (**A**) U87-MG and U251 cells were stably transduced with two *CSRP2* shRNA lentiviruses (shCSRP2-1 and shCSRP2-2) or control shRNA lentiviruses (shNC), and the mRNA levels of *CSRP2* were compared. One-way ANOVA with Tukey’s post hoc test, *n* = 3 per group. (**B**, **C**) CSRP2 protein levels in U87-MG and U251 cells stably transduced with CSRP2 shRNA and control shRNA lentiviruses were analyzed by western blotting (**B**) and densitometry quantification for comparison (**C**). One-way ANOVA with Tukey’s post hoc test, *n* = 3 per group. (**D**, **E**) U87-MG and U251 cells with CSRP2 knockdown and controls were assayed for colony formation (**D**) and the colony numbers were compared (**E**). One-way ANOVA with Tukey’s post hoc test, *n* = 6 per group. (**F**, **G**) U87-MG (**F**) and U251 (**G**) cells with CSRP2 knockdown and controls were assayed for their proliferation. Two-way ANOVA with Sidak’s post hoc test, *n* = 6 per group for U87-MG and U251. (**H**-**K**) U87-MG and U251 cells with CSRP2 knockdown were studied for their migration ability (**H**, **I**) and invasion ability (**J**, **K**). One-way ANOVA with Tukey’s post hoc test, *n* = 3 per group. (**L**, **M**) U87-MG cells with CSRP2 knockdown (shCSRP2) and controls (shNC) were xenografted into the mouse brain. Tumor formation was observed by MRI brain images (**L**) and tumor weights were compared (**M**). Red cycles indicate tumor areas. Unpaired t test, *n* = 6 per group. (**N**) Kaplan-Meier survival curves of xenografted mice. Log-rank test, *n* = 6 per group. Data represent mean ± SEM, *p < 0.05, **p < 0.01, ***p < 0.001, ****p < 0.0001

Subsequently, we infected U87-MG and U251 cells with lentiviruses expressing two CSRP2 shRNAs or control, and obtained stable cell lines after puromycin selection. qRT-PCR and western blotting analyses confirmed reduced CSRP2 expression in these cells (Fig. 3A-C). CSRP2 knockdown significantly inhibited colony formation (Fig. 3D, E), cell proliferation (Fig. 3F, G), cell migration (Fig. 3H, I), and cell invasion (Fig. 3J, K), and increased cell apoptosis (Supplementary Fig. S4A, B) and promoted the formation of apoptotic vesicles (Supplementary Fig. S4F). CSRP2 knockdown also decreased the levels of Bcl-2 and Bcl-xL, whereas increased the levels of cleaved PARP and cleaved Caspase-8 (Supplementary Fig. S4C-E).

To assess the effect of CSRP2 knockdown on tumor formation in vivo, we transplanted U87-MG cells with CSRP2 knockdown and control cells into the brains of nude mice. Approximately one month later, mice were scanned by MRI to confirm the presence of tumors (Fig. 3L). We subsequently dissected tumors from mice for comparison. We found that tumors derived from CSRP2-knockdown cells were markedly smaller and lighter than those derived from control cells (Fig. 3M and Supplementary Fig. S4J), and this was confirmed by H&E staining (Supplementary Fig. S4G, H). Furthermore, the ratio of Ki-67 positive cells was lower in tumors derived from CSRP2-knockdown cells than that in tumors derived from control cells (Supplementary Fig. S4K, L), confirming that CSRP2 knockdown also inhibits cell proliferation in vivo. Moreover, mice xenografted with CSRP2-knockdown cells (median survival of 55.5 days) had longer survival time than controls (median survival of 40 days) (Fig. 3N). Body weight statistics revealed that at the late stage when control mice started to lose body weight significantly, the CSRP2-knockdown group mice still had relatively stable body weight (Supplementary Fig. S4I).

### CSRP2 promotes PMT and regulates the NF-κB and the MAPK signaling pathways

**Fig. 4:**
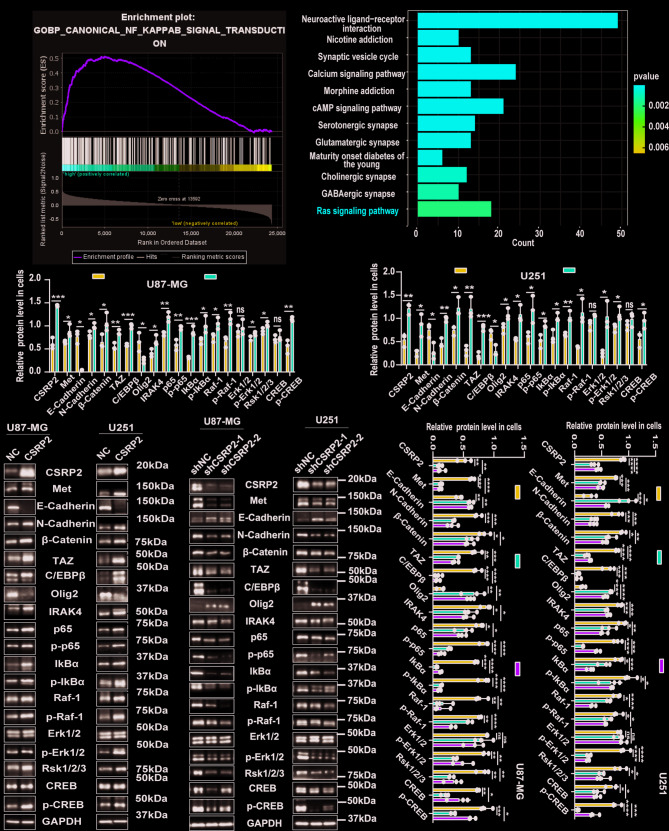
CSRP2 promotes PMT and the NF-κB and the MAPK signaling activities in GBM. (**A**) GSEA analysis of the correlation between CSRP2 expression and the NF-κB signaling pathway based on the CGGA data. (**B**) KEGG enrichment analysis of the correlation between CSRP2 expression and the Ras/MAPK signaling pathway based on the TCGA data. (**C-E**) Levels of PMT markers and NF-κB and MAPK signaling-related proteins in U87-MG and U251 cells with stable CSRP2 overexpression (CSRP2) and controls were analyzed by western blotting (**C**) and densitometry quantification comparison (**D**, **E**). Unpaired t test, *n* = 3 per group. (**F-H**) Levels of PMT markers and NF-κB and MAPK signaling-related proteins in U87-MG and U251 cells with stable CSRP2 knockdown and controls were analyzed by western blotting (**F**) and densitometry quantification comparison (**G, H**). One-way ANOVA with Tukey’s post hoc test, *n* = 3 per group. Data represent mean ± SEM, *p < 0.05, **p < 0.01, ***p < 0.001, ****p < 0.0001, ns: not significant

To explore the potential mechanisms by which CSRP2 promotes malignant progression in GBM, we performed GSEA using the CGGA data and found that CSRP2 was positively correlated with the NF-κB signaling pathway (Fig. 4A). KEGG enrichment analysis using the TCGA data revealed that CSRP2 may also affect the Ras/MAPK signaling pathway (Fig. 4B).

Both the NF-κB and the MAPK signaling pathways play important roles in promoting the malignant progression of GBM, including its PMT process [26, 50–52]. The NF-κB and the MAPK signaling pathways can also be interlinked and together exacerbate PMT and GBM malignant progression [26, 52, 53]. The PMT process is accompanied by a decrease in the protein levels of some proneural-type markers, such as E-Cadherin and Olig2 [54, 55], and an increase in the protein levels of markers of mesenchymal-type, such as Met, N-Cadherin, β-Catenin, TAZ, and C/EBPβ [54, 55]. Herein, we further analyzed whether and how CSRP2 regulates PMT via the NF-κB and/or the MAPK signaling pathways in GBM. We found that CSRP2 overexpression significantly increased the levels of Met, N-Cadherin, β-Catenin, TAZ, C/EBPβ, IRAK4, NF-κB/p65, phosphorylated (p-)NF-κB/p65, IκBα, phosphorylated (p-)-IκBα, Raf-1, phosphorylated (p-) Raf-1, phosphorylated (p-) Erk1/2, Rsk1/2/3, and phosphorylated (p-) CREB, and reduced the levels of E-Cadherin and Olig2 (Fig. 4C-E). While CSRP2 knockdown had opposite effects on the levels of these proteins (Fig. 4F-H). These results suggest that elevated CSRP2 promotes NF-κB and MAPK activities and PMT, thereby facilitating the malignant progression of GBM.

### NF-κB activity Inhibition suppresses the malignant effects of CSRP2 overexpression

**Fig. 5:**
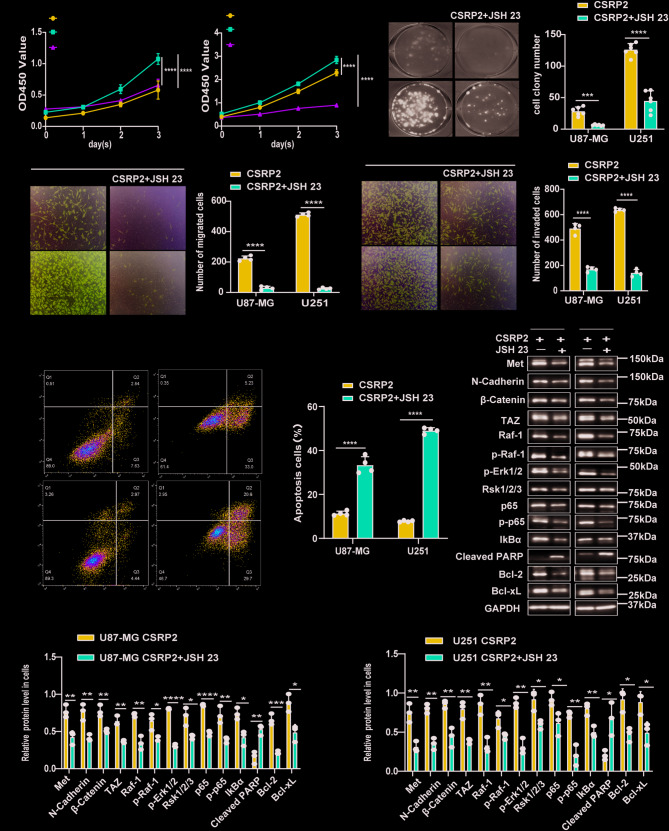
Inhibition of the NF-κB activity reduces the malignancy of GBM cells with CSRP2 overexpression and suppresses PMT and the MAPK activity. (**A, B**) U87-MG (**A**) and U251 (**B**) cells with stable CSRP2 expression (CSRP2) were treated with or without the NF-κB inhibitor JSH 23 (80 µM) for indicated time periods and cell proliferation was analyzed. Cells transfected with control vector (NC) were used as a negative control. Two-way ANOVA with Sidak’s post hoc test, *n* = 6 per group for U87-MG and U251. (**C, D**) U87-MG and U251 cells with stable CSRP2 overexpression were treated with 80 µM JSH 23 and analyzed for their colony formation ability (**C**) and colony numbers (**D**). Unpaired t test, *n* = 6 per group. (**E-H**) U87-MG and U251 cells with stable CSRP2 overexpression were treated with 80 µM JSH 23 and studied for their migration (**E, F**) and invasion (**G, H**) abilities. Unpaired t test, *n* = 4 per group. (**I, J**) U87-MG and U251 cells with stable CSRP2 overexpression were treated with 80 µM JSH 23 for 48 h and cell apoptosis was measured by flow cytometry (**I**) for apoptosis ratio comparison (**J**). Unpaired t test, *n* = 4 per group. (**K-M**) U87-MG and U251 cells with stable CSRP2 overexpression were treated with 80 µM JSH 23 for 48 h and equal amounts of cell lysates were subjected to western blotting (**K**) and quantification analysis (**L, M**) for PMT markers, NF-κB and MAPK signaling-related proteins, and apoptosis-related proteins. Unpaired t test, *n* = 3 per group. Data represent mean ± SEM, *p < 0.05, **p < 0.01, ***p < 0.001, ****p < 0.0001

Since the NF-κB signaling pathway is crucial for GBM malignant progression, we studied whether inhibiting this pathway can suppress the malignant effects of CSRP2 overexpression. When GBM cells stably overexpressing CSRP2 were treated with the NF-κB inhibitor JSH 23, cell proliferation (Fig. 5A, B), colony formation (Fig. 5C, D), cell migration (Fig. 5E, F), and cell invasion (Fig. 5G, H) were all reduced. JSH 23 treatment also significantly promoted apoptosis of these cells (Fig. 5I, J). Moreover, JSH 23 treatment dramatically reduced the protein levels of Met, N-Cadherin, β-Catenin, TAZ, Raf-1, p-Raf-1, p-Erk1/2, Rsk1/2/3, NF-κB/p65, p-NF-κB/p65, and IκBα (Fig. 5K-M), indicating that JSH 23 treatment not only inhibited the NF-κB signaling pathway, but also inhibited the MAPK signaling pathway and PMT.

### CSRP2 promotes PMT and the NF-κB and the MAPK signaling pathways in a p130Cas-dependent manner

**Fig. 6:**
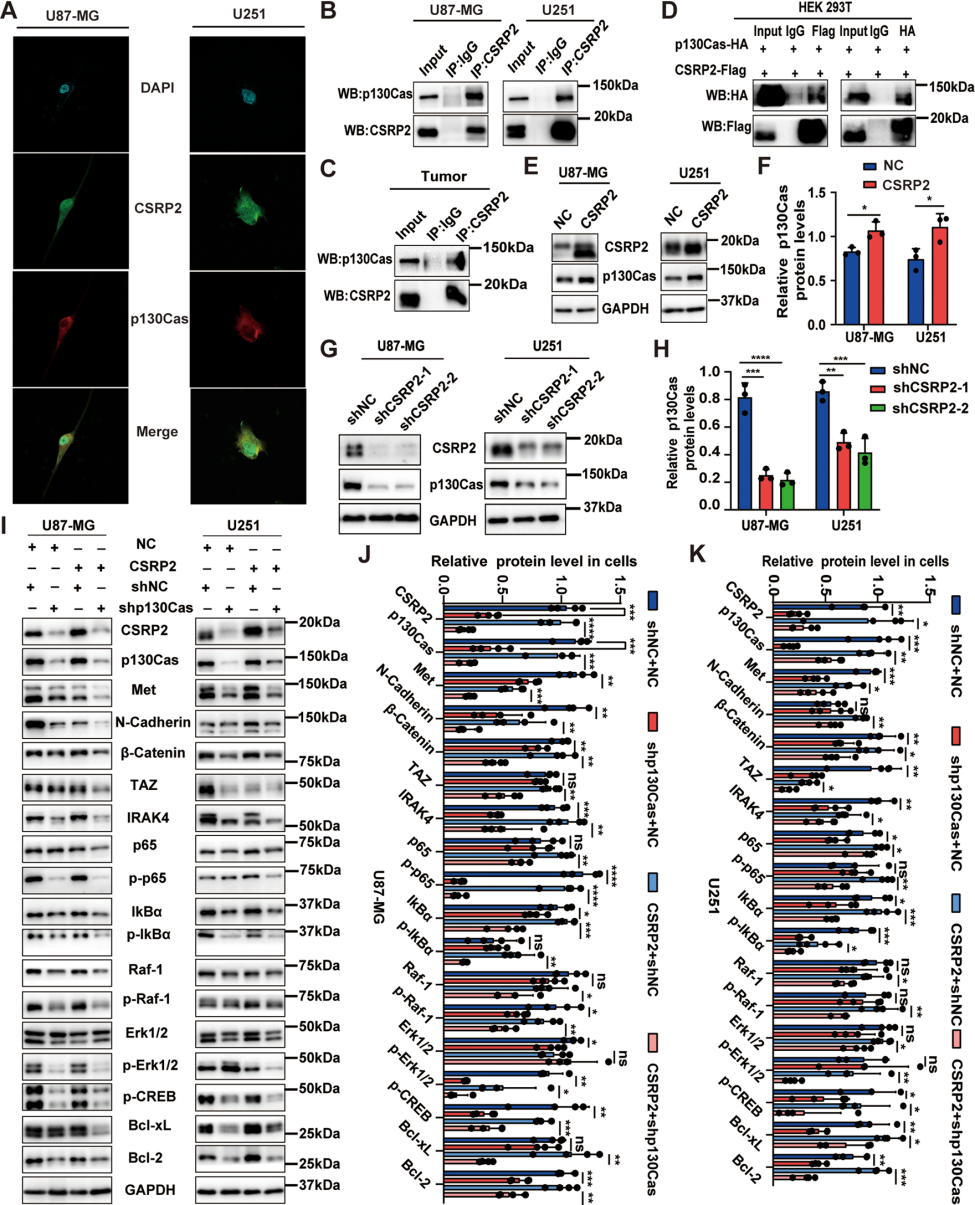
p130Cas interacts with CSRP2 and mediates CSRP2 functions. (**A**) U87-MG and U251 cells were immunostained with antibodies against CSRP2 (in green) and p130Cas (in red). The Nuclei were stained by DAPI (in blue). (**B, C**) Equal amounts of U87-MG and U251 cell lysates (**B**) or GBM tissue lysates (**C**) were immunoprecipitated (IP) with an anti-CSRP2 antibody and IgG and then immunoblotted (WB) with anti-CSRP2 and anti-p130Cas antibodies. (**D**) HEK 293T cells were co-transfected with CSRP2-Flag and p130Cas-HA vectors. Equal amounts of cell lysates were immunoprecipitated with anti-Flag and anti-HA antibodies and IgG and then immunoblotted with anti-Flag and anti-HA antibodies. (**E-H**) The p130Cas protein levels in U87-MG and U251 cells with CSRP2 overexpression (**E, F**) or CSRP2 knockdown (**G, H**) were analyzed by western blotting (**E, G**) and quantified for comparison (**F, H**). Unpaired t test for (**F**) and One-way ANOVA with Tukey’s post hoc test for (**H**), *n* = 3 per group. (**I-K**) U87-MG and U251 cells with stable CSRP2 overexpression and controls (NC) were transfected with p130Cas shRNA (shp130Cas) or control shRNA (shNC) and equal amounts of protein lysates were subjected to western blotting (**I**) and quantification comparison (**J, K**) for PMT-, apoptosis-, and NF-κB and MAPK signaling-related proteins. Unpaired t test, *n* = 4 per group. Data represent mean ± SEM, *p < 0.05, **p < 0.01, ***p < 0.001, ****p < 0.0001, ns: not significant

We further explored how CSRP2 promotes PMT and activates the NF-κB and the MAPK signaling pathways. The p130Cas protein is known to mediate the NF-κB signaling pathway for regulating bone homeostasis [56] and activate the MAPK signaling to promote breast cancer invasion [57]. Since it was reported that CSRP2 interacted with p130Cas in mouse vascular smooth muscle and in colorectal cancer [17, 58], we explored whether CSRP2 interacts with p130Cas in GBM. Immunofluorescence assays detected co-localization of endogenous CSRP2 with p130Cas in GBM cells (Fig. 6A). Co-immunoprecipitation assays ascertained the interaction between endogenous CSRP2 and p130Cas in GBM cells and tissues (Fig. 6B, C), and between exogenous CSRP2 and p130Cas when they were co-expressed in HEK 293T cells (Fig. 6D). In addition, we found that CSRP2 overexpression increased p130Cas protein levels in GBM cells (Fig. 6E, F), whereas CSRP2 knockdown decreased p130Cas protein levels (Fig. 6G, H). Importantly, in cells stably overexpressing CSRP2, p130Cas downregulation reversed PMT by decreasing the protein levels of Met, N-Cadherin, β-Catenin, and TAZ, reversed elevated NF-κB signaling activity by decreasing the protein levels of IRAK4, NF-κB/p65, p-NF-κB/p65, IκBα, and p-IκBα, reversed elevated MAPK signaling activity by decreasing the protein levels of Raf-1, p-Raf-1, p-Erk1/2, and p-CREB, and increased apoptosis by decreasing the protein levels of Bcl-xL and Bcl-2 (Fig. 6I-K). These results suggest that increased CSRP2 promotes p130Cas to activate the NF-κB and the MAPK signaling pathways and PMT, and this can be reversed by knocking down p130Cas.

### CSRP2 promotes Temozolomide (TMZ) resistance in GBM

**Fig. 7:**
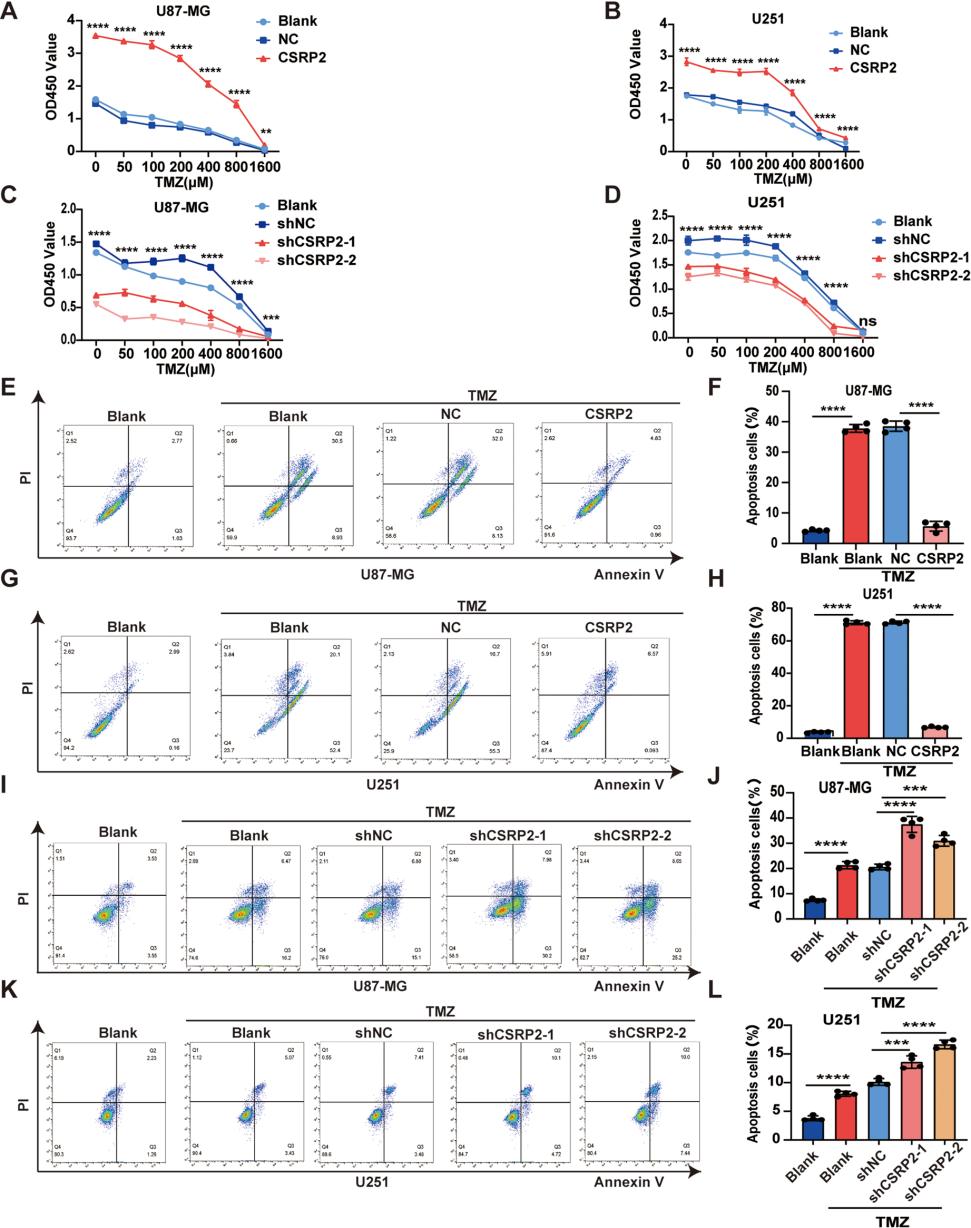
CSRP2 promotes glioma cell resistance to TMZ. (**A-D**) U87-MG (**A, C**) and U251 (**B, D**) cells with CSRP2 overexpression (**A, B**) or with CSRP2 knockdown (**C, D**) and respective control cells were treated with indicated amounts of TMZ for 48 h, and then analyzed for cell proliferation. Two-way ANOVA with Sidak’s post hoc test, *n* = 5 per group. (**E-H**) U87-MG (**E, F**) and U251 (**G, H**) cells with stable CSRP2 overexpression and control cells were treated with 200 µM TMZ for 48 h. Cell apoptosis was measured by flow cytometry (**E, G**) for apoptosis ratio comparison (**F, H**). Unpaired t test, *n* = 4 per group. (**I-L**) U87-MG (**I, J**) and U251 (**K, L**) cells with stable CSRP2 knockdown and control cells were treated with 200 µM TMZ for 48 h. Cell apoptosis was measured by flow cytometry (**I, K**) for apoptosis ratio comparison (**J, L**). One-way ANOVA with Tukey’s post hoc test, *n* = 4 per group. Data represent mean ± SEM, **p < 0.01, ***p < 0.001, ****p < 0.0001, ns: not significant

TMZ resistance in GBM is an important contributor to high mortality rates, and the molecular mechanisms underlying TMZ resistance have yet to be completely elucidated [59]. To investigate whether CSRP2 is associated with TMZ resistance in GBM, we treated CSRP2-overexpressing or CSRP2-knockdown cells with different concentrations of TMZ. TMZ treatment dose-dependently reduced proliferation in all cell groups (Fig. 7A-D). However, the proliferation of CSRP2-overexpressing cells was more resistant to TMZ treatment compared to controls (Fig. 7A, B), whereas the proliferation of CSRP2-knockdown cells was more sensitive to TMZ treatment compared to controls (Fig. 7C, D). In addition, CSRP2-overexpressing cells exhibited less apoptosis in response to TMZ treatment compared to control cells (Fig. 7E-H). In contrast, CSRP2-knockdown cells exhibited more apoptosis in response to TMZ treatment than control cells (Fig. 7I-L).

It has been shown that radiotherapy and chemotherapy induce TMZ resistance by promoting the phenotypic shift from PN to MES [60]. Since CSRP2 promotes the PMT process in GBM, we also investigated the expression of CSRP2 and the mechanism of drug resistance in a TMZ-resistant cell line, U87-MG T3rd. We treated this cell line and a TMZ-sensitive cell line, U87-MG S, with TMZ, and found that the IC50 of U87-MG T3rd (771.6 µM) was much higher than that of U87-MG S (166.1 µM) (Supplementary Fig. S5A), confirming the TMZ resistance of U87-MG T3rd cells. We further found that compared to U87-MG S cells, U87-MG T3rd cells exhibited significantly increased levels of CSRP2 and p130Cas, along with increased levels of Met, N-Cadherin, β-Catenin, TAZ, C/EBPβ, Raf-1, p-Raf-1, p-Erk1/2, Rsk1/2/3, IRAK4, NF-κB/p65, p-NF-κB/p65, p-IκBα, Bcl-2, and Bcl-xL (Supplementary Fig. S5B, C), suggesting that the TMZ resistance of U87-MG T3rd cells is associated with elevated CSRP2 and p130Cas, promoted PMT process, and activated NF-κB and MAPK signaling pathways. Moreover, we showed that knockdown of CSRP2 in U87-MG T3rd cells (Supplementary Fig. S6A, B) also significantly inhibited cell proliferation (Supplementary Fig. S6C) and colony formation (Supplementary Fig. S6E, F), and increased cell sensitivity to TMZ treatment (Supplementary Fig. S6D) and apoptosis (Supplementary Fig. S6G, H). While overexpression of CSRP2 (Supplementary Fig. S6I, J) had the opposite effects (Supplementary Fig. S6K-P). Together, these results further indicate that increased CSRP2 exacerbates GBM resistance to TMZ through promoting the PMT process and activating the NF-κB and the MAPK signaling pathways.

### Mitoxantrone (MTO) inhibits the malignant function of CSRP2 in GBM

**Fig. 8:**
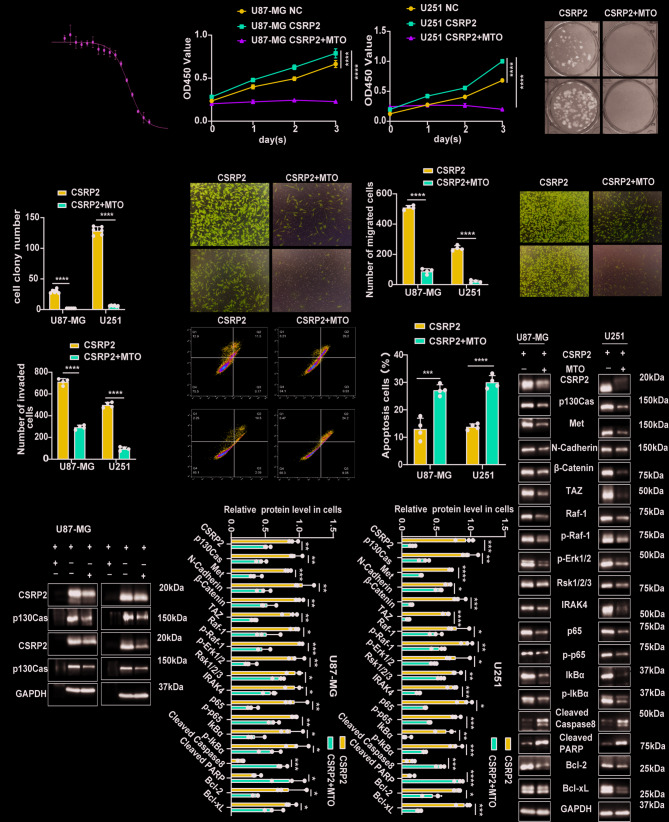
Mitoxantrone binds CSRP2 and inhibits the malignant function of CSRP2 in GBM. (**A**) Fluorescence-labeled CSRP2 recombinant protein was incubated with different concentrations of mitoxantrone (MTO) and measured for their binding using the microscale thermophoresis assay. (**B, C**) U87-MG (**B**) and U251 (**C**) cells with stable CSRP2 overexpression (CSRP2) were treated with or without 0.5 µM MTO for indicated time periods and the cell proliferation was analyzed. Cells transfected with control vector (NC) were used as a negative control. Two-way ANOVA with Sidak’s post hoc test, *n* = 6 per group for U87-MG and U251. (**D, E**) U87-MG and U251 cells with stable CSRP2 overexpression were treated with 0.5 µM MTO and analyzed for their colony formation ability (**D**) and colony numbers (**E**). Unpaired t test, *n* = 6 per group. (**F-I**) U87-MG and U251 cells with stable CSRP2 overexpression were treated with 0.5 µM MTO and studied for their migration (**F, G**) and invasion (**H, I**) abilities. Unpaired t test, *n* = 4 per group. (**J, K**) U87-MG and U251 cells with stable CSRP2 overexpression were treated with 0.5 µM MTO for 48 h and cell apoptosis was measured by flow cytometry (**J**) for apoptosis ratio comparison (**K**). Unpaired t test, *n* = 4 per group. (**L**) U87-MG and U251 cells with stable CSRP2 overexpression were treated with 0.5 µM MTO for 48 h. Equal amounts of cell lysates were immunoprecipitated (IP) with anti-CSRP2 and anti-p130Cas antibodies and IgG and then immunoblotted with anti-CSRP2 and anti-p130Cas antibodies. (**M-O**) U87-MG and U251 cells with stable CSRP2 overexpression were treated with 0.5 µM MTO for 48 h and equal amounts of cell lysates were subjected to western blotting (**M**) and quantification analysis (**N, O**) for PMT markers, NF-κB and MAPK signaling-related proteins, and apoptosis-related proteins. Unpaired t test, *n* = 3 per group. Data represent mean ± SEM, *p < 0.05, **p < 0.01, ***p < 0.001, ****p < 0.0001

Finally, we explored whether there are small molecule drugs that can bind and affect the function of CSRP2. We virtually screened drugs that bind CSRP2 and identified mitoxantrone (MTO) as a candidate. MTO is a topoisomerase inhibitor used in the treatment of various tumors and intratumoral injection of MTO can improve the survival of GBM patients [38–40]. The antitumor effects of MTO may be associated with the NF-κB and the MAPK signaling pathways but the exact mechanisms remain unclear [41, 42]. We then verified the interaction between MTO and CSRP2 using the microscale thermophoresis (MST) assay, which showed that MTO dose-dependently bound to fluorescence-labeled CSRP2 recombinant protein, with a Kd of 0.37 µM (Fig. 8A). Next, we treated GBM cells stably overexpressing CSRP2 with MTO and found that MTO treatment significantly inhibited cell proliferation (Fig. 8B, C), colony formation (Fig. 8D, E), migration (Fig. 8F, G), and invasive ability (Fig. 8H, I), and increased cell apoptosis (Fig. 8J, K). Moreover, we found that the interaction between CSRP2 and p130Cas was dramatically reduced after MTO treatment (Fig. 8L). MTO treatment also significantly reduced the levels of CSRP2, p130Cas, Met, N-Cadherin, β-Catenin, TAZ, Raf-1, p-Raf-1, p-Erk1/2, Rsk1/2/3, IRAK4, NF-κB/p65, p-NF-κB/p65, IκBα, p-IκBα, Bcl-2, and Bcl-xL (Fig. 8M-O). These results suggest that MTO binds CSRP2 and inhibits its malignant function in GBM.

## Discussion

CSRP2 belongs to a group of short LIM structural domain proteins that are key regulators of cell development and differentiation [61]. Multiple studies have reported that CSRP2 is associated with a variety of tumors but exhibits different functions [16, 17, 62–65]. Knockdown of CSRP2 significantly inhibited the invasion of breast cancers [62], the proliferation of desmoid tumors [64], and the migration and invasive ability of head and neck squamous cell carcinoma cells [65], and increased drug sensitivity in acute lymphoblastic leukemia [15]. CSRP2 expression was also found increased in hepatocellular carcinoma [63]. However, knockdown of CSRP2 promoted the malignant progression of gastric and colorectal cancers [16, 17]. Therefore, the exact mechanisms underlying the role of CSRP2 in various cancers need to be scrutinized. Very recently, two studies reported that CSRP2 could maintain the malignant phenotypes of gliomas through the Notch signaling pathway and inhibit glioma necroptosis through activating the JAK-STAT1 pathway [18, 19]. In this study, we confirmed that CSRP2 expression significantly increased in GBM and that high CSRP2 expression possibly associated with poor prognosis of GBM patients. Furthermore, we found that CSRP2 could be used as a predictor of the risk of survival or death in GBM patients. We also carried out functional study and demonstrated that CSRP2 overexpression significantly promoted proliferation, colony formation, migration, and invasion, and reduced apoptosis in GBM cells. GBM cells stably overexpressing CSRP2 formed larger and heavier tumors than control cells during intracerebral xenografts in mice, resulting in reduced survival. On the other hand, knockdown of CSRP2 reduced the malignancy of GBM cells both in vitro and in vivo. Moreover, we showed that CSRP2 overexpression conferred GBM cell resistance to TMZ, whereas CSRP2 knockdown made cells more sensitive to TMZ. Taken together, these results indicate that CSRP2 is a potential biomarker and therapeutic target for GBM.

PMT is a key mechanism contributing to GBM invasion, radiotherapy resistance, and poor prognosis. During GBM progression or radiotherapy treatment, PMT can make GBM cells to lose cell polarity and intercellular adhesion to enhance their invasive capacity, resulting in a mesenchymal and more motile phenotype [60]. Herein, we found that overexpression of CSRP2 increased the protein levels of mesenchymal-type markers and decreased the protein levels of proneural-type markers in GBM cells, whereas knockdown of CSRP2 had the opposite effects. These results suggest that CSRP2 can promote PMT in GBM cells.

Mechanistically, we found that CSRP2 could interact with p130Cas to regulate the NF-κB and the MAPK signaling pathways. p130Cas is a versatile scaffolding molecule containing a Src homology 3 (SH3) structural domain, a proline-rich structural domain, and a substrate structural domain with a sequence of 15 YxxP repeats (YxxP 15) [66]. Increasing evidence suggests that p130Cas and its interactions with other proteins can play a key role in tumor progression by enhancing cell migration and invasion, promoting tumor chemoresistance and maintaining intercellular mesenchymal morphology [67–69]. A study in hepatocellular carcinoma found that elevated levels of p130Cas associated with more advanced disease and increased lymph node invasion, and that p130Cas expression also associated with elevated levels of β-Catenin and decreased E-Cadherin expression [67]. In breast cancer, upregulation of p130Cas could promote resistance to the estrogen receptor antagonist tamoxifen [68]. In addition, it was found that in the highly aggressive human glioblastoma cell line U87-MG, knockdown of p130Cas resulted in the transformation of cells from an elongated mesenchymal morphology to a rounded amoeboid morphology, suggesting that p130Cas plays a role in the maintenance of polarized mesenchymal morphology in U87-MG cells [69]. Previous studies noticed the interaction between CSRP2 and p130Cas in mouse vascular smooth muscle and in colorectal cancer [17, 58]. Herein, we also found that CSRP2 interacted with p130Cas in GBM. More importantly, we showed that overexpression and knockdown of CSRP2 increased and decreased p130Cas levels, respectively; while p130Cas knockdown rescued CSRP2 overexpression-induced GBM malignancy and PMT. These results indicate that p130Cas mediates the malignant function of CSRP2 in GBM.

GSEA profiling revealed a positive correlation between CSRP2 expression and the NF-κB signaling pathway in GBM. The contribution of aberrant activation of NF-κB signaling in GBM, including its PMT process has been documented [70]. For example, activation of the NF-κB signaling was found to promote PMT in glioblastoma stem cells (GSCs), contributing to GBM malignant progression [71, 72], whereas its blockade in patient-derived GSCs resulted in increased sensitivity of GSCs to radiotherapy [26]. In addition, it was reported that p130Cas also correlated with NF-κB signaling [56]. Herein, we found that CSRP2 overexpression promoted, whereas CSRP2 knockdown reduced NF-κB activity. Furthermore, we found that inhibition of NF-κB activity reversed the increased malignancy of GBM caused by CSRP2 overexpression. These findings further suggest that CSRP2 promotes PMT in GBM by enhancing the NF-κB signaling pathway.

KEGG profiling of genes differentially expressed in CSRP2-high and CSRP2-low groups revealed a positive correlation between CSRP2 expression and the MAPK signaling pathway in GBM. The MAPK signaling pathway is one of the most dysregulated pathways in human cancers including gliomas [51], and its aberrant activation may enhance resistance to TMZ [52]. The MAPK signaling and the NF-κB signaling synergistically promote glioma cell invasion and are cross-linked in tumors [22, 36, 37, 73]. In addition, p130Cas was reported to promote breast cancer invasion by activating the Erk1/2 MAPK signaling pathway [57]. Therefore, we also studied the effects of CSRP2 on the MAPK signaling pathway. As expected, CSRP2 overexpression promoted the MAPK signaling activity, whereas CSRP2 knockdown had the opposite effect. Inhibition of the NF-κB activity also inhibited the MAPK signaling pathway. These results suggest that CSRP2 promotes PMT in GBM also by enhancing the MAPK signaling pathway.

We further virtually screened and identified MTO as a CSRP2-binding molecule. The binding between MTO and CSRP2 was confirmed by the microscale thermophoresis (MST) assay. MTO is a topoisomerase inhibitor used in the treatment of multiple tumors [38–40]. Interestingly, some studies found that the antitumor effects of MTO are associated with the NF-κB and MAPK signaling pathways. For example, Inhibition of the MAPK signaling pathway increases the inhibitory effect of MTO on leukemia cells [41]. Knockdown breast cancer resistance protein (BCRP) increases sensitivity of breast cancer cells to MTO by inhibiting the NF-κB activity [42]. Therefore, we hypothesized that MTO binding to CSRP2 could inhibit its biological function in GBM. As expected, we found that in GBM cells overexpressing CSRP2, treatment with MTO significantly inhibited cell proliferation, colony formation, migration, and invasion, and increased cell apoptosis. Moreover, MTO treatment significantly inhibited the interaction between CSRP2 and p130Cas and suppressed the PMT process and the NF-κB and the MAPK signaling activities. These results reveal a novel mechanism by which MTO inhibits GBM malignancy.

## Conclusions

In summary, our study not only confirms the elevation of CSRP2 expression in GBM, but also demonstrates that CSRP2 promotes GBM malignancy, including its PMT and TMZ resistance through interacting with p130Cas, thereby activating the NF-κB and the MAPK signaling pathways. Moreover, we reveal that MTO exerts its anti-GBM function through binding CSRP2 to inhibit the CSRP2/p130Cas interaction and thus the downstream signaling pathways.

## Electronic supplementary material

Below is the link to the electronic supplementary material.

Supplementary Material 1

Supplementary Material 2

Supplementary Material 3

## Data Availability

No datasets were generated or analysed during the current study.

## Abbreviations

BCRP: breast cancer resistance protein
CGGA: China Glioma Genome Atlas
CL: classical
co-IP: co-immunoprecipitation
CSRP2: Cysteine-rich protein 2
DAPI: 4-amino-6-diamino-2-phenylindole
ECM: extracellular matrix
EMT: epithelial-mesenchymal transition
FITC: fluorescein isothiocyanate
GBM: glioblastoma multiforme
GEPIA: Gene Expression Profiling Interaction Analysis
GSCs: glioblastoma stem cells
GSEA: gene set enrichment analysis
IDH: isocitrate dehydrogenase
IF: immunofluorescence
KEGG: Kyoto Encyclopedia of Genes and Genomes
LGG: low-grade glioma
MES: mesenchymal
MRI: magnetic resonance imaging
MST: Microscale thermophoresis
MTO: mitoxantrone
NHA: normal human astrocyte
NL: neural
PI: propidium iodide
PLGG: pediatric low grade gliomas
PMT: proneural-to-mesenchymal transition
PN: proneural
qRT–PCR: Quantitative real-time PCR
ROC: Receiver Operating Characteristic curve
RTK: receptor tyrosine kinase
SH3: Src homology 3
TCGA: The Cancer Genome Atlas Databases
TMZ: temozolomide
WB: western blotting
WHO: World Health Organization
