# Supplementary material for: CSRP2 promotes the glioblastoma mesenchymal phenotype via p130Cas-mediated NF-κB and MAPK pathways
Source: J Exp Clin Cancer Res. 2025 Aug 5;44:228. doi: 10.1186/s13046-025-03484-7 (PMC12323131; doi:10.1186/s13046-025-03484-7)
Supplement: Supplementary file 1 — Supplementary Material 1 [file 13046_2025_3484_MOESM1_ESM.pdf]

CSRP2 promotes the glioblastoma mesenchymal phenotype via p130Cas-mediated NF-κB and MAPK pathways

Jiawei He et al.

Supplementary Figures S1

Supplementary Figure S1. Original Western blotting data. WB bands in the red box are representative images used in text figures and other supplementary figures. WB bands in the green box are those used for quantification and statistical analysis.

Full unedited gel for Figure 1H

Green： Statistical graph

Red： Representative graph

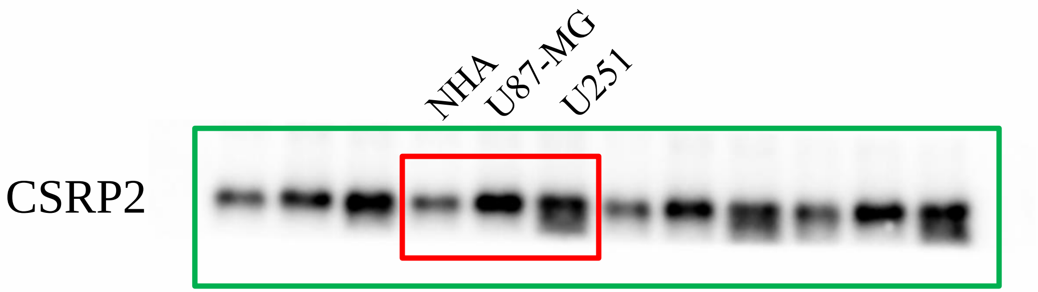

The membrane was imaged with Azure Biosystems 300

Full unedited gel for Figure 1H

Green： Statistical graph

Red： Representative graph

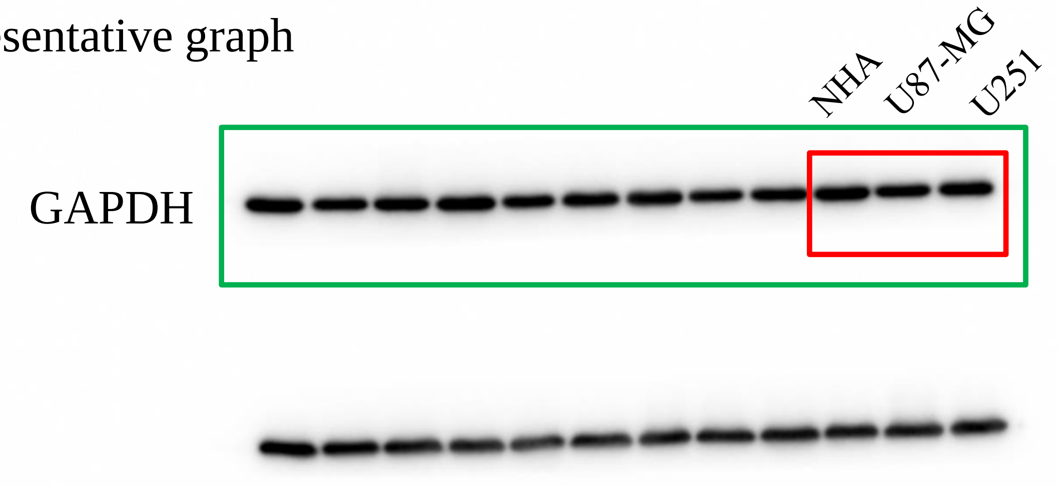

The membrane was imaged with Azure Biosystems 300

Full unedited gel for Figure 1K

Red： Statistical graph and representative graph

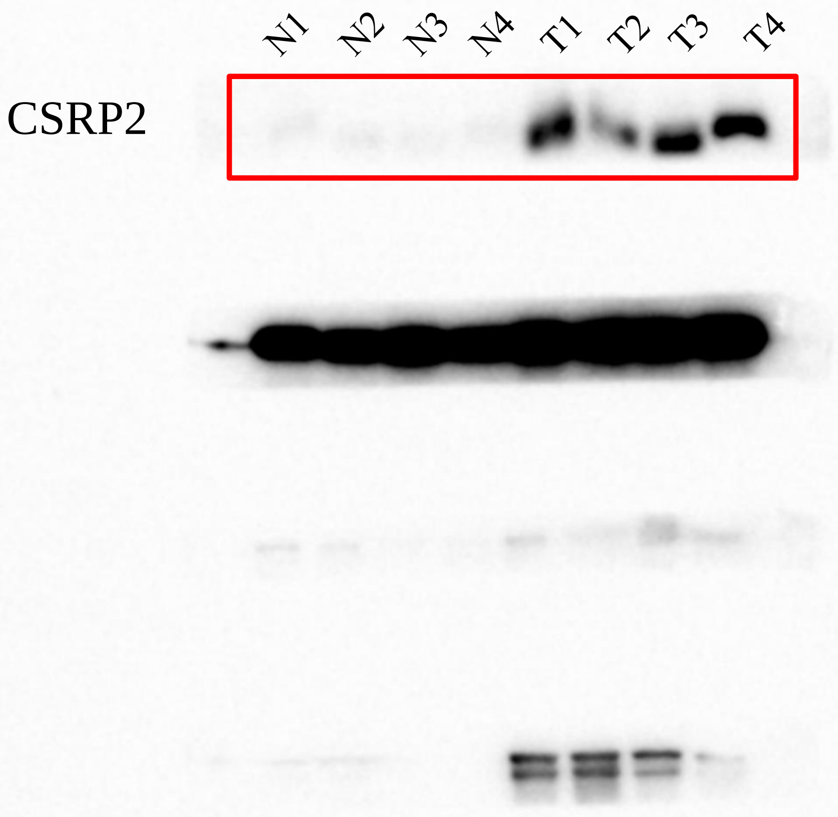

The membrane was imaged with Azure Biosystems 300

Full unedited gel for Figure 1K

Red： Statistical graph and representative graph

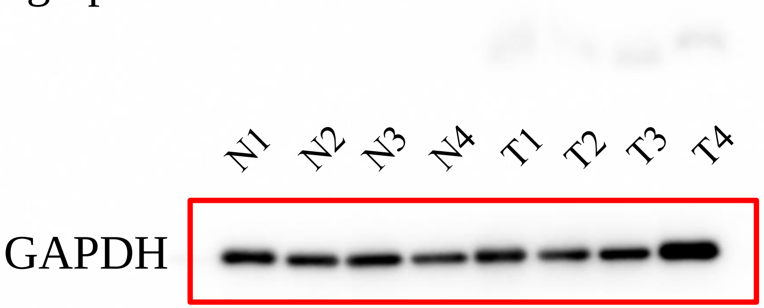

The membrane was imaged with Azure Biosystems 300

Full unedited gel for Figure 2B

Green： Statistical graph

Red： Representative graph

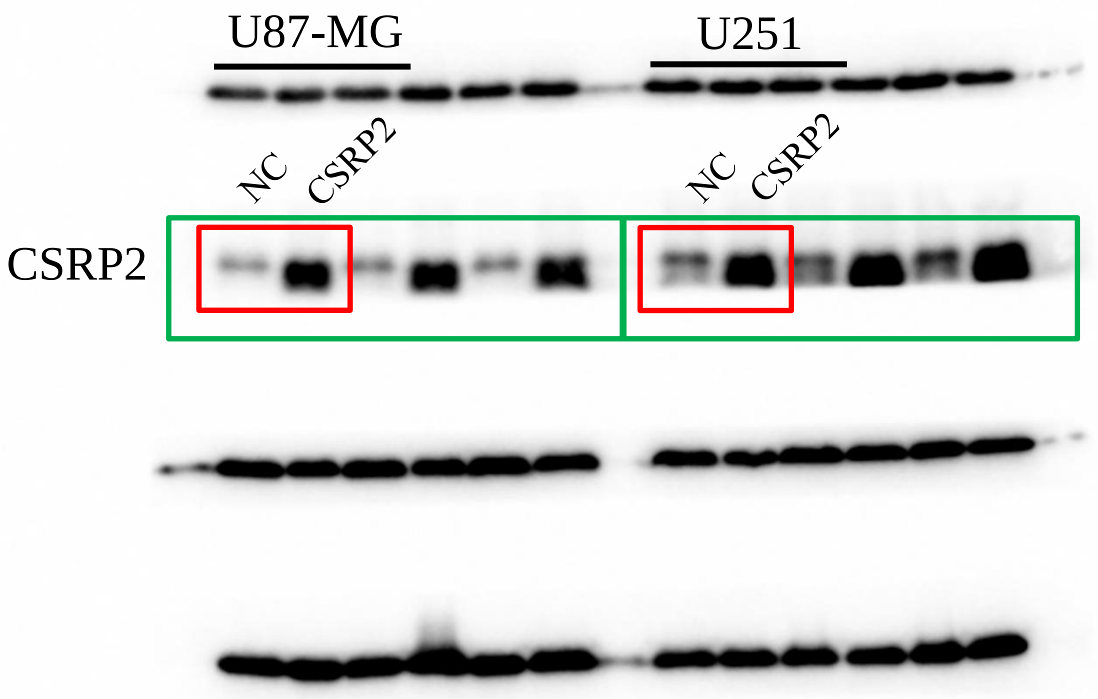

The membrane was imaged with Azure Biosystems 300

Full unedited gel for Figure 2B

Green： Statistical graph

Red： Representative graph

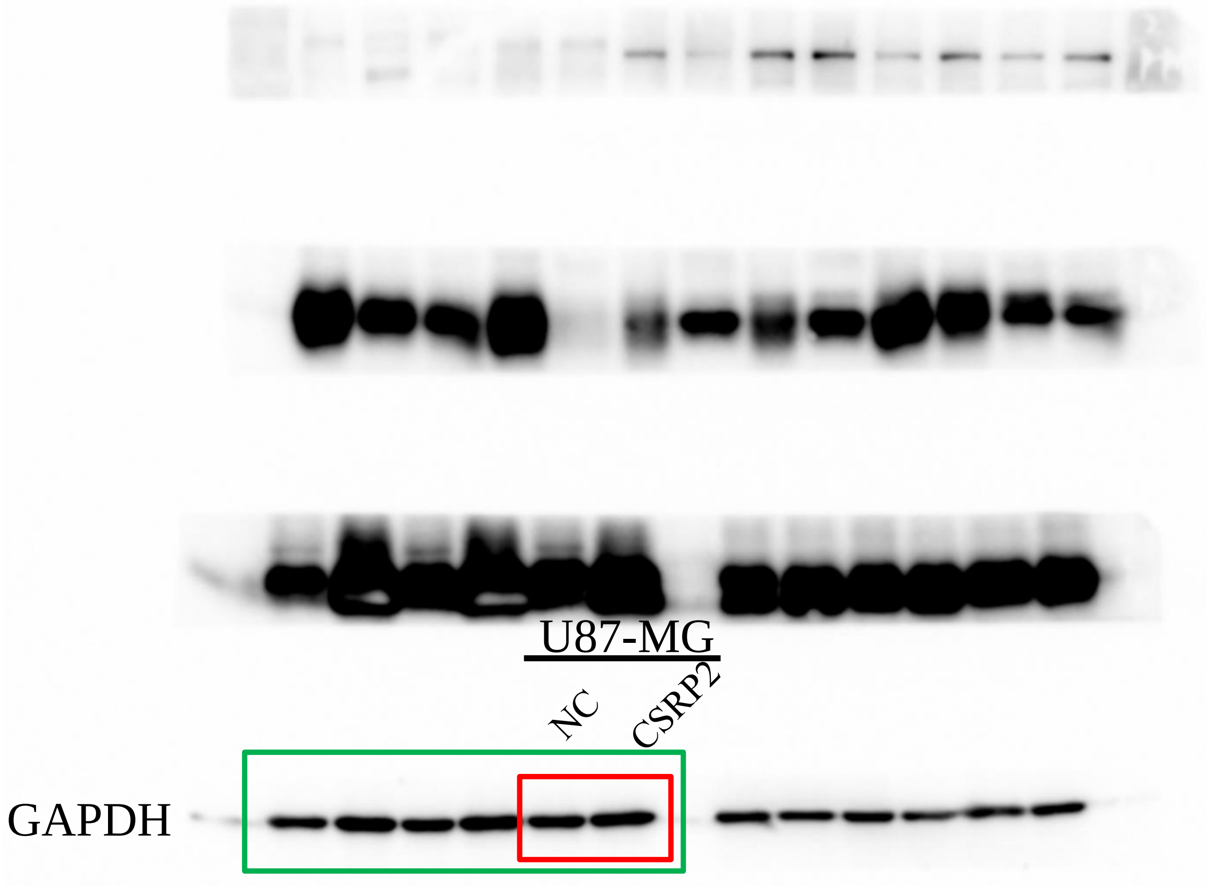

The membrane was imaged with Azure Biosystems 300

Full unedited gel for Figure 2B

Green： Statistical graph

Red： Representative graph

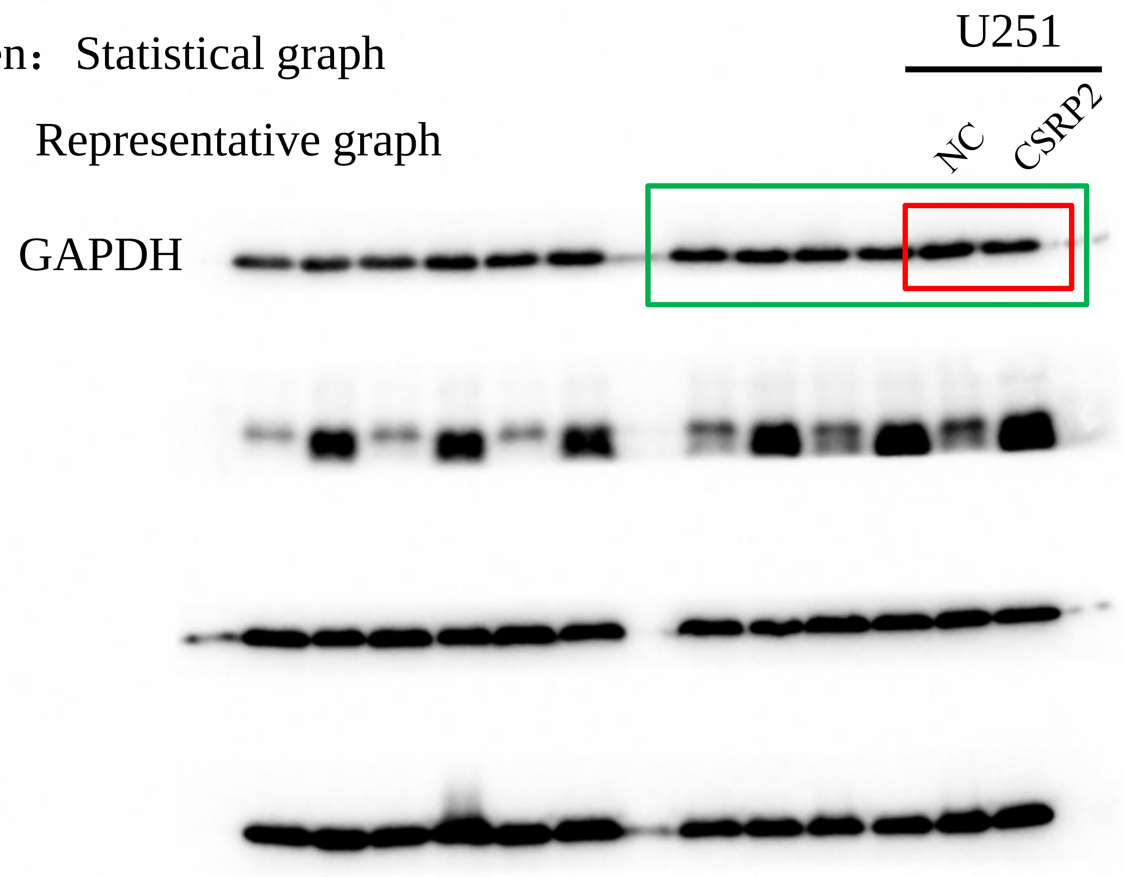

The membrane was imaged with Azure Biosystems 300

Full unedited gel for Figure 3B

Green： Statistical graph

Red： Representative graph

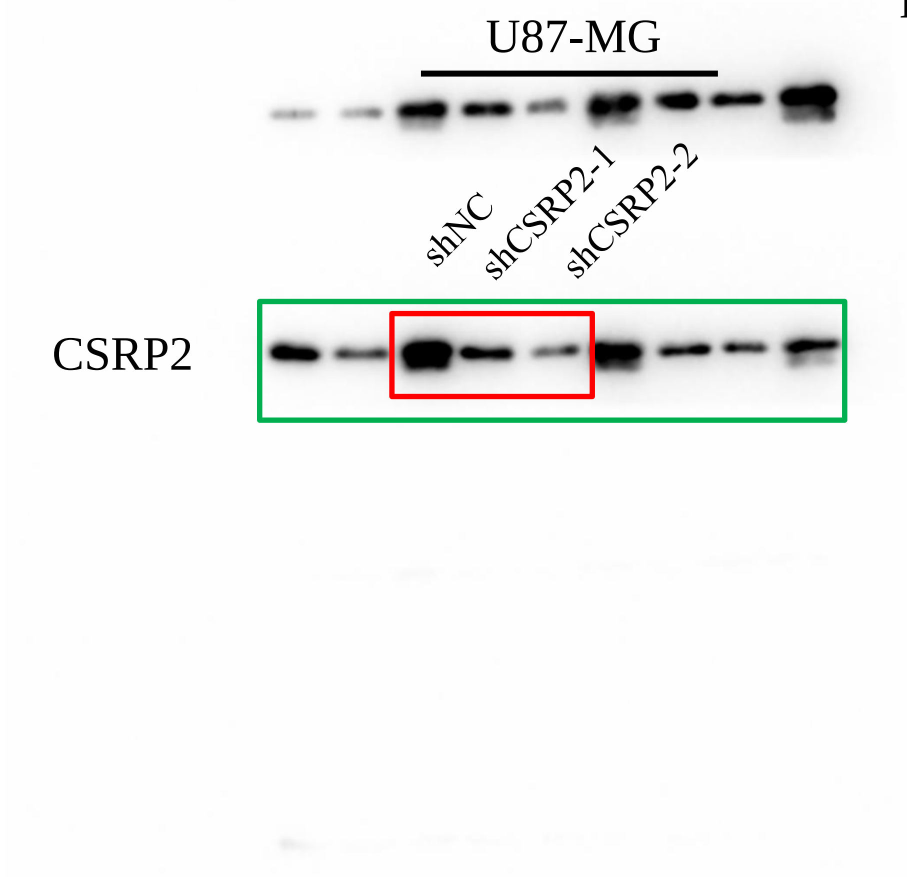

The membrane was imaged with Azure Biosystems 300

Full unedited gel for Figure 3B

Green： Statistical graph

Red： Representative graph

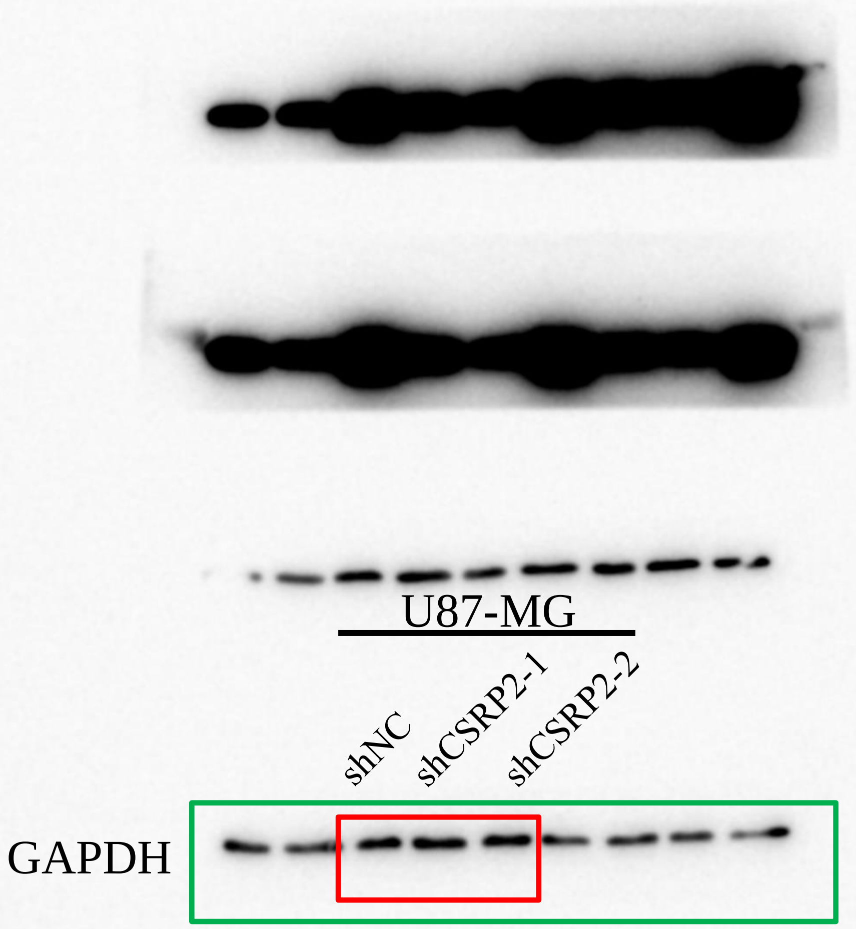

The membrane was imaged with Azure Biosystems 300

Full unedited gel for Figure 3B

Green： Statistical graph

Red： Representative graph

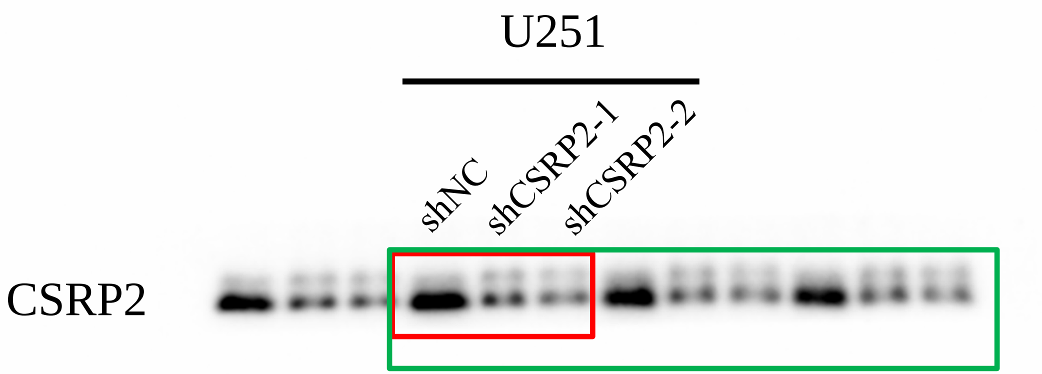

The membrane was imaged with Azure Biosystems 300

Full unedited gel for Figure 3B

Green： Statistical graph

Red： Representative graph

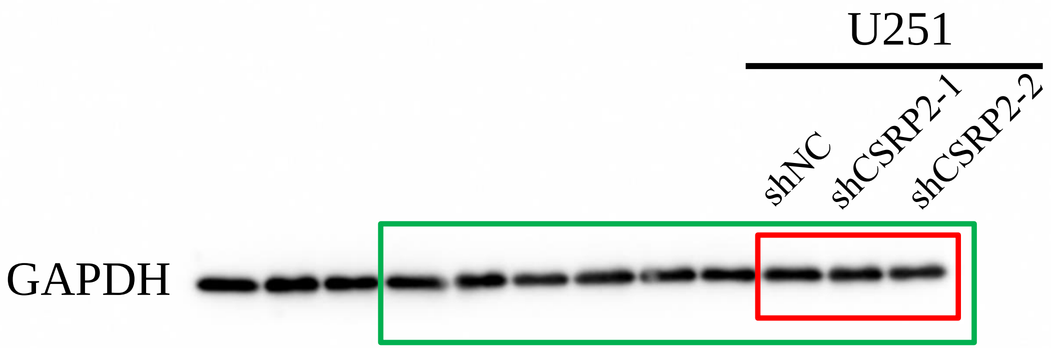

The membrane was imaged with Azure Biosystems 300

Full unedited gel for Figure 4C

Green： Statistical graph

Red： Representative graph

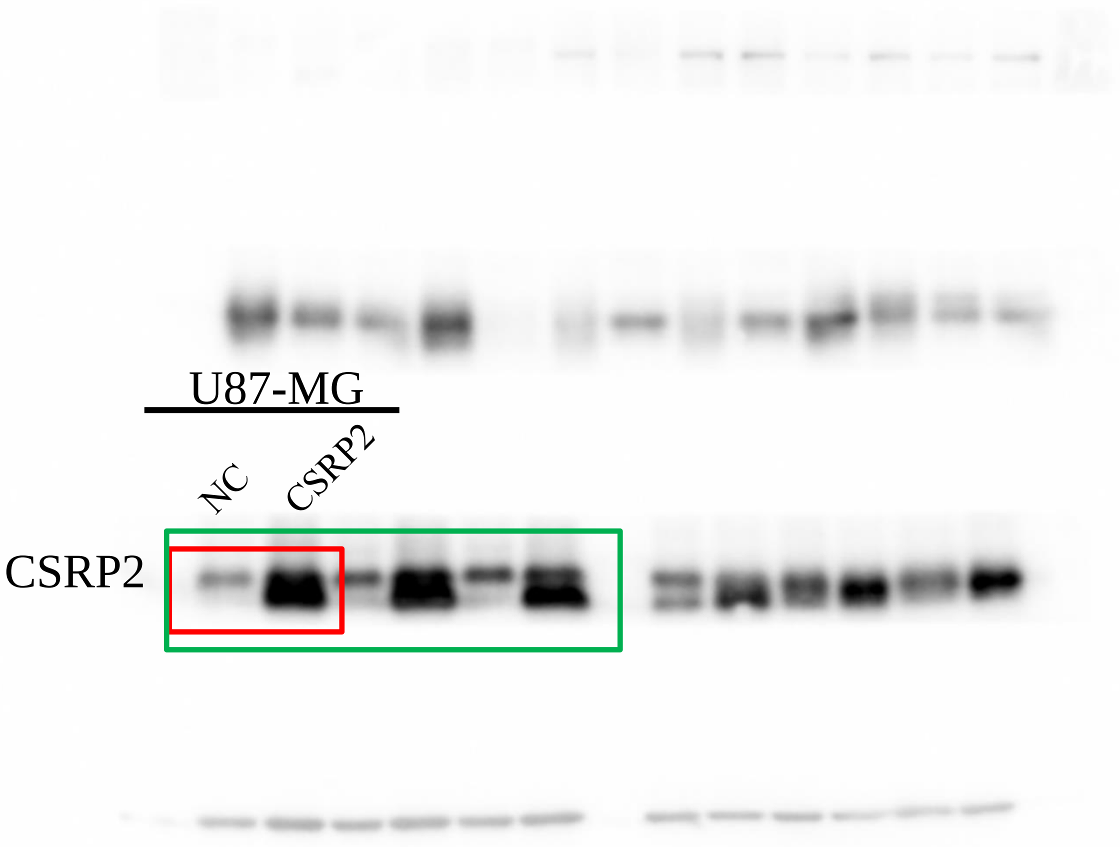

The membrane was imaged with Azure Biosystems 300

Full unedited gel for Figure 4C

Green： Statistical graph

Red： Representative graph

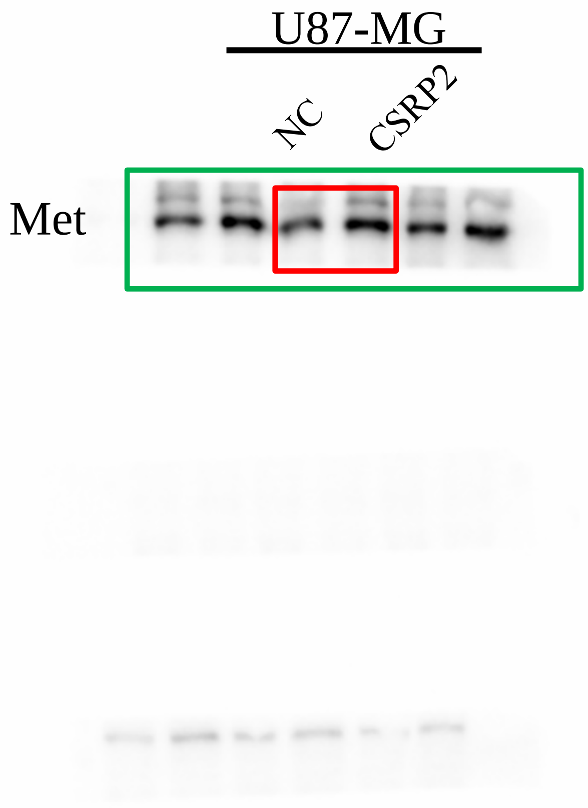

The membrane was imaged with Azure Biosystems 300

Full unedited gel for Figure 4C

Green： Statistical graph

Red： Representative graph

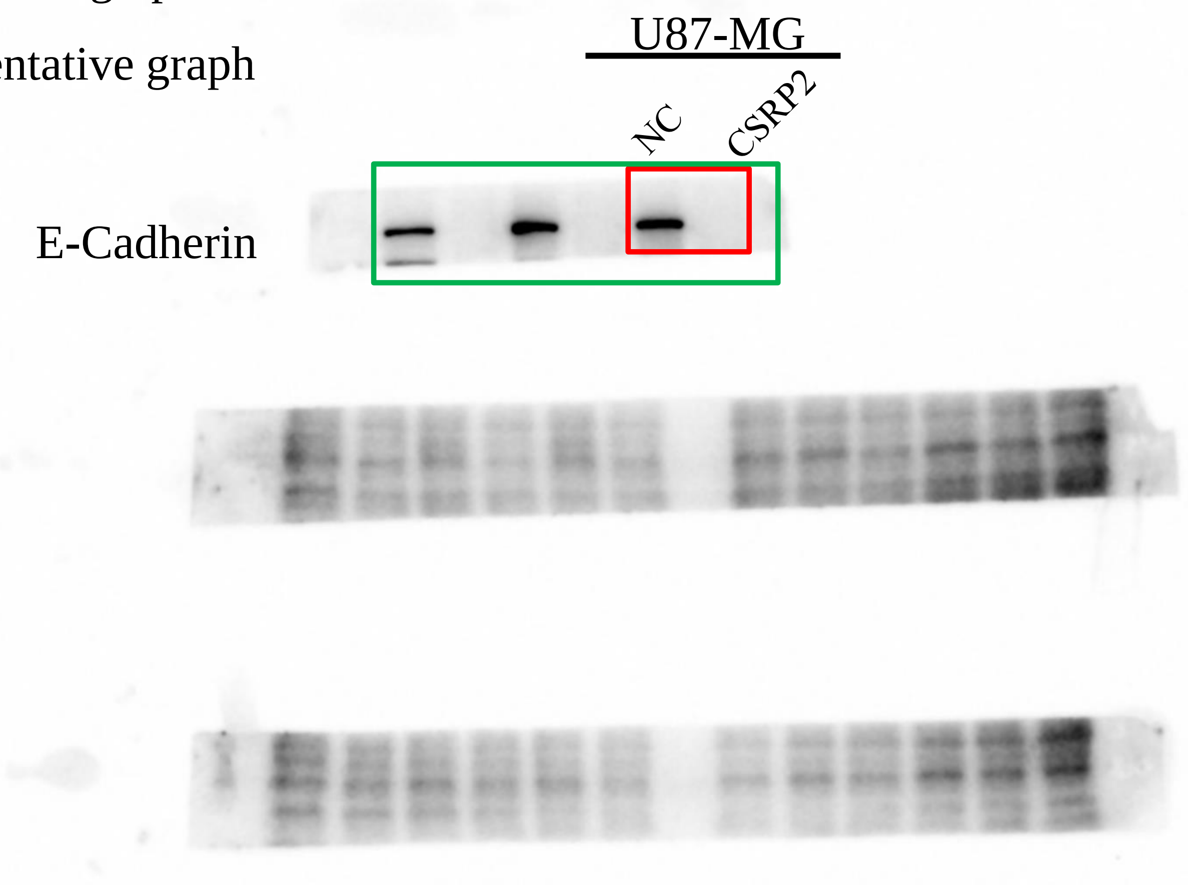

The membrane was imaged with Azure Biosystems 300

Full unedited gel for Figure 4C

Green： Statistical graph

Red： Representative graph

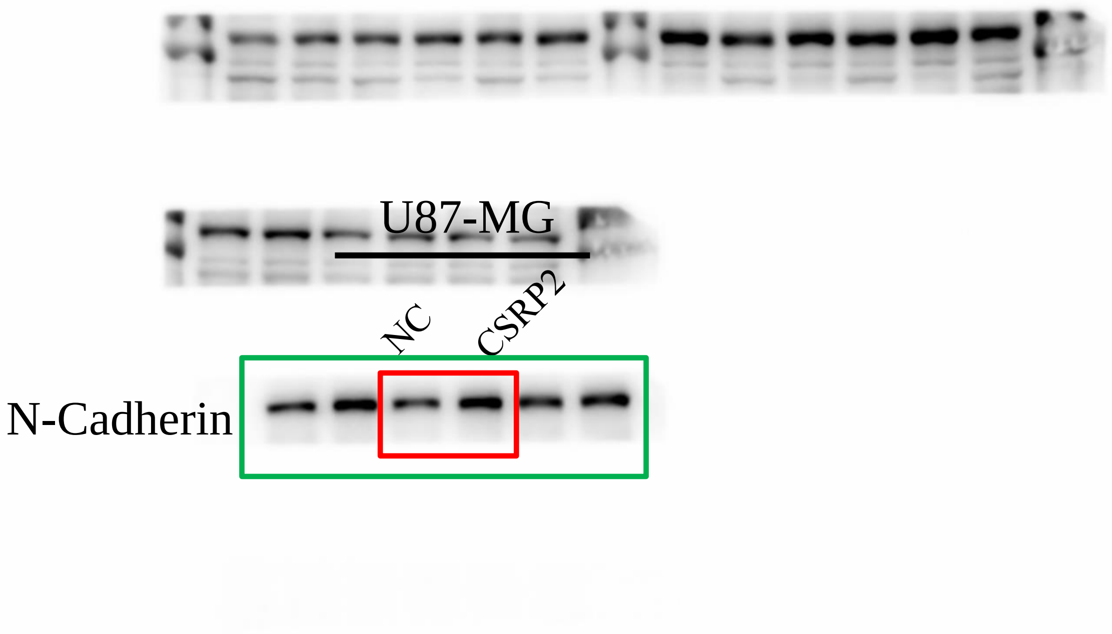

The membrane was imaged with Azure Biosystems 300

Full unedited gel for Figure 4C

Green： Statistical graph

Red： Representative graph

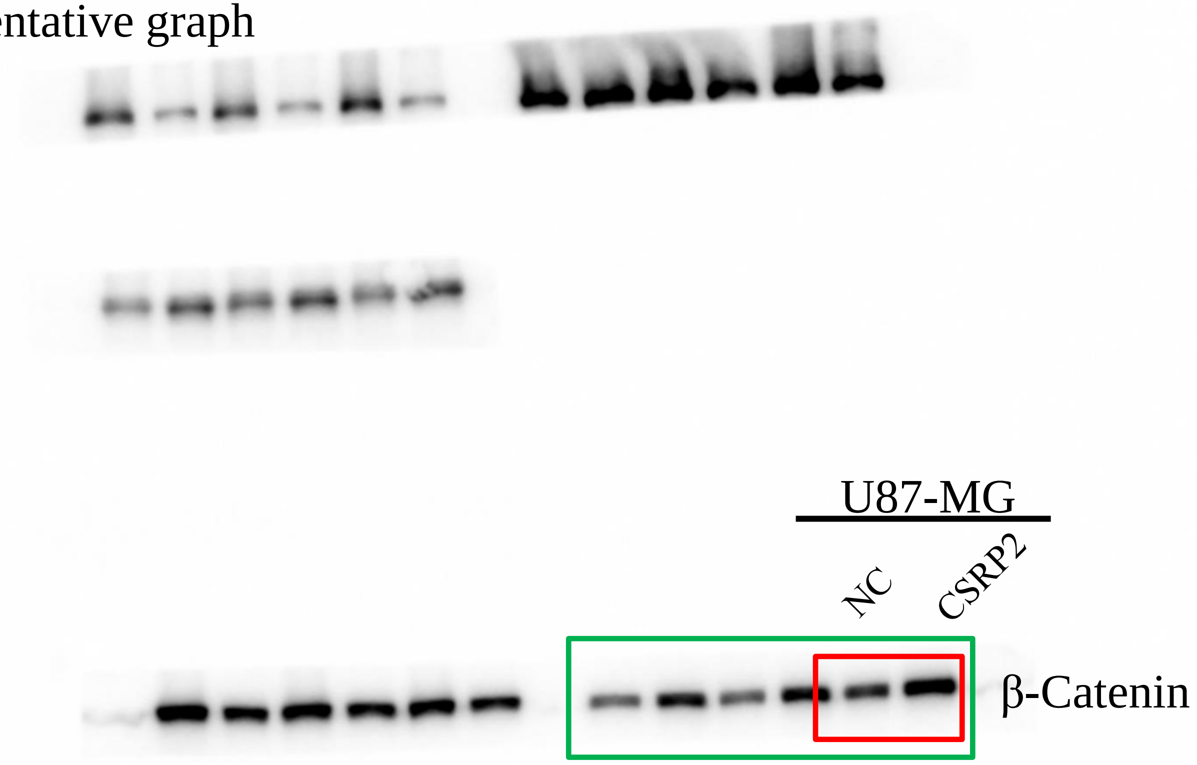

The membrane was imaged with Azure Biosystems 300

Full unedited gel for Figure 4C

Green： Statistical graph

Red： Representative graph

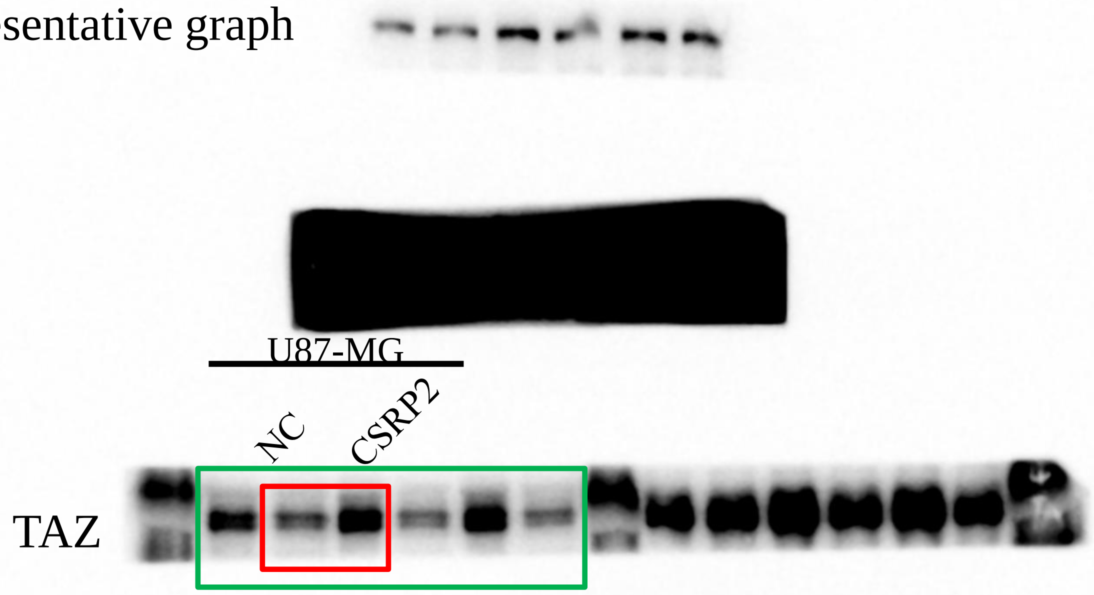

The membrane was imaged with Azure Biosystems 300

Full unedited gel for Figure 4C

Green： Statistical graph

Red： Representative graph

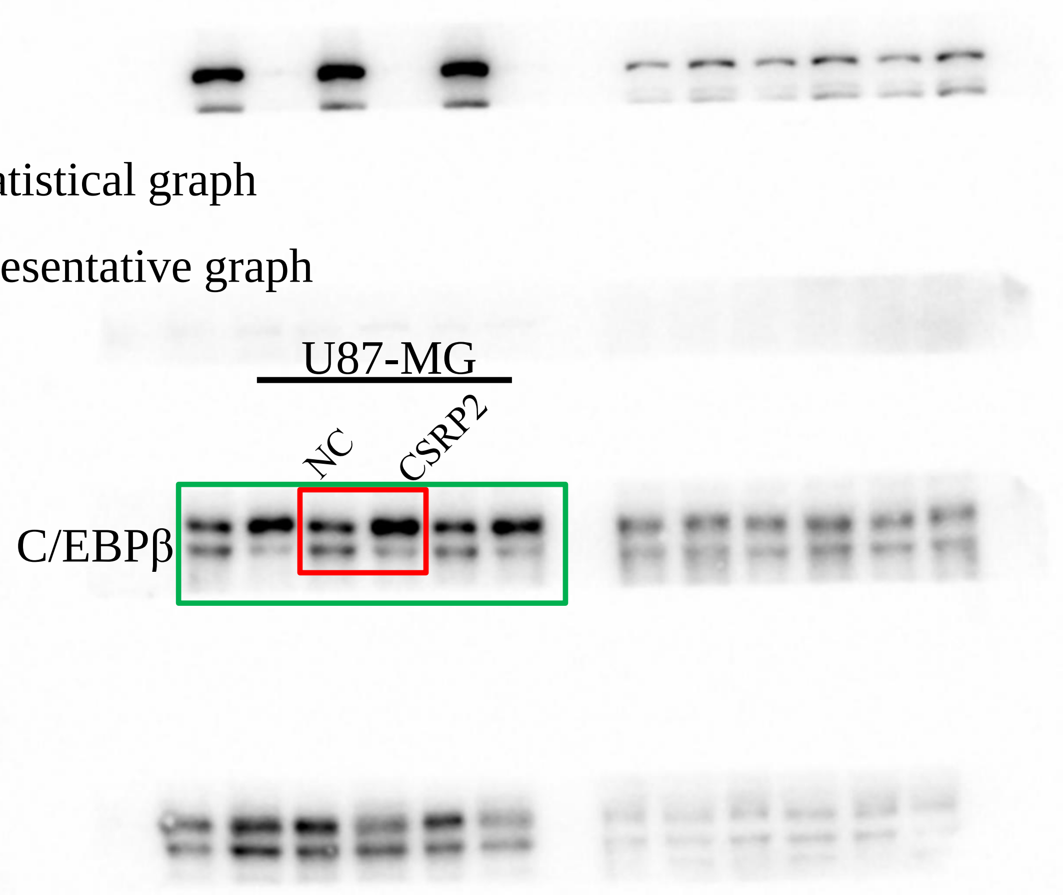

The membrane was imaged with Azure Biosystems 300

Full unedited gel for Figure 4C

Green： Statistical graph

Red： Representative graph

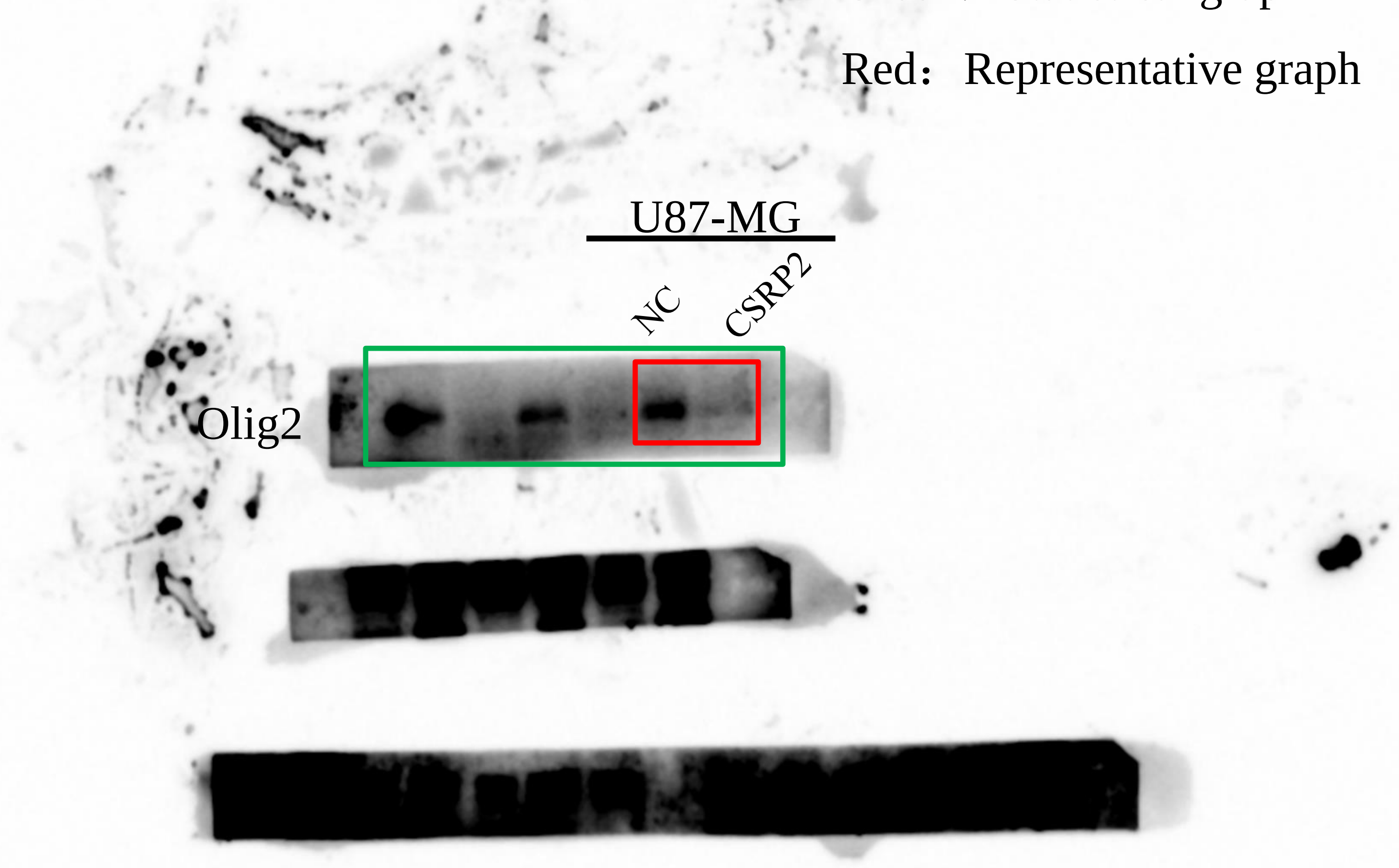

The membrane was imaged with Azure Biosystems 300

Full unedited gel for Figure 4C

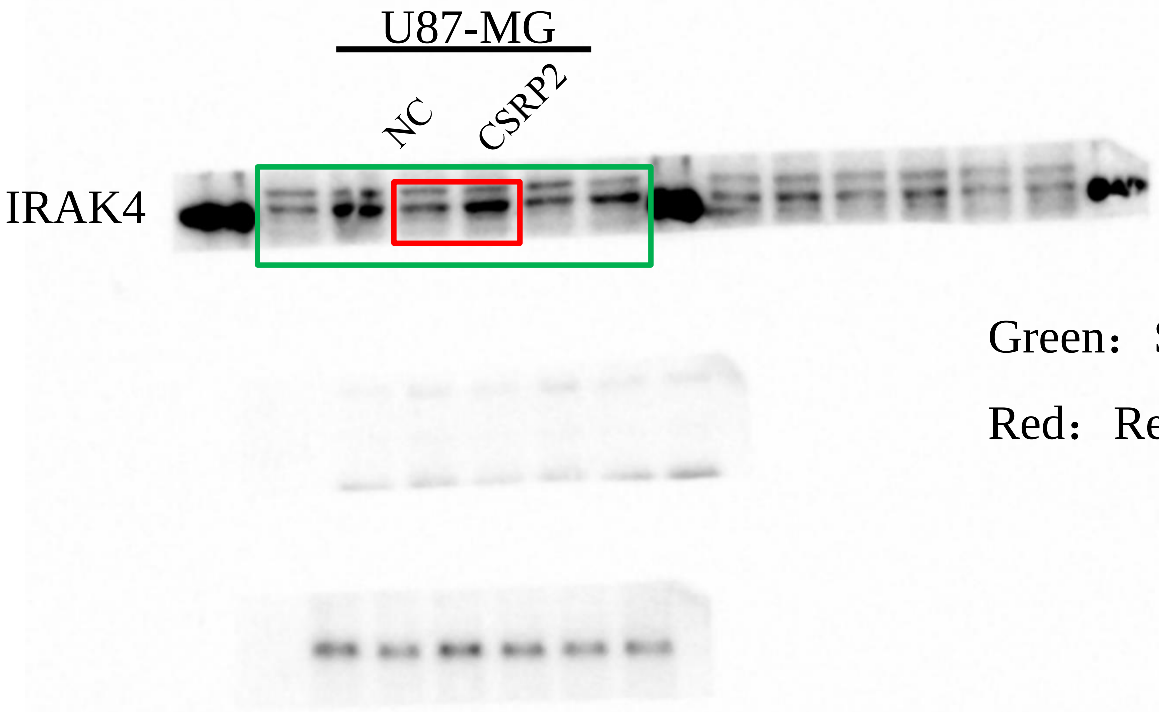

Green： Statistical graph

Red： Representative graph

The membrane was imaged with Azure Biosystems 300

Full unedited gel for Figure 4C

Green： Statistical graph

Red： Representative graph

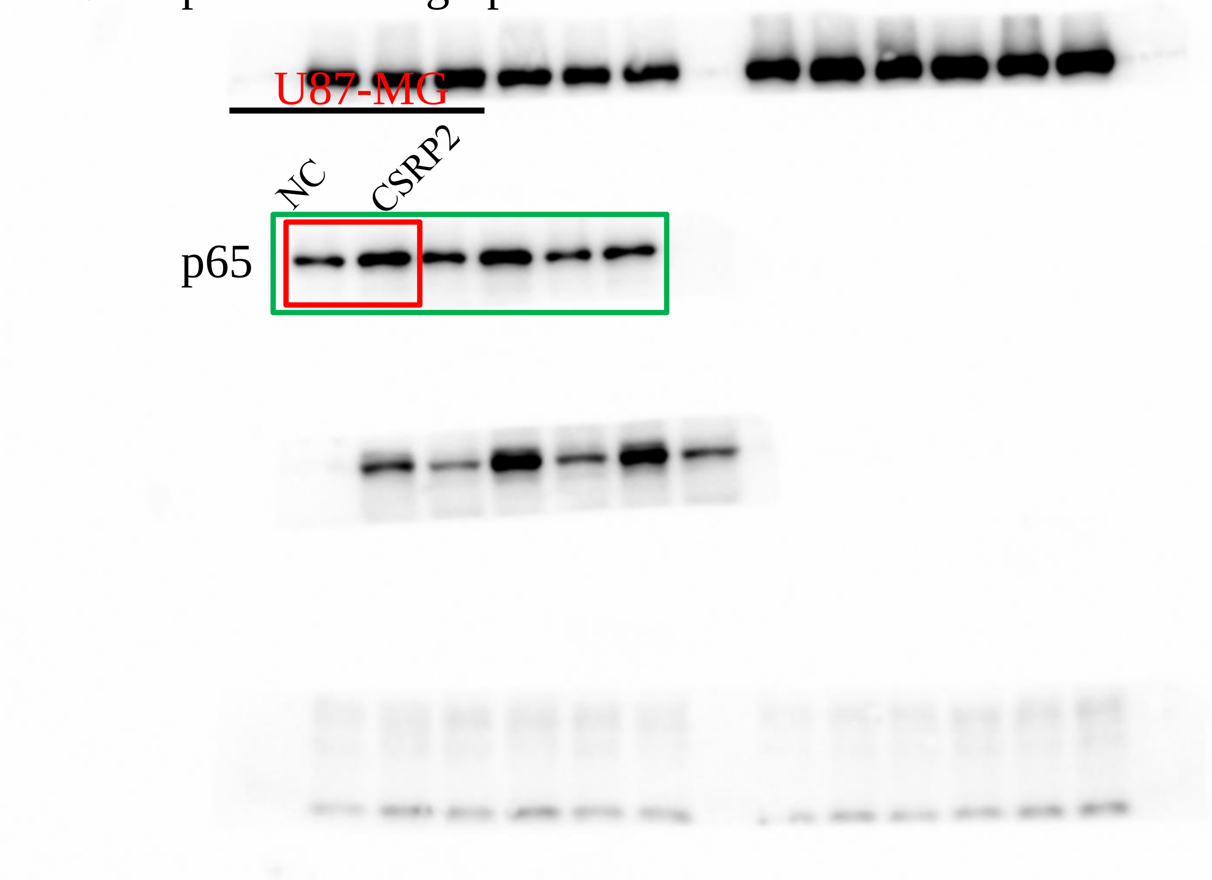

The membrane was imaged with Azure Biosystems 300

Full unedited gel for Figure 4C

Green： Statistical graph

Red： Representative graph

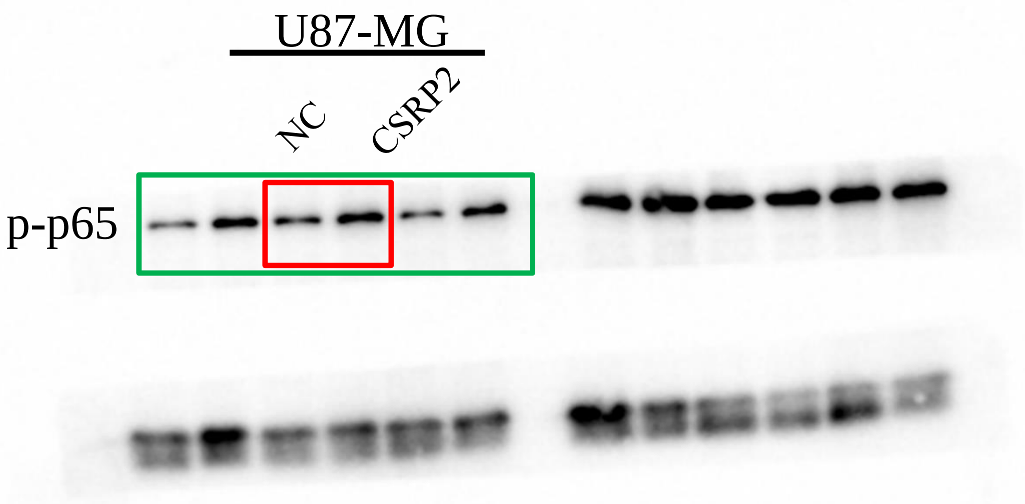

The membrane was imaged with Azure Biosystems 300

Full unedited gel for Figure 4C

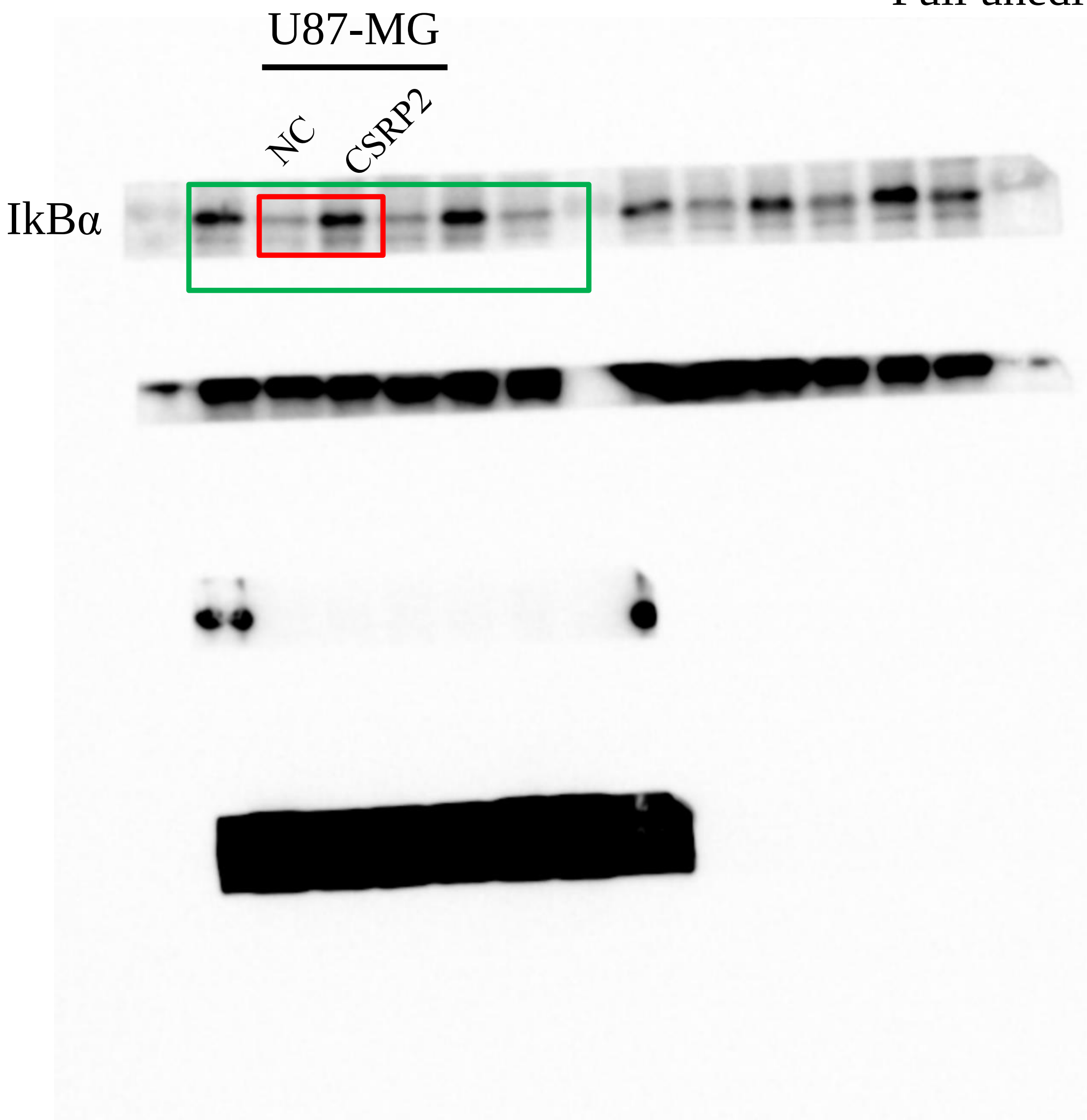

Green： Statistical graph

Red： Representative graph

The membrane was imaged with Azure Biosystems 300

Full unedited gel for Figure 4C

Green: Statistical graph

Red: Representative graph

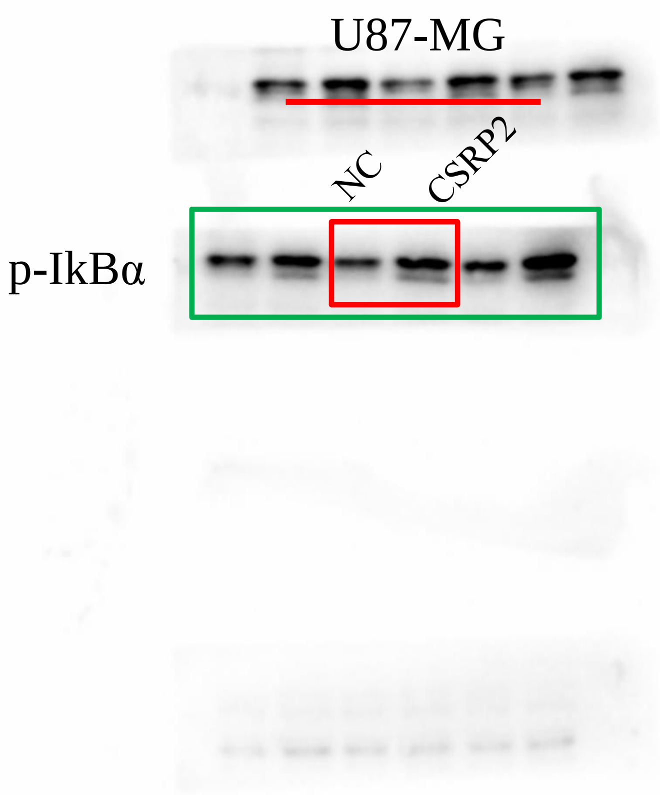

The membrane was imaged with Azure Biosystems 300

Full unedited gel for Figure 4C

Green：Statistical graph

Red：Representative graph

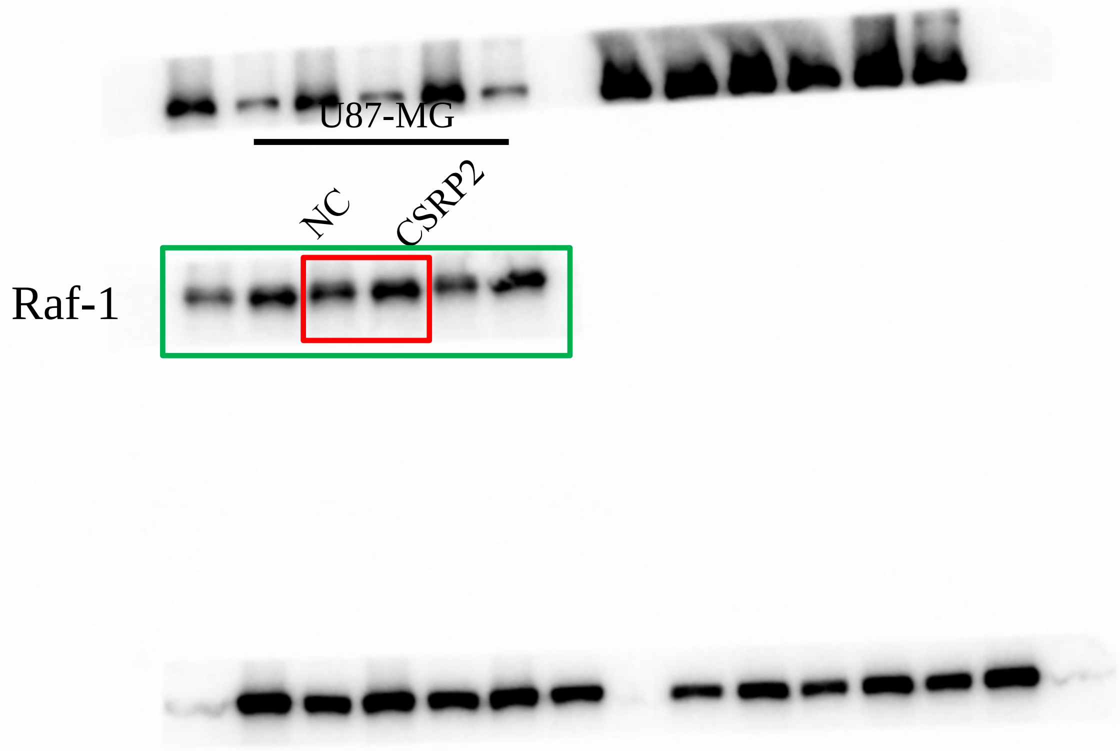

The membrane was imaged with Azure Biosystems 300

Full unedited gel for Figure 4C

Green： Statistical graph

Red： Representative graph

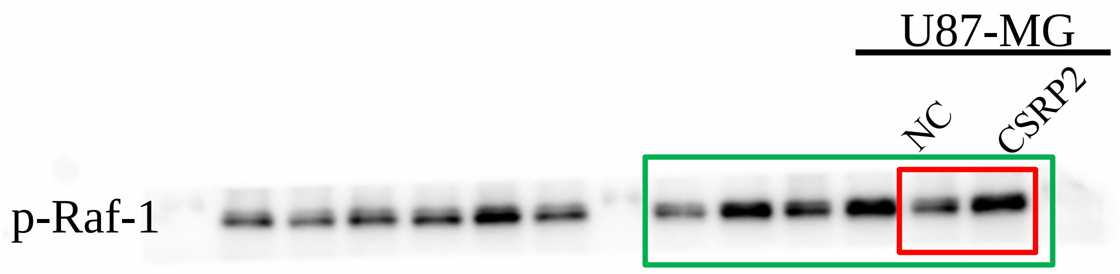

The membrane was imaged with Azure Biosystems 300

Full unedited gel for Figure 4C

Green： Statistical graph

Red： Representative graph

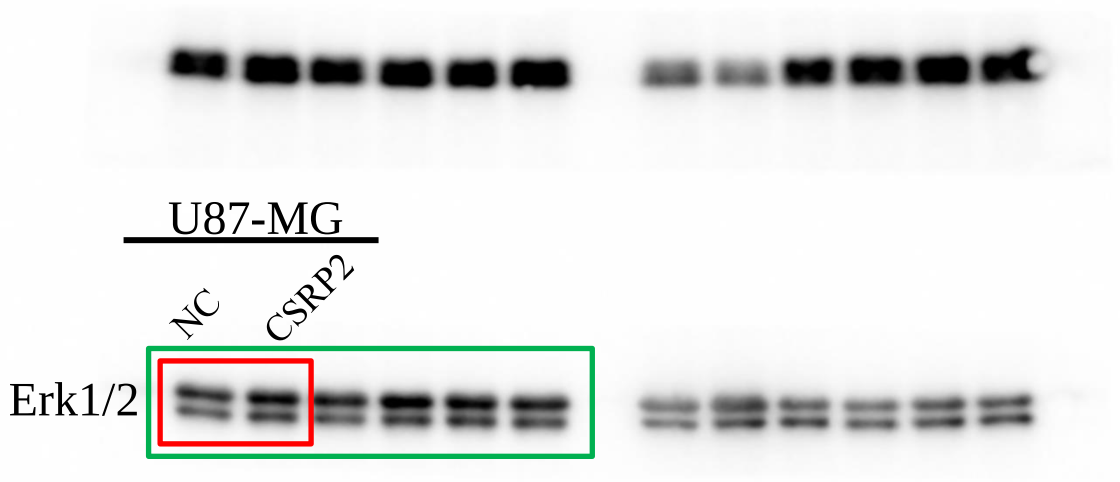

The membrane was imaged with Azure Biosystems 300

Full unedited gel for Figure 4C

Green： Statistical graph

Red： Representative graph

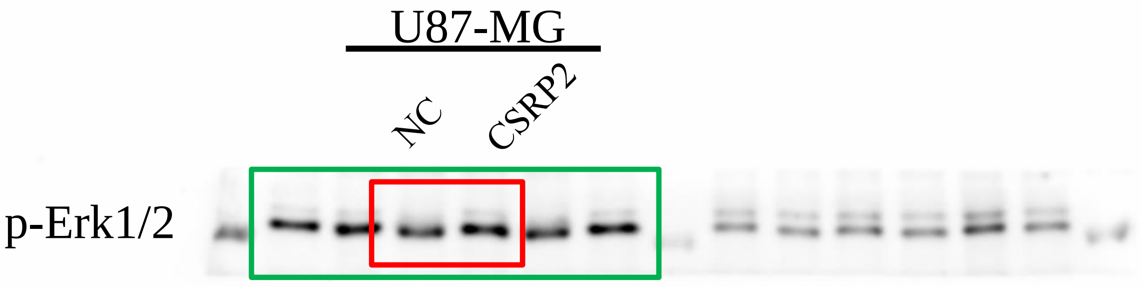

The membrane was imaged with Azure Biosystems 300

Full unedited gel for Figure 4C

Green： Statistical graph

Red： Representative graph

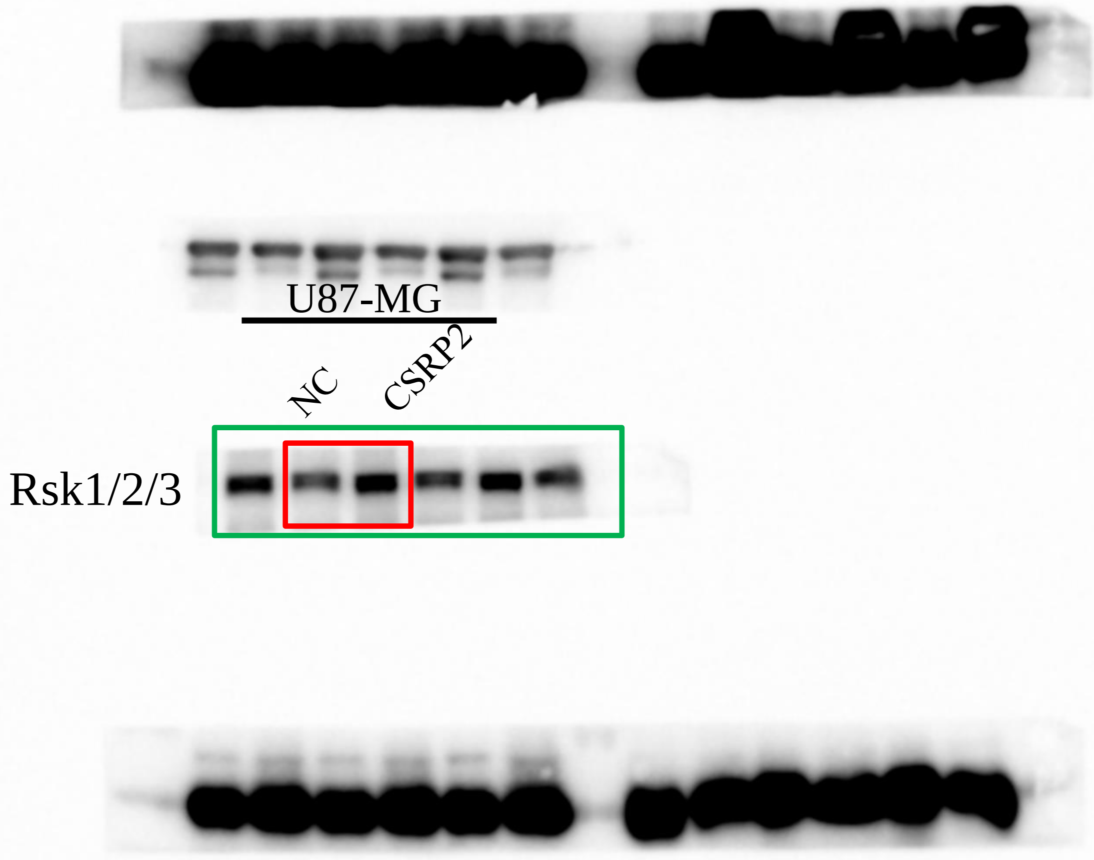

The membrane was imaged with Azure Biosystems 300

Full unedited gel for Figure 4C

Green： Statistical graph

Red： Representative graph

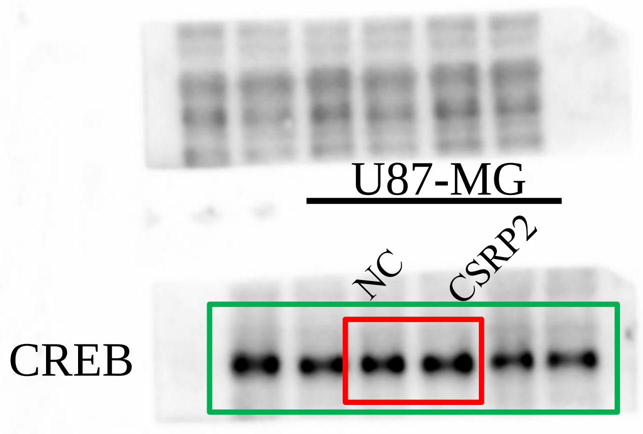

The membrane was imaged with Azure Biosystems 300

Full unedited gel for Figure 4C

Green: Statistical graph

Red: Representative graph

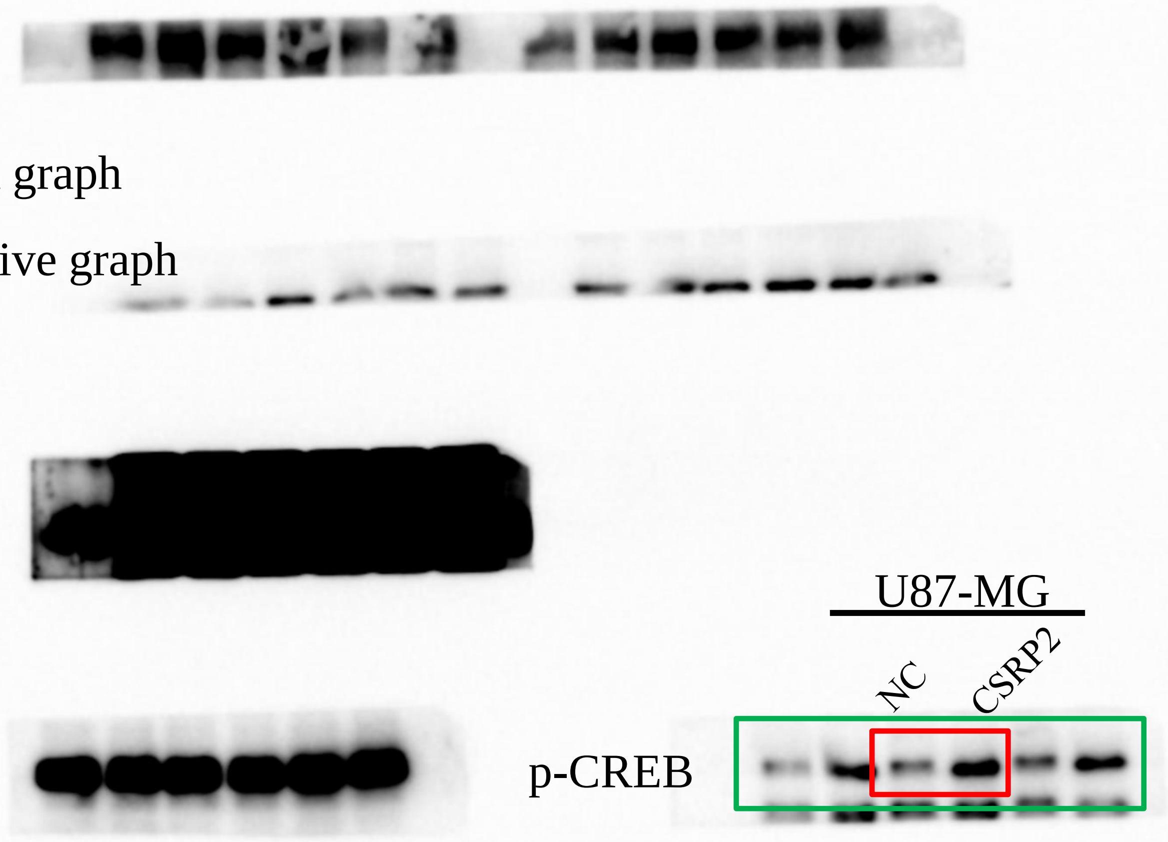

The membrane was imaged with Azure Biosystems 300

Full unedited gel for Figure 4C

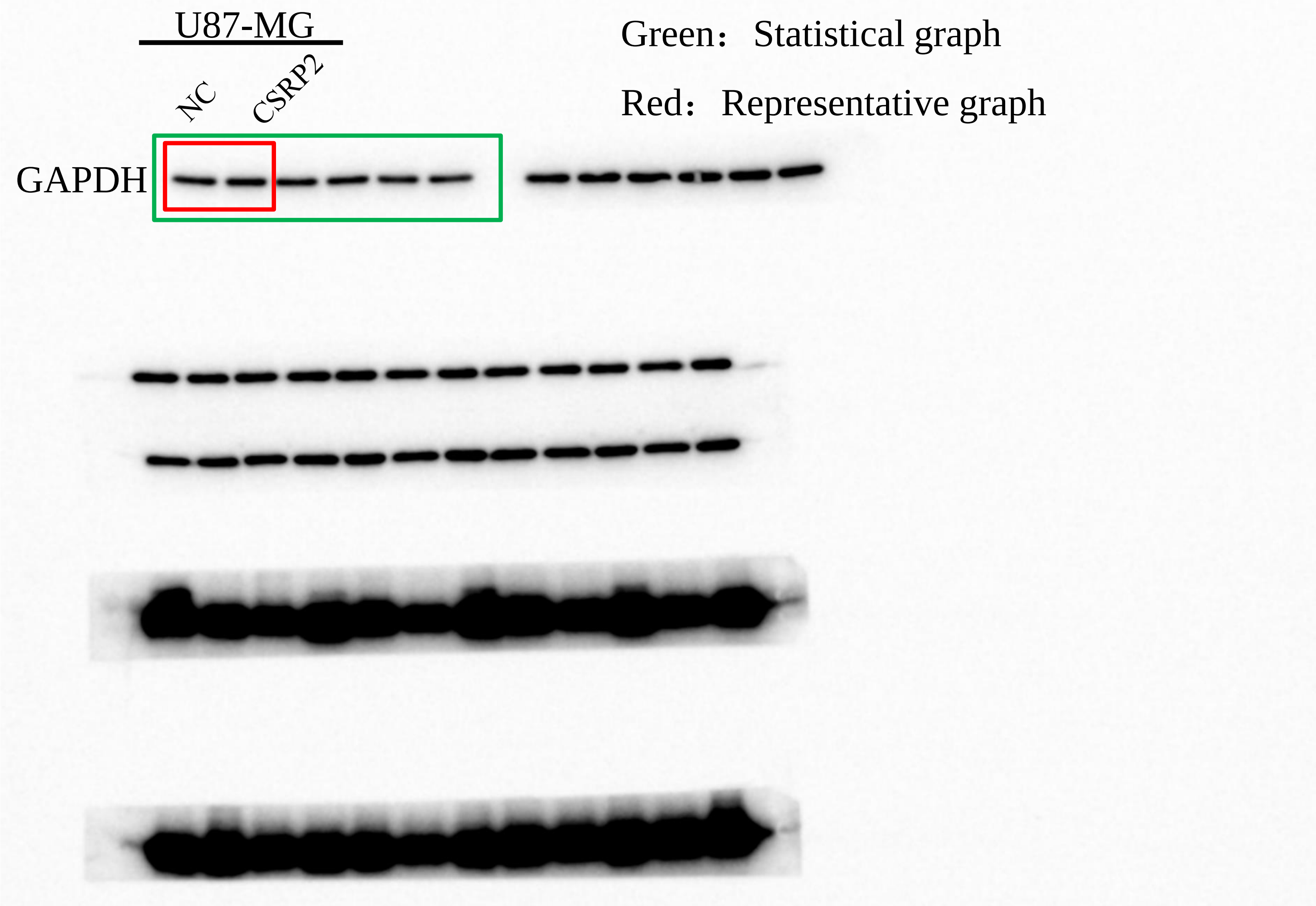

The membrane was imaged with Azure Biosystems 300

Full unedited gel for Figure 4C

Green: Statistical graph

Red: Representative graph

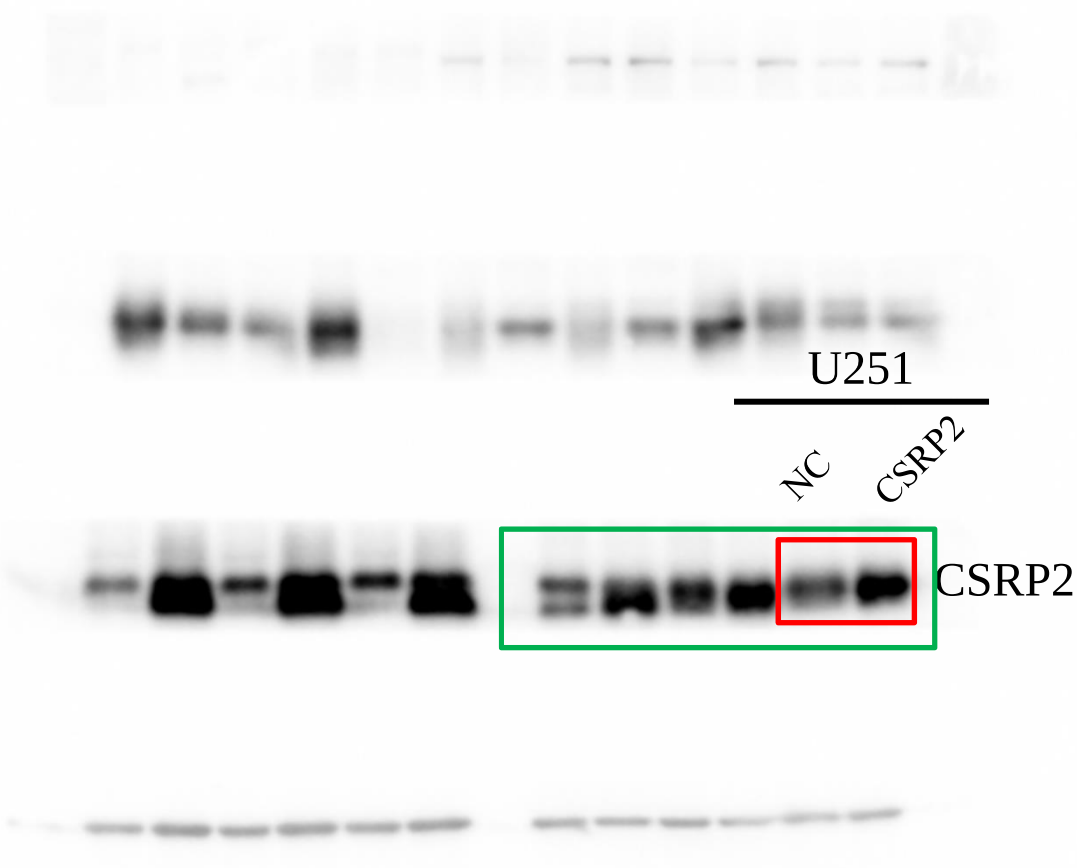

The membrane was imaged with Azure Biosystems 300

Full unedited gel for Figure 4C

Green： Statistical graph

Red： Representative graph

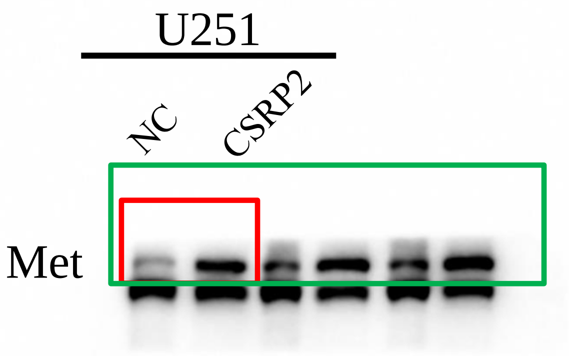

The membrane was imaged with Azure Biosystems 300

Full unedited gel for Figure 4C

Green： Statistical graph

Red： Representative graph

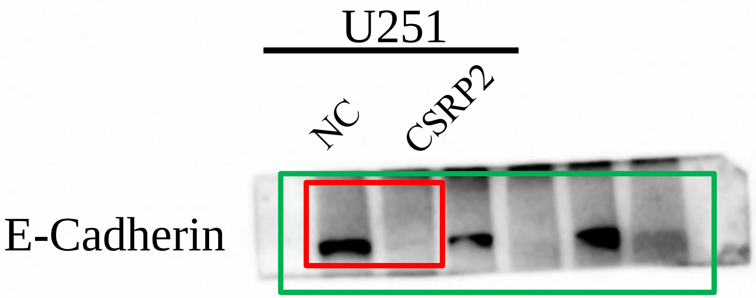

The membrane was imaged with Azure Biosystems 300

Full unedited gel for Figure 4C

Green： Statistical graph

Red： Representative graph

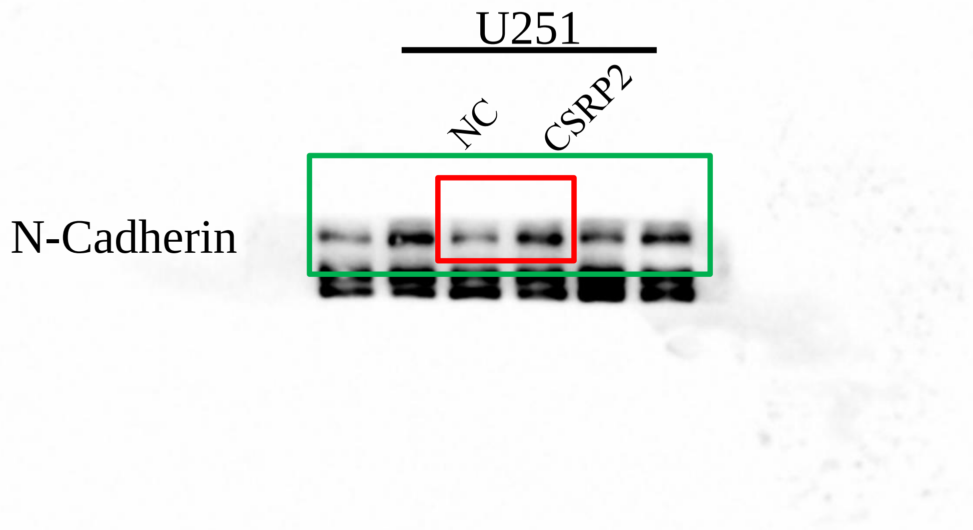

The membrane was imaged with Azure Biosystems 300

Full unedited gel for Figure 4C

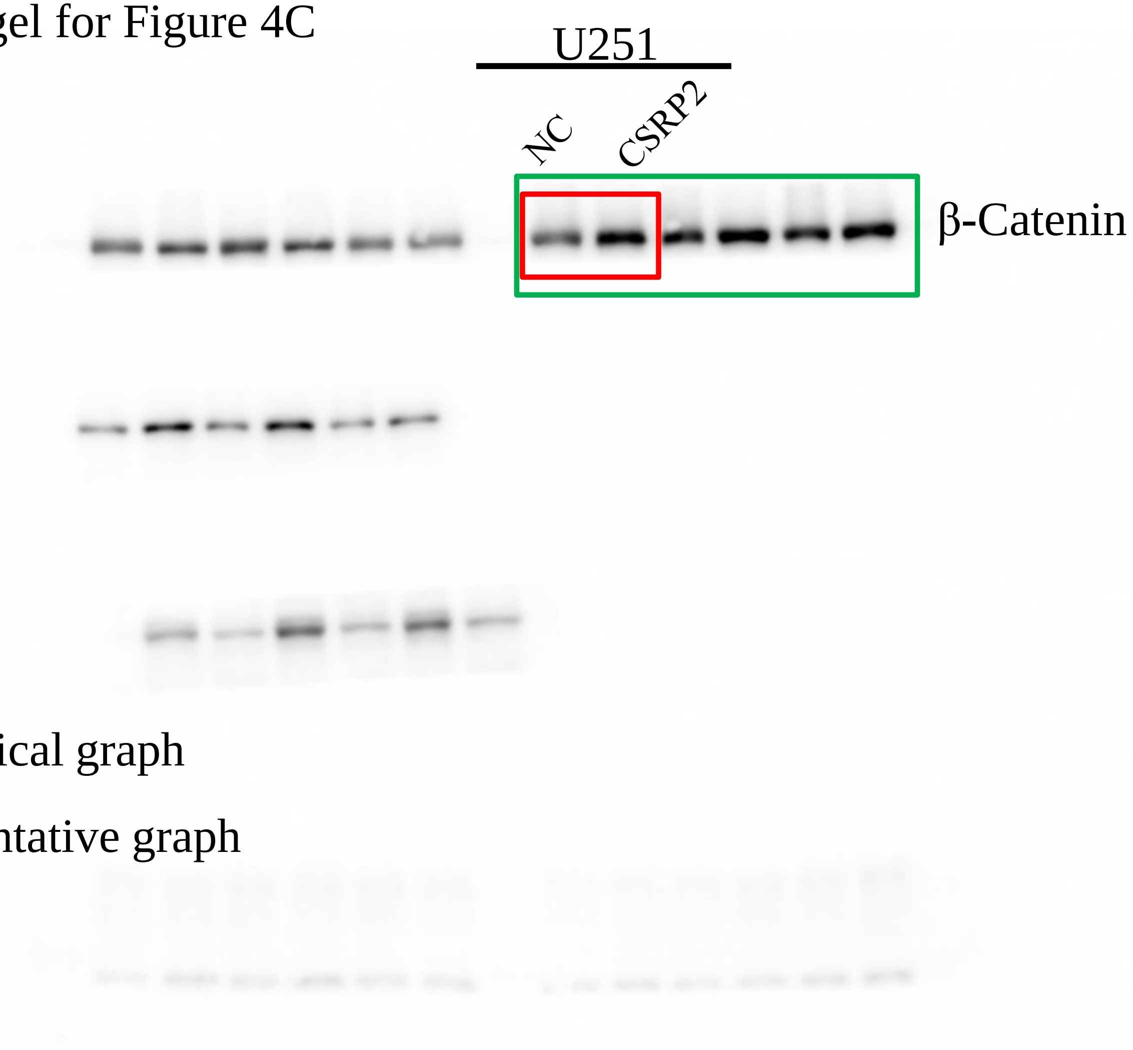

Green: Statistical graph

Red: Representative graph

The membrane was imaged with Azure Biosystems 300

Full unedited gel for Figure 4C

Green： Statistical graph

Red： Representative graph

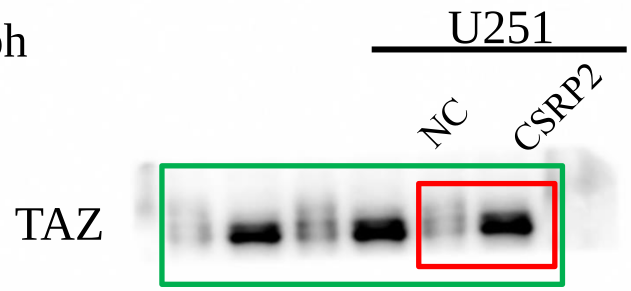

The membrane was imaged with Azure Biosystems 300

Full unedited gel for Figure 4C

Green： Statistical graph

Red： Representative graph

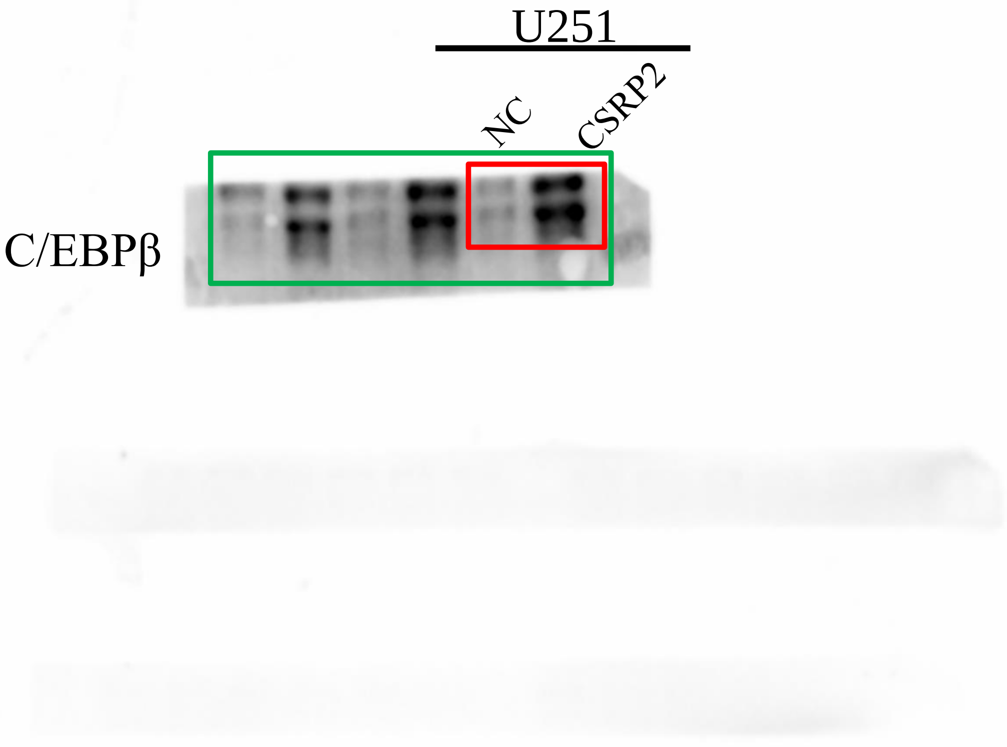

The membrane was imaged with Azure Biosystems 300

Full unedited gel for Figure 4C

Green： Statistical graph

Red： Representative graph

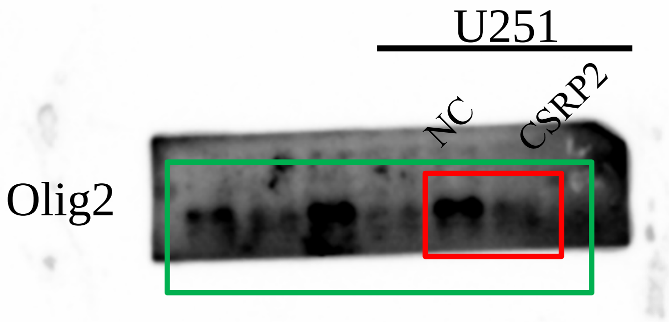

The membrane was imaged with Azure Biosystems 300

Full unedited gel for Figure 4C

Green： Statistical graph

Red： Representative graph

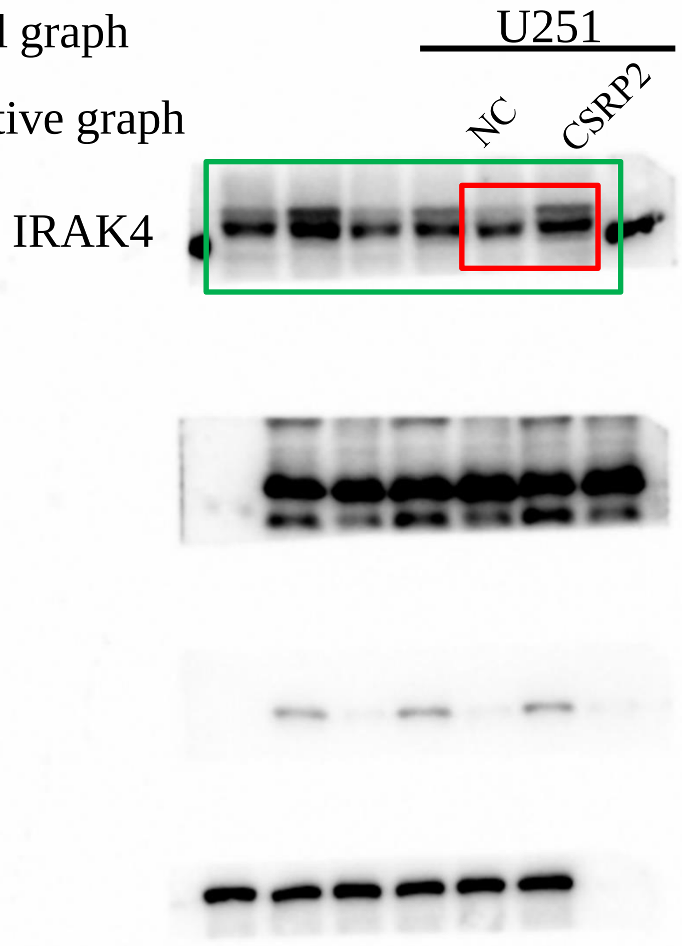

The membrane was imaged with Azure Biosystems 300

Full unedited gel for Figure 4C

Green： Statistical graph

Red： Representative graph

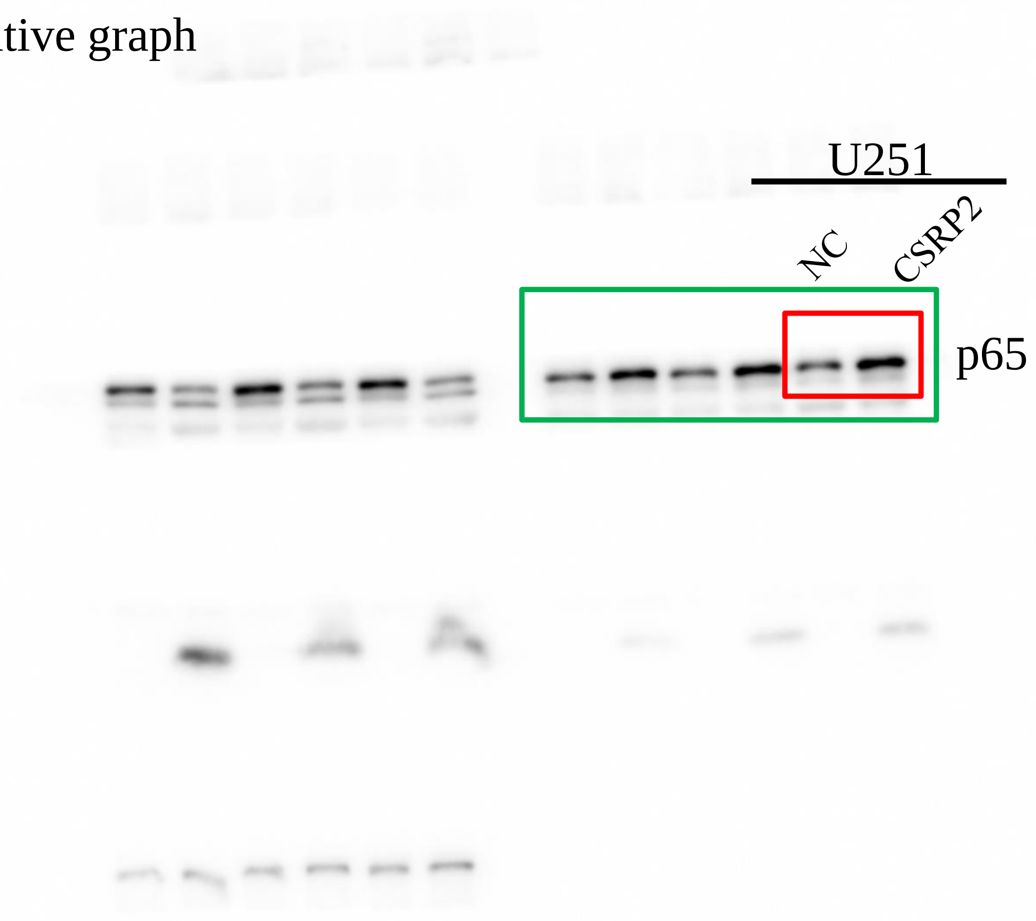

The membrane was imaged with Azure Biosystems 300

Full unedited gel for Figure 4C

Green： Statistical graph

Red： Representative graph

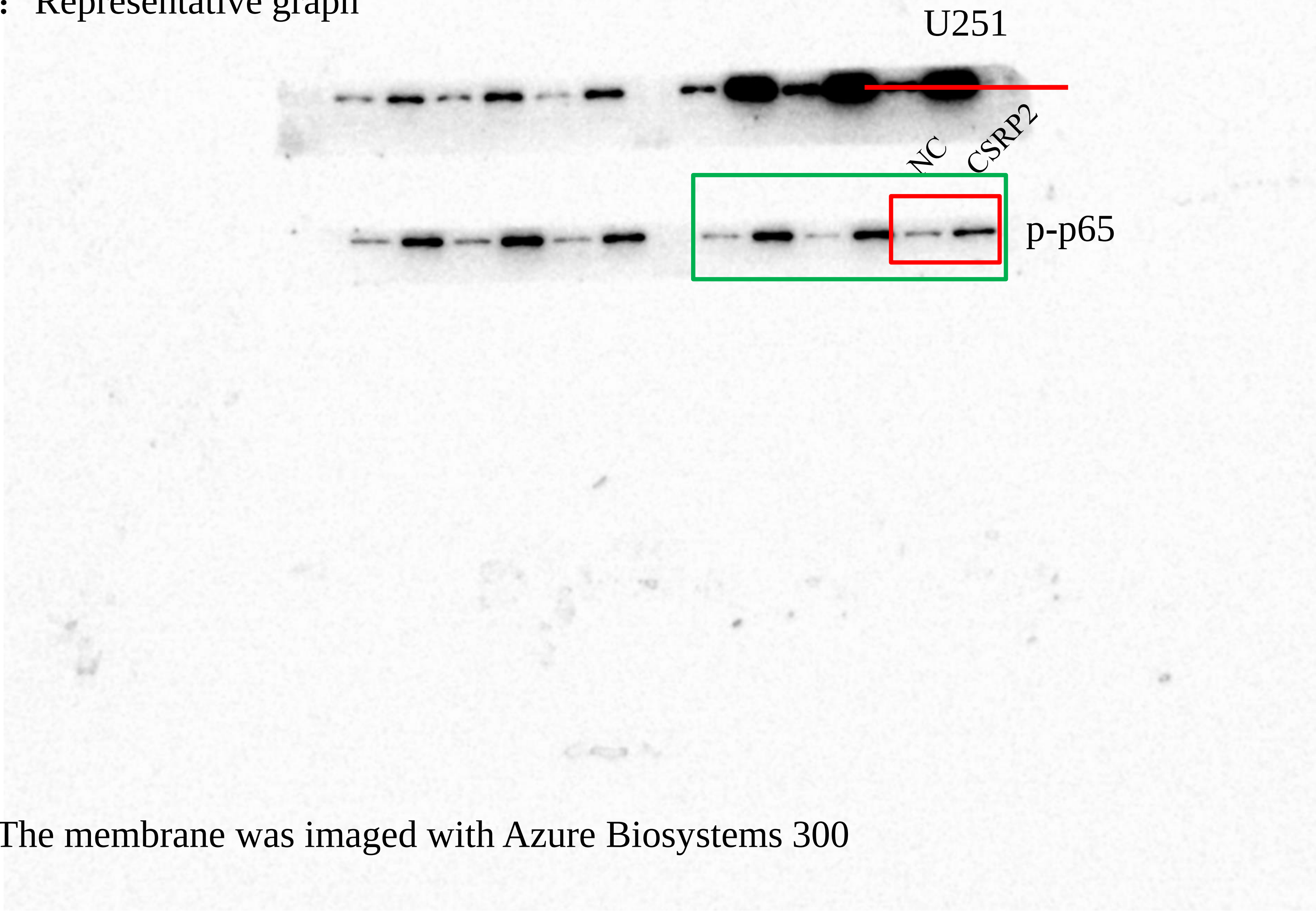

The membrane was imaged with Azure Biosystems 300

Full unedited gel for Figure 4C

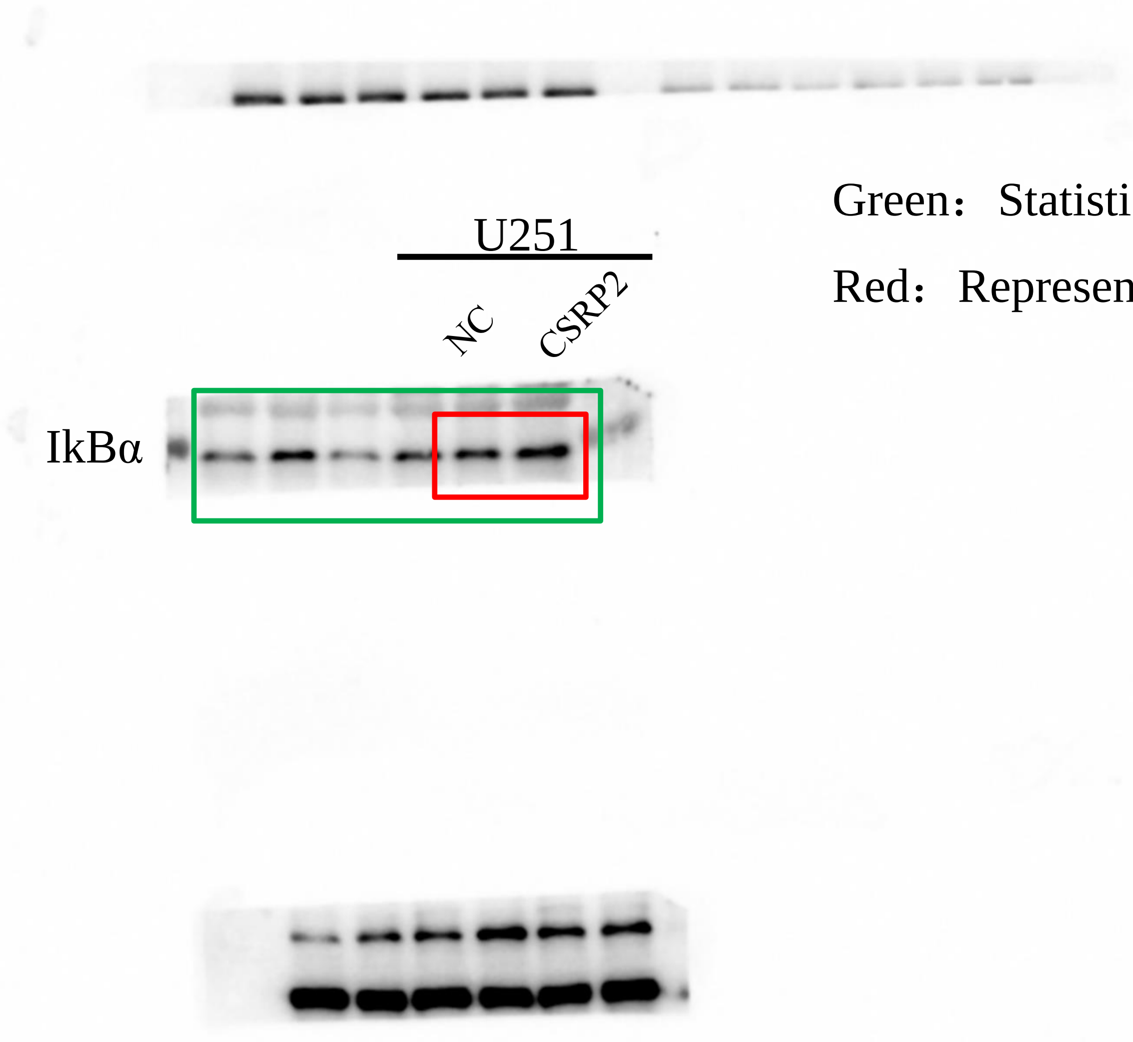

Green: Statistical graph

Red: Representative graph

The membrane was imaged with Azure Biosystems 300

Full unedited gel for Figure 4C

Green： Statistical graph

Red： Representative graph

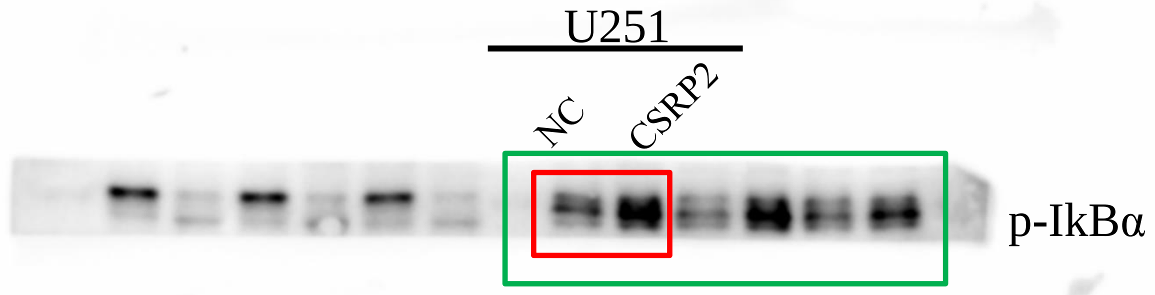

The membrane was imaged with Azure Biosystems 300

Full unedited gel for Figure 4C

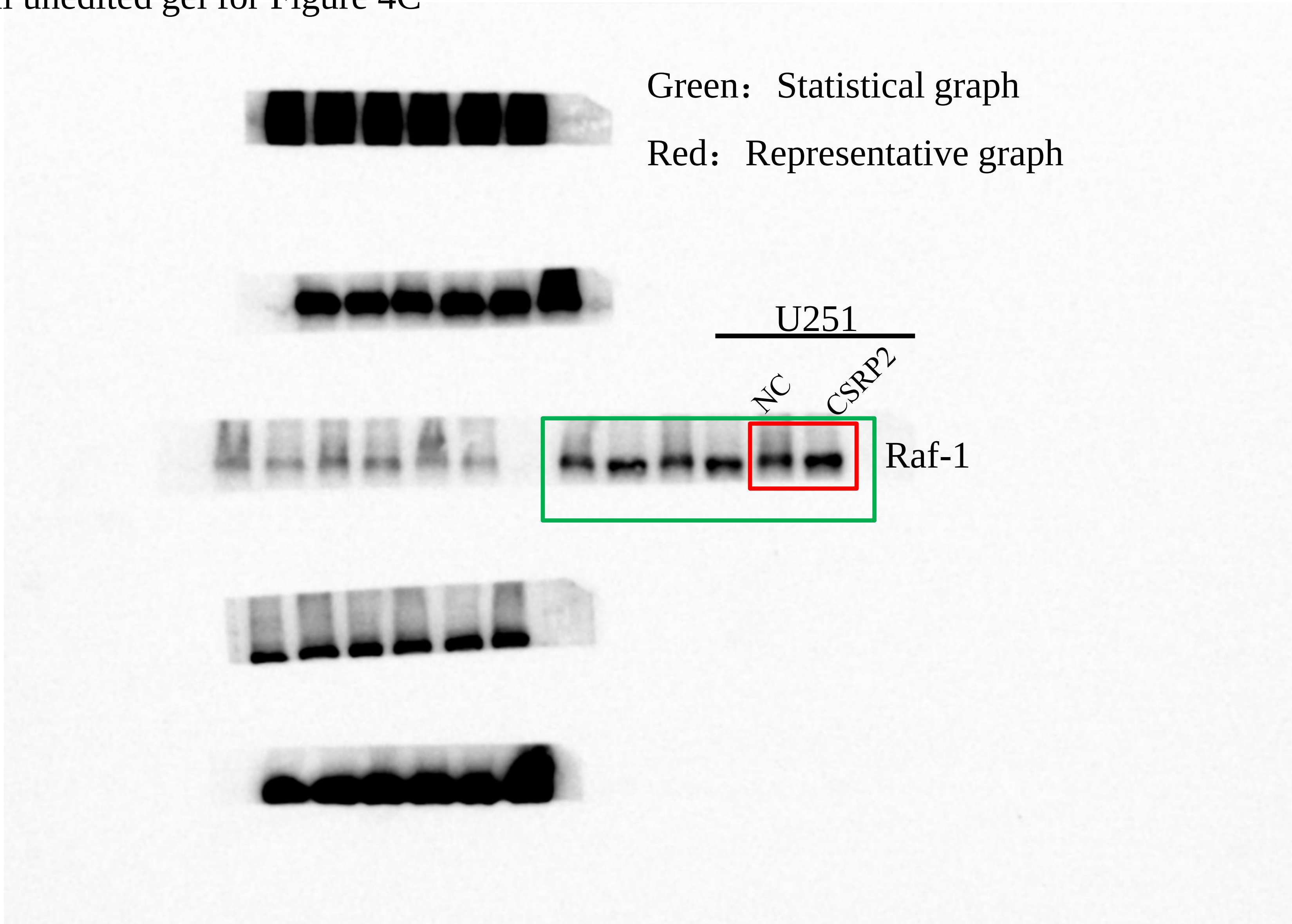

The membrane was imaged with Azure Biosystems 300

Full unedited gel for Figure 4C

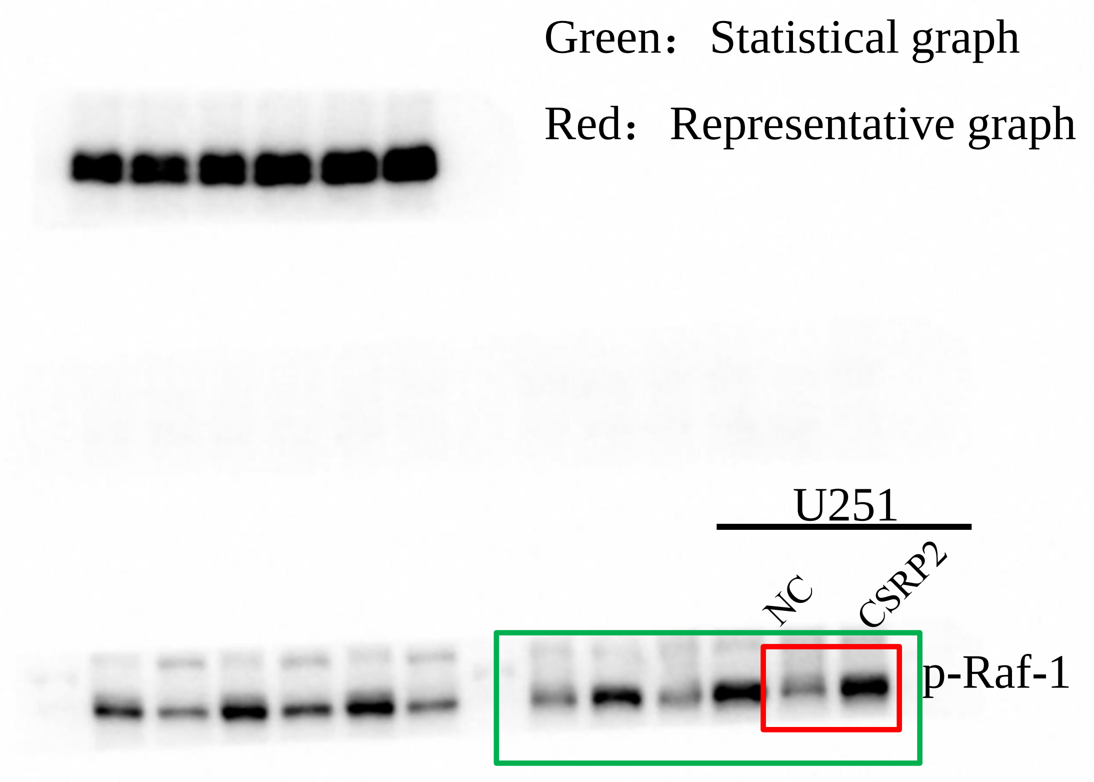

The membrane was imaged with Azure Biosystems 300

Full unedited gel for Figure 4C

Green: Statistical graph

Red: Representative graph

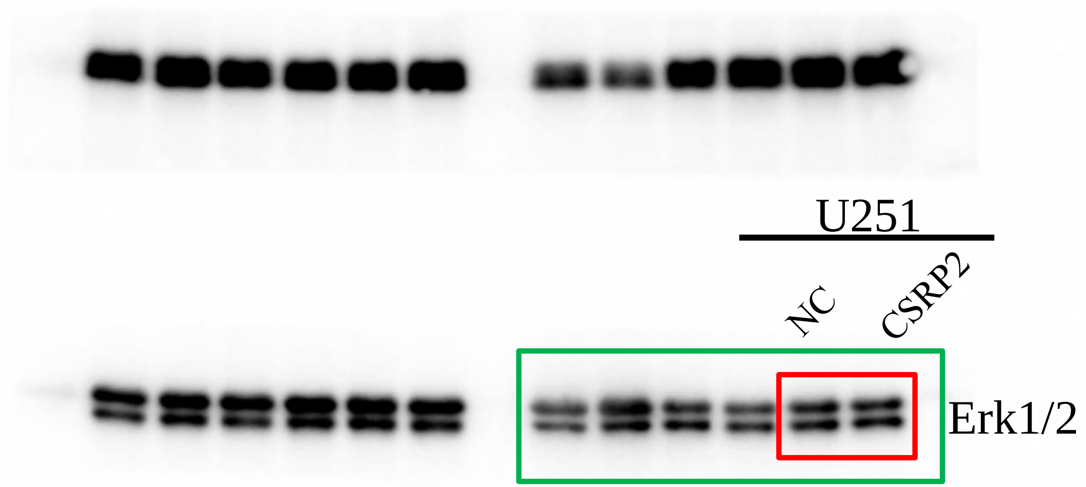

The membrane was imaged with Azure Biosystems 300

Full unedited gel for Figure 4C

Green: Statistical graph

Red: Representative graph

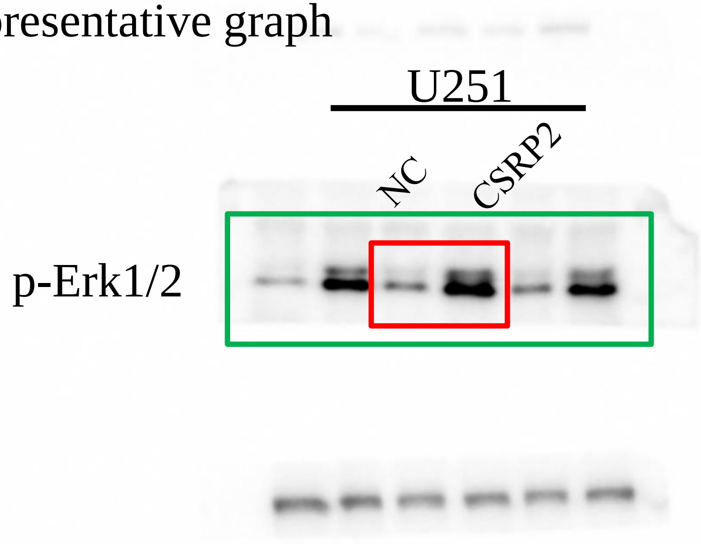

The membrane was imaged with Azure Biosystems 300

Full unedited gel for Figure 4C

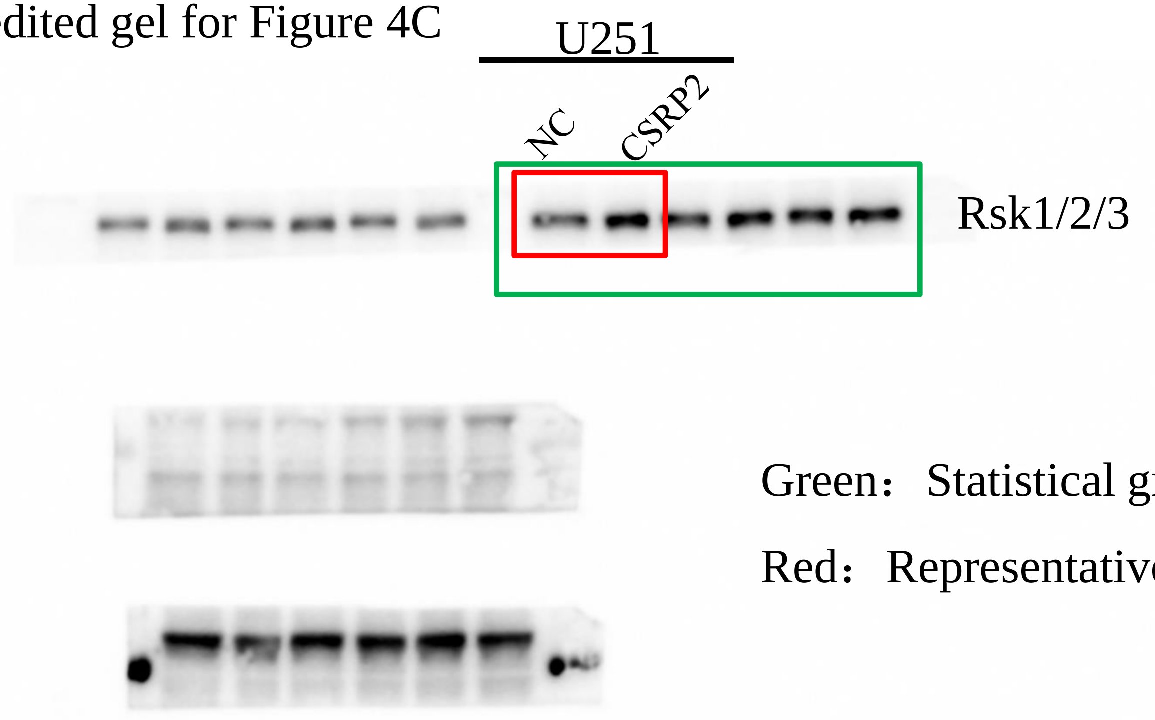

Green: Statistical graph

Red: Representative graph

The membrane was imaged with Azure Biosystems 300

Full unedited gel for Figure 4C

Green： Statistical graph

Red： Representative graph

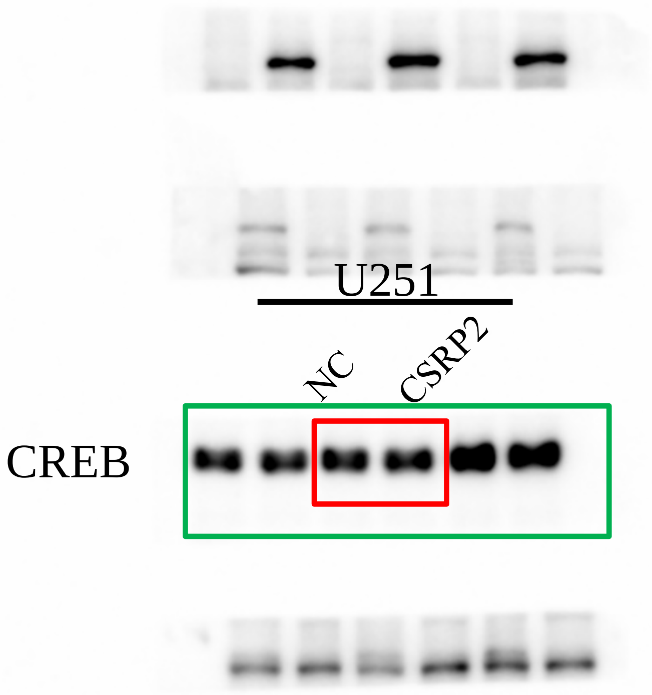

The membrane was imaged with Azure Biosystems 300

Full unedited gel for Figure 4C

Green： Statistical graph

Red： Representative graph

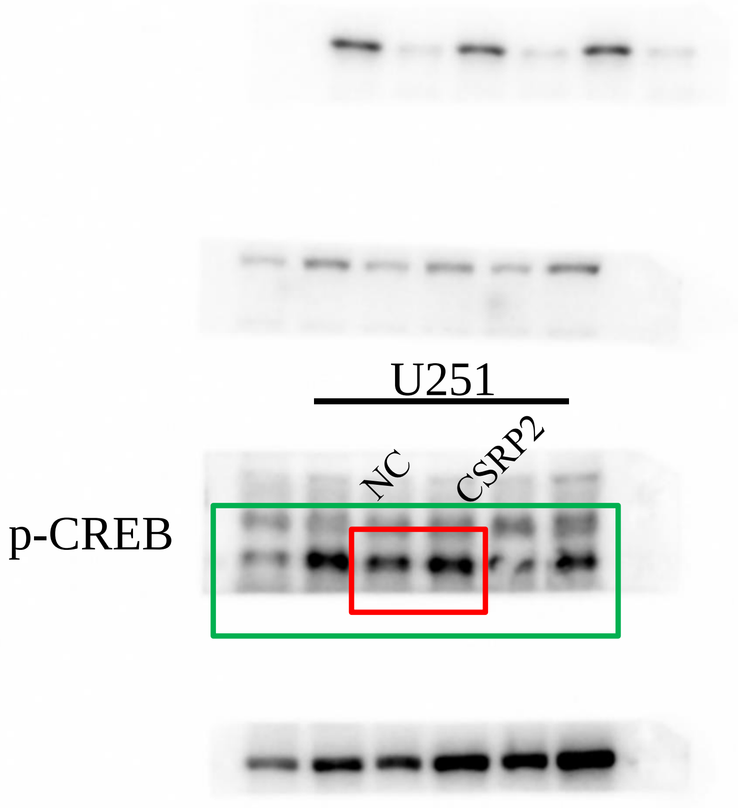

The membrane was imaged with Azure Biosystems 300

Full unedited gel for Figure 4C

Green： Statistical graph

Red： Representative graph

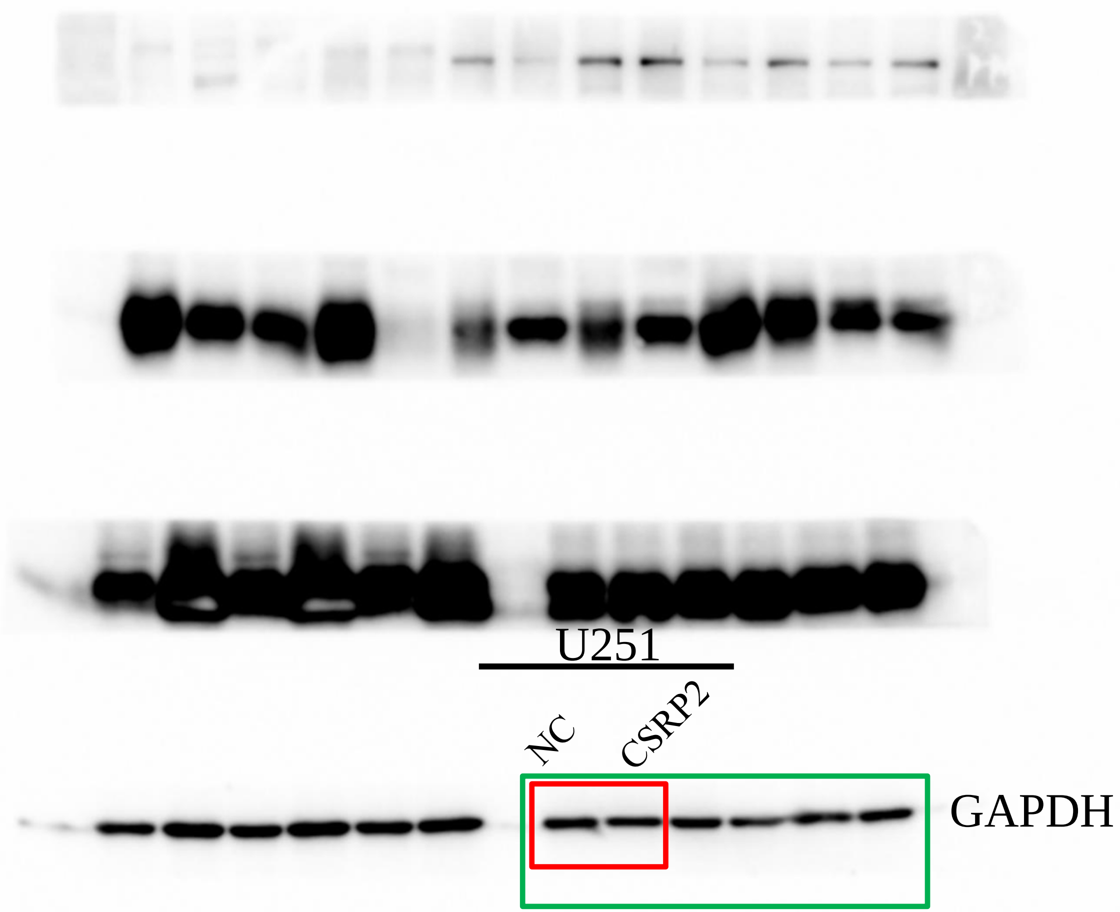

The membrane was imaged with Azure Biosystems 300

Full unedited gel for Figure 4F

Green： Statistical graph

Red： Representative graph

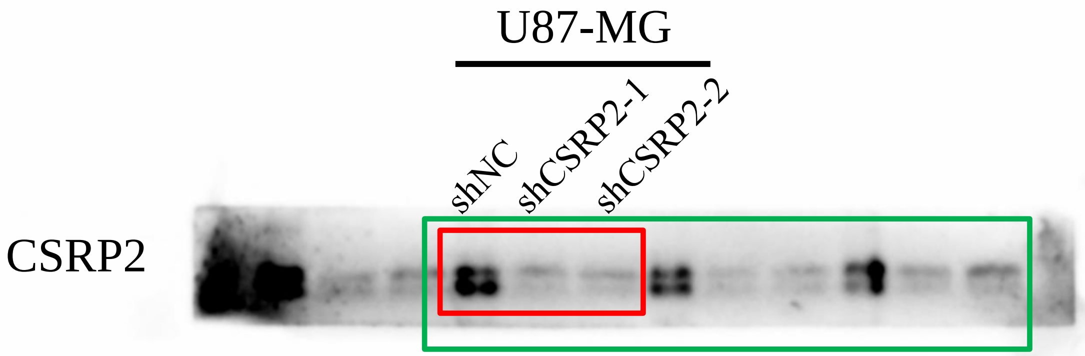

The membrane was imaged with Azure Biosystems 300

Full unedited gel for Figure 4F

Green： Statistical graph

Red： Representative graph

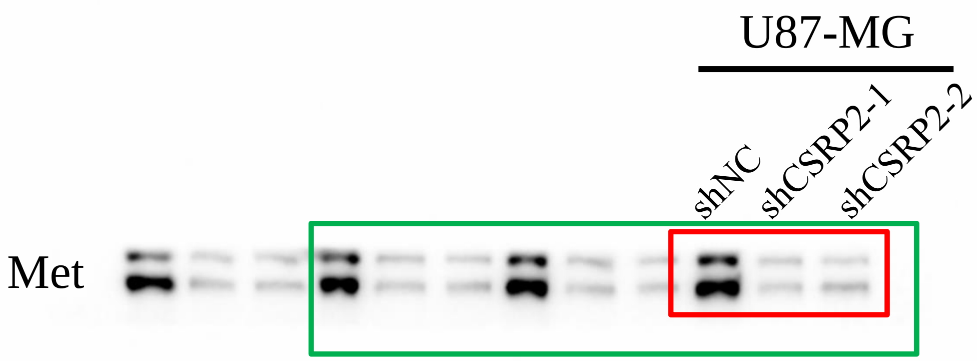

The membrane was imaged with Azure Biosystems 300

Full unedited gel for Figure 4F

Green： Statistical graph

Red： Representative graph

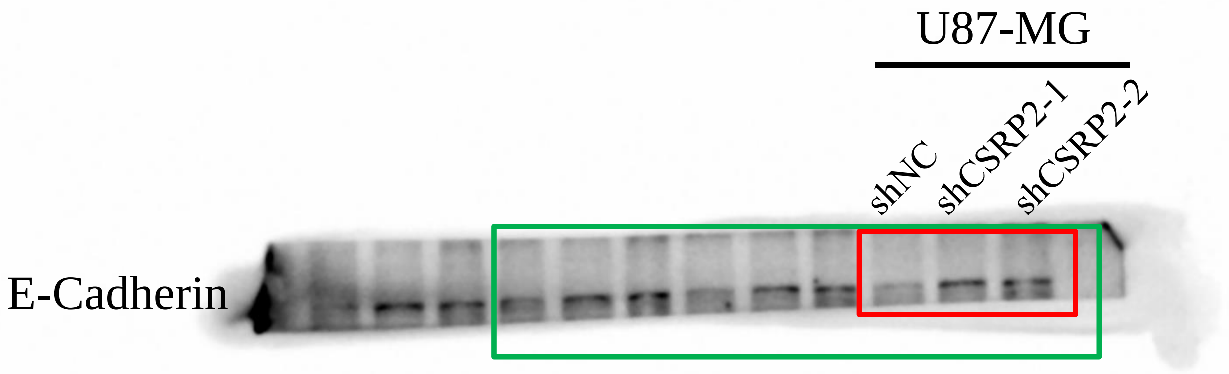

The membrane was imaged with Azure Biosystems 300

Full unedited gel for Figure 4F

Green： Statistical graph

Red： Representative graph

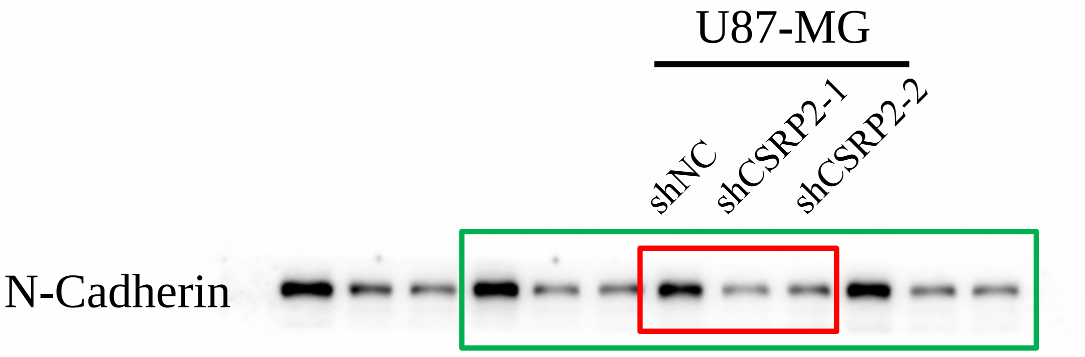

The membrane was imaged with Azure Biosystems 300

Full unedited gel for Figure 4F

Green： Statistical graph

Red： Representative graph

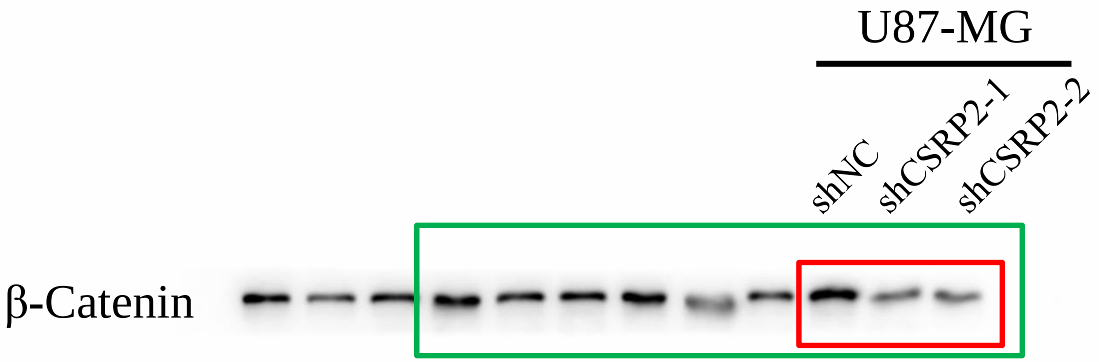

The membrane was imaged with Azure Biosystems 300

Full unedited gel for Figure 4F

Green: Statistical graph

Red: Representative graph

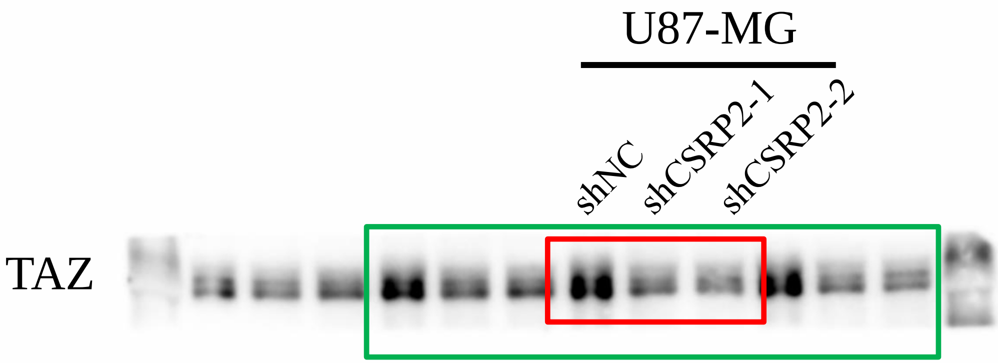

The membrane was imaged with Azure Biosystems 300

Full unedited gel for Figure 4F

Green： Statistical graph

Red： Representative graph

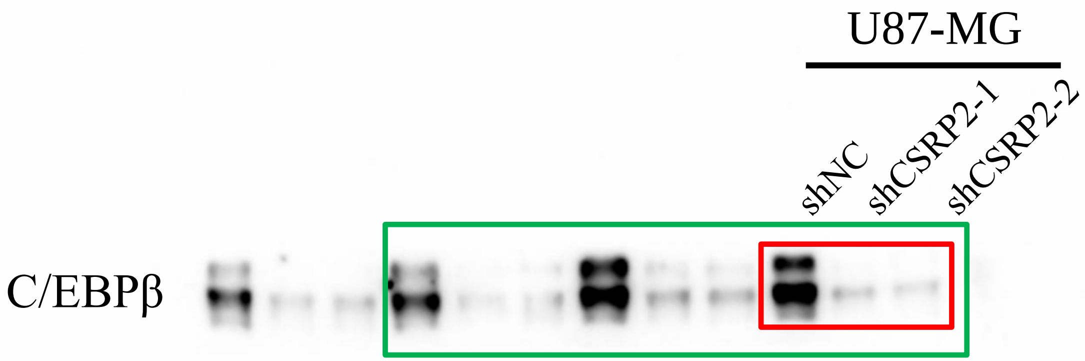

The membrane was imaged with Azure Biosystems 300

Full unedited gel for Figure 4F

Green: Statistical graph

Red: Representative graph

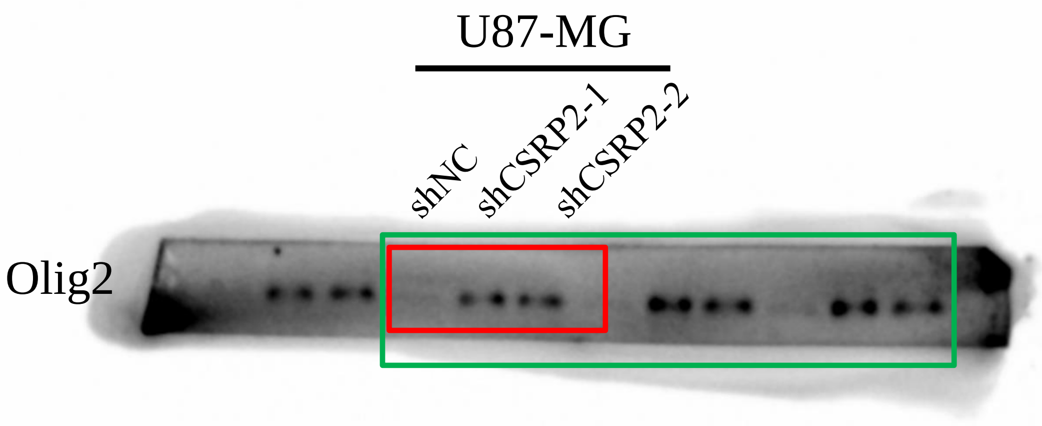

The membrane was imaged with Azure Biosystems 300

Full unedited gel for Figure 4F

Green： Statistical graph

Red： Representative graph

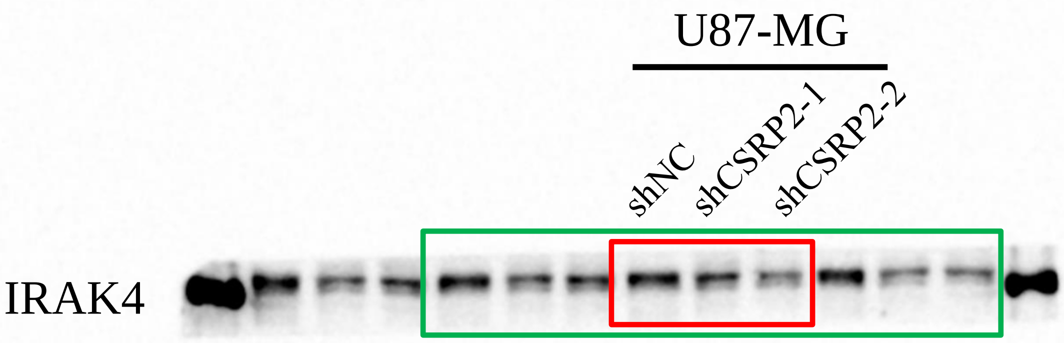

The membrane was imaged with Azure Biosystems 300

Full unedited gel for Figure 4F

Green： Statistical graph

Red： Representative graph

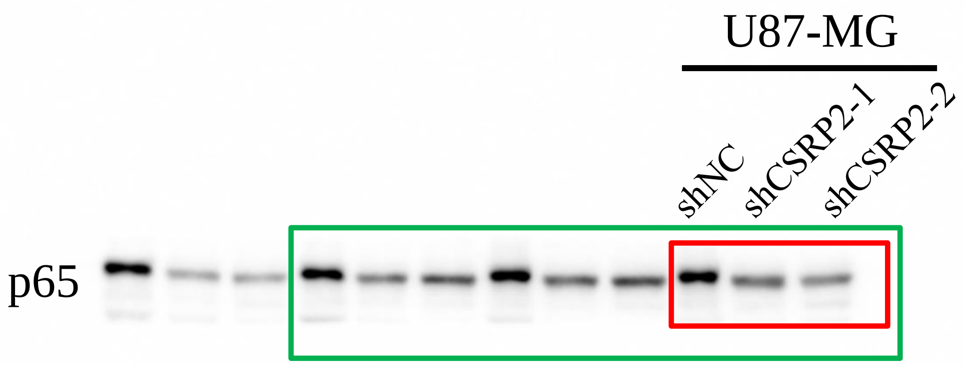

The membrane was imaged with Azure Biosystems 300

Full unedited gel for Figure 4F

Green： Statistical graph

Red： Representative graph

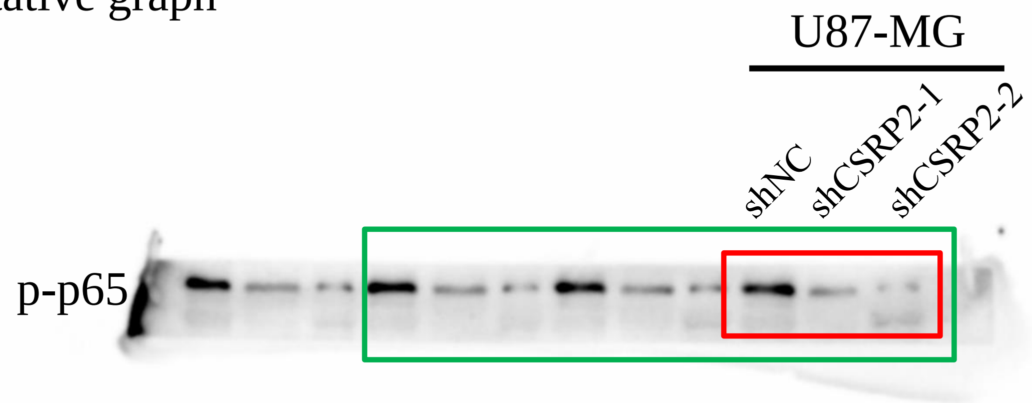

The membrane was imaged with Azure Biosystems 300

Full unedited gel for Figure 4F

Green： Statistical graph

Red： Representative graph

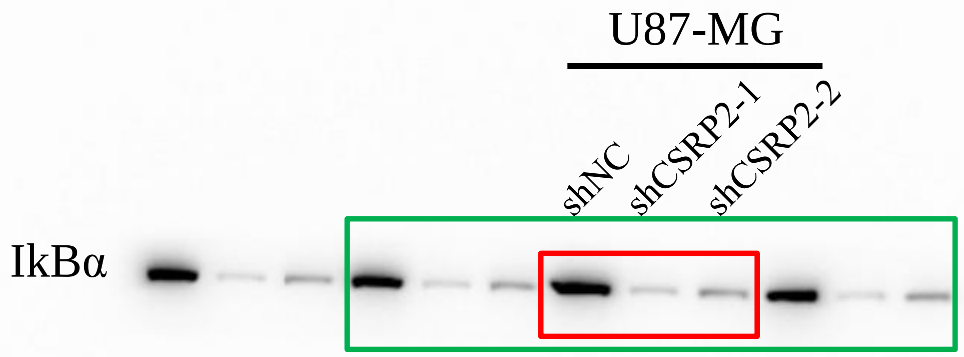

The membrane was imaged with Azure Biosystems 300

Full unedited gel for Figure 4F

Green： Statistical graph

Red： Representative graph

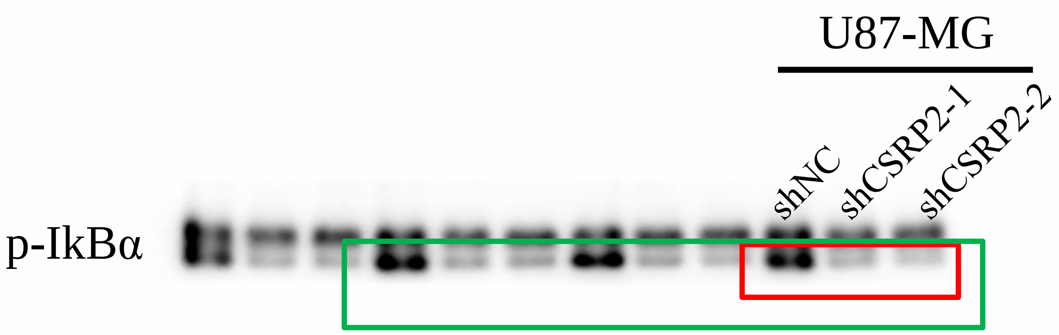

The membrane was imaged with Azure Biosystems 300

Full unedited gel for Figure 4F

Green： Statistical graph

Red： Representative graph

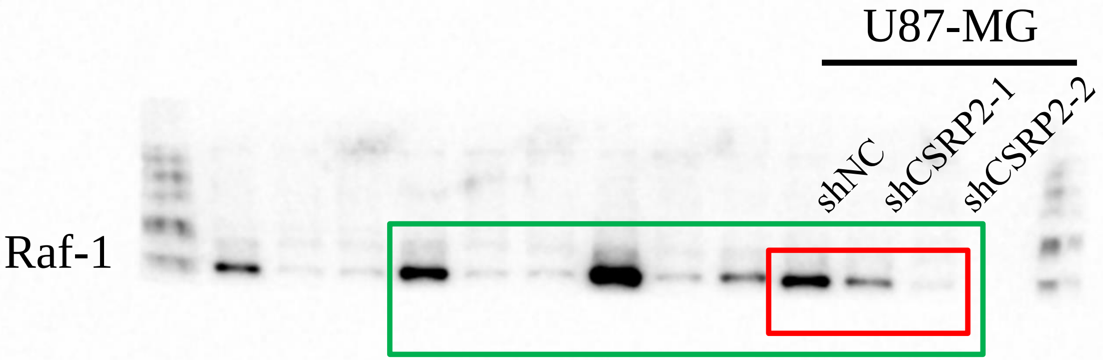

The membrane was imaged with Azure Biosystems 300

Full unedited gel for Figure 4F

Green： Statistical graph

Red： Representative graph

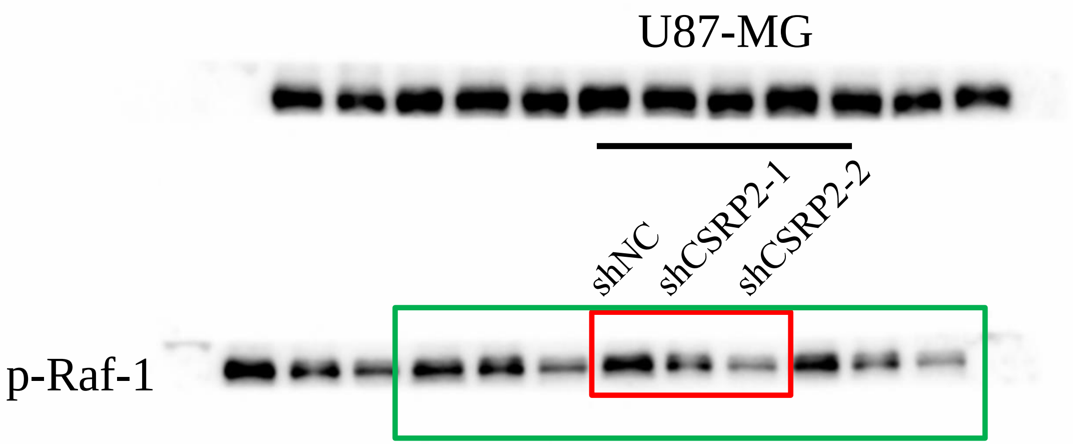

The membrane was imaged with Azure Biosystems 300

Full unedited gel for Figure 4F

Green： Statistical graph

Red： Representative graph

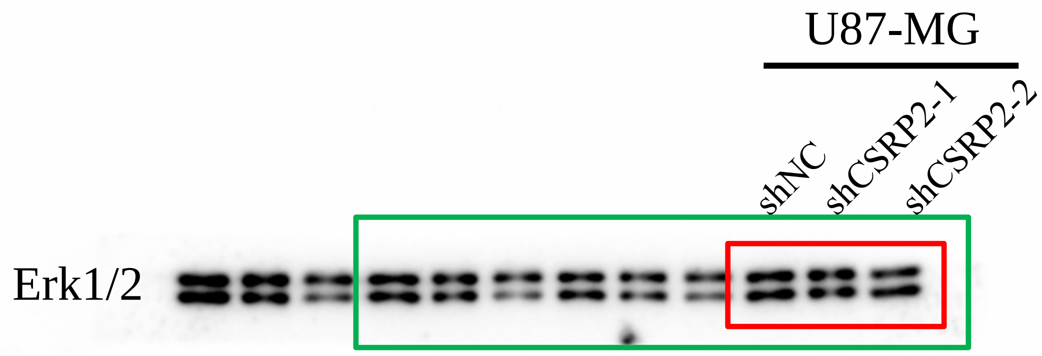

The membrane was imaged with Azure Biosystems 300

Full unedited gel for Figure 4F

Green： Statistical graph

Red： Representative graph

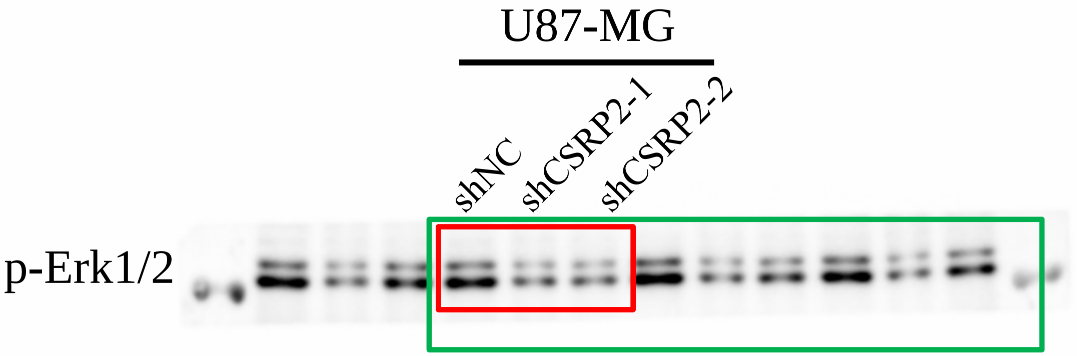

The membrane was imaged with Azure Biosystems 300

Full unedited gel for Figure 4F

Green： Statistical graph

Red： Representative graph

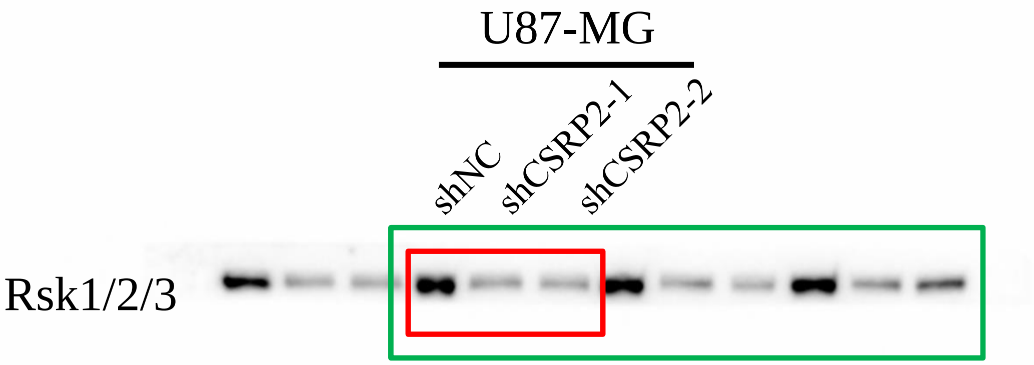

The membrane was imaged with Azure Biosystems 300

Full unedited gel for Figure 4F

Green： Statistical graph

Red： Representative graph

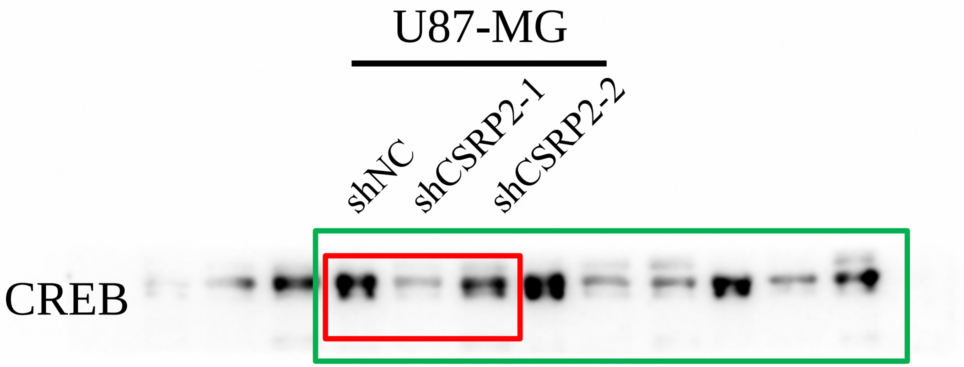

The membrane was imaged with Azure Biosystems 300

Full unedited gel for Figure 4F

Green： Statistical graph

Red： Representative graph

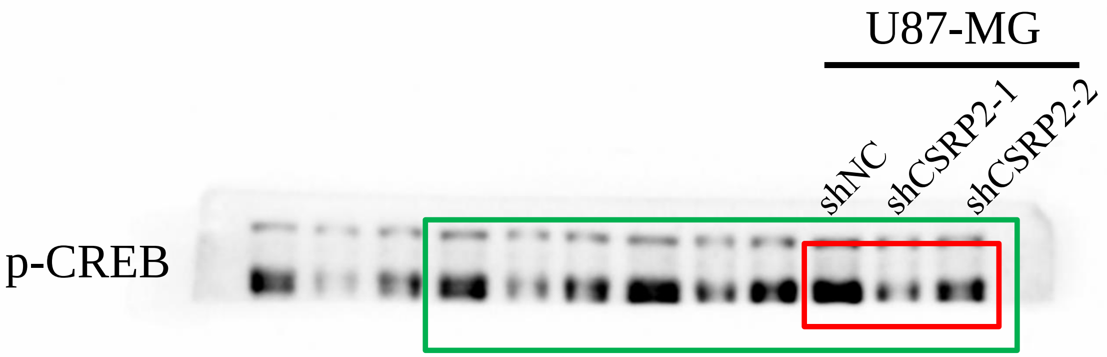

The membrane was imaged with Azure Biosystems 300

Full unedited gel for Figure 4F

Green： Statistical graph

Red： Representative graph

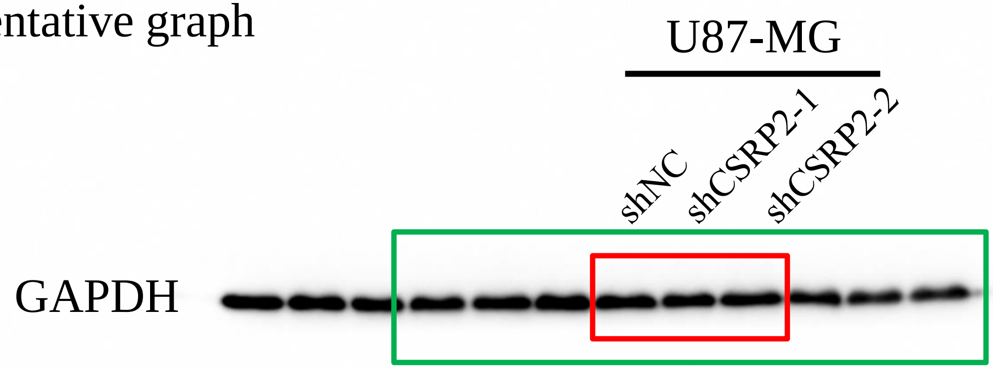

The membrane was imaged with Azure Biosystems 300

Full unedited gel for Figure 4F

Green： Statistical graph

Red： Representative graph

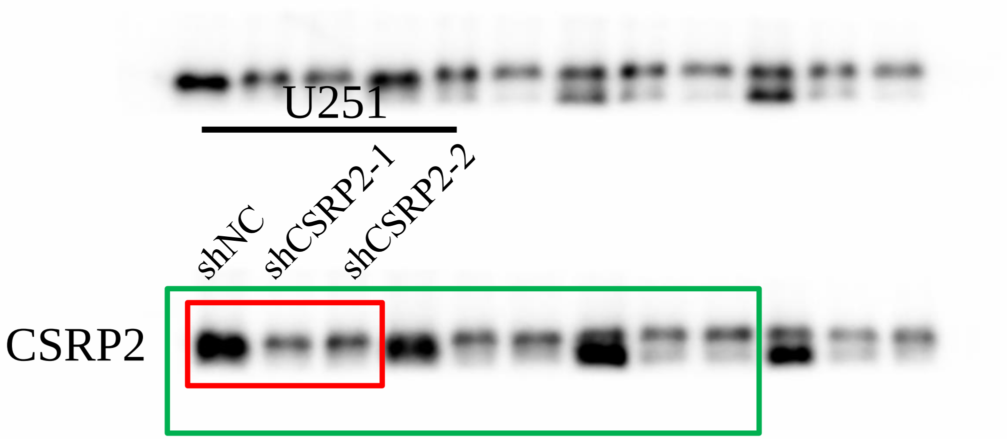

The membrane was imaged with Azure Biosystems 300

Full unedited gel for Figure 4F

Green： Statistical graph

Red： Representative graph

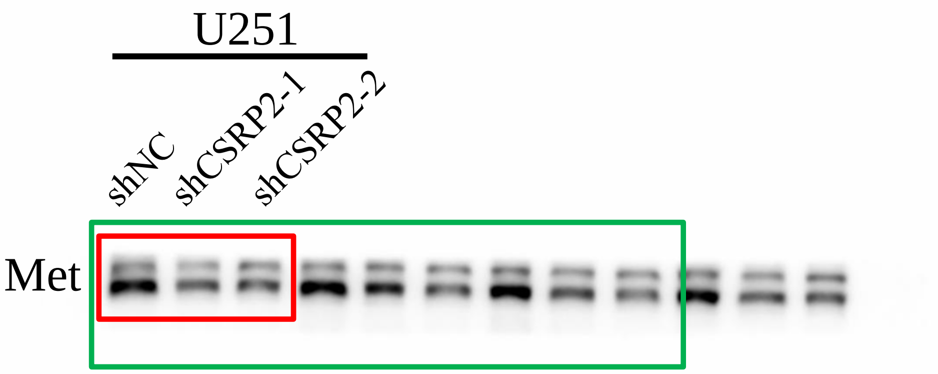

The membrane was imaged with Azure Biosystems 300

Full unedited gel for Figure 4F

Green： Statistical graph

Red： Representative graph

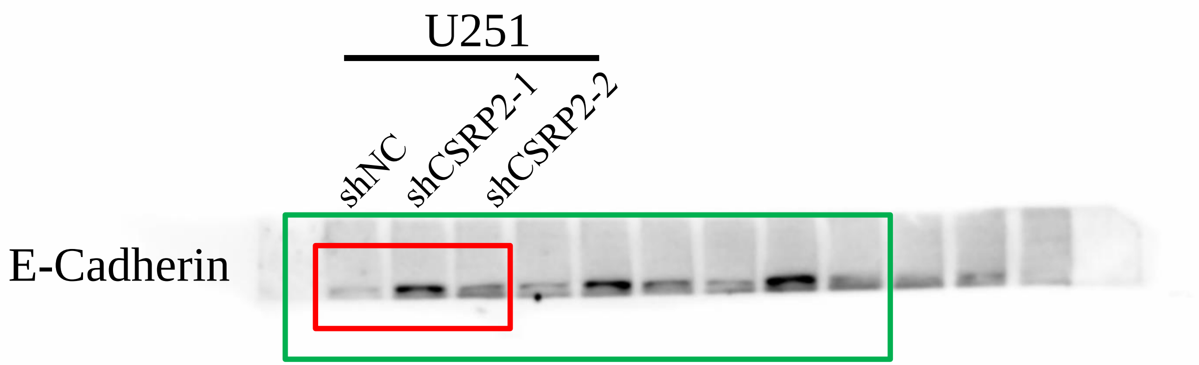

The membrane was imaged with Azure Biosystems 300

Full unedited gel for Figure 4F

Green： Statistical graph

Red： Representative graph

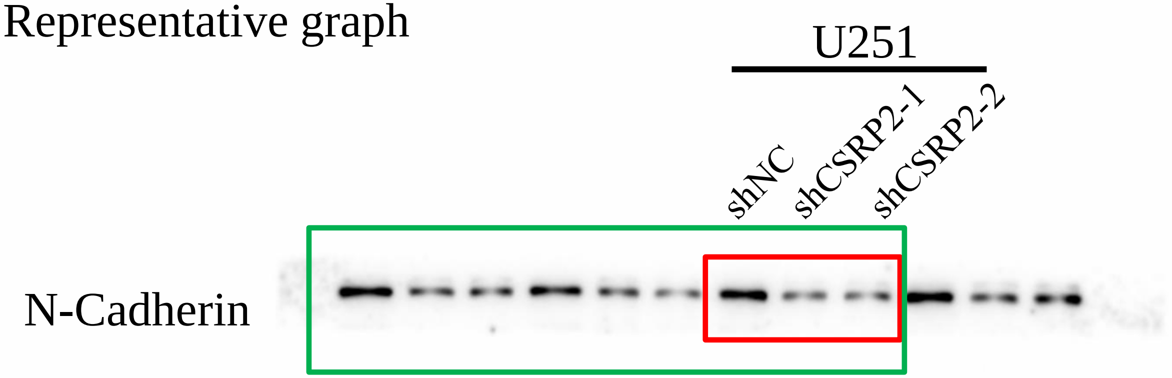

The membrane was imaged with Azure Biosystems 300

Full unedited gel for Figure 4F

Green： Statistical graph

Red： Representative graph

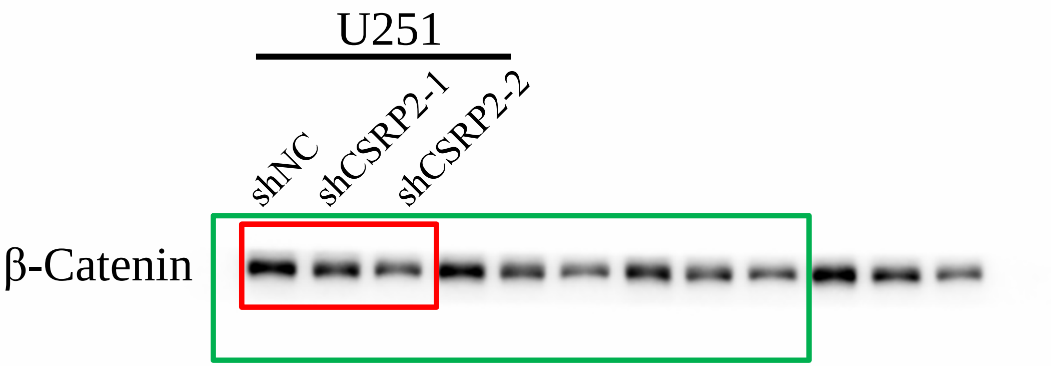

The membrane was imaged with Azure Biosystems 300

Full unedited gel for Figure 4F

Green： Statistical graph

Red： Representative graph

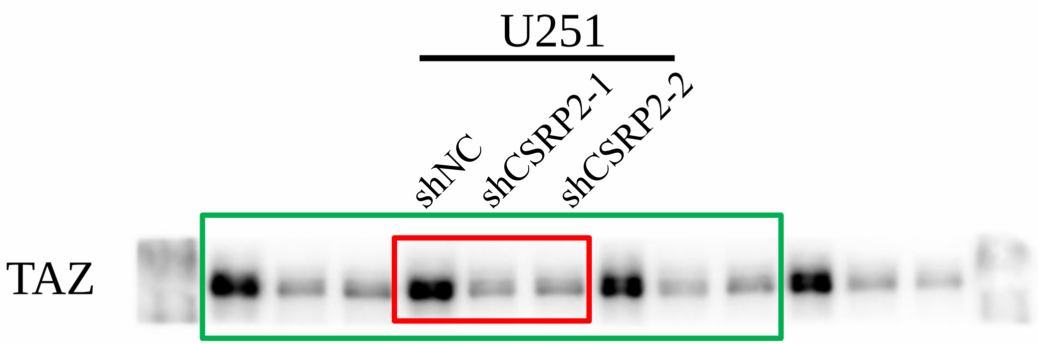

The membrane was imaged with Azure Biosystems 300

Full unedited gel for Figure 4F

Green： Statistical graph

Red： Representative graph

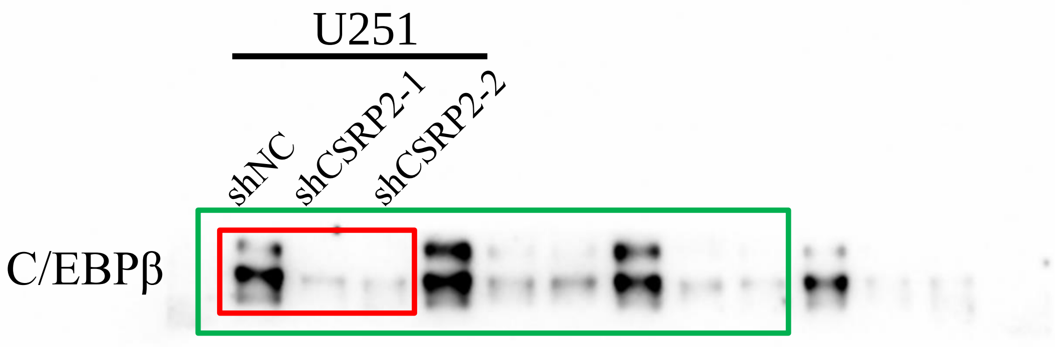

The membrane was imaged with Azure Biosystems 300

Full unedited gel for Figure 4F

Green: Statistical graph

Red: Representative graph

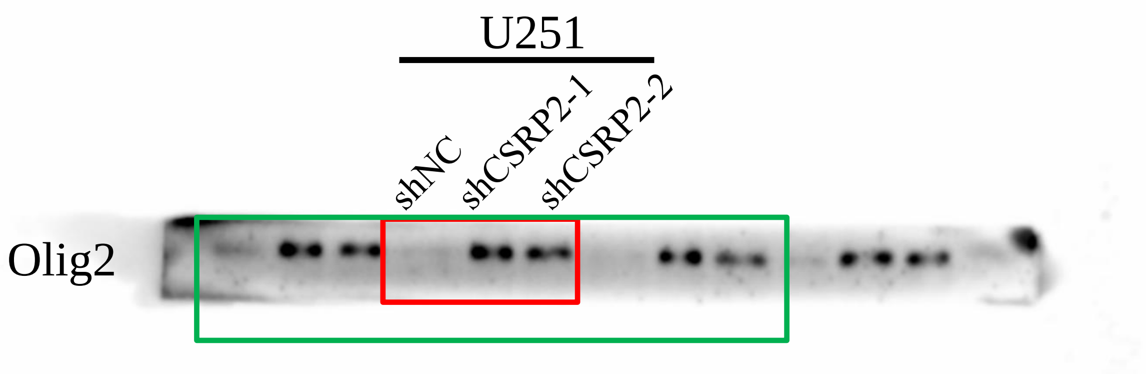

The membrane was imaged with Azure Biosystems 300

Full unedited gel for Figure 4F

Green： Statistical graph

Red： Representative graph

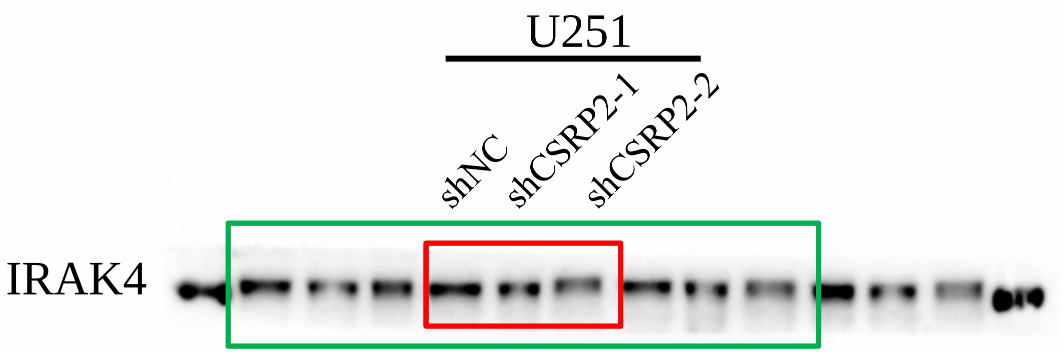

The membrane was imaged with Azure Biosystems 300

Full unedited gel for Figure 4F

Green： Statistical graph

Red： Representative graph

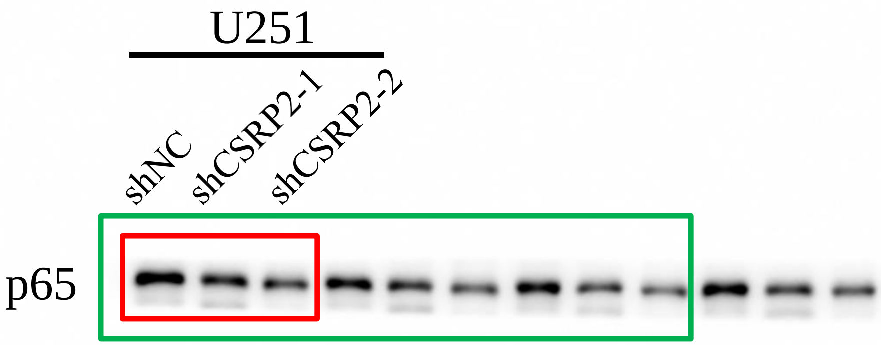

The membrane was imaged with Azure Biosystems 300

Full unedited gel for Figure 4F

Green： Statistical graph

Red： Representative graph

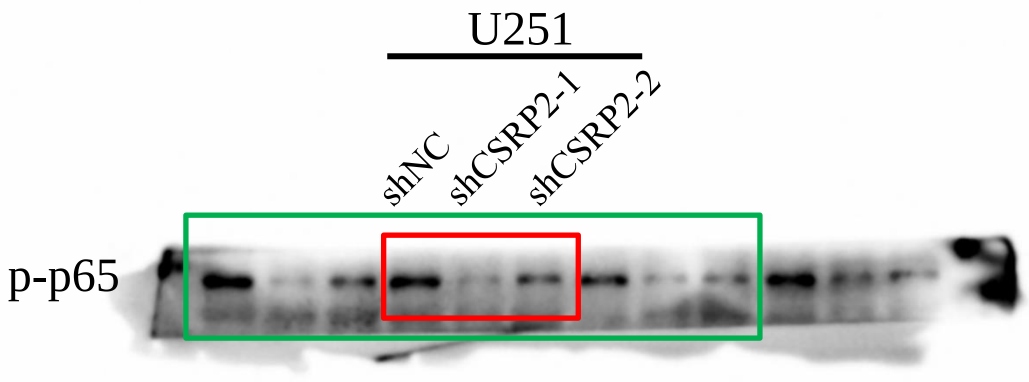

The membrane was imaged with Azure Biosystems 300

Full unedited gel for Figure 4F

Green： Statistical graph

Red： Representative graph

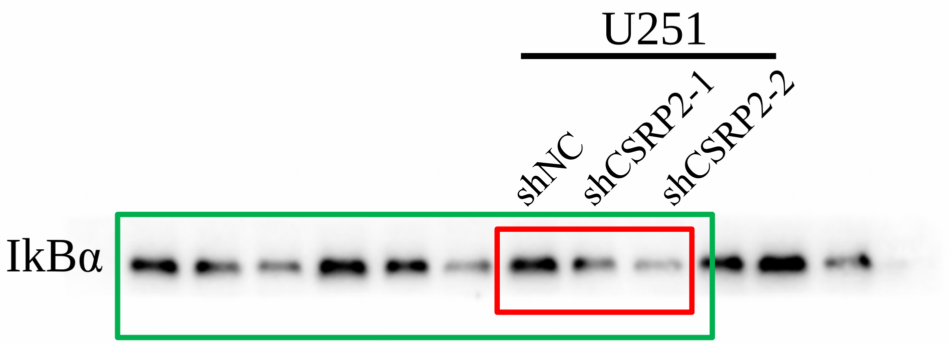

The membrane was imaged with Azure Biosystems 300

Full unedited gel for Figure 4F

Green： Statistical graph

Red： Representative graph

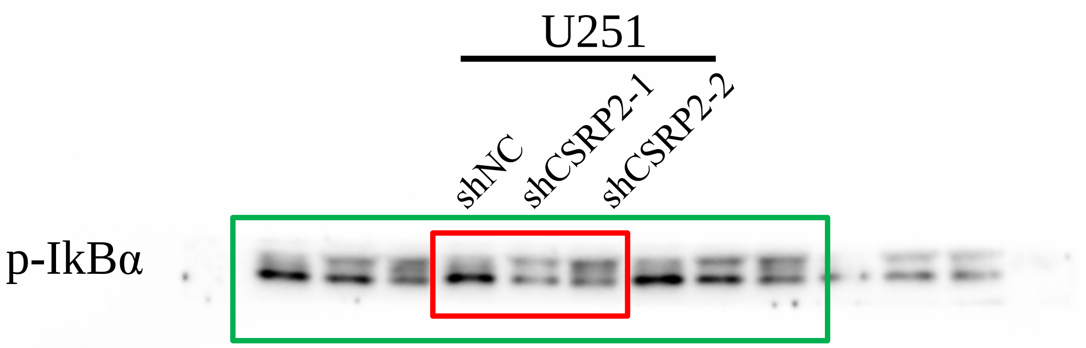

The membrane was imaged with Azure Biosystems 300

Full unedited gel for Figure 4F

Green： Statistical graph

Red： Representative graph

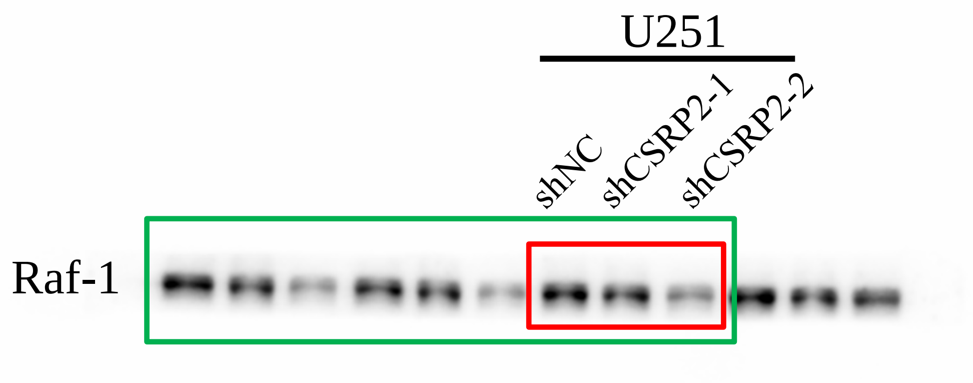

The membrane was imaged with Azure Biosystems 300

Full unedited gel for Figure 4F

Green： Statistical graph

Red： Representative graph

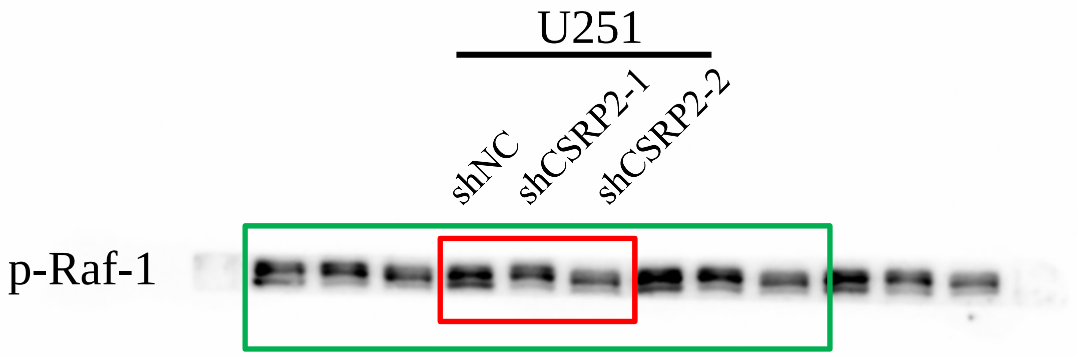

The membrane was imaged with Azure Biosystems 300

Full unedited gel for Figure 4F

Green： Statistical graph

Red： Representative graph

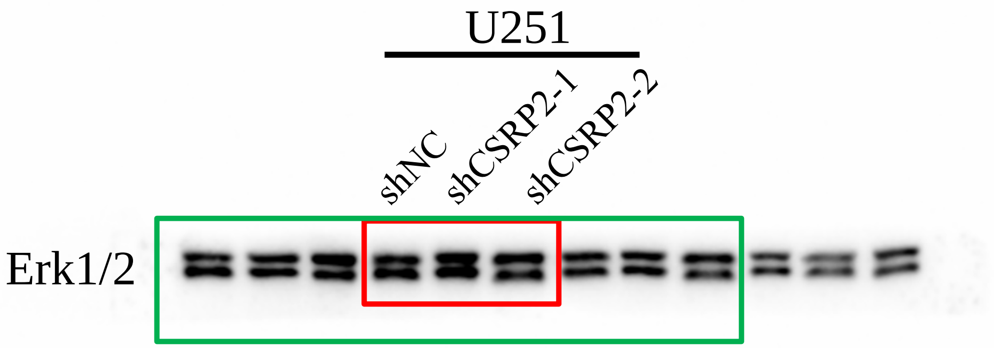

The membrane was imaged with Azure Biosystems 300

Full unedited gel for Figure 4F

Green： Statistical graph

Red： Representative graph

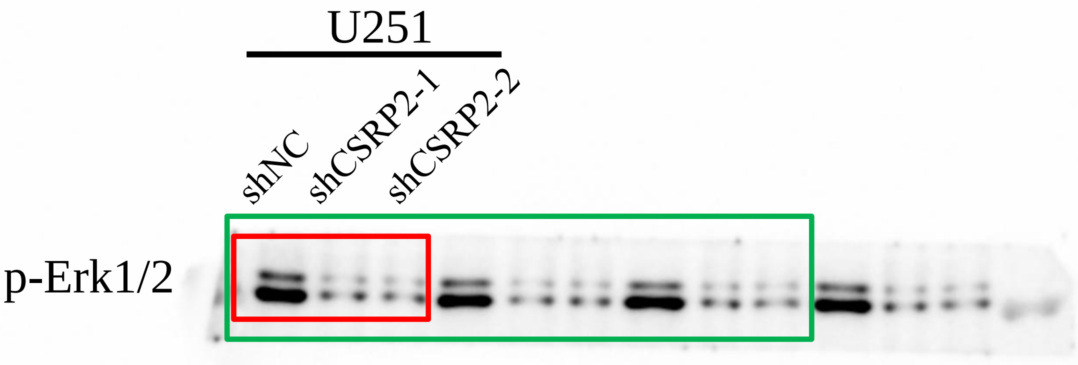

The membrane was imaged with Azure Biosystems 300

Full unedited gel for Figure 4F

Green： Statistical graph

Red： Representative graph

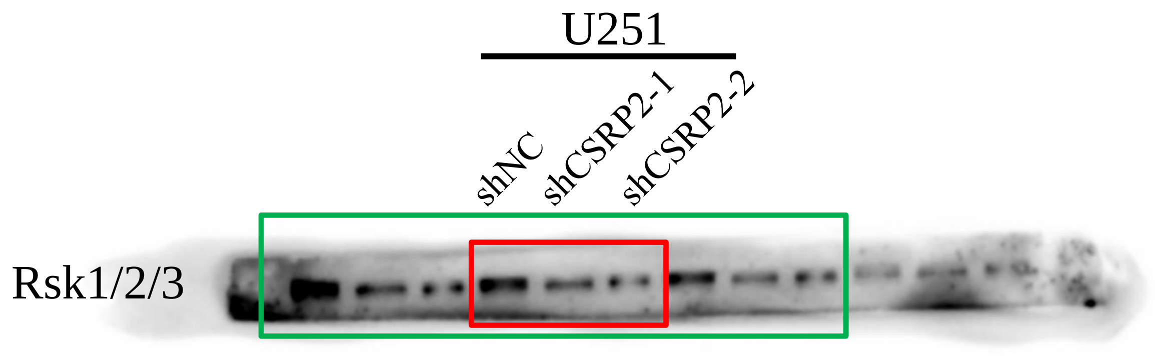

The membrane was imaged with Azure Biosystems 300

Full unedited gel for Figure 4F

Green： Statistical graph

Red： Representative graph

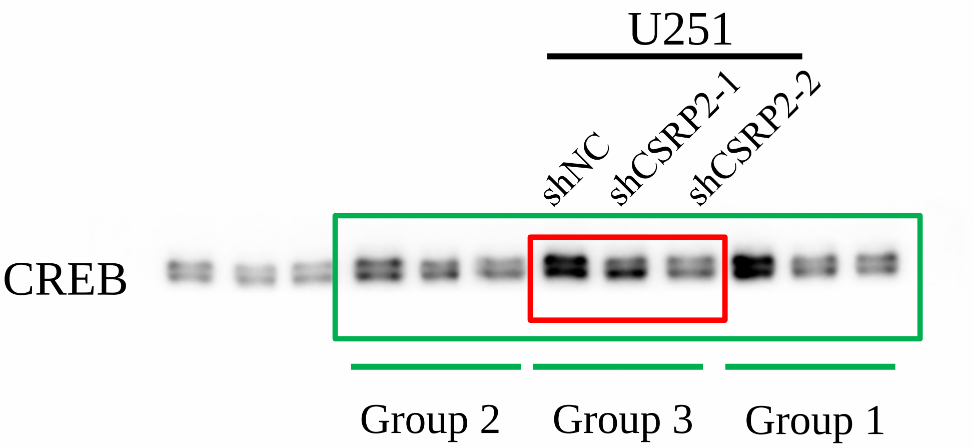

The membrane was imaged with Azure Biosystems 300

Full unedited gel for Figure 4F

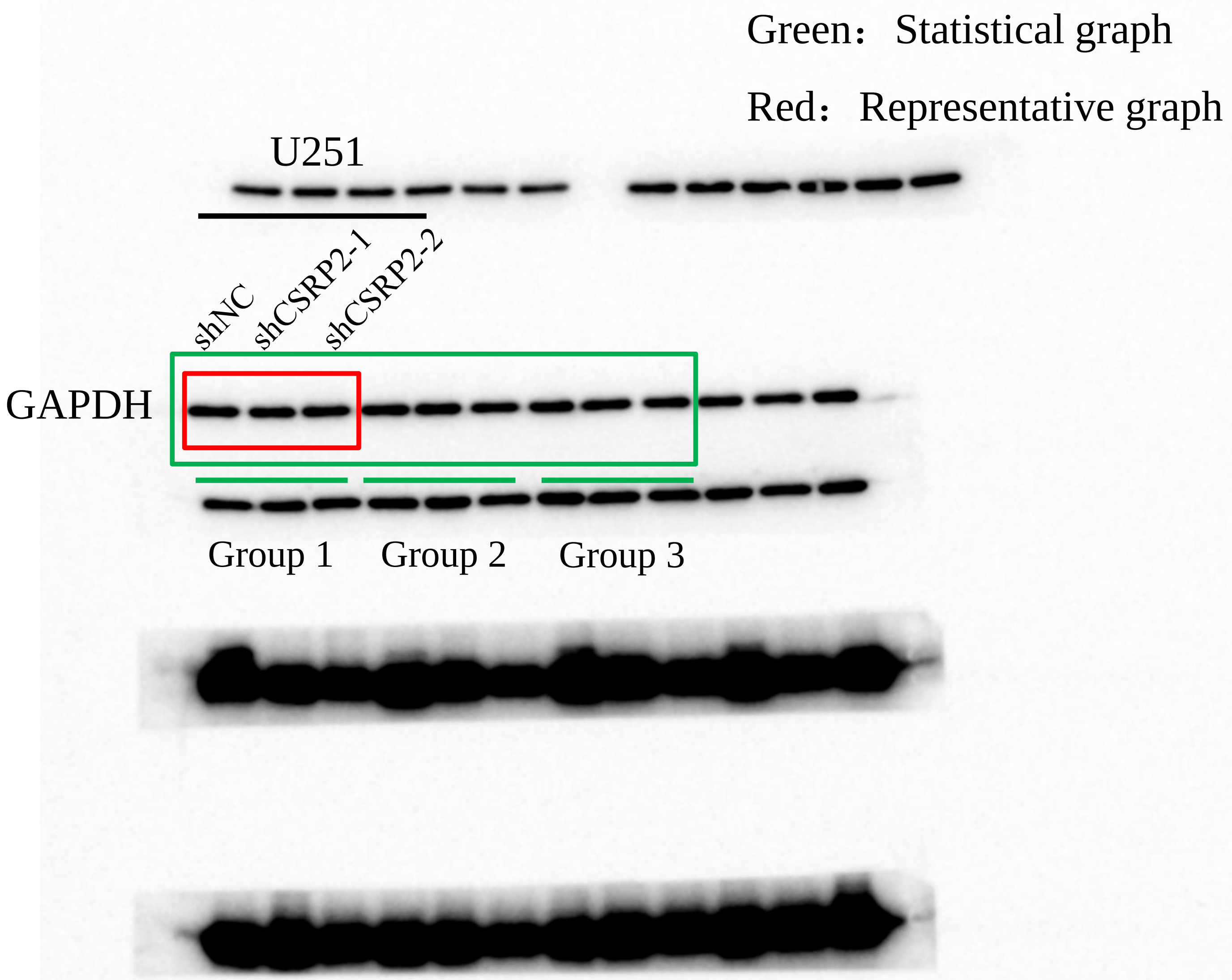

The membrane was imaged with Azure Biosystems 300

Full unedited gel for Figure 4F

Green： Statistical graph

Red： Representative graph

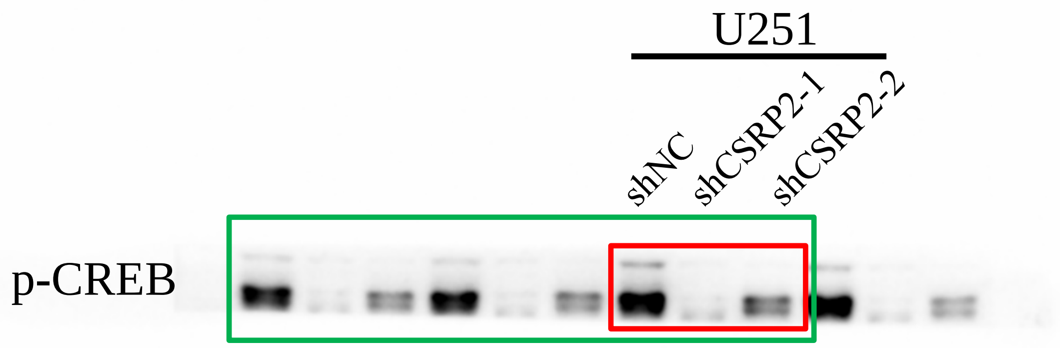

The membrane was imaged with Azure Biosystems 300

Full unedited gel for Figure 4F

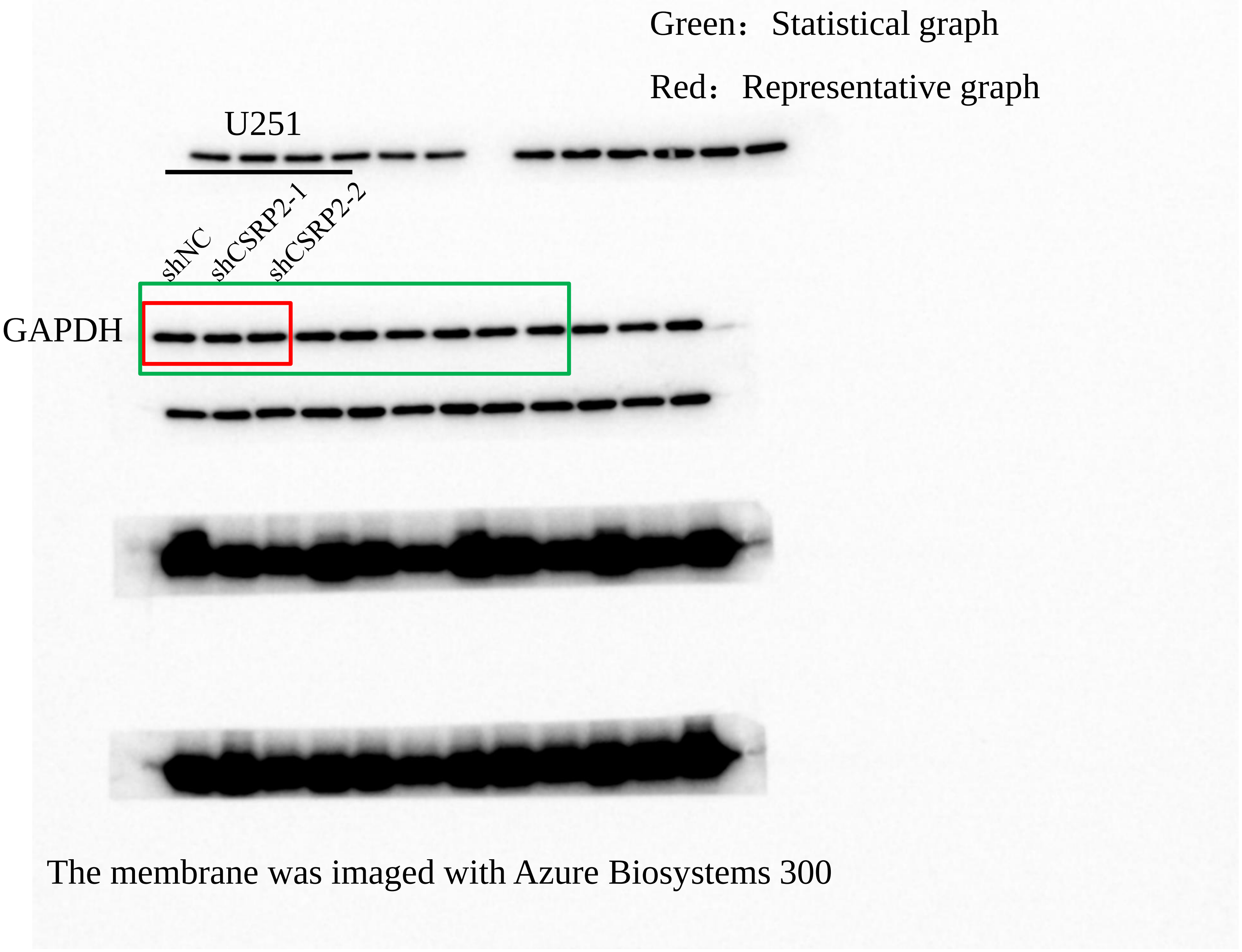

The membrane was imaged with Azure Biosystems 300

Full unedited gel for Figure 5K

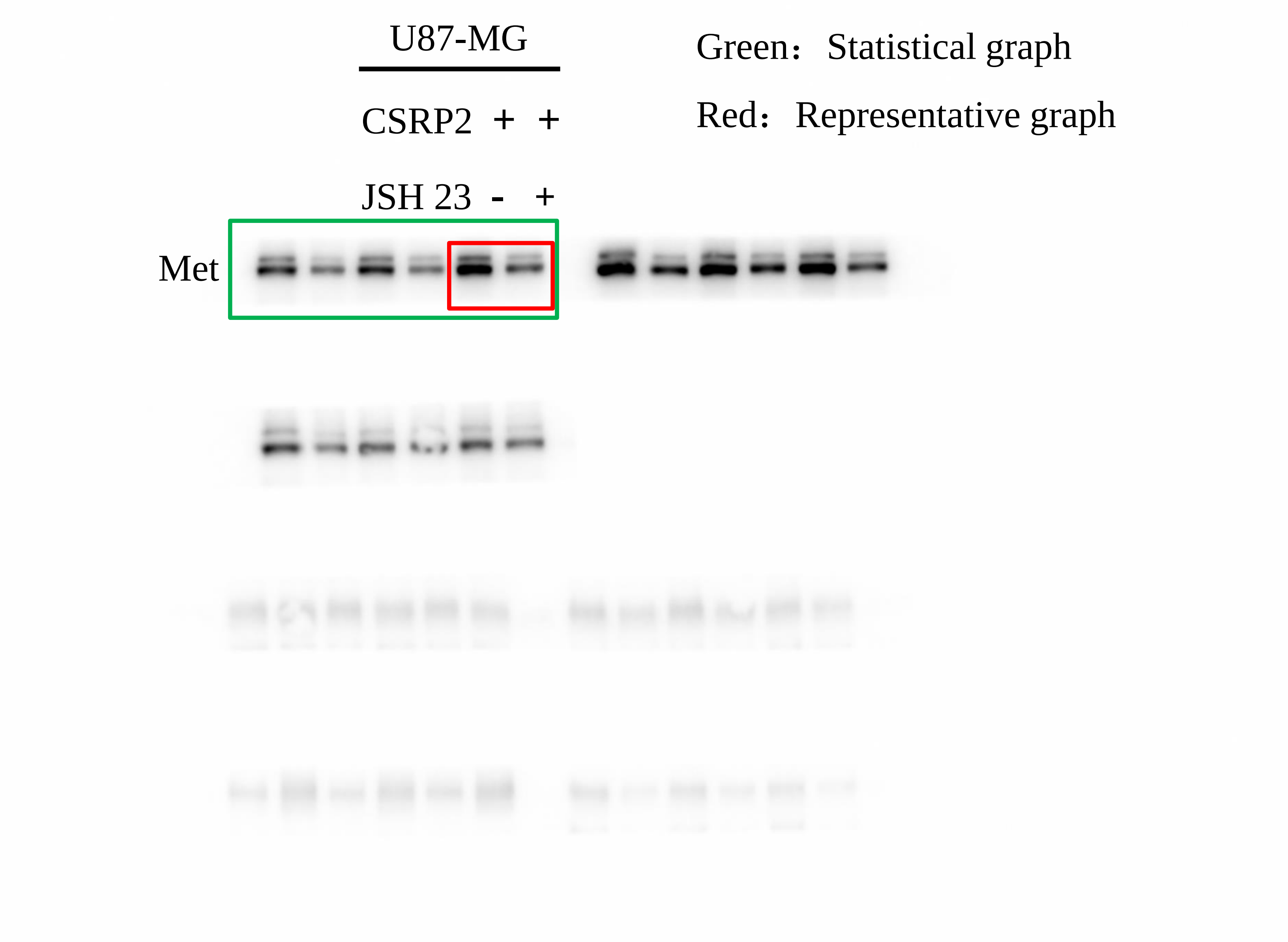

The membrane was imaged with Azure Biosystems 300

Full unedited gel for Figure 5K

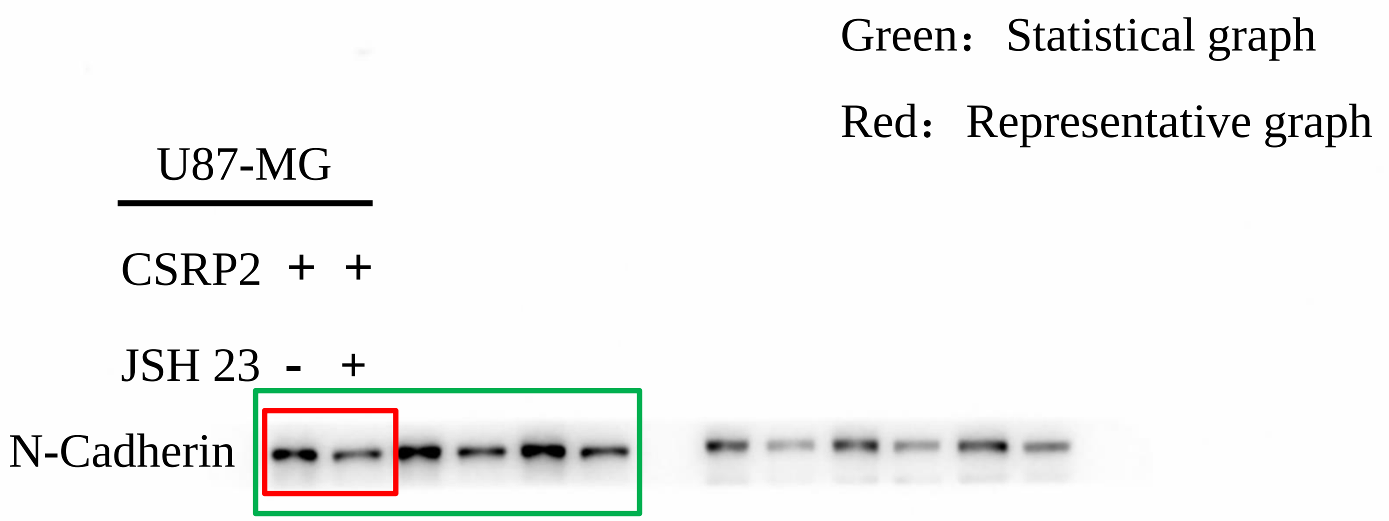

The membrane was imaged with Azure Biosystems 300

Full unedited gel for Figure 5K

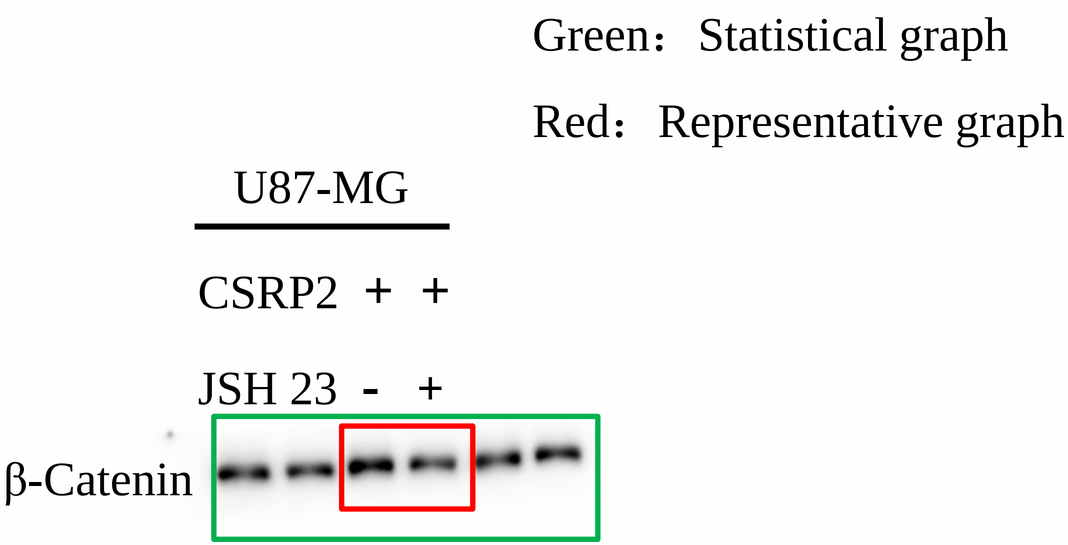

The membrane was imaged with Azure Biosystems 300

Full unedited gel for Figure 5K

Green： Statistical graph

Red： Representative graph

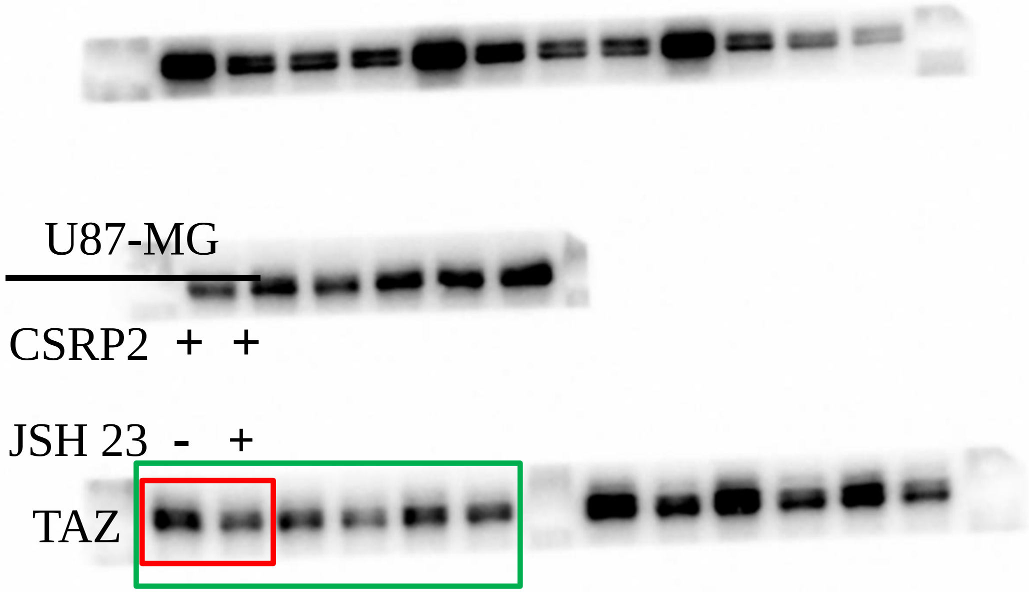

The membrane was imaged with Azure Biosystems 300

Full unedited gel for Figure 5K

Green： Statistical graph

Red： Representative graph

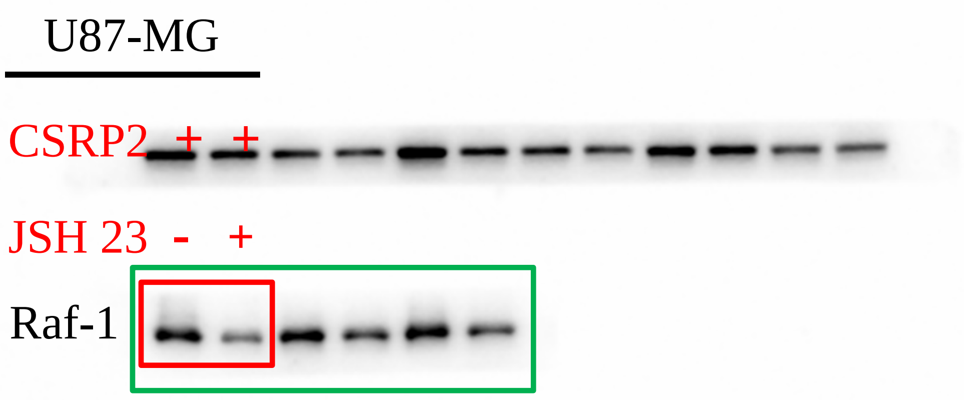

The membrane was imaged with Azure Biosystems 300

Full unedited gel for Figure 5K

Green： Statistical graph

Red： Representative graph

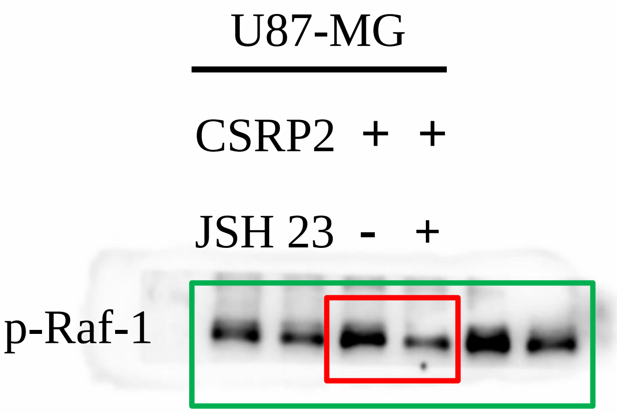

The membrane was imaged with Azure Biosystems 300

Full unedited gel for Figure 5K

Green: Statistical graph

Red: Representative graph

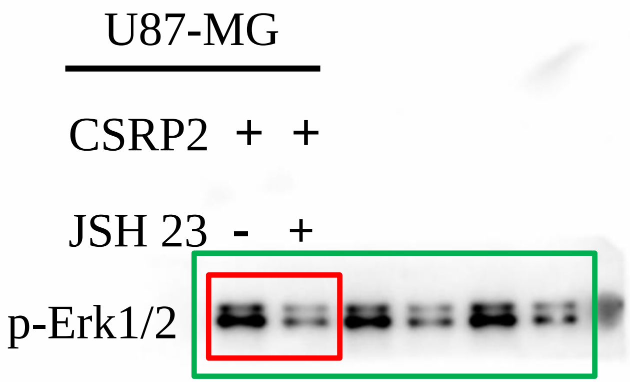

The membrane was imaged with Azure Biosystems 300

Full unedited gel for Figure 5K

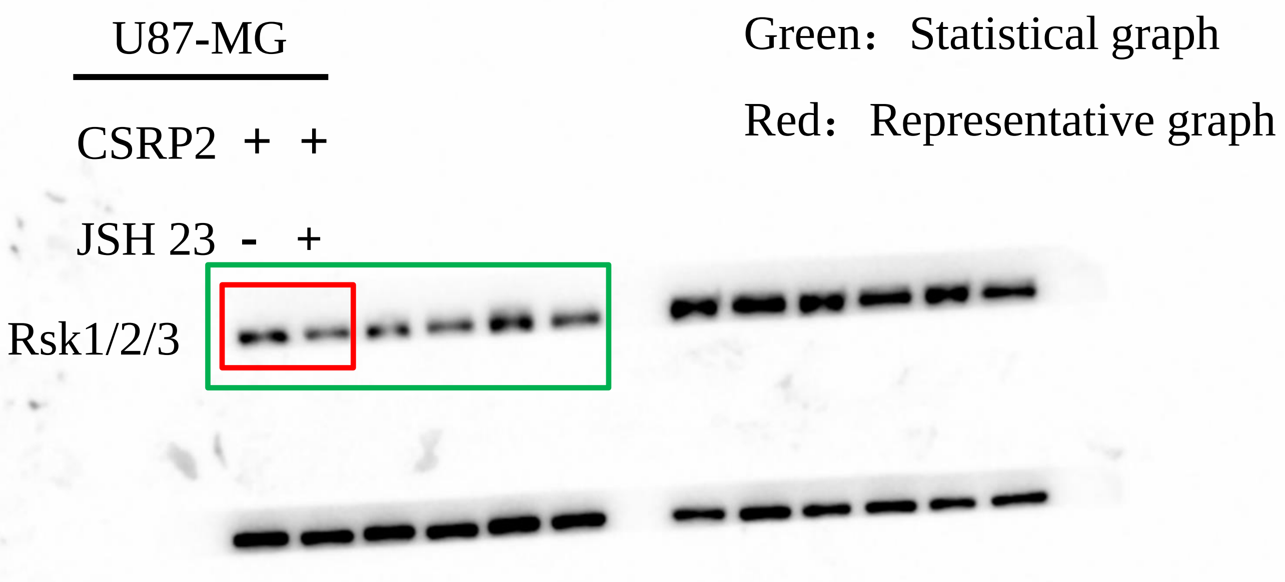

The membrane was imaged with Azure Biosystems 300

Full unedited gel for Figure 5K

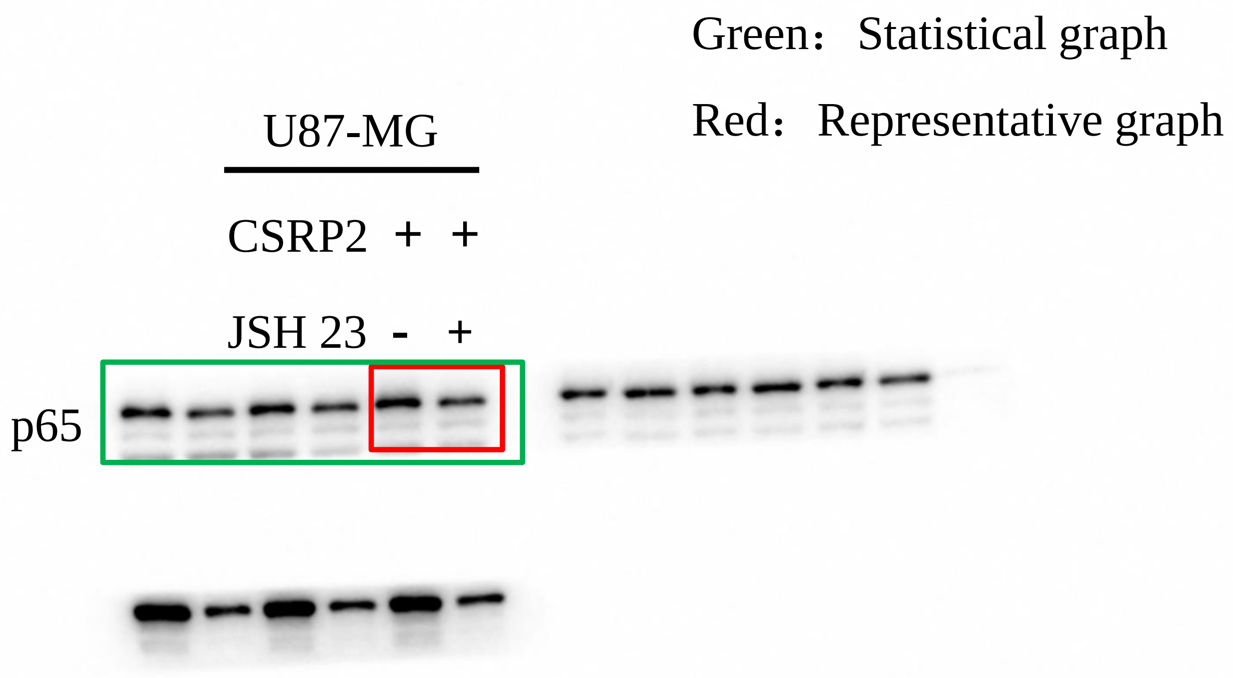

The membrane was imaged with Azure Biosystems 300

Full unedited gel for Figure 5K

Green： Statistical graph

Red： Representative graph

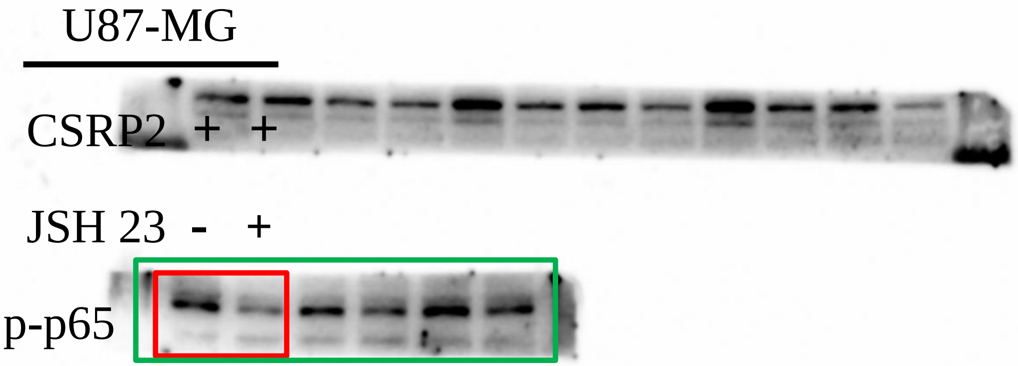

The membrane was imaged with Azure Biosystems 300

Full unedited gel for Figure 5K

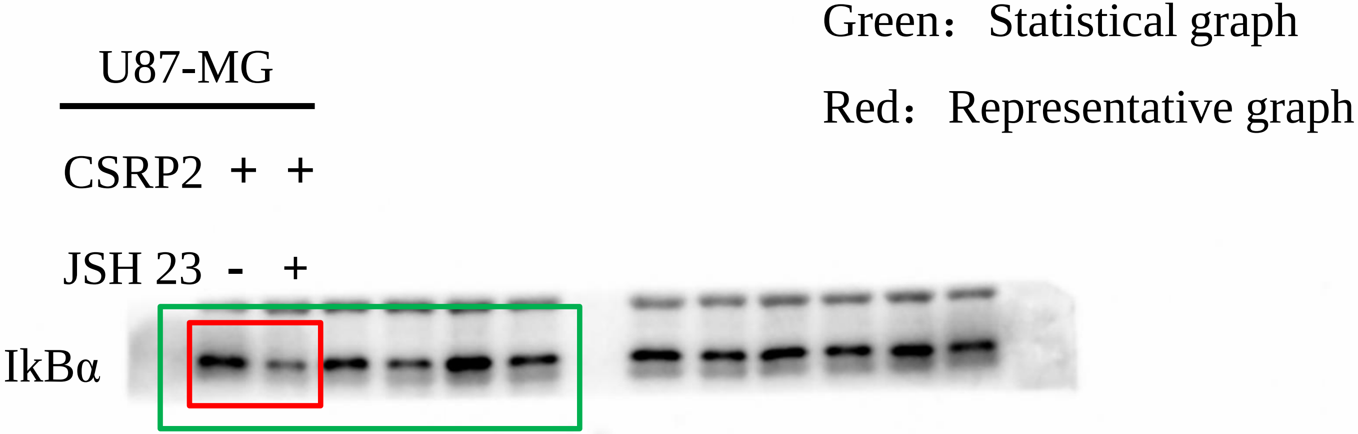

The membrane was imaged with Azure Biosystems 300

Full unedited gel for Figure 5K

Green： Statistical graph

Red： Representative graph

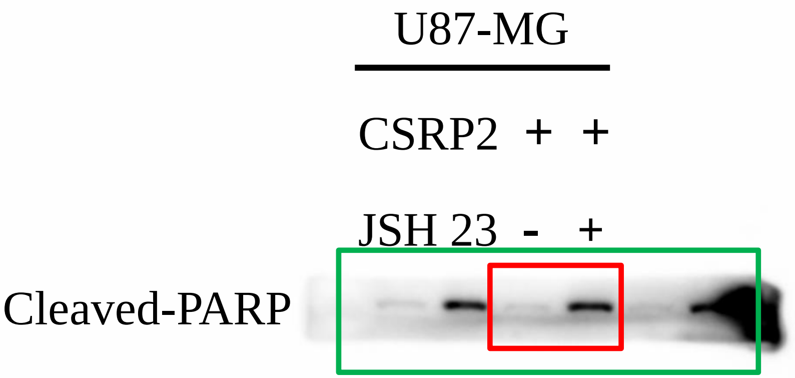

The membrane was imaged with Azure Biosystems 300

Full unedited gel for Figure 5K

Green： Statistical graph

Red： Representative graph

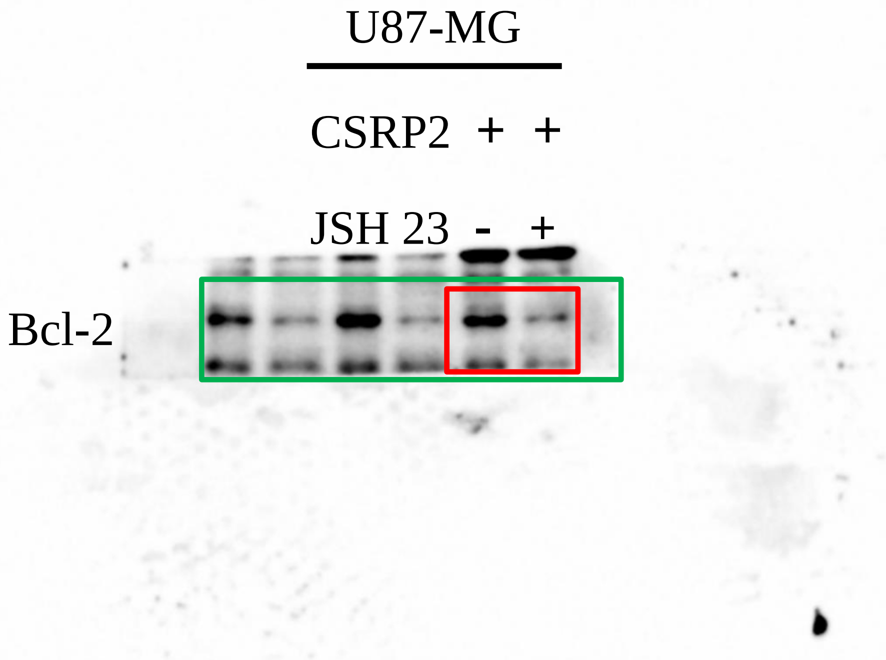

The membrane was imaged with Azure Biosystems 300

Full unedited gel for Figure 5K

Green：Statistical graph

Red：Representative graph

U87-MG

CSRP2 + +

JSH 23 - +

Bcl-xL

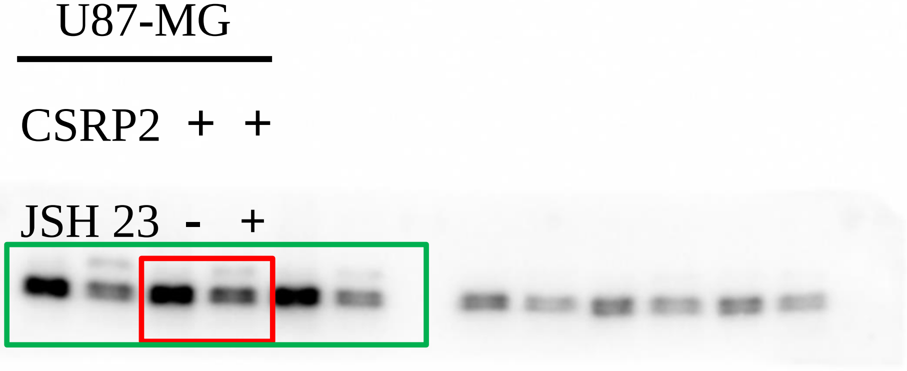

The membrane was imaged with Azure Biosystems 300

Full unedited gel for Figure 5K

Green: Statistical graph

Red: Representative graph

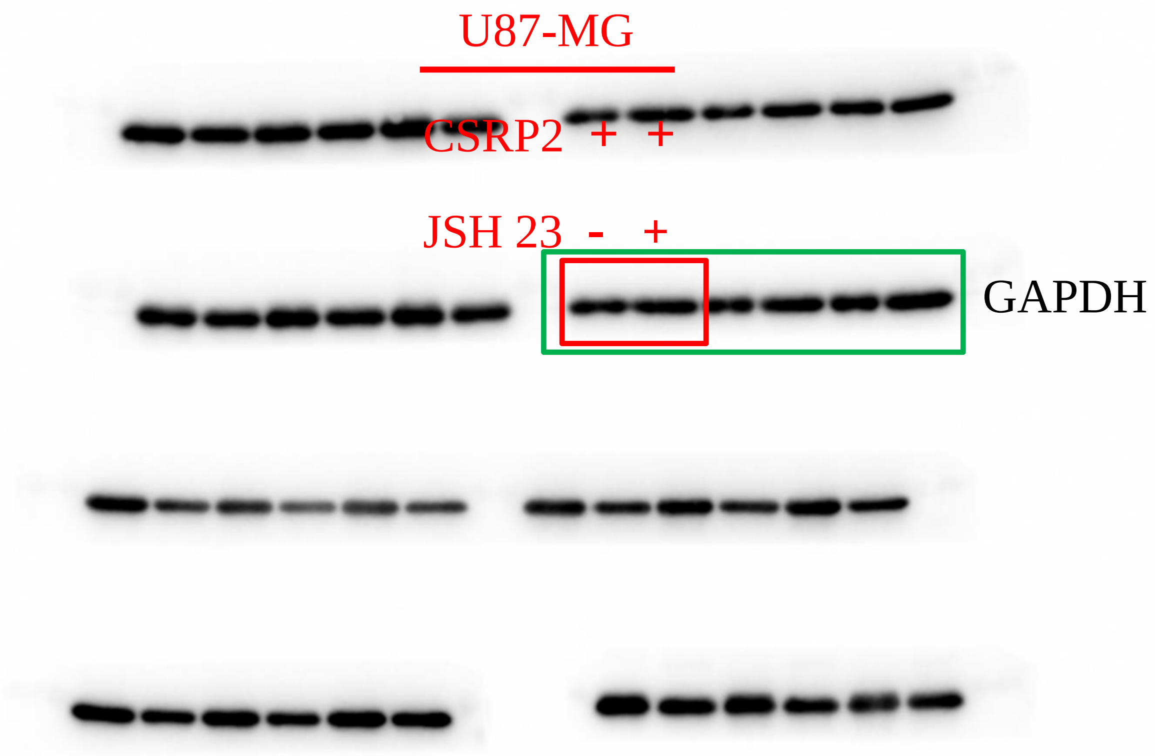

The membrane was imaged with Azure Biosystems 300

Full unedited gel for Figure 5K

Green： Statistical graph

Red： Representative graph

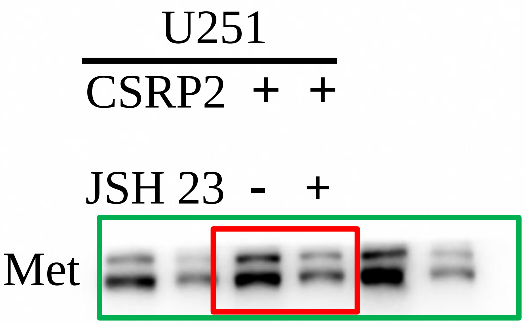

The membrane was imaged with Azure Biosystems 300

Full unedited gel for Figure 5K

Green： Statistical graph

Red： Representative graph

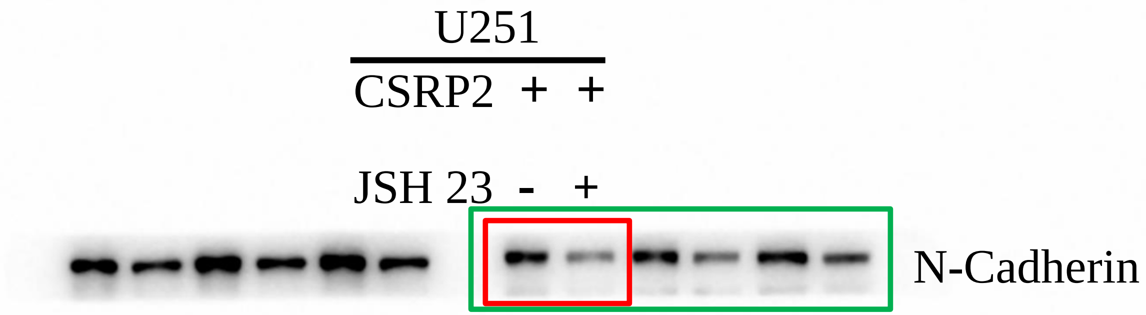

The membrane was imaged with Azure Biosystems 300

Full unedited gel for Figure 5K

Green: Statistical graph

Red: Representative graph

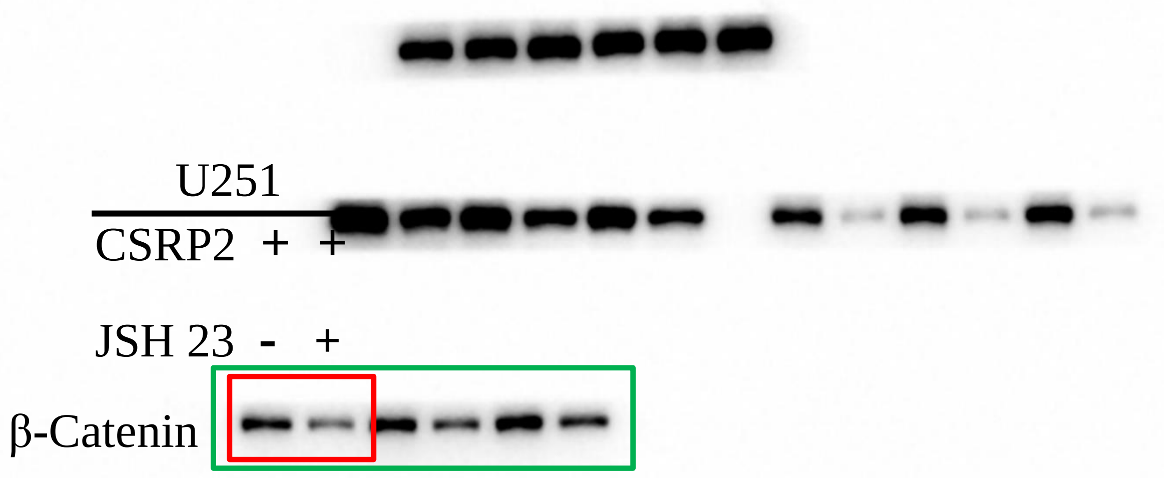

The membrane was imaged with Azure Biosystems 300

Full unedited gel for Figure 5K

Green: Statistical graph

Red: Representative graph

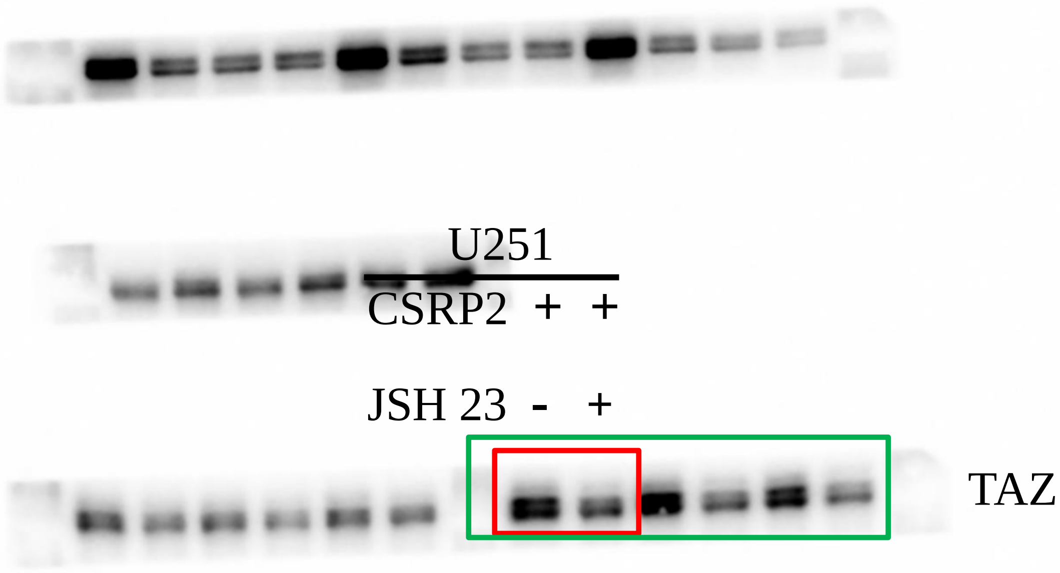

The membrane was imaged with Azure Biosystems 300

Full unedited gel for Figure 5K

Green： Statistical graph

Red： Representative graph

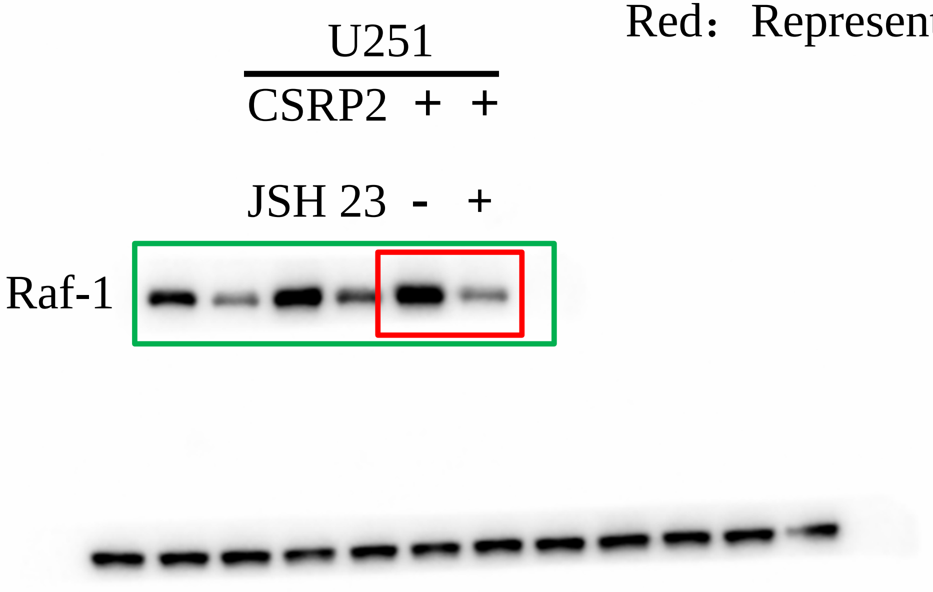

The membrane was imaged with Azure Biosystems 300

Full unedited gel for Figure 5K

Green：Statistical graph

Red：Representative graph

U251

CSRP2 + +

JSH 23 - +

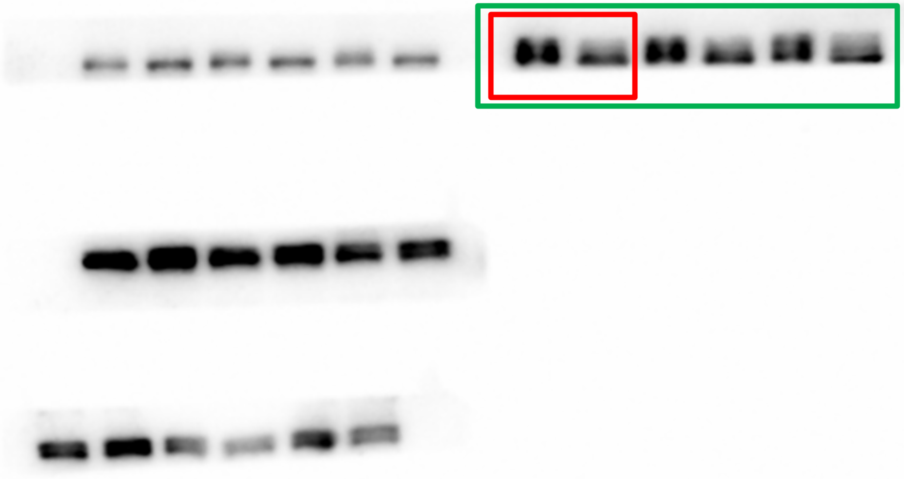

p-Raf-1

The membrane was imaged with Azure Biosystems 300

Full unedited gel for Figure 5K

Green： Statistical graph

Red： Representative graph

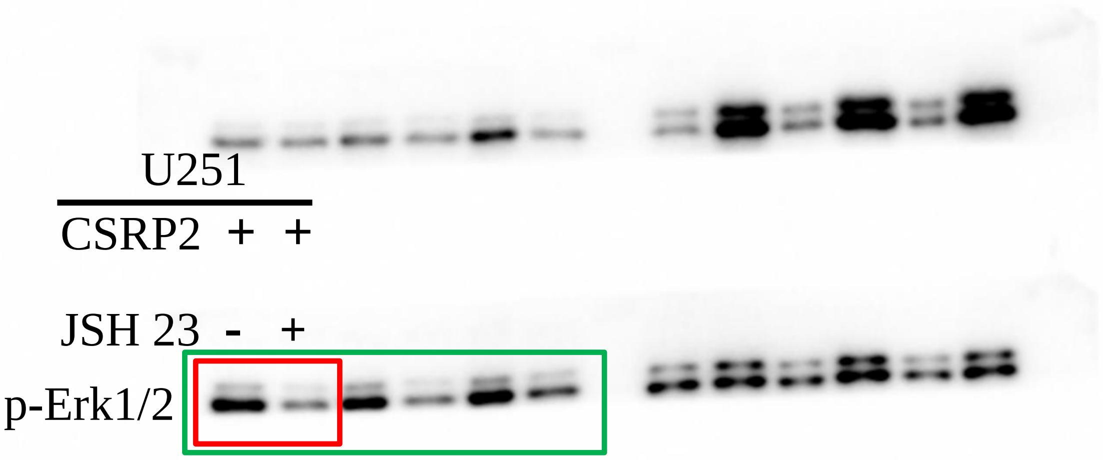

The membrane was imaged with Azure Biosystems 300

Full unedited gel for Figure 5K

Green： Statistical graph

Red： Representative graph

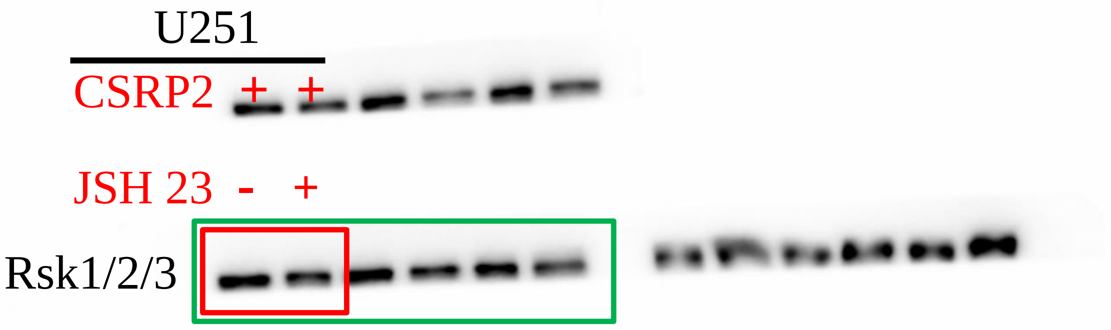

The membrane was imaged with Azure Biosystems 300

Full unedited gel for Figure 5K

Green： Statistical graph

Red： Representative graph

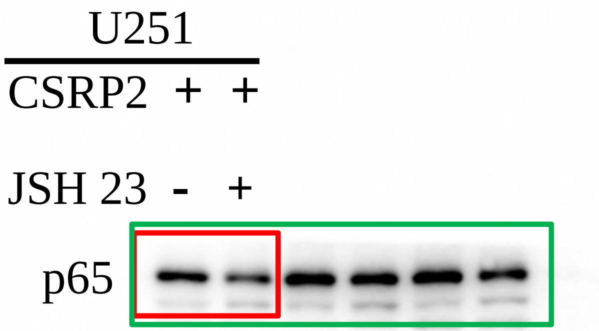

The membrane was imaged with Azure Biosystems 300

Full unedited gel for Figure 5K

Green： Statistical graph

Red： Representative graph

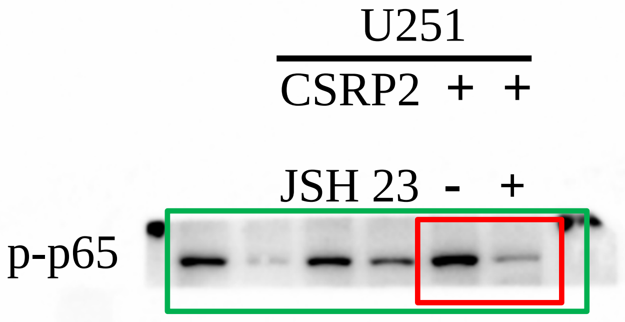

The membrane was imaged with Azure Biosystems 300

Full unedited gel for Figure 5K

Green： Statistical graph

Red： Representative graph

U251

CSRP2 + +

JSH 23 - +

IkBα

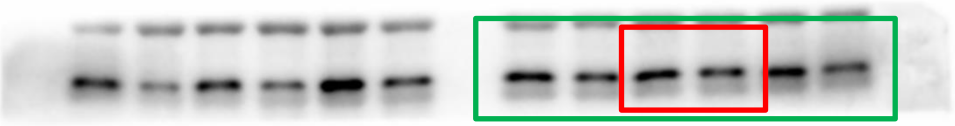

The membrane was imaged with Azure Biosystems 300

Full unedited gel for Figure 5K

Green: Statistical graph

Red: Representative graph

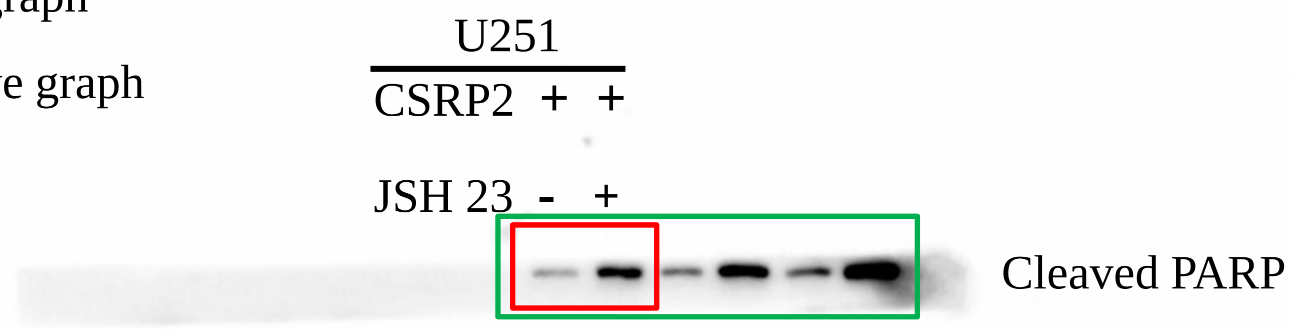

The membrane was imaged with Azure Biosystems 300

Full unedited gel for Figure 5K

Green： Statistical graph

Red： Representative graph

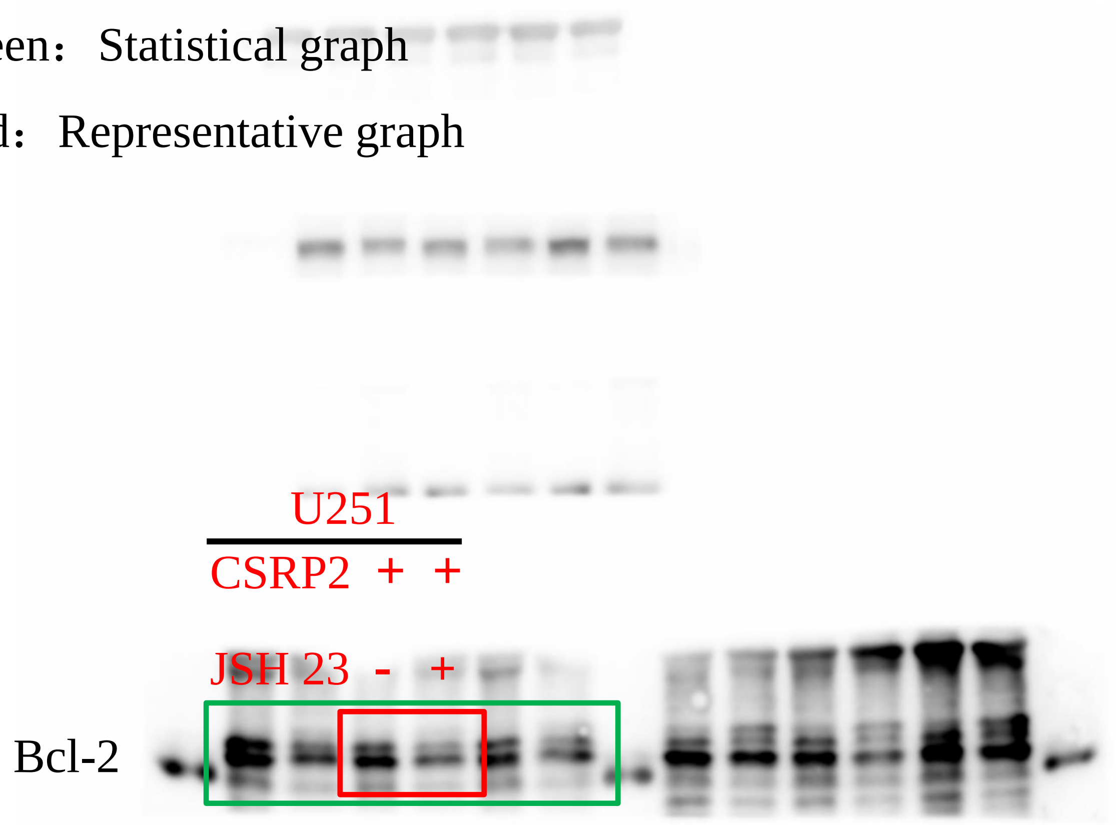

The membrane was imaged with Azure Biosystems 300

Full unedited gel for Figure 5K

Green： Statistical graph

Red： Representative graph

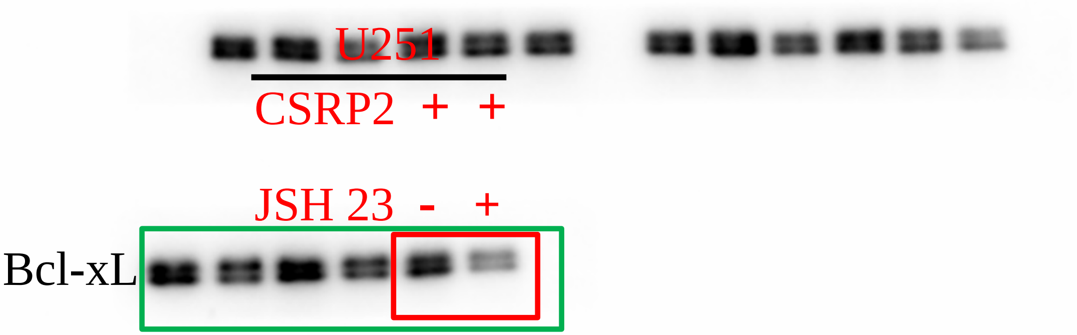

The membrane was imaged with Azure Biosystems 300

Full unedited gel for Figure 5K

Green： Statistical graph

Red： Representative graph

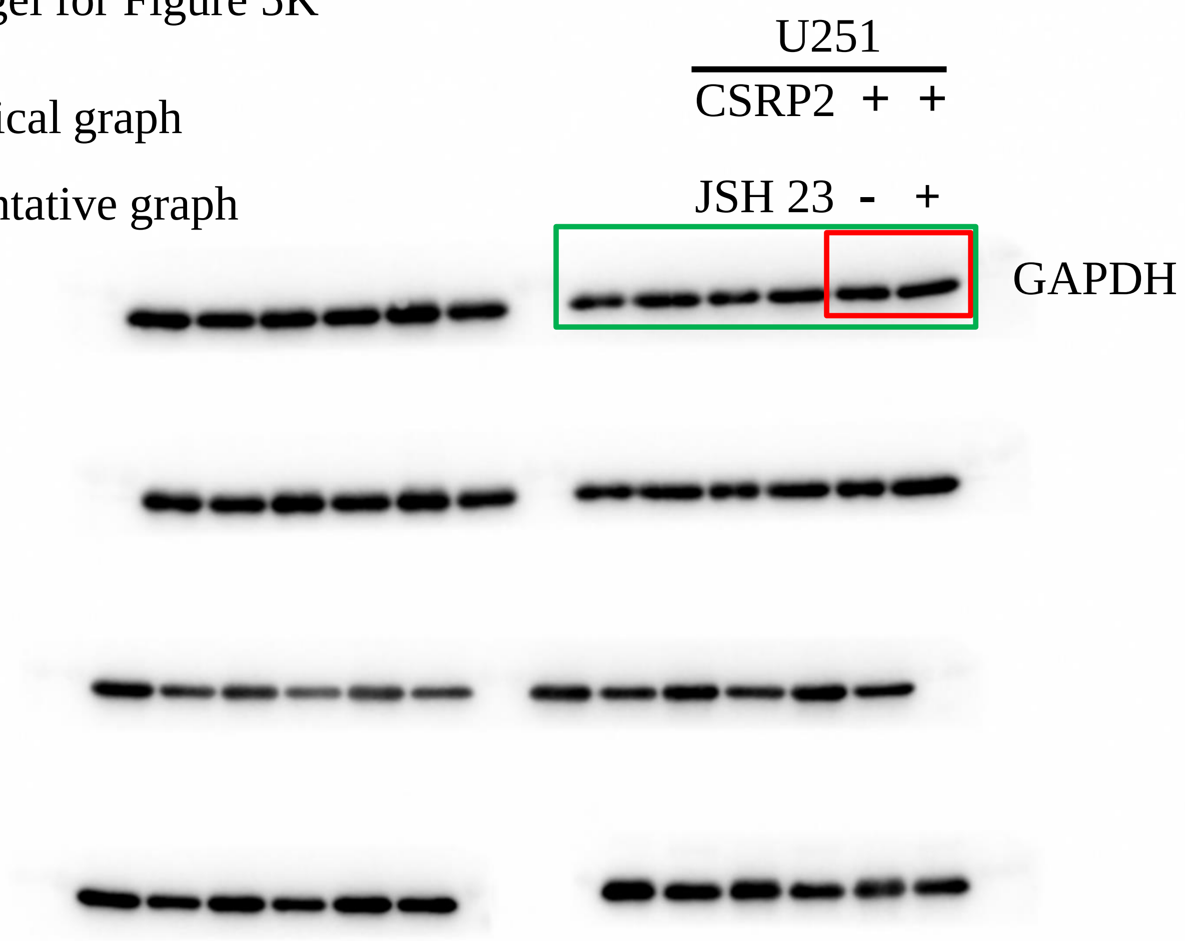

The membrane was imaged with Azure Biosystems 300

Full unedited gel for Figure 6B

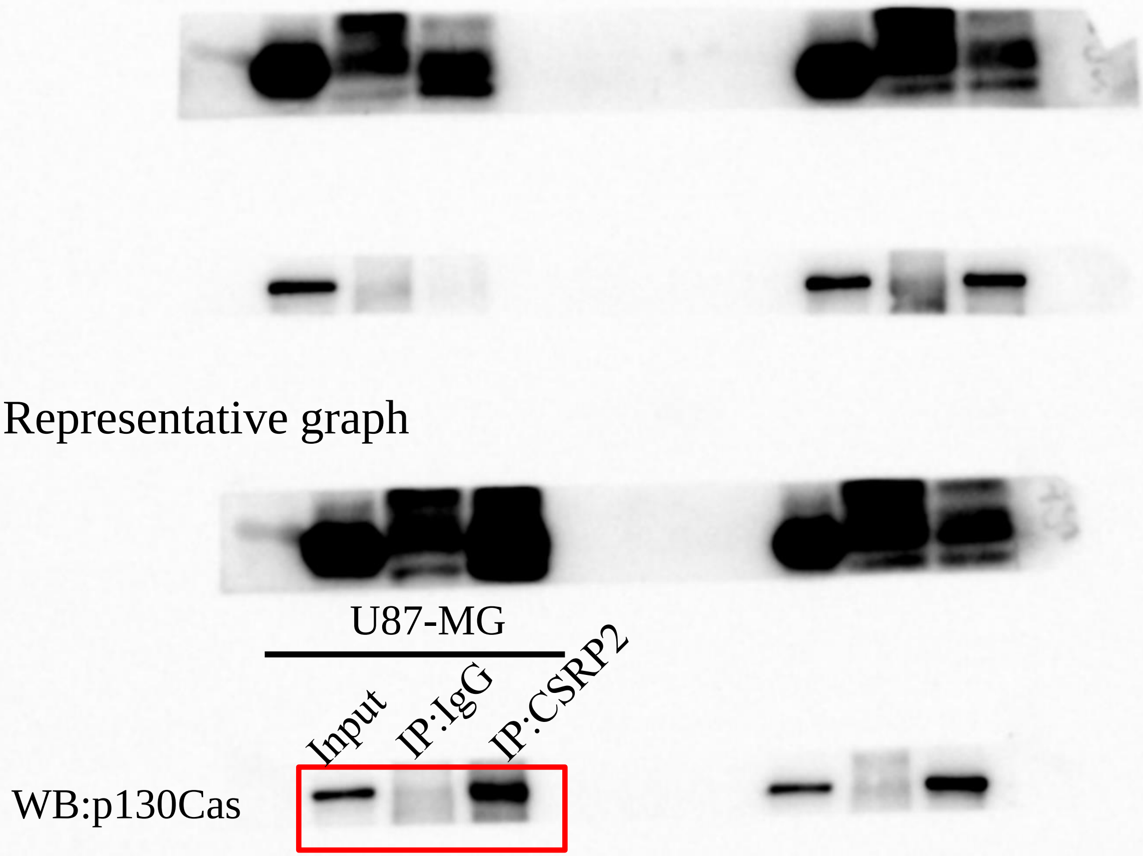

Red： Representative graph

The membrane was imaged with Azure Biosystems 300

Full unedited gel for Figure 6B

Red： Representative graph

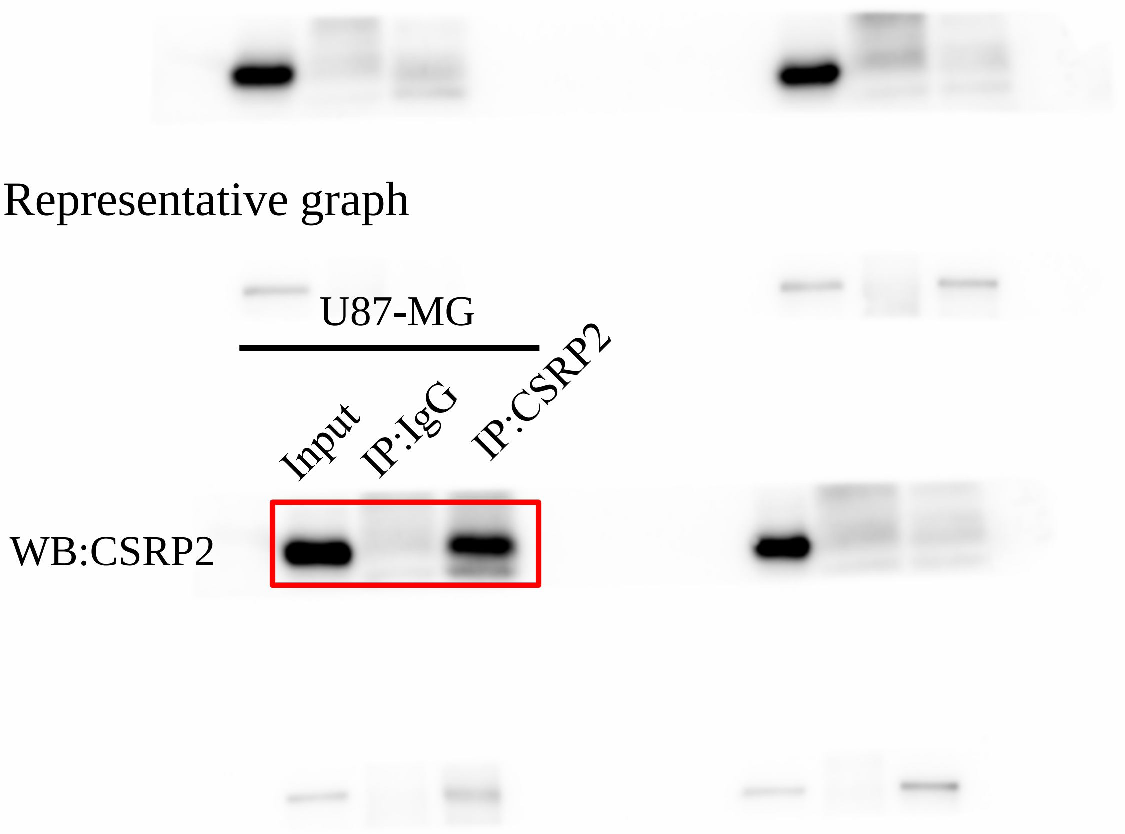

The membrane was imaged with Azure Biosystems 300

Full unedited gel for Figure 6B

Red: Representative graph

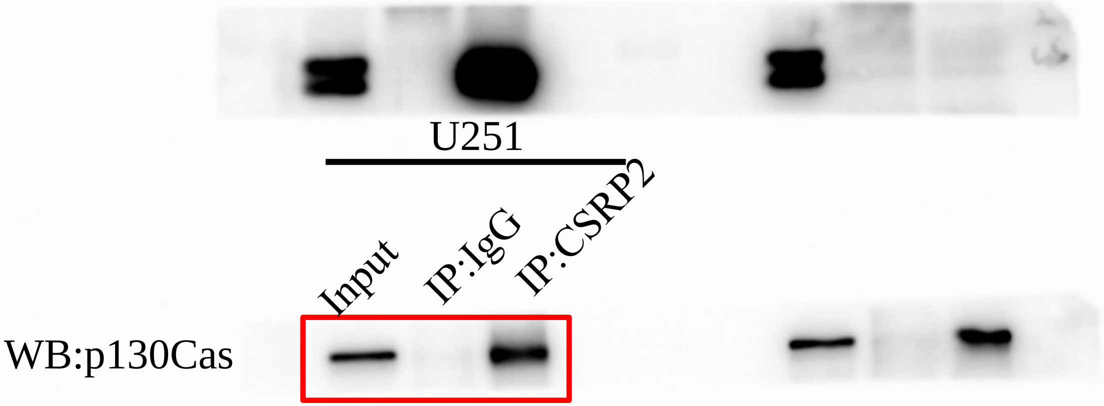

The membrane was imaged with Azure Biosystems 300

Full unedited gel for Figure 6B

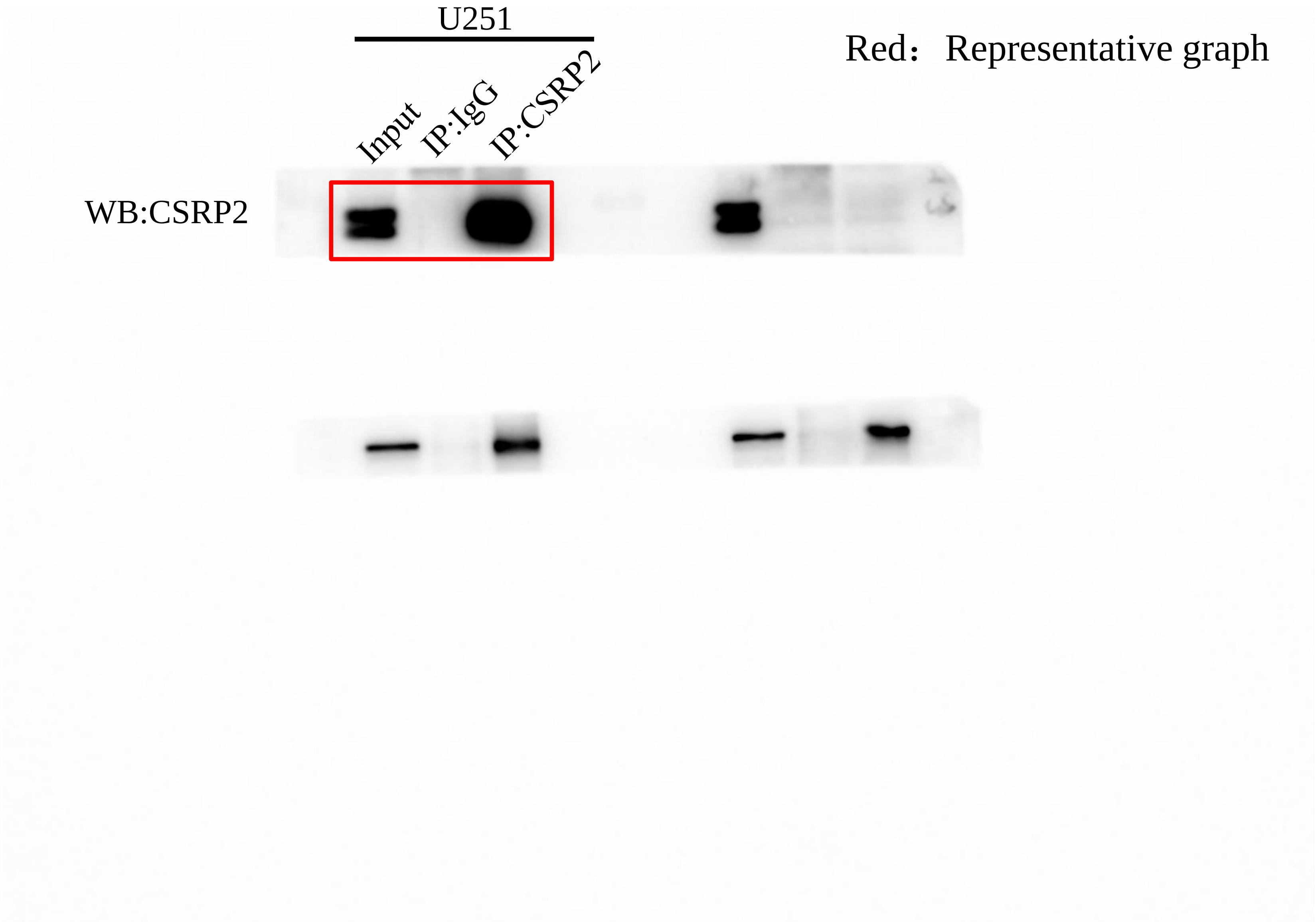

The membrane was imaged with Azure Biosystems 300

Full unedited gel for Figure 6C

Red： Representative graph

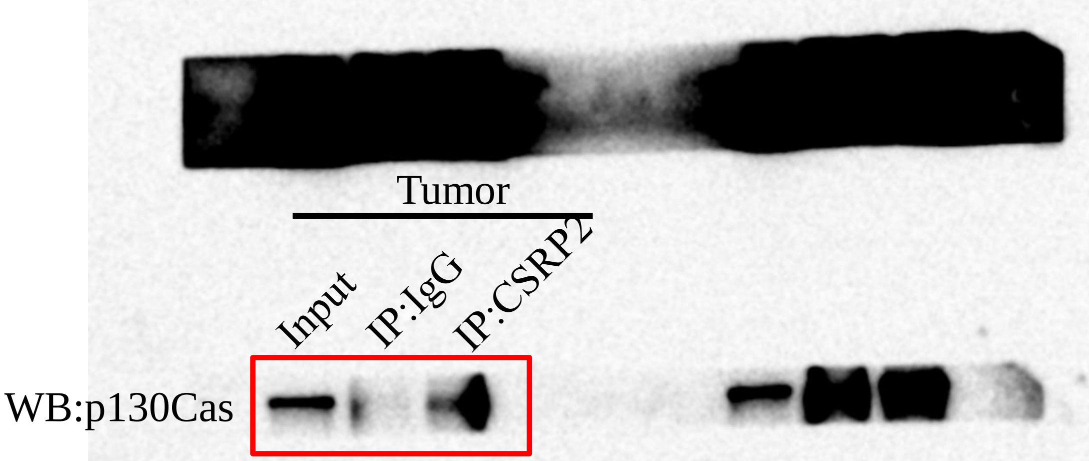

The membrane was imaged with Azure Biosystems 300

Full unedited gel for Figure 6C

Red： Representative graph

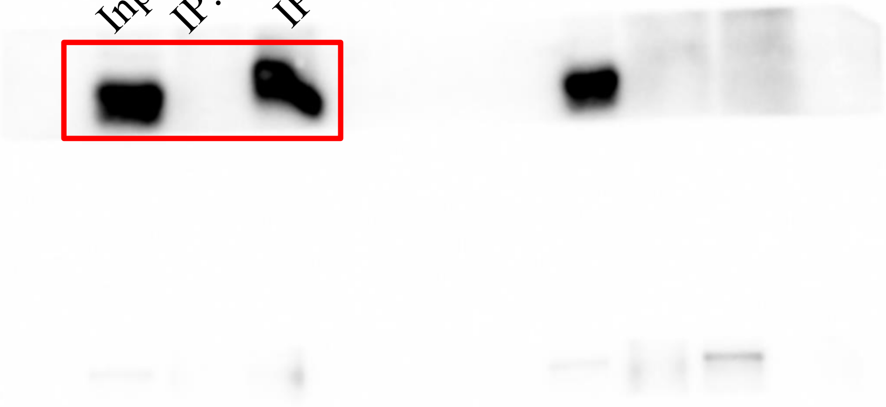

The membrane was imaged with Azure Biosystems 300

Full unedited gel for Figure 6D

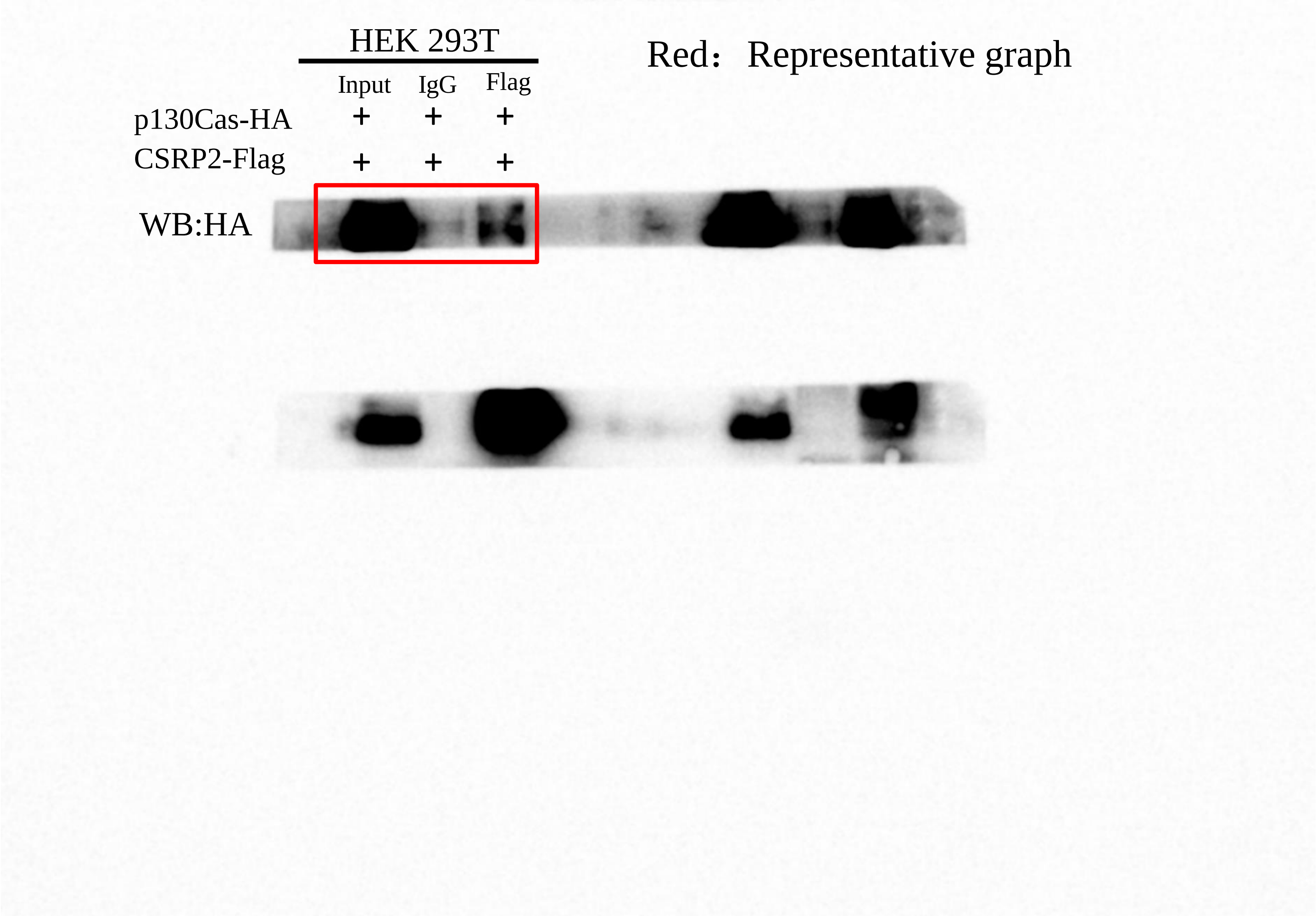

The membrane was imaged with Azure Biosystems 300

Full unedited gel for Figure 6D

Red： Representative graph

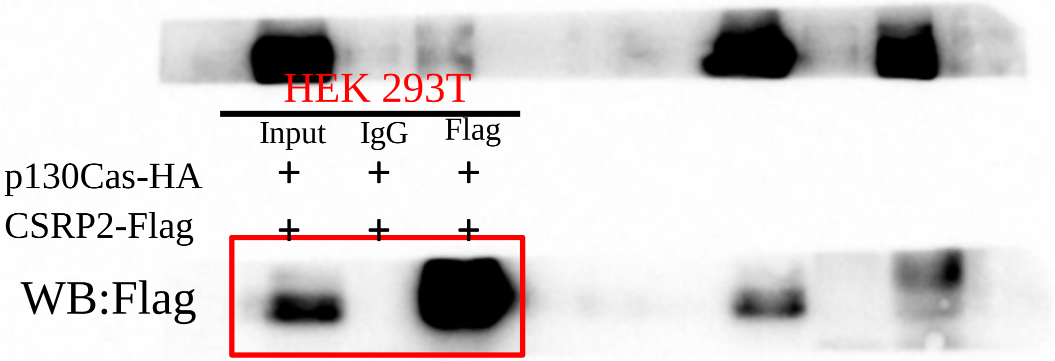

The membrane was imaged with Azure Biosystems 300

Full unedited gel for Figure 6D

Red： Representative graph

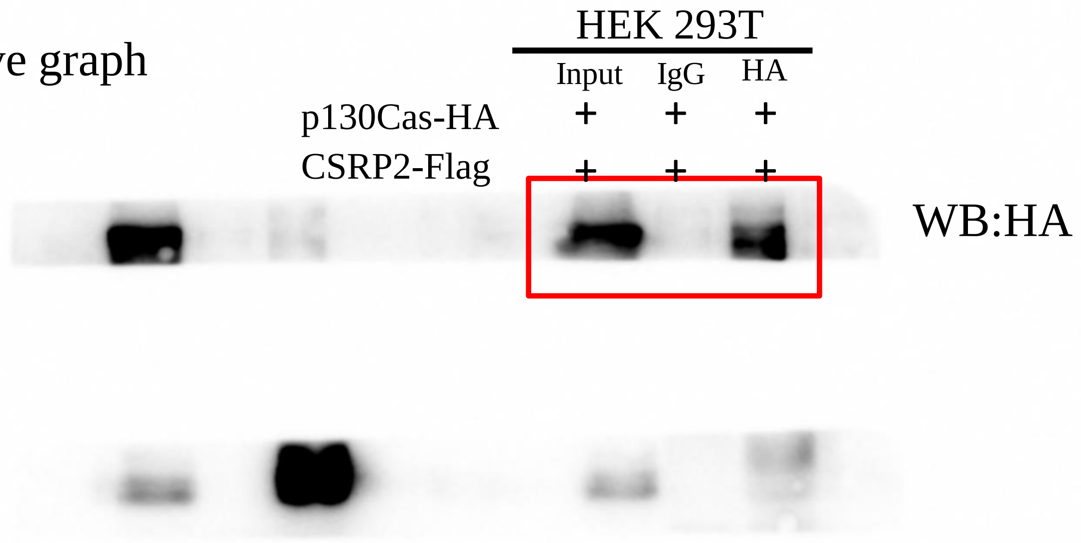

The membrane was imaged with Azure Biosystems 300

Full unedited gel for Figure 6D

Red： Representative graph

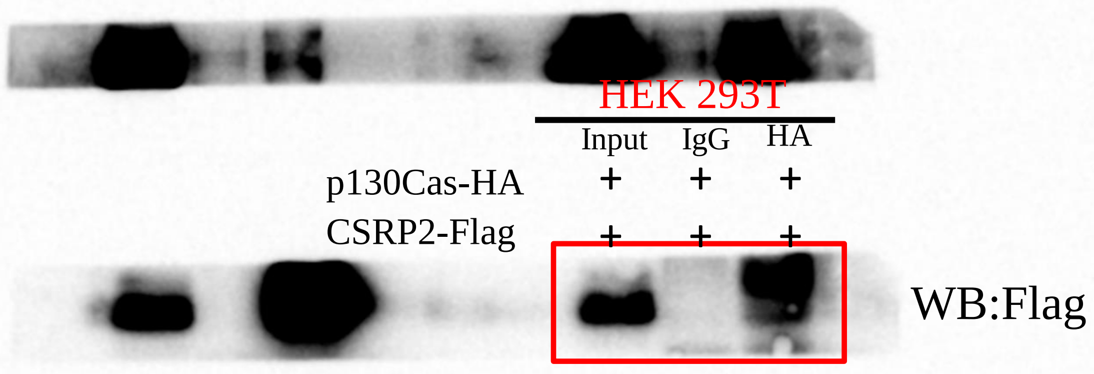

The membrane was imaged with Azure Biosystems 300

Full unedited gel for Figure 6E

Red： Representative graph

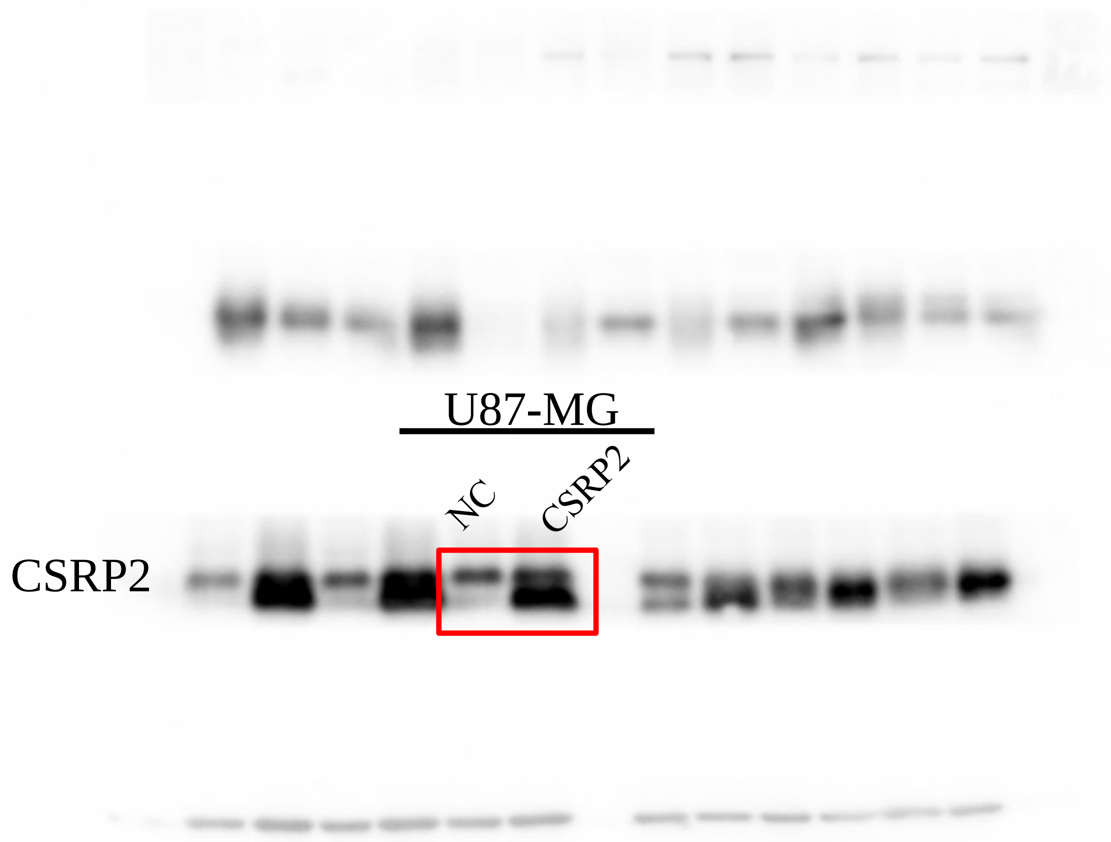

The membrane was imaged with Azure Biosystems 300

Full unedited gel for Figure 6E

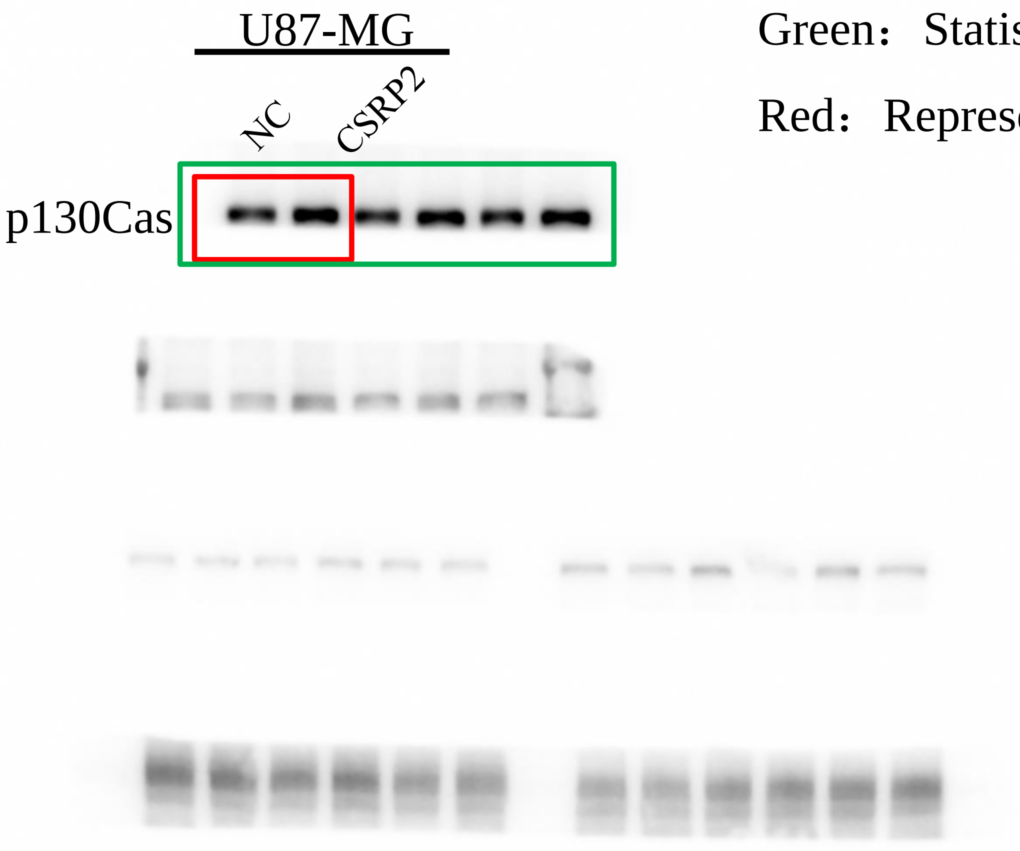

Green： Statistical graph

Red： Representative graph

The membrane was imaged with Azure Biosystems 300

Full unedited gel for Figure 6E

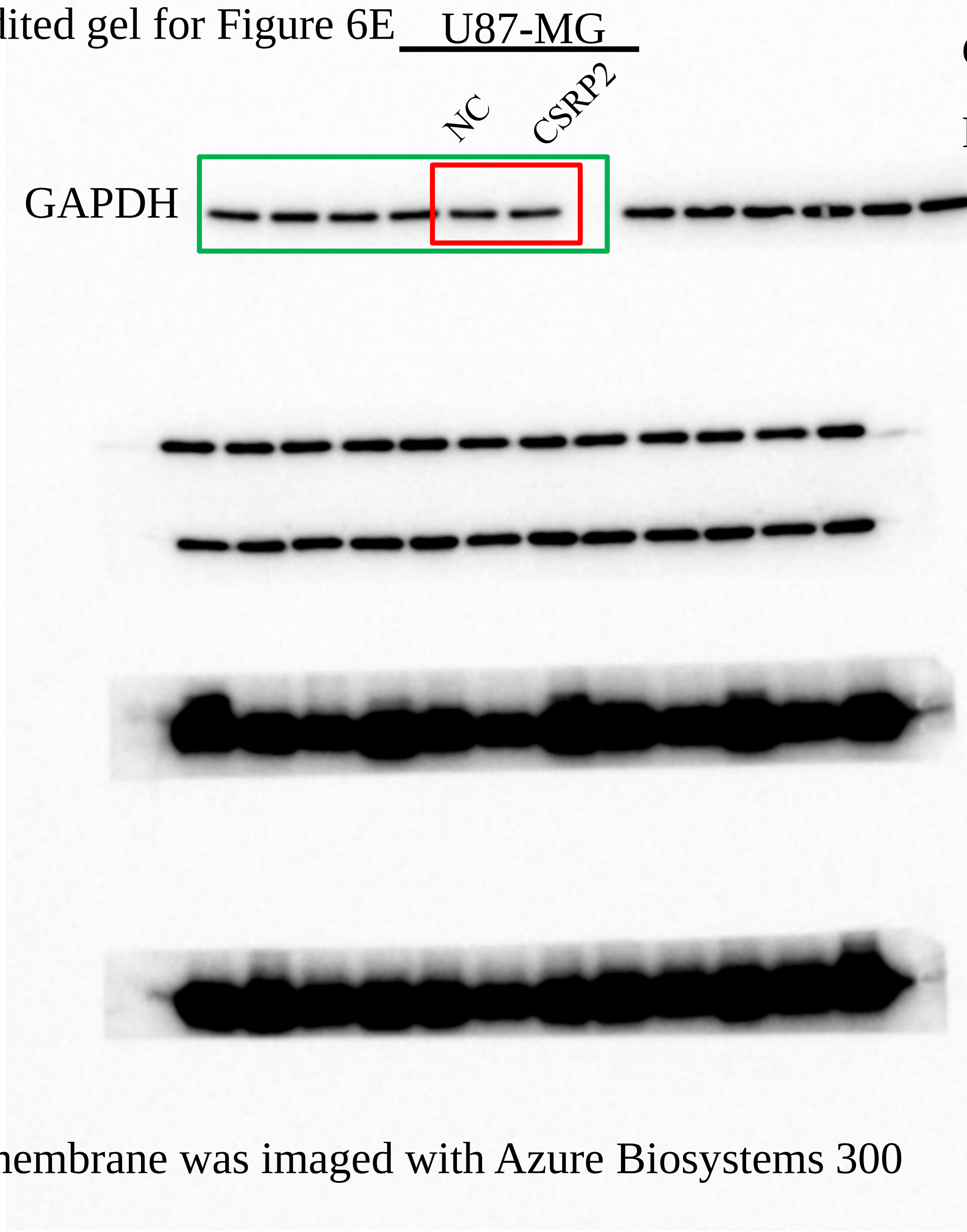

Green： Statistical graph

Red： Representative graph

The membrane was imaged with Azure Biosystems 300

Full unedited gel for Figure 6E

Red： Representative graph

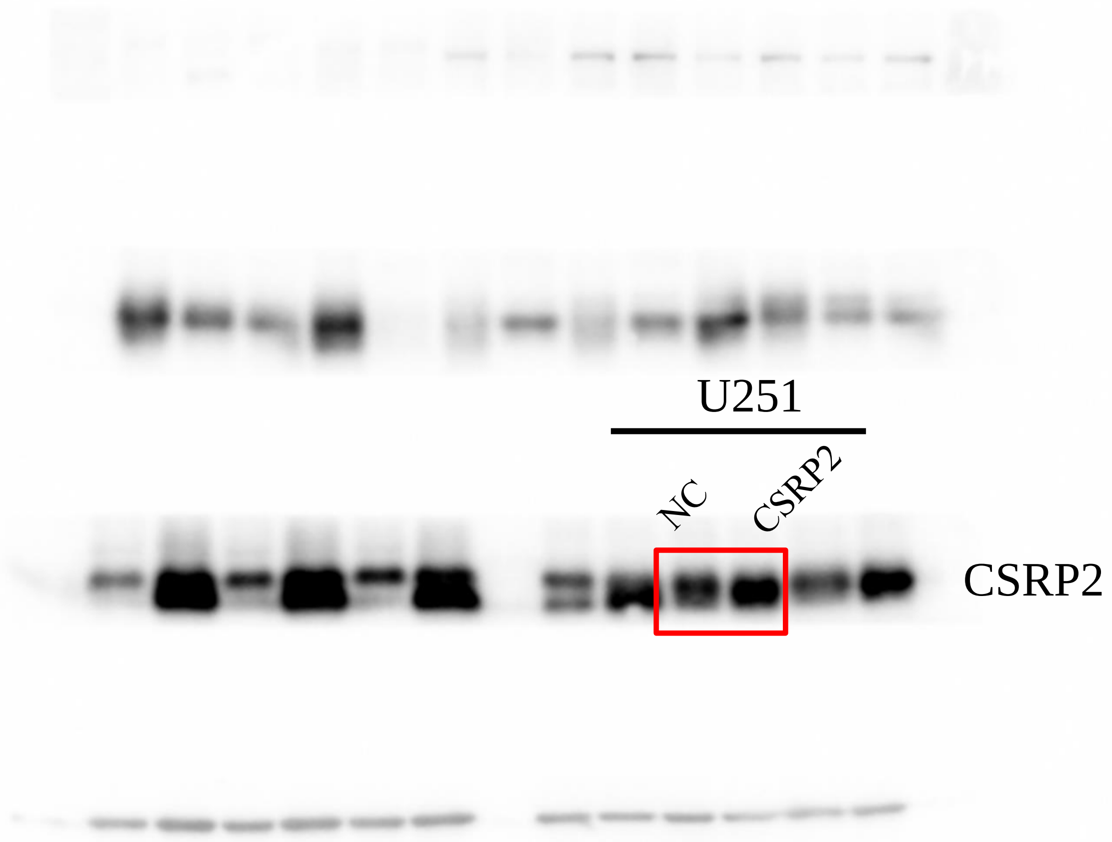

The membrane was imaged with Azure Biosystems 300

Full unedited gel for Figure 6E

Green： Statistical graph

Red： Representative graph

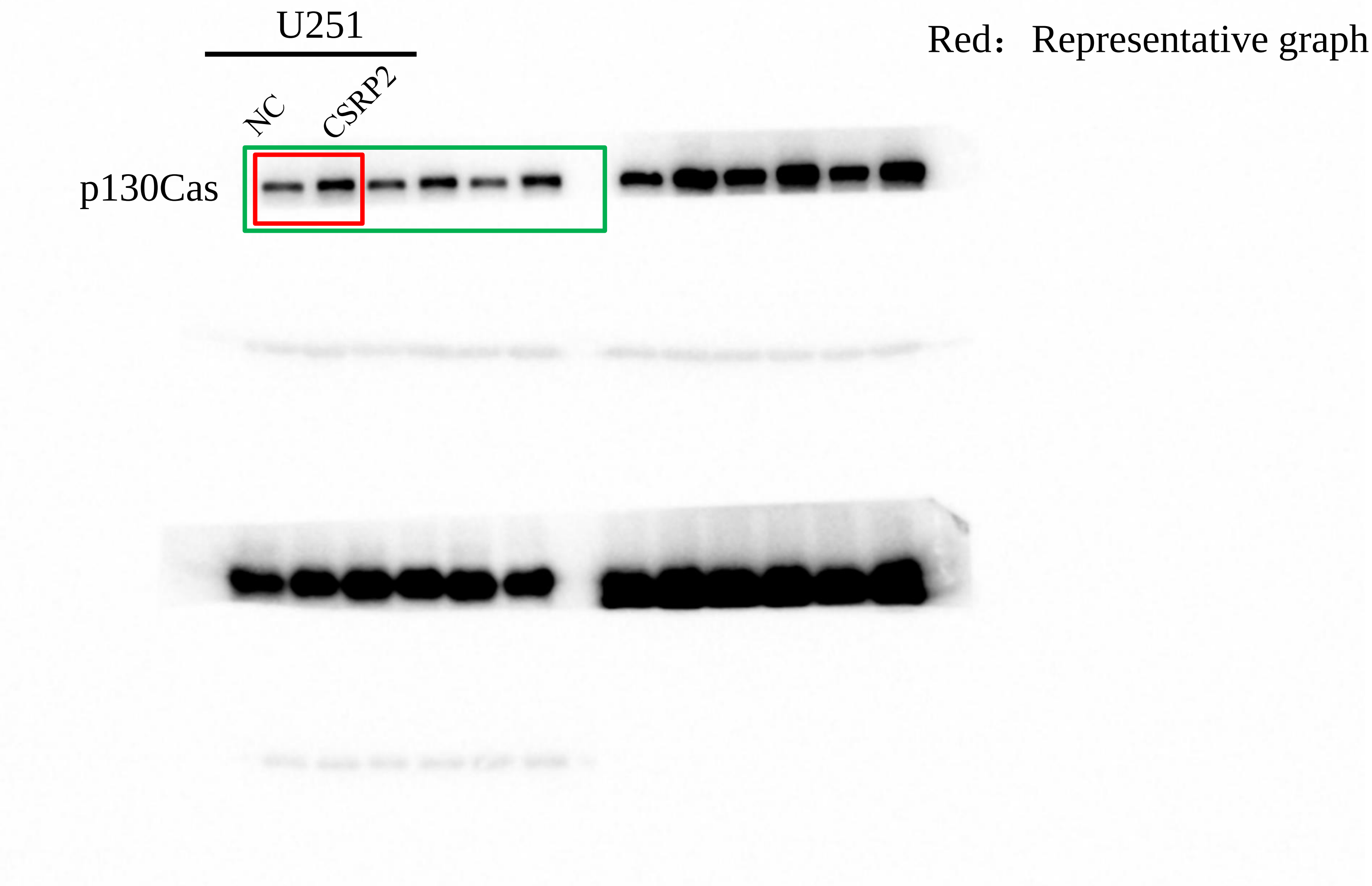

The membrane was imaged with Azure Biosystems 300

Full unedited gel for Figure 6E

Green： Statistical graph

Red： Representative graph

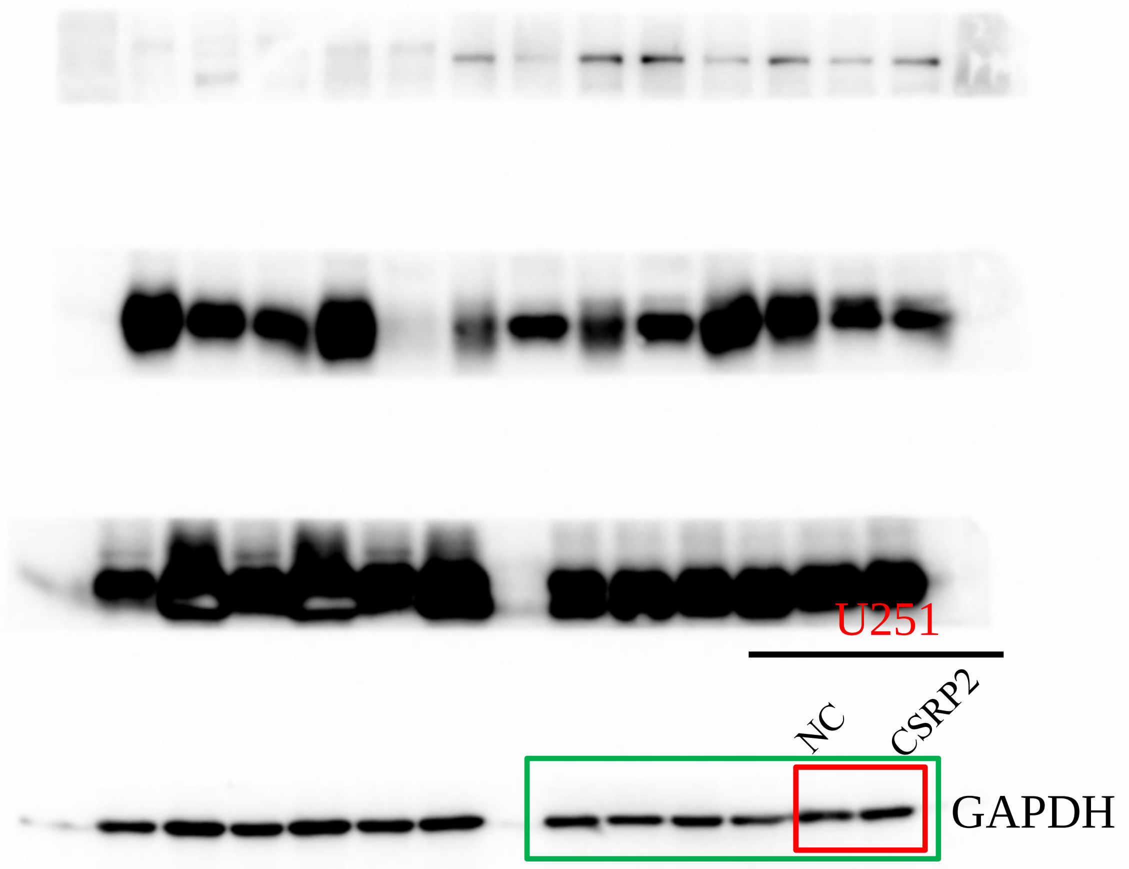

The membrane was imaged with Azure Biosystems 300

Full unedited gel for Figure 6G

Red： Representative graph

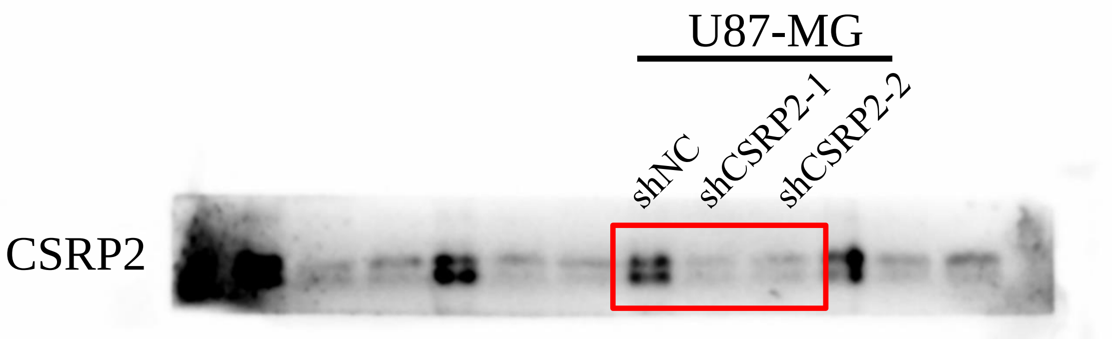

The membrane was imaged with Azure Biosystems 300

Full unedited gel for Figure 6G

Green： Statistical graph

Red： Representative graph

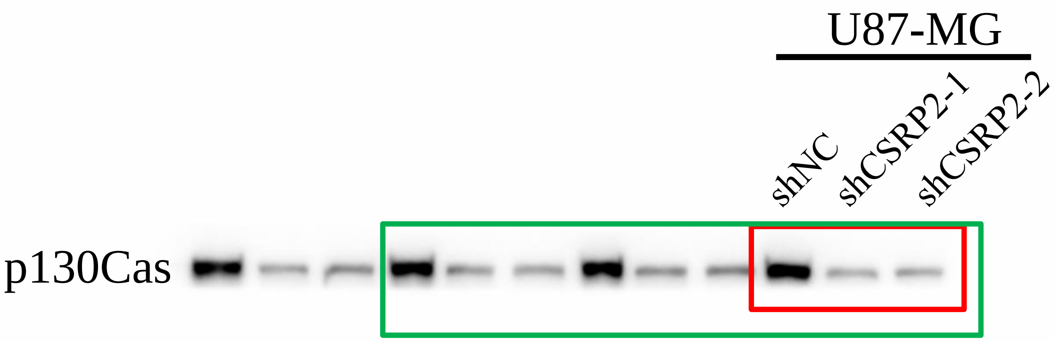

The membrane was imaged with Azure Biosystems 300

Full unedited gel for Figure 6G

Green： Statistical graph

Red： Representative graph

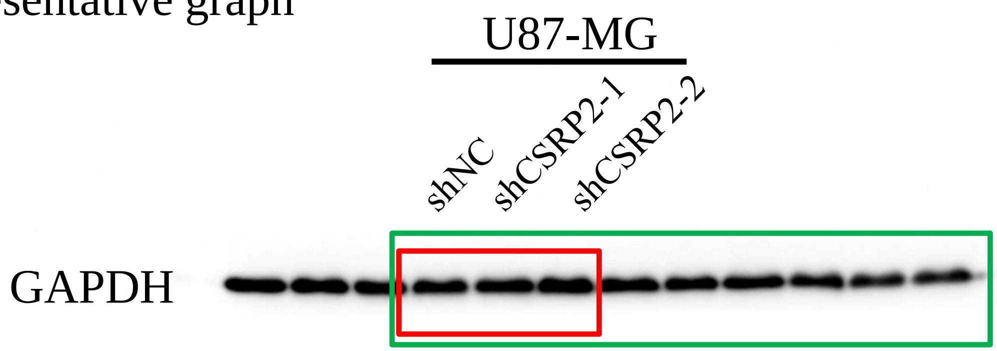

The membrane was imaged with Azure Biosystems 300

Full unedited gel for Figure 6G

Red： Representative graph

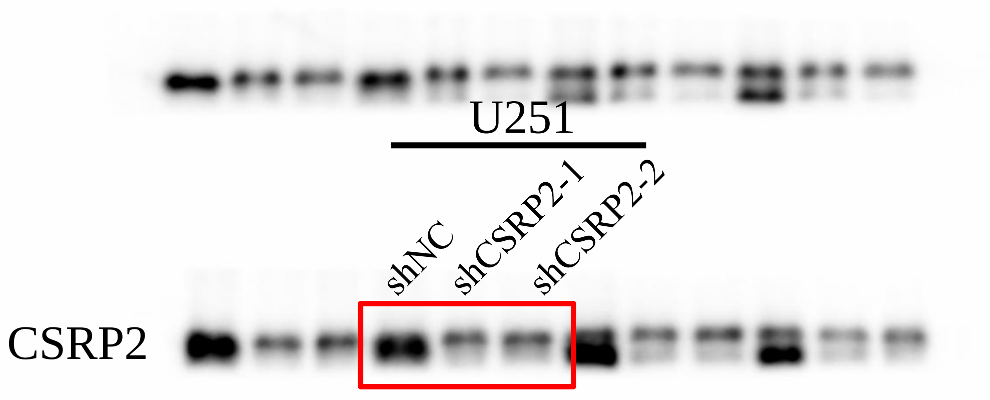

The membrane was imaged with Azure Biosystems 300

Full unedited gel for Figure 6G

Green： Statistical graph

Red： Representative graph

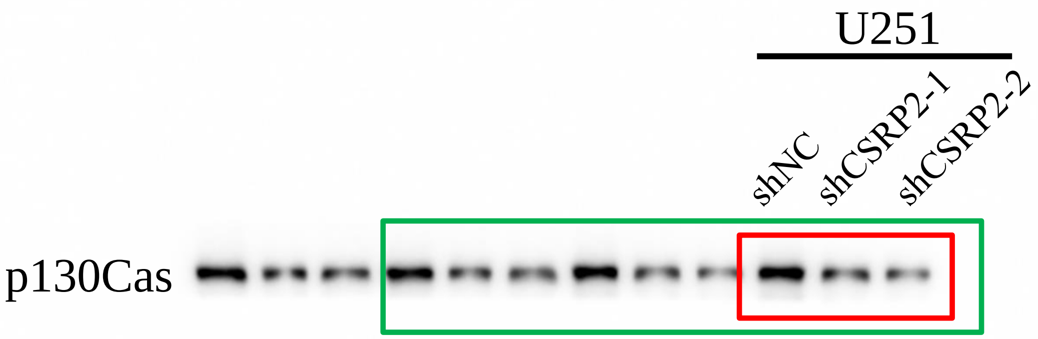

The membrane was imaged with Azure Biosystems 300

Full unedited gel for Figure 6G

Green: Statistical graph

Red: Representative graph

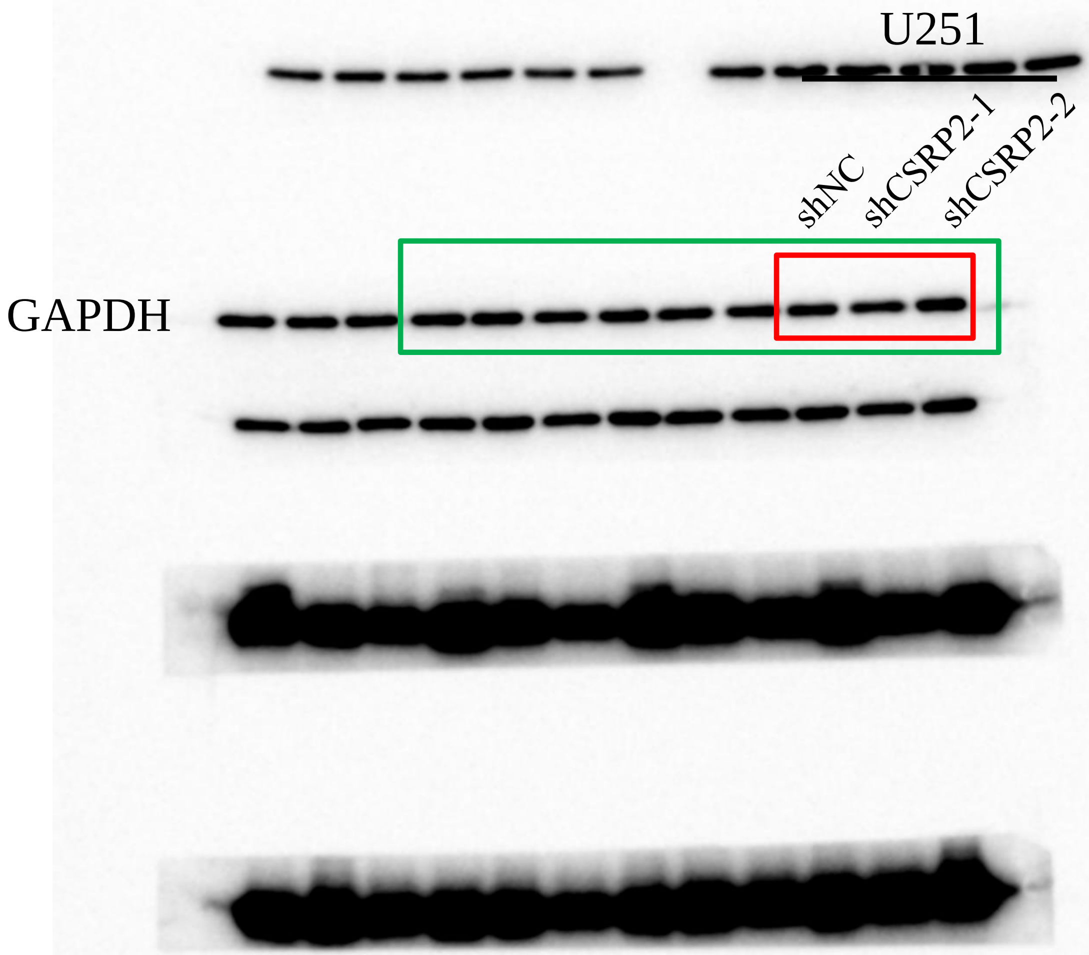

The membrane was imaged with Azure Biosystems 300

Full unedited gel for Figure 6I

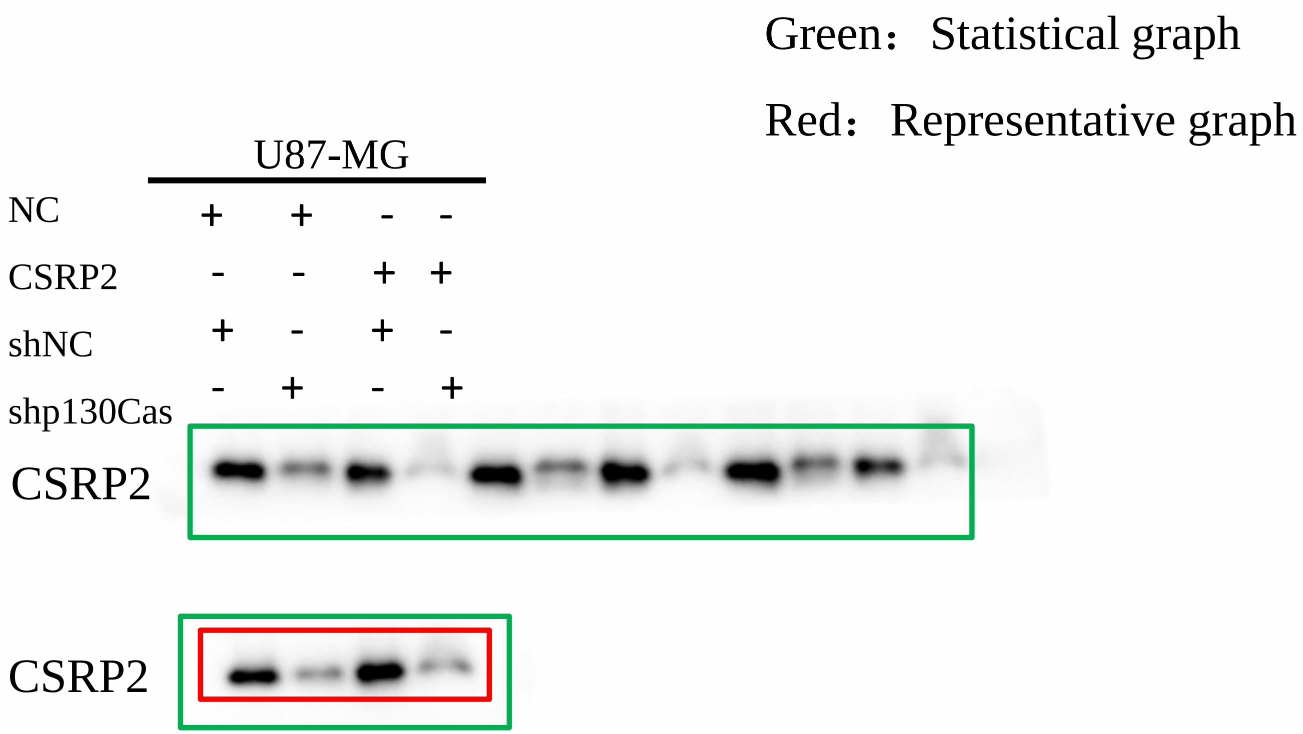

The membrane was imaged with Azure Biosystems 300

Full unedited gel for Figure 6I

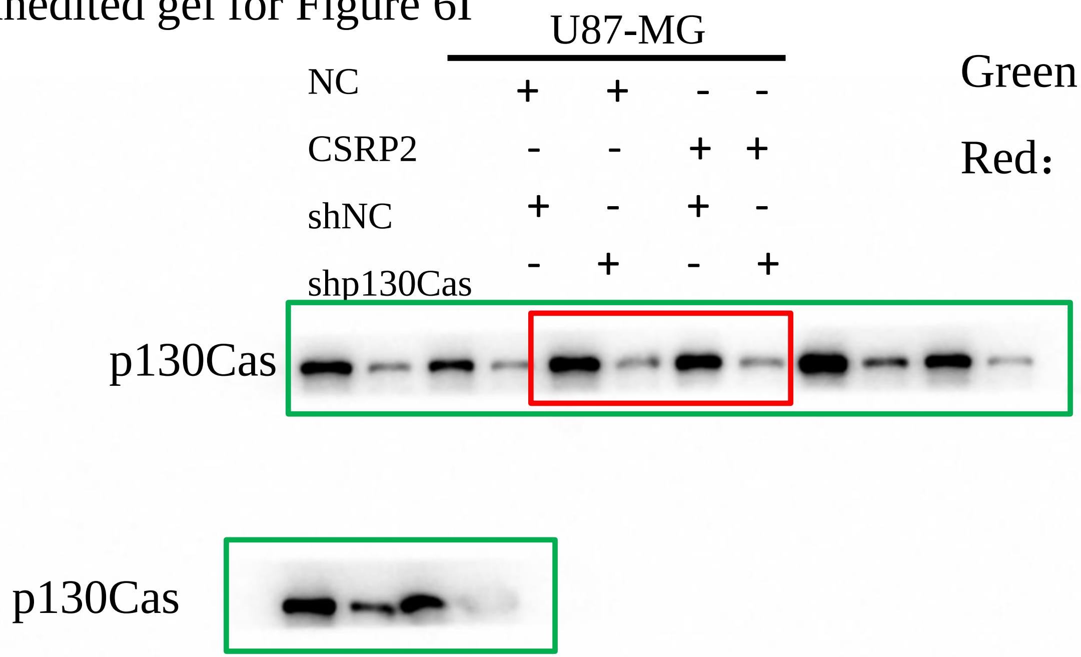

Green： Statistical graph

Red： Representative graph

The membrane was imaged with Azure Biosystems 300

Full unedited gel for Figure 6I

U87-MG

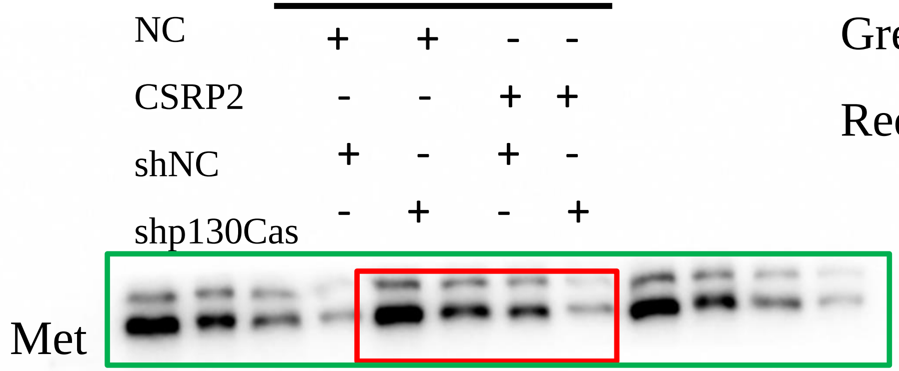

Green： Statistical graph

Red： Representative graph

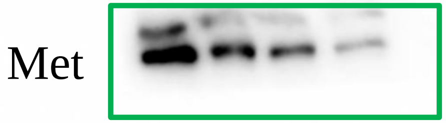

The membrane was imaged with Azure Biosystems 300

Full unedited gel for Figure 6I

Green： Statistical graph

Red： Representative graph

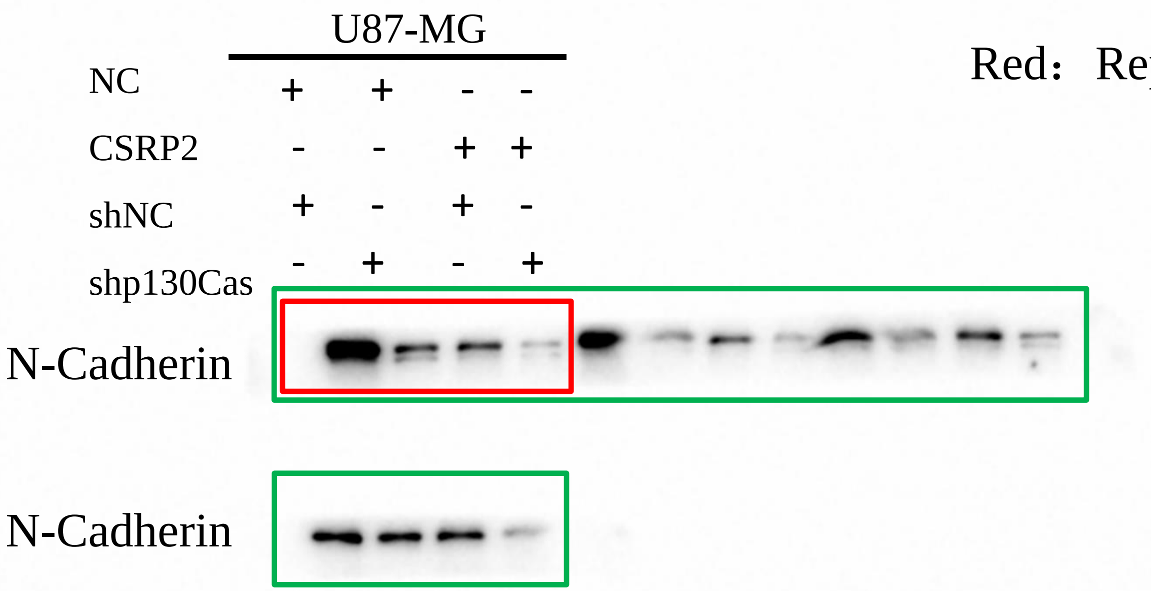

The membrane was imaged with Azure Biosystems 300

Full unedited gel for Figure 6I

Green： Statistical graph

Red： Representative graph

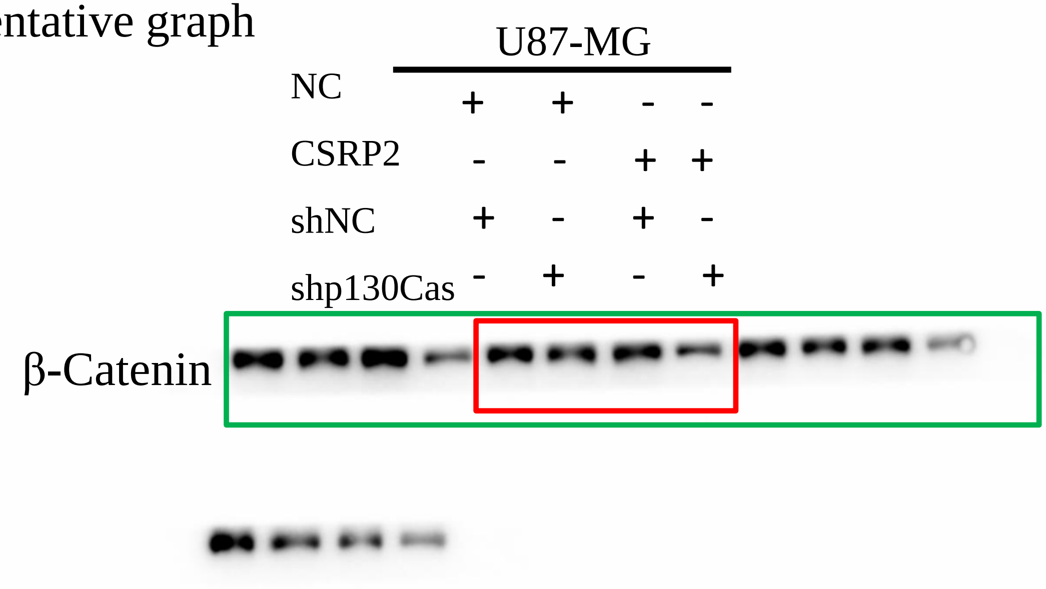

The membrane was imaged with Azure Biosystems 300

Full unedited gel for Figure 6I

Green： Statistical graph

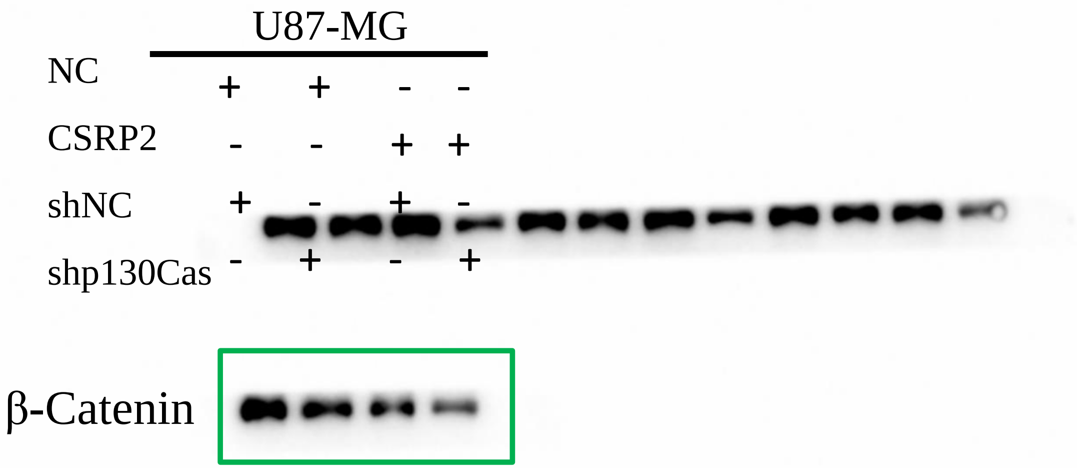

The membrane was imaged with Azure Biosystems 300

Full unedited gel for Figure 6I

Green： Statistical graph

Red： Representative graph

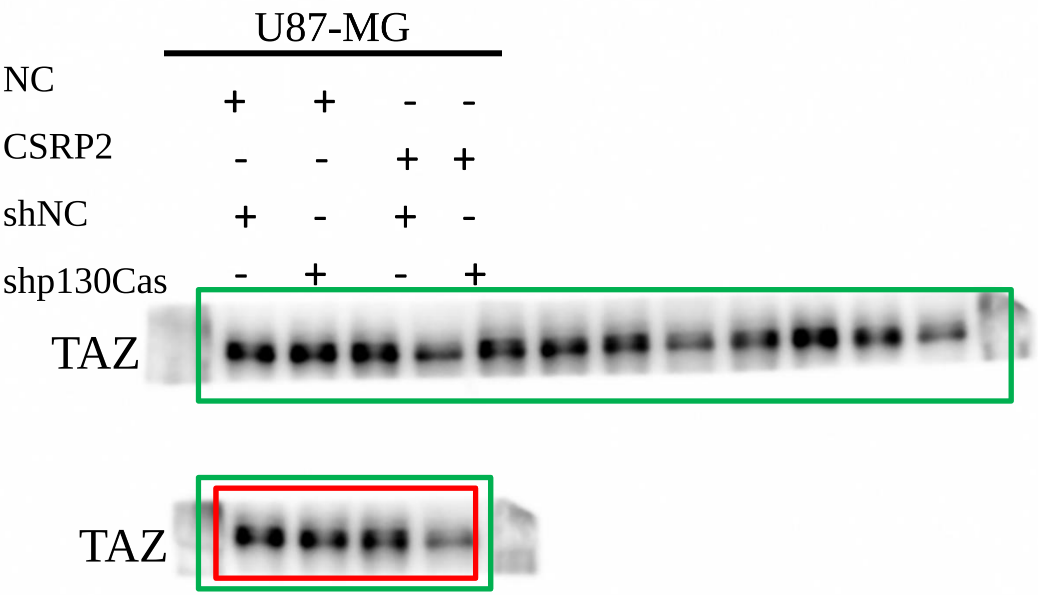

The membrane was imaged with Azure Biosystems 300

Full unedited gel for Figure 6I

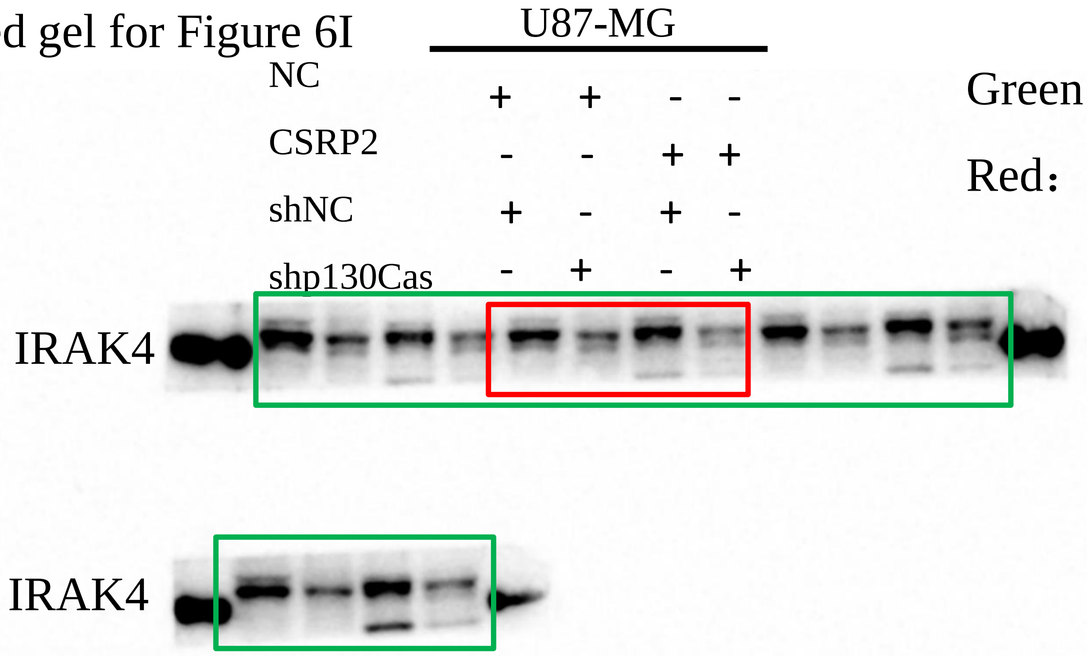

Green： Statistical graph

Red： Representative graph

The membrane was imaged with Azure Biosystems 300

Full unedited gel for Figure 6I

Green： Statistical graph

Red： Representative graph

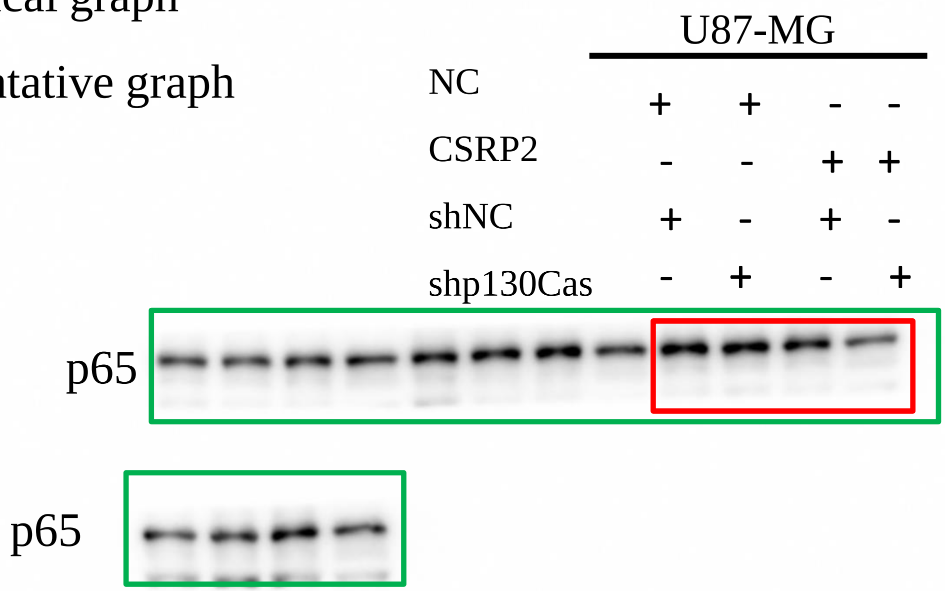

The membrane was imaged with Azure Biosystems 300

Full unedited gel for Figure 6I

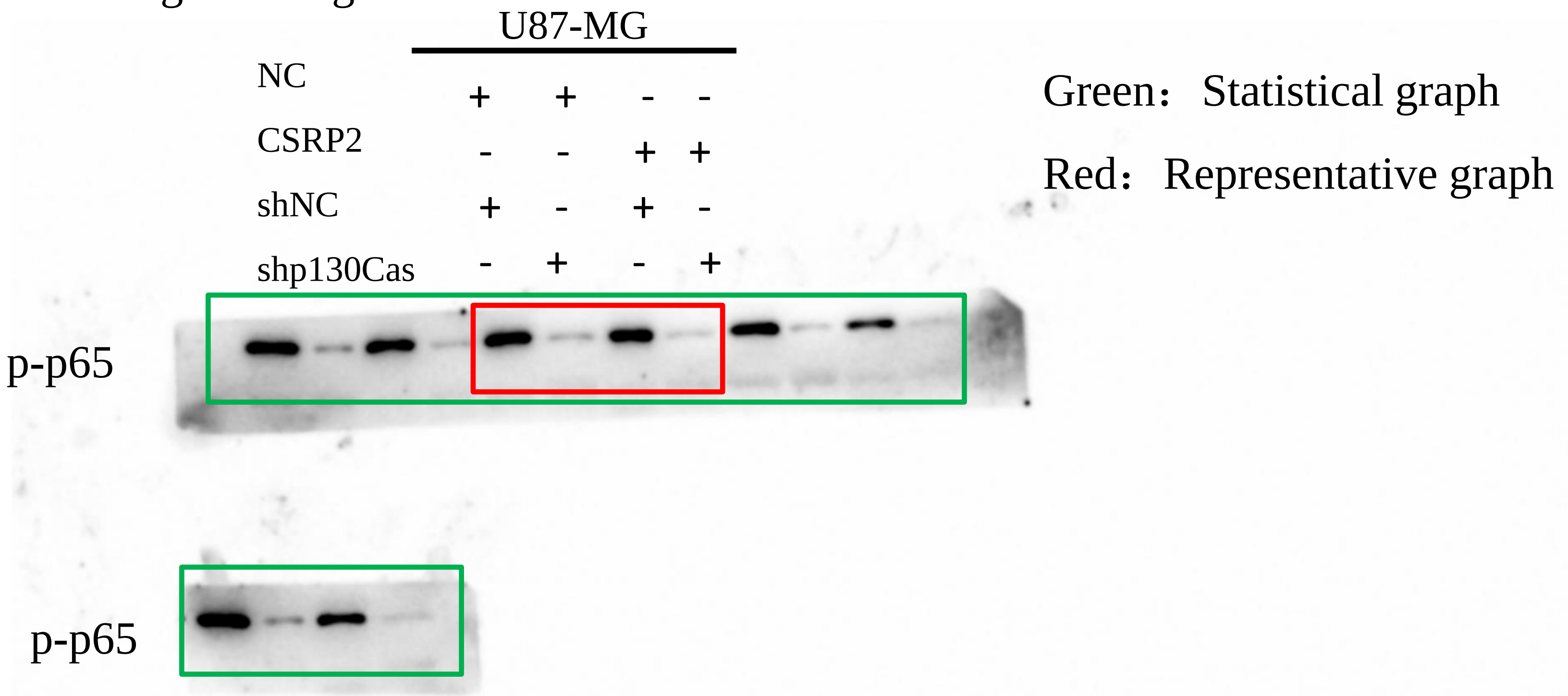

The membrane was imaged with Azure Biosystems 300

Full unedited gel for Figure 6I

Green: Statistical graph

Red: Representative graph

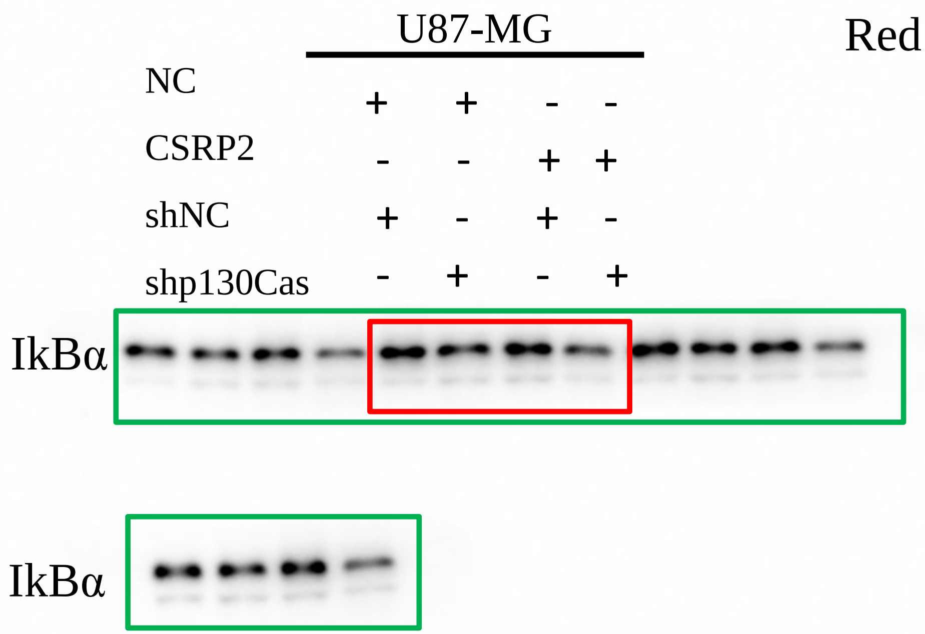

The membrane was imaged with Azure Biosystems 300

Full unedited gel for Figure 6I

Green： Statistical graph

Red： Representative graph

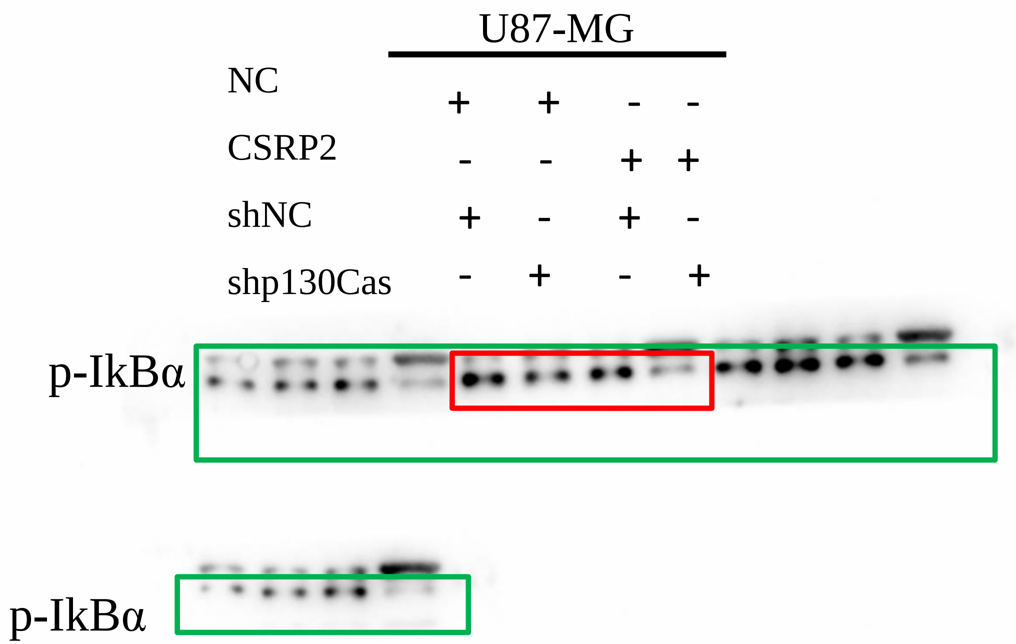

The membrane was imaged with Azure Biosystems 300

Full unedited gel for Figure 6I

Green: Statistical graph

Red: Representative graph

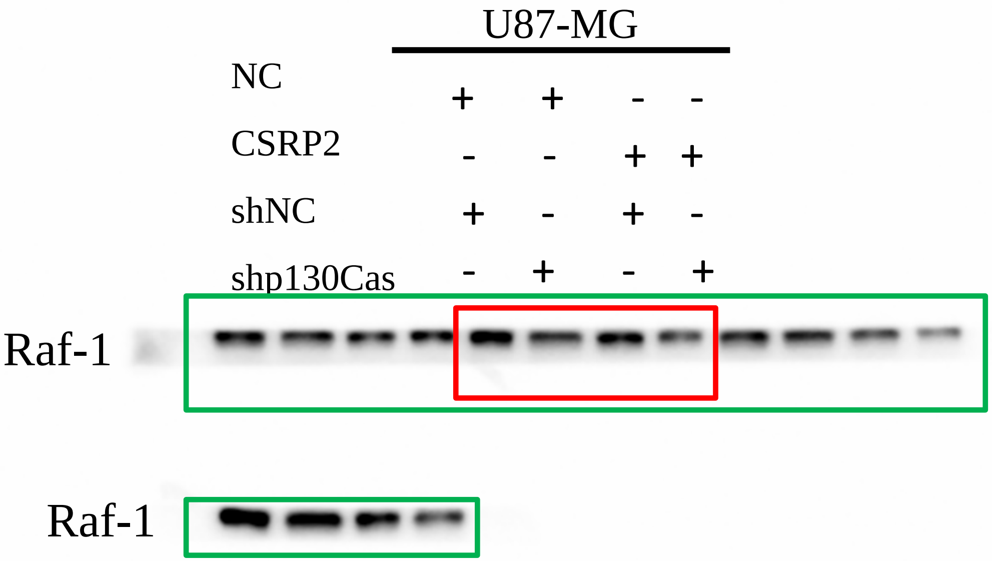

The membrane was imaged with Azure Biosystems 300

Full unedited gel for Figure 6I

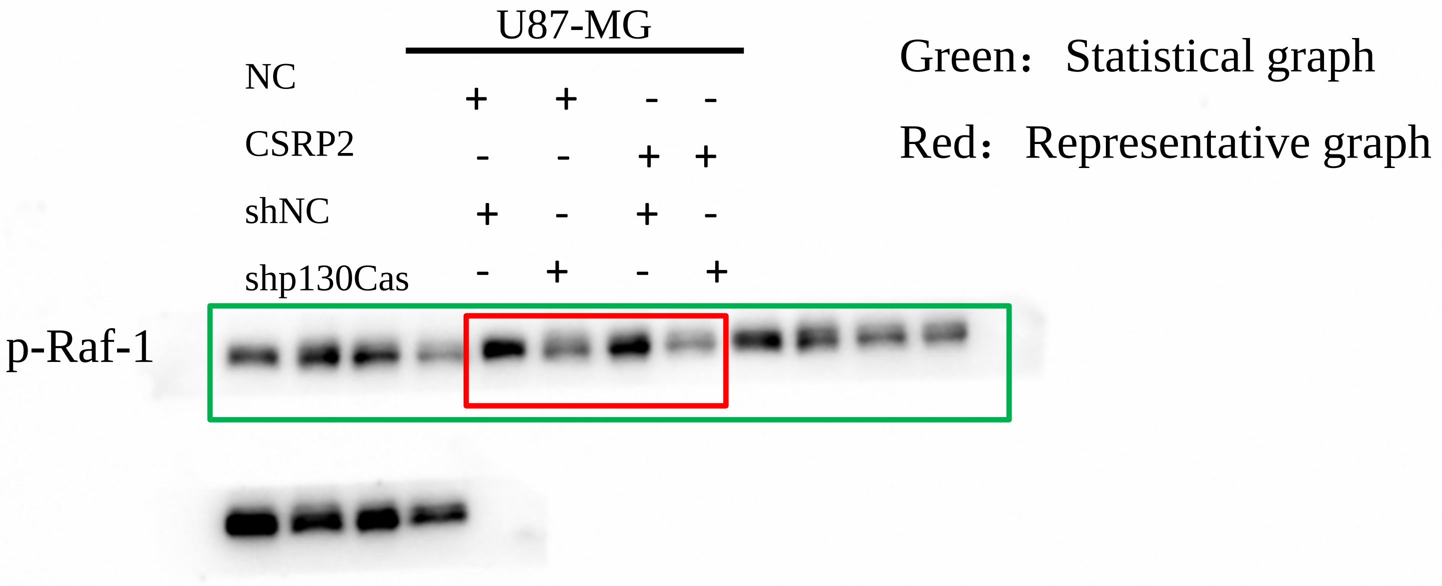

The membrane was imaged with Azure Biosystems 300

Full unedited gel for Figure 6I

Green： Statistical graph

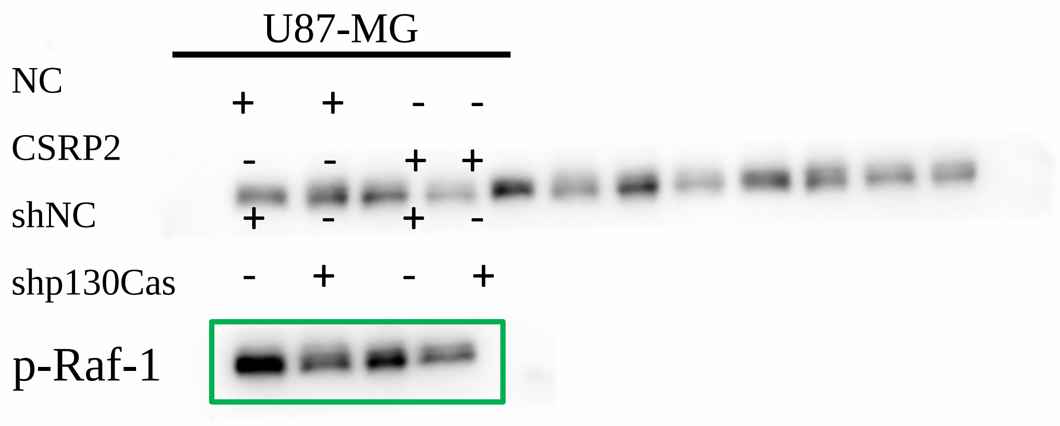

The membrane was imaged with Azure Biosystems 300

Full unedited gel for Figure 6I

Green： Statistical graph

Red： Representative graph

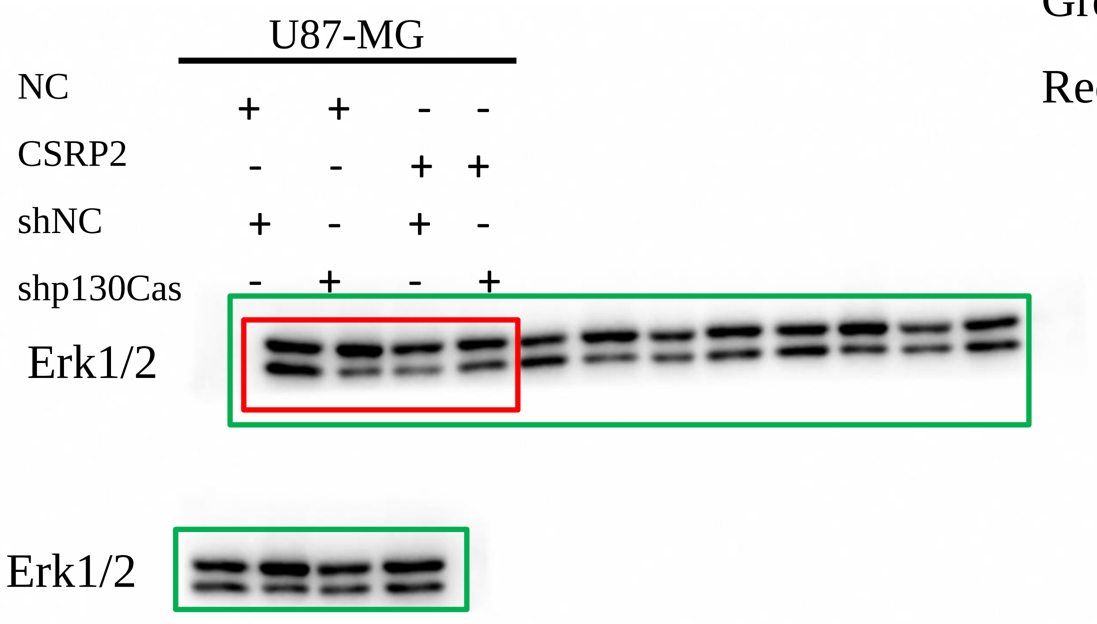

The membrane was imaged with Azure Biosystems 300

Full unedited gel for Figure 6I

Green： Statistical graph

Red： Representative graph

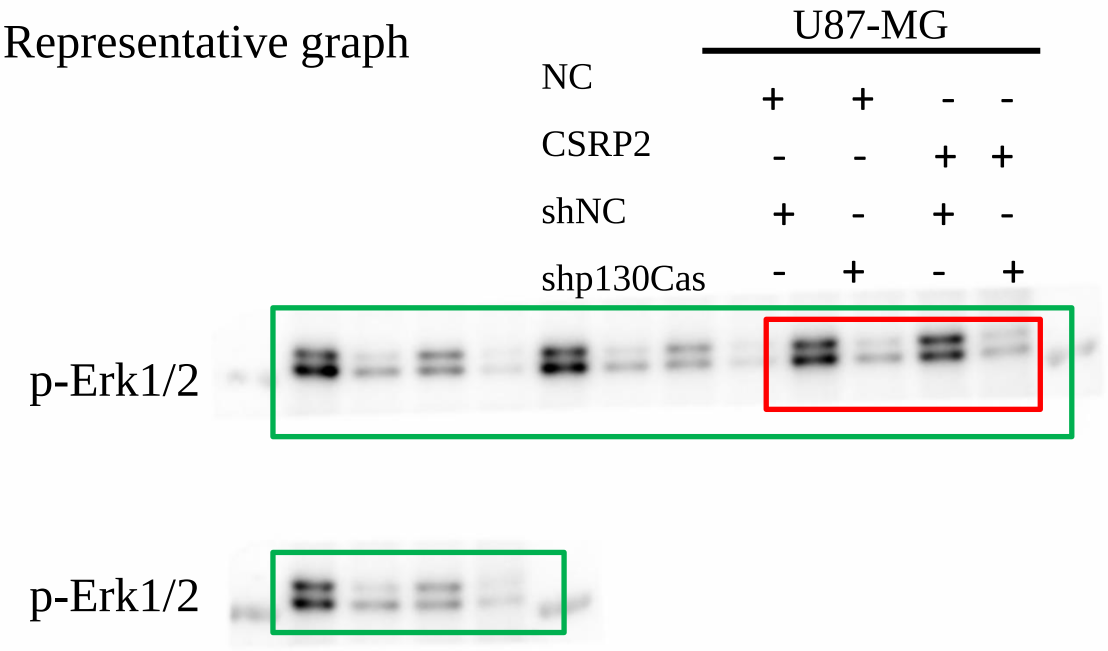

The membrane was imaged with Azure Biosystems 300

Full unedited gel for Figure 6I

Green： Statistical graph

Red： Representative graph

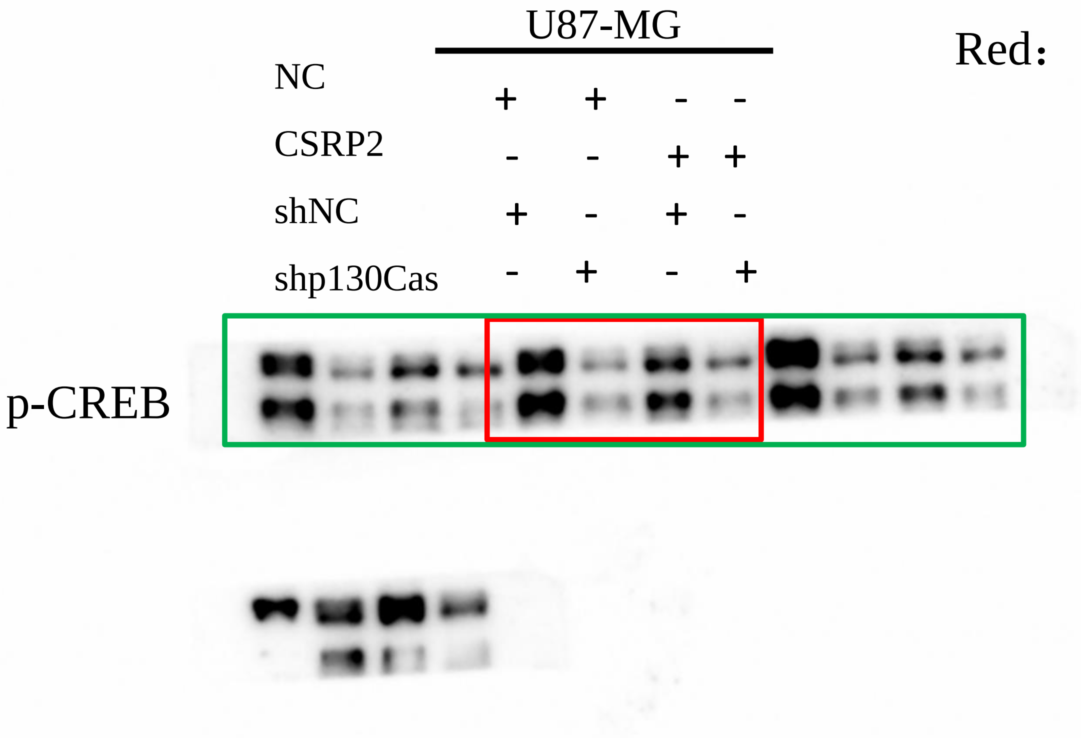

The membrane was imaged with Azure Biosystems 300

Full unedited gel for Figure 6I

Green： Statistical graph

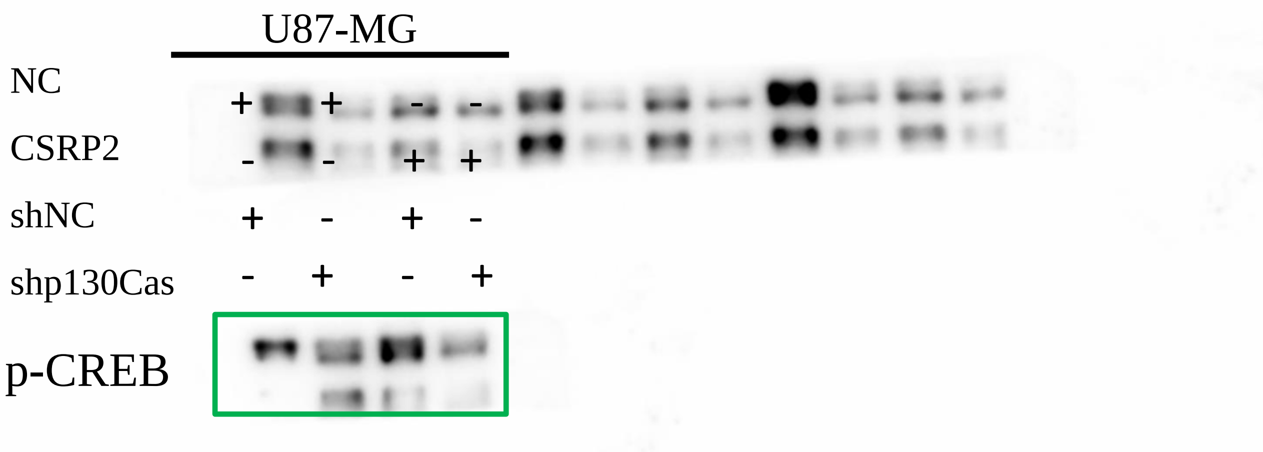

The membrane was imaged with Azure Biosystems 300

Full unedited gel for Figure 6I

Green：Statistical graph

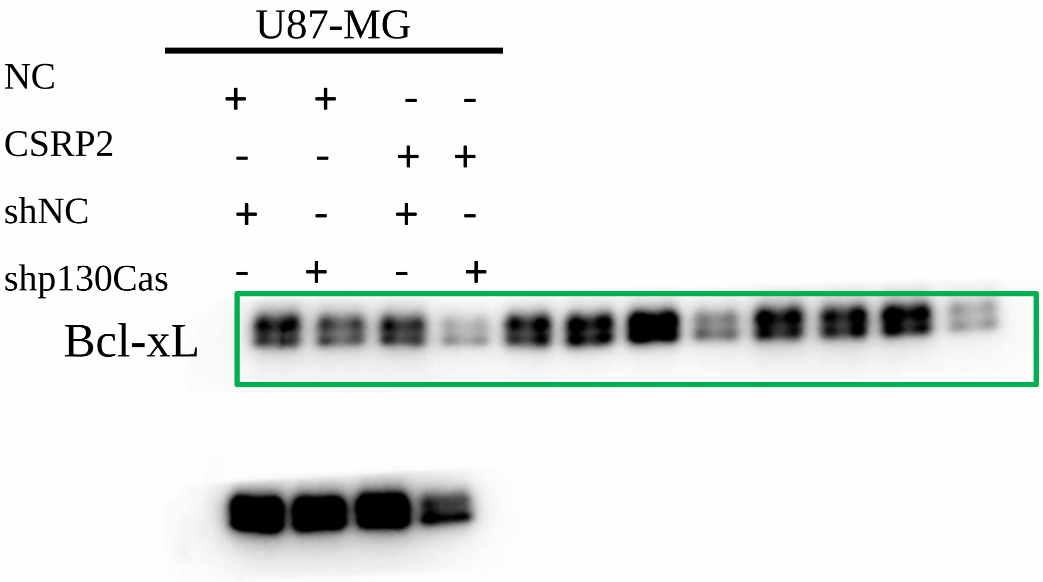

The membrane was imaged with Azure Biosystems 300

Full unedited gel for Figure 6I

Green： Statistical graph

Red： Representative graph

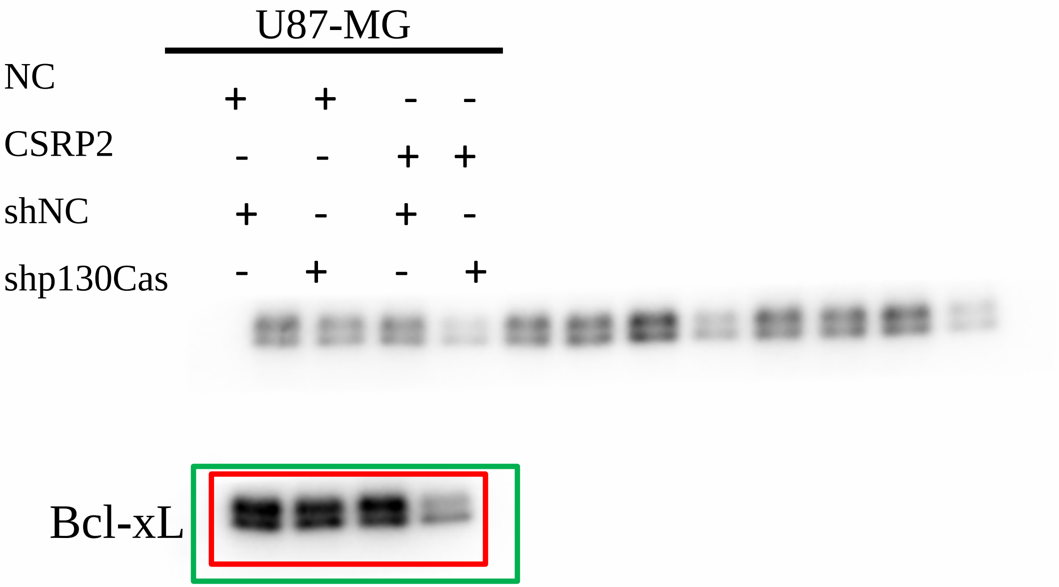

The membrane was imaged with Azure Biosystems 300

Full unedited gel for Figure 6I

Green： Statistical graph

Red： Representative graph

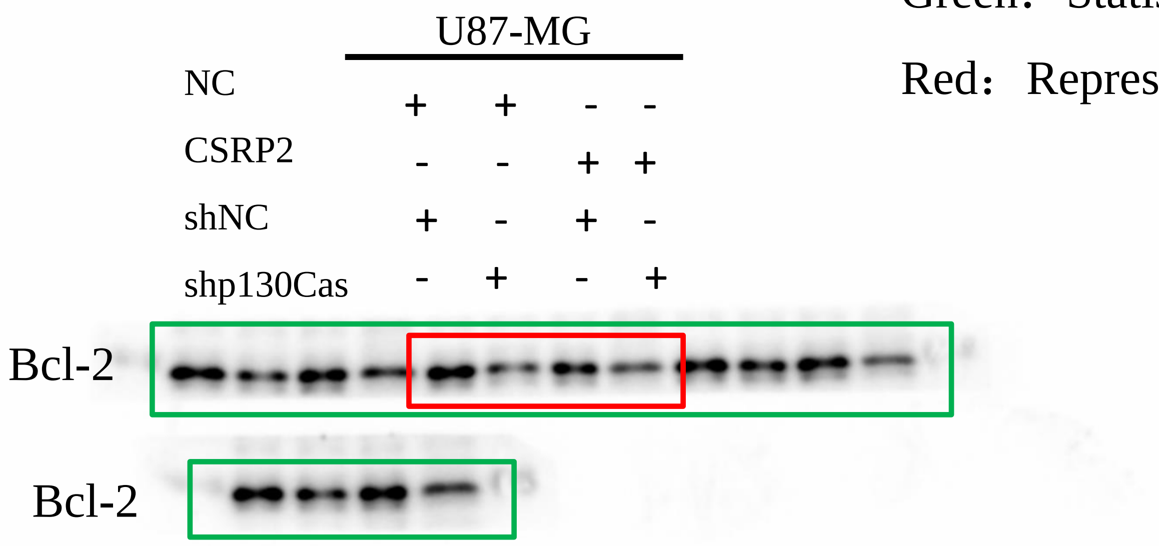

The membrane was imaged with Azure Biosystems 300

Full unedited gel for Figure 6I

U87-MG

NC	+	+	-	-
CSRP2	-	-	+	+
shNC	+	-	+	-
shp130Cas	-	+	-	+

Green： Statistical graph

GAPDH

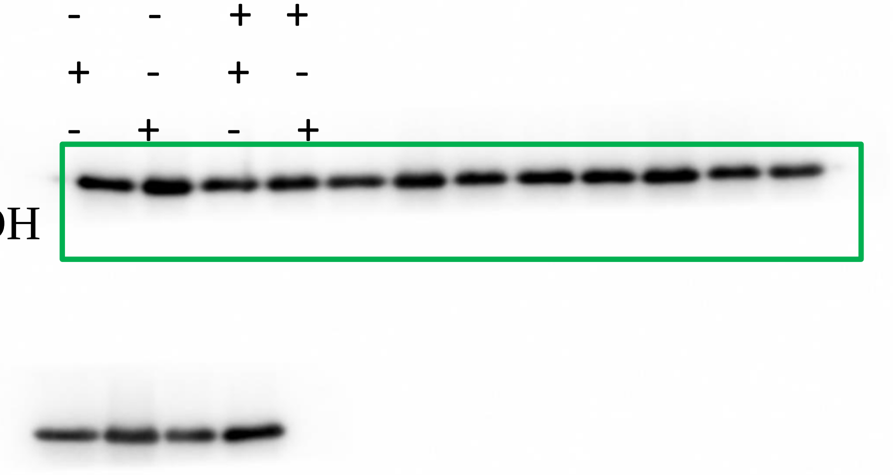

The membrane was imaged with Azure Biosystems 300

Full unedited gel for Figure 6I

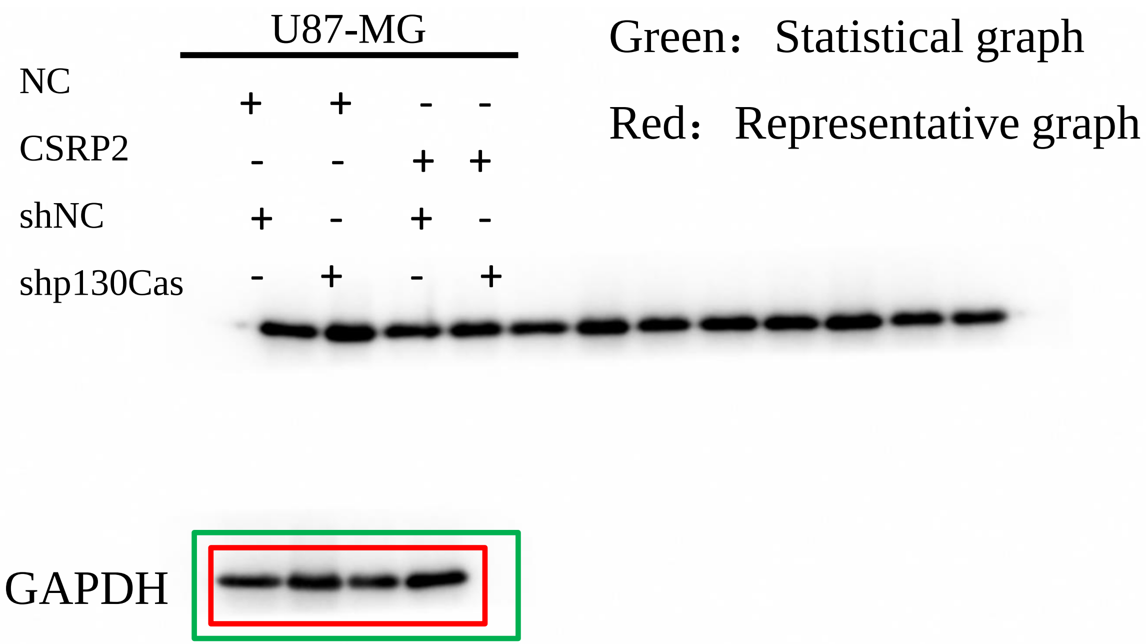

The membrane was imaged with Azure Biosystems 300

Full unedited gel for Figure 6I

Green： Statistical graph

Red： Representative graph

	U251			
NC	+	+	-	-
CSRP2	-	-	+	+
shNC	+	-	+	-
shp130Cas	-	+	-	+

CSRP2

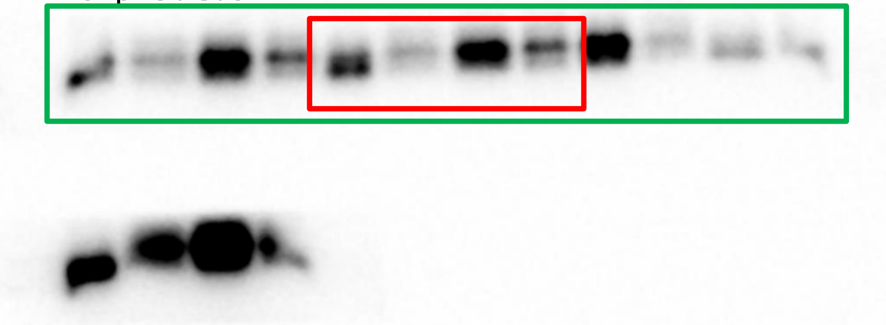

The membrane was imaged with Azure Biosystems 300

Full unedited gel for Figure 6I

Green： Statistical graph

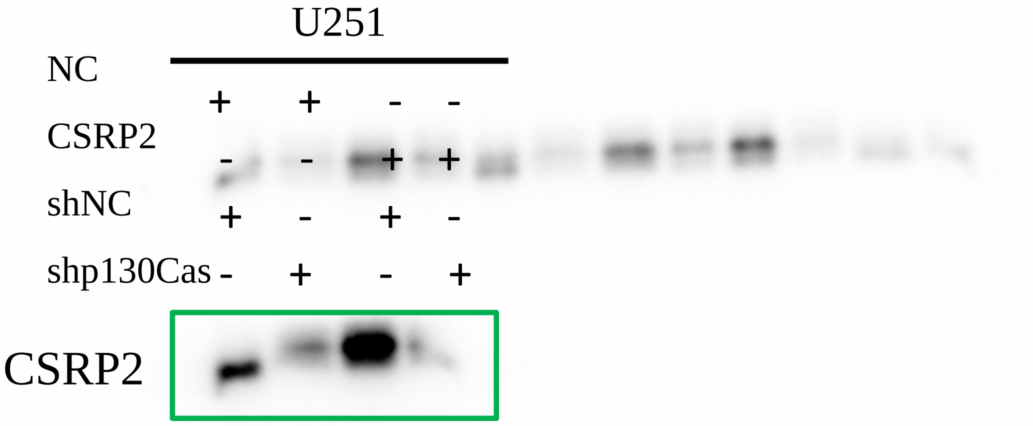

The membrane was imaged with Azure Biosystems 300

Full unedited gel for Figure 6I

Green： Statistical graph

Red： Representative graph

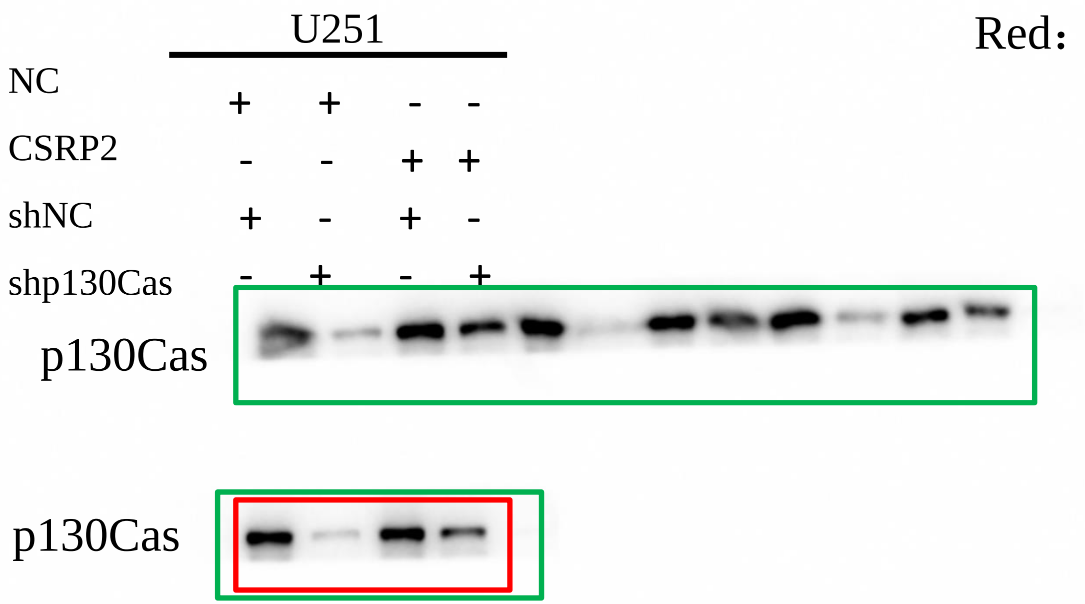

The membrane was imaged with Azure Biosystems 300

Full unedited gel for Figure 6I

Green: Statistical graph

Red: Representative graph

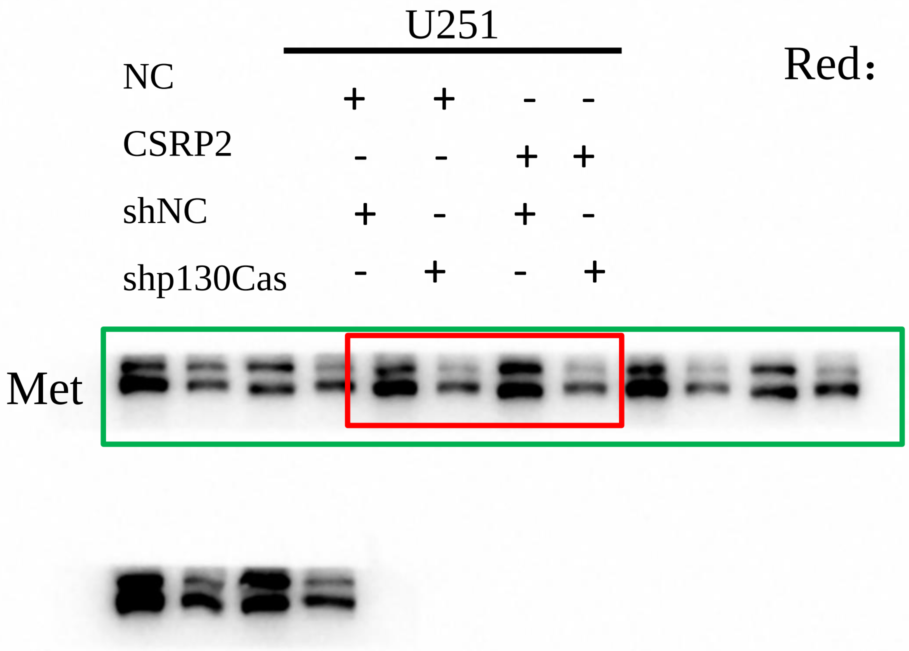

The membrane was imaged with Azure Biosystems 300

Full unedited gel for Figure 6I

Green: Statistical graph

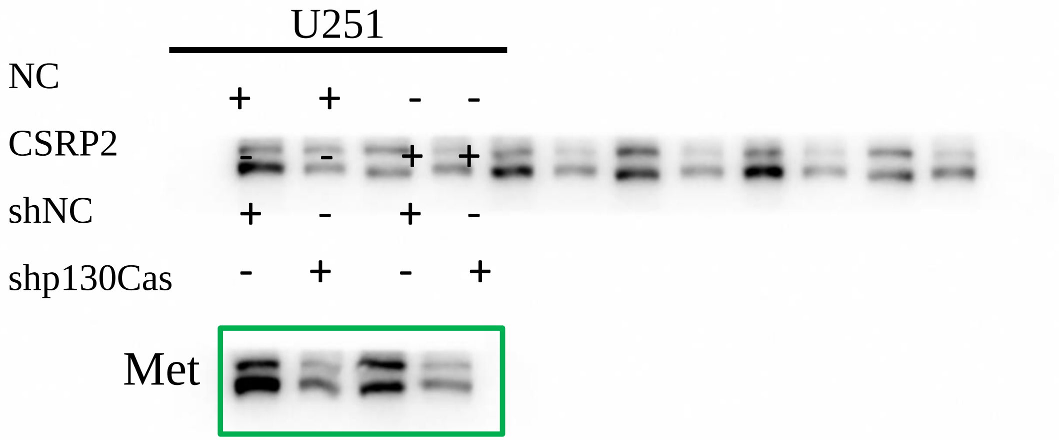

The membrane was imaged with Azure Biosystems 300

Full unedited gel for Figure 6I

Green： Statistical graph

Red： Representative graph

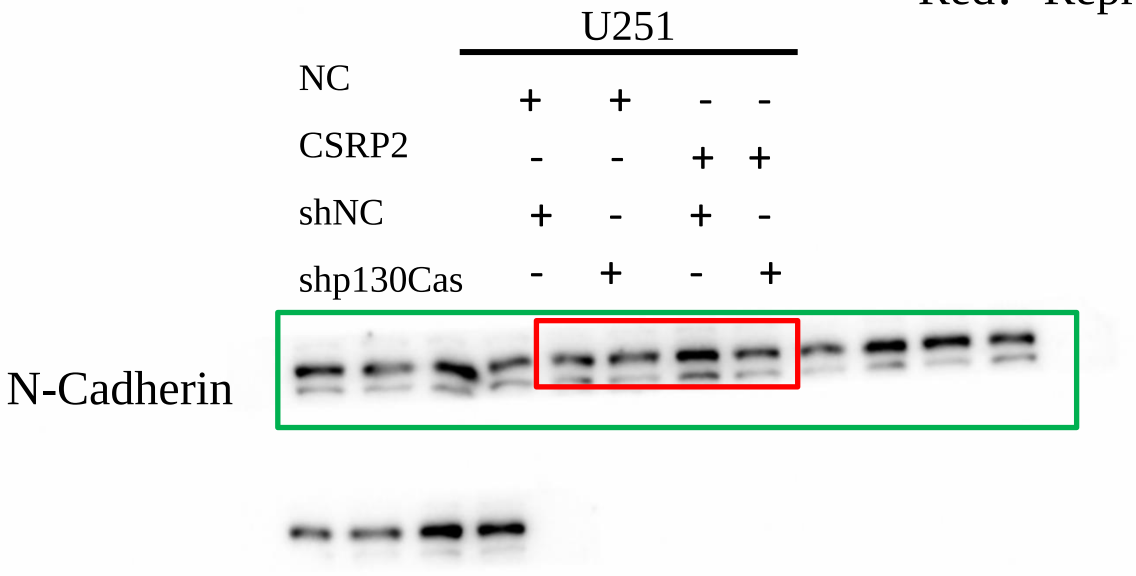

The membrane was imaged with Azure Biosystems 300

Full unedited gel for Figure 6I

Green： Statistical graph

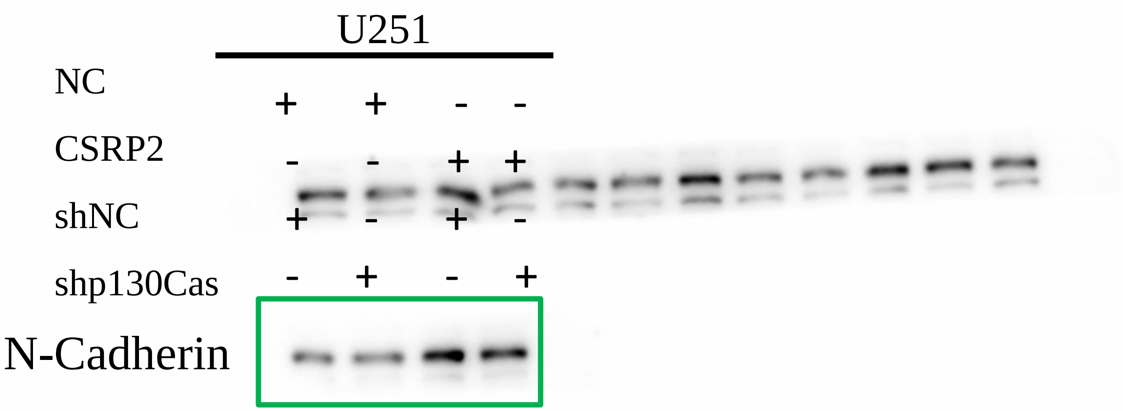

The membrane was imaged with Azure Biosystems 300

Full unedited gel for Figure 6I

Green： Statistical graph

Red： Representative graph

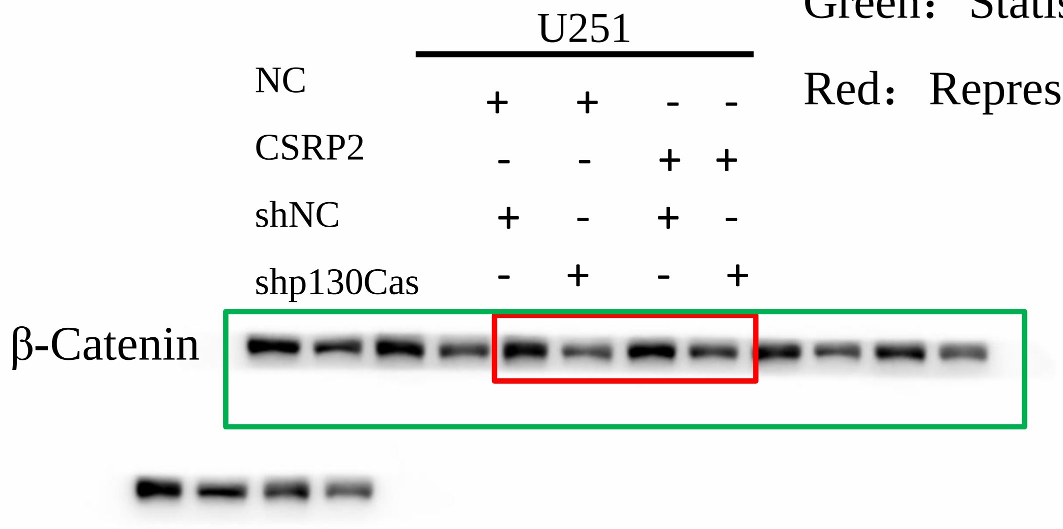

The membrane was imaged with Azure Biosystems 300

Full unedited gel for Figure 6I

Green： Statistical graph

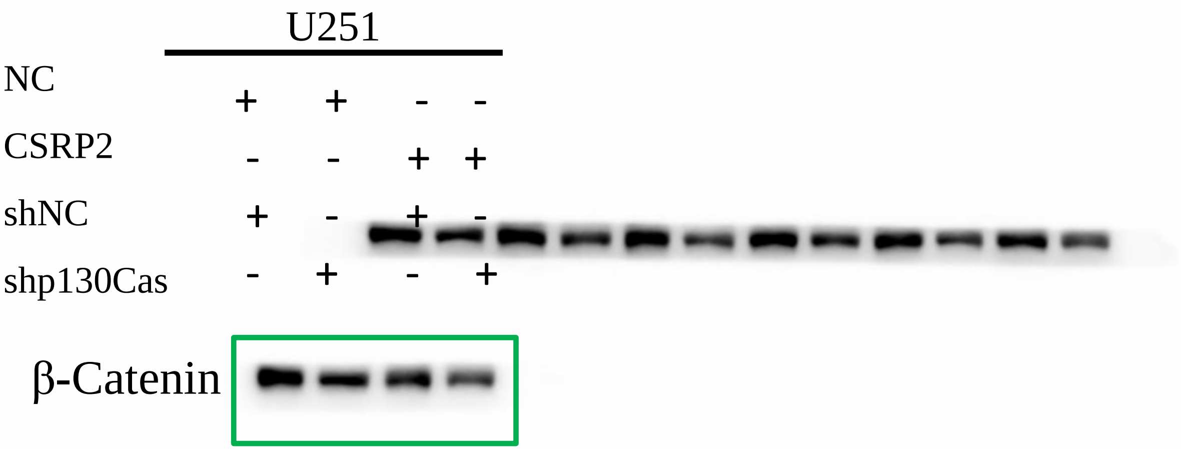

The membrane was imaged with Azure Biosystems 300

Full unedited gel for Figure 6I

Green： Statistical graph

Red： Representative graph

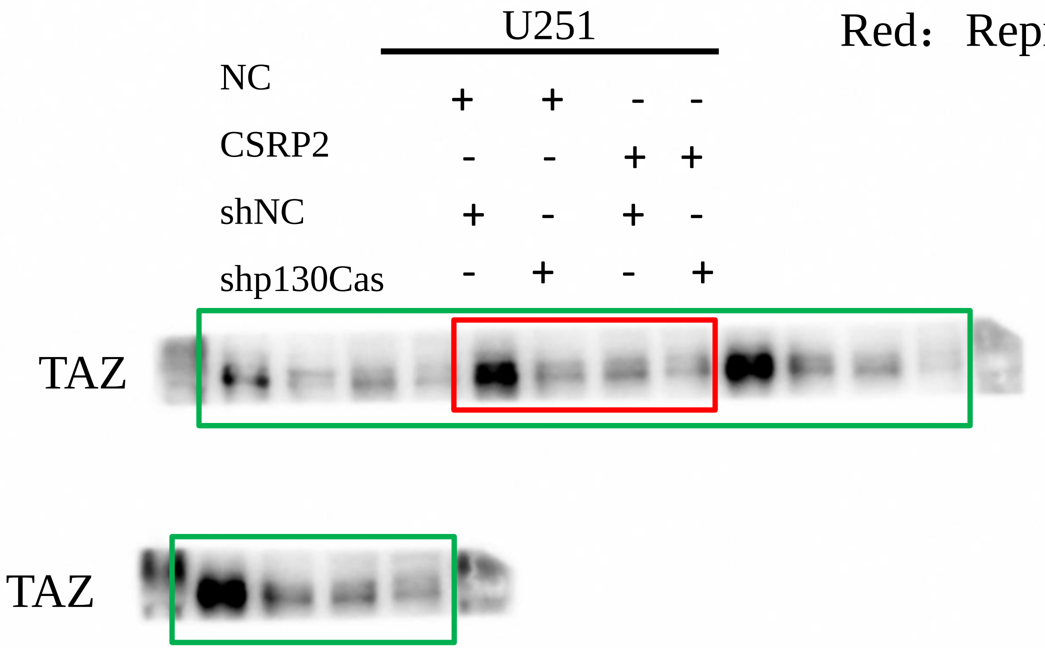

The membrane was imaged with Azure Biosystems 300

Full unedited gel for Figure 6I

Green： Statistical graph

Red： Representative graph

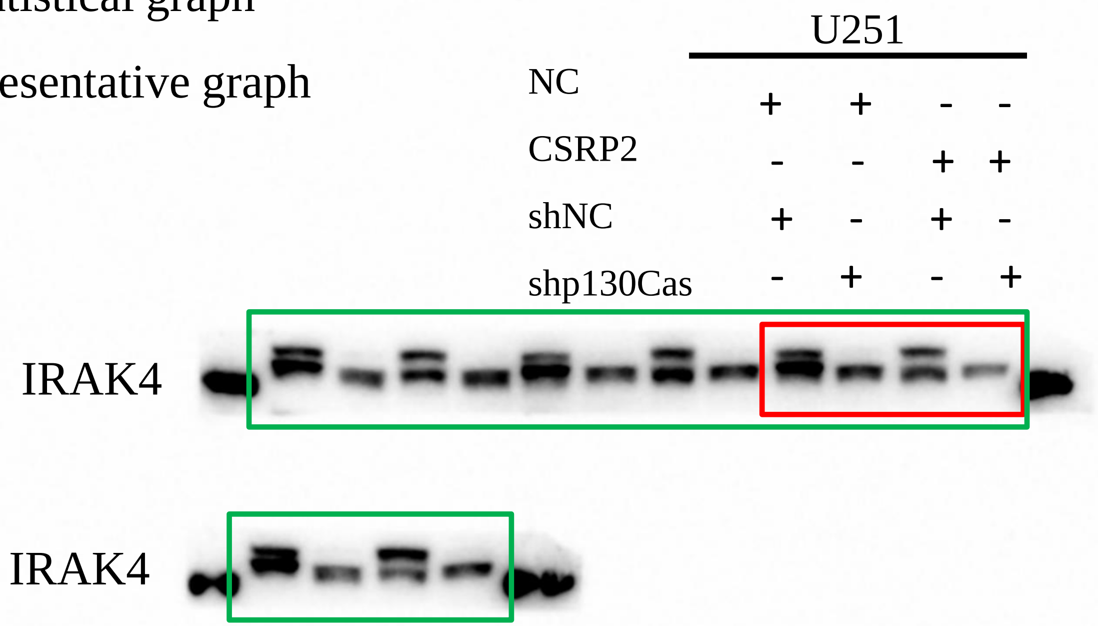

The membrane was imaged with Azure Biosystems 300

Full unedited gel for Figure 6I

Green： Statistical graph

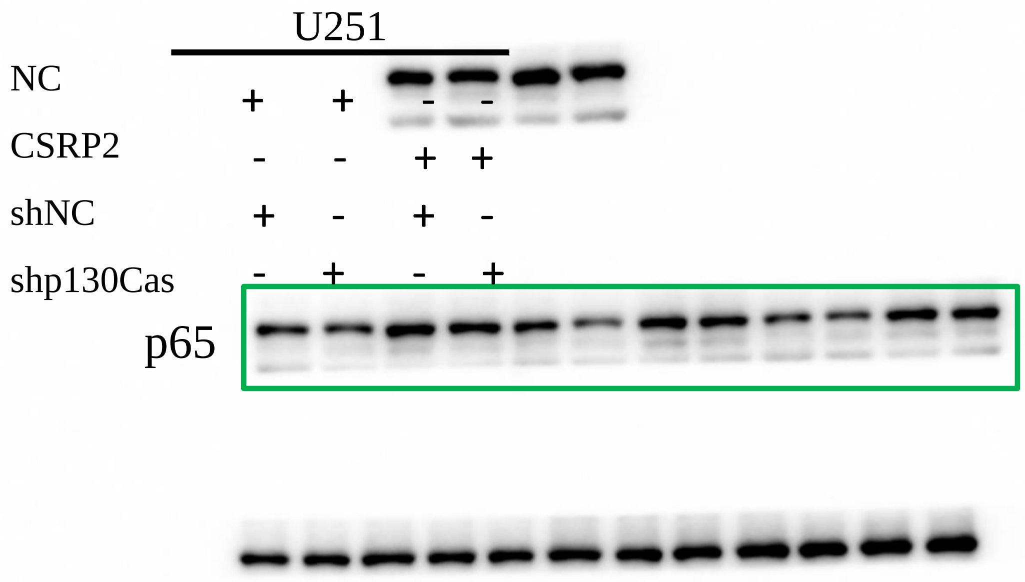

The membrane was imaged with Azure Biosystems 300

Full unedited gel for Figure 6I

Green： Statistical graph

Red： Representative graph

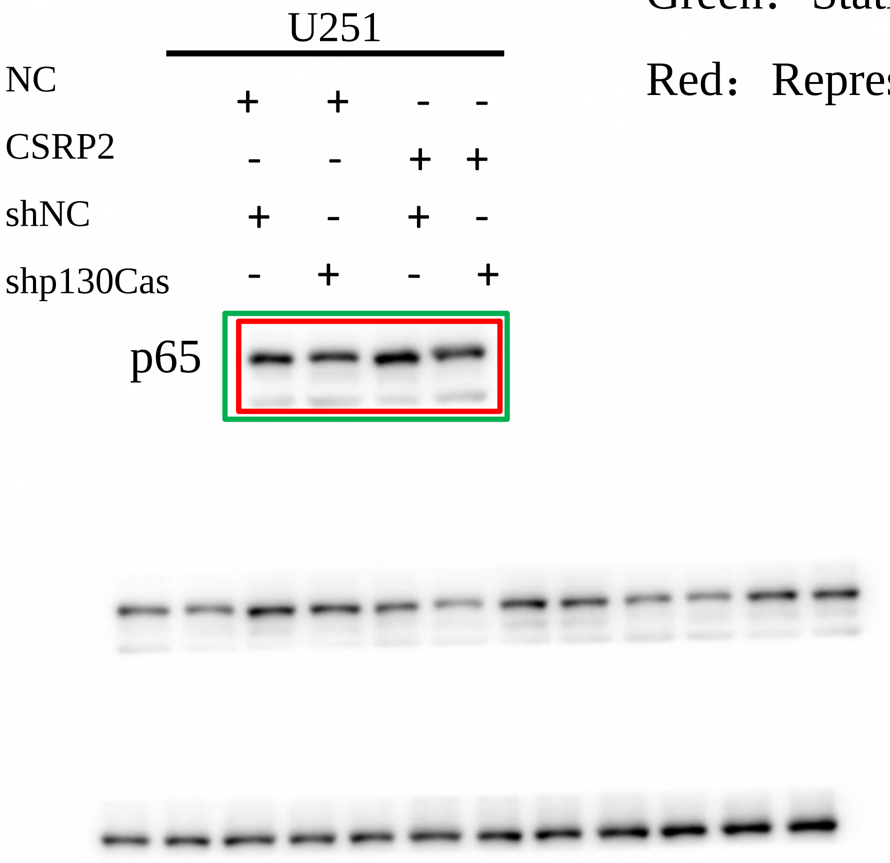

The membrane was imaged with Azure Biosystems 300

Full unedited gel for Figure 6I

Green： Statistical graph

Red： Representative graph

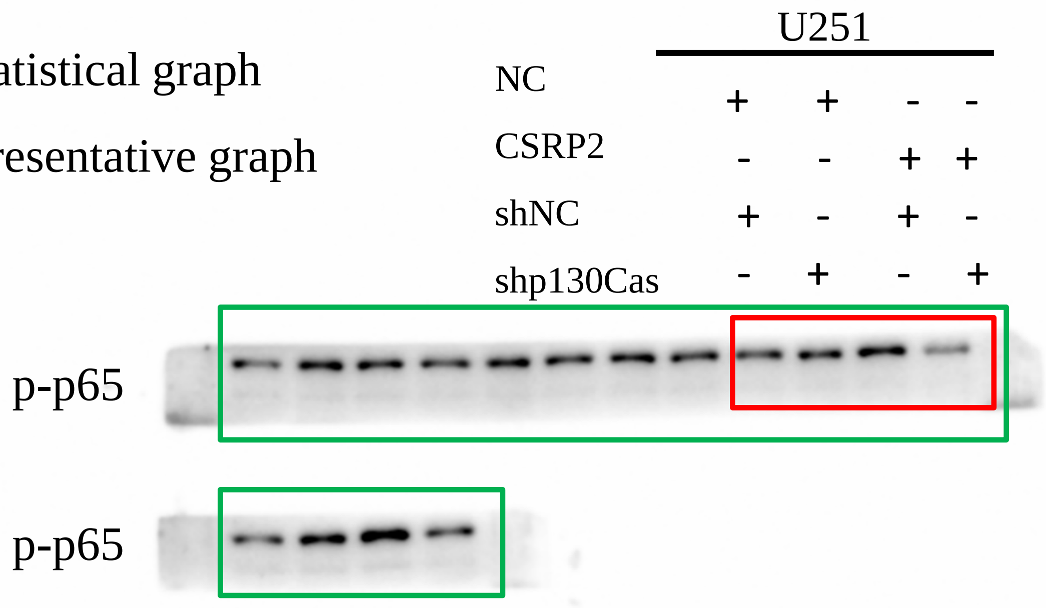

The membrane was imaged with Azure Biosystems 300

Full unedited gel for Figure 6I

Green： Statistical graph

Red： Representative graph

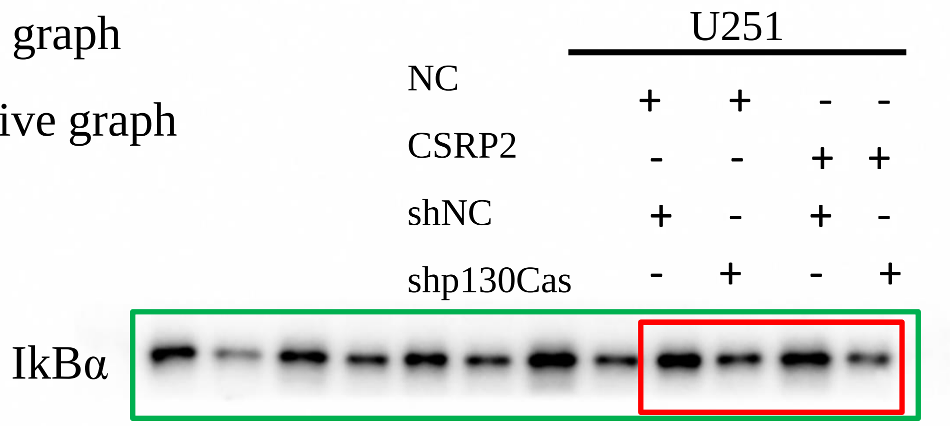

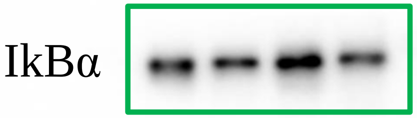

The membrane was imaged with Azure Biosystems 300

Full unedited gel for Figure 6I

Green： Statistical graph

Red： Representative graph

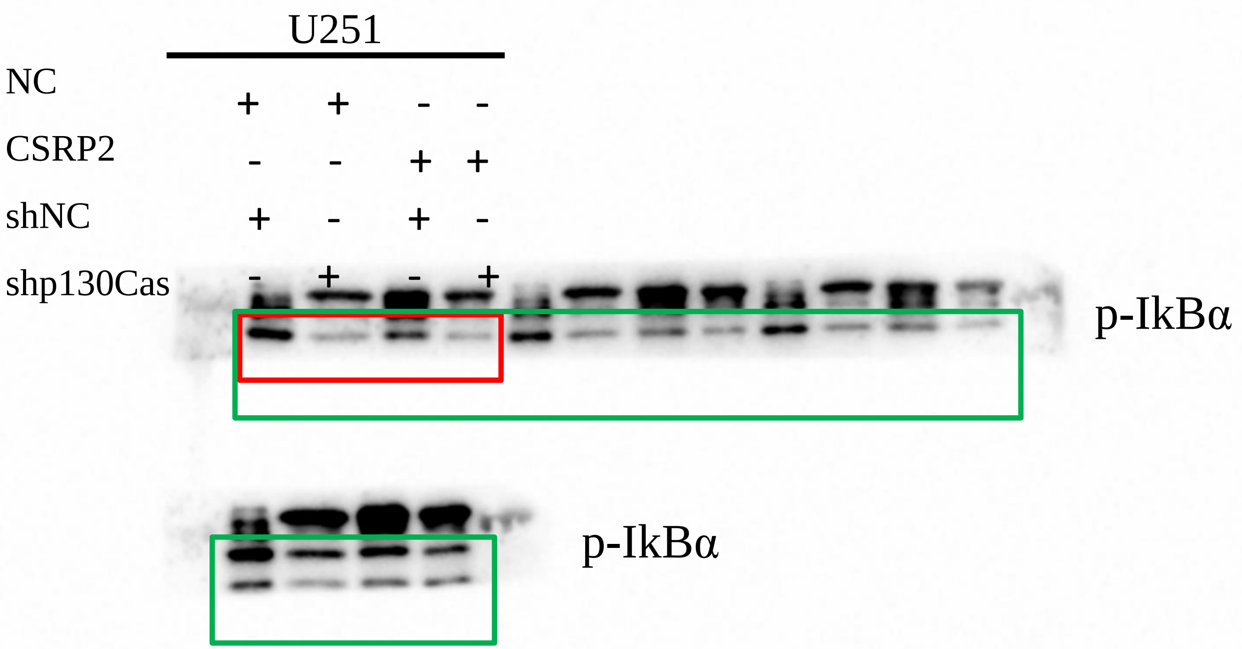

The membrane was imaged with Azure Biosystems 300

Full unedited gel for Figure 6I

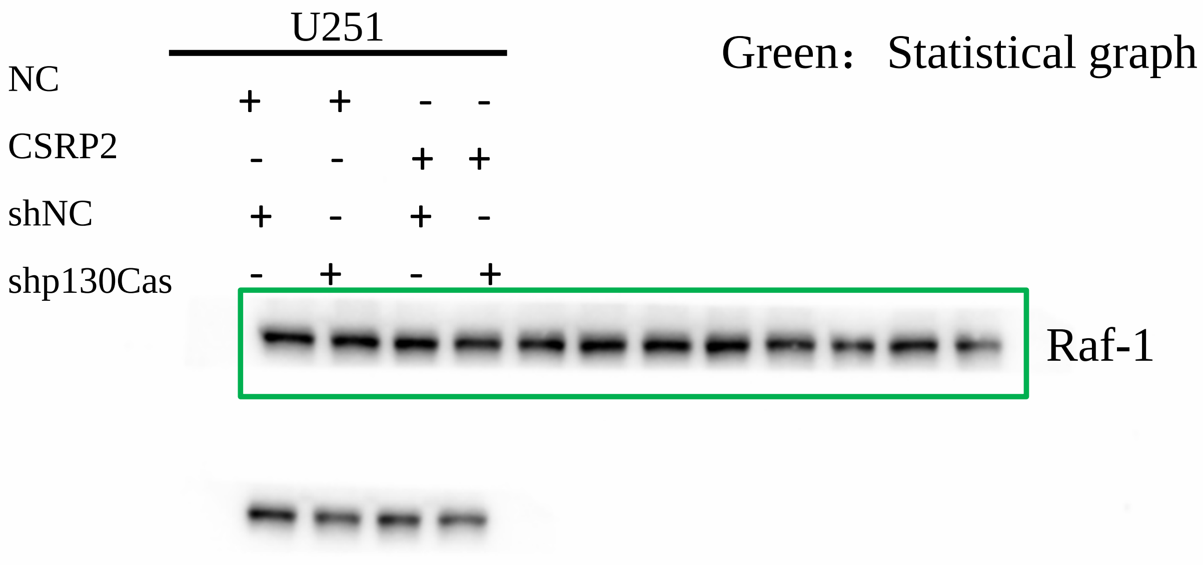

The membrane was imaged with Azure Biosystems 300

Full unedited gel for Figure 6I

Green： Statistical graph

Red： Representative graph

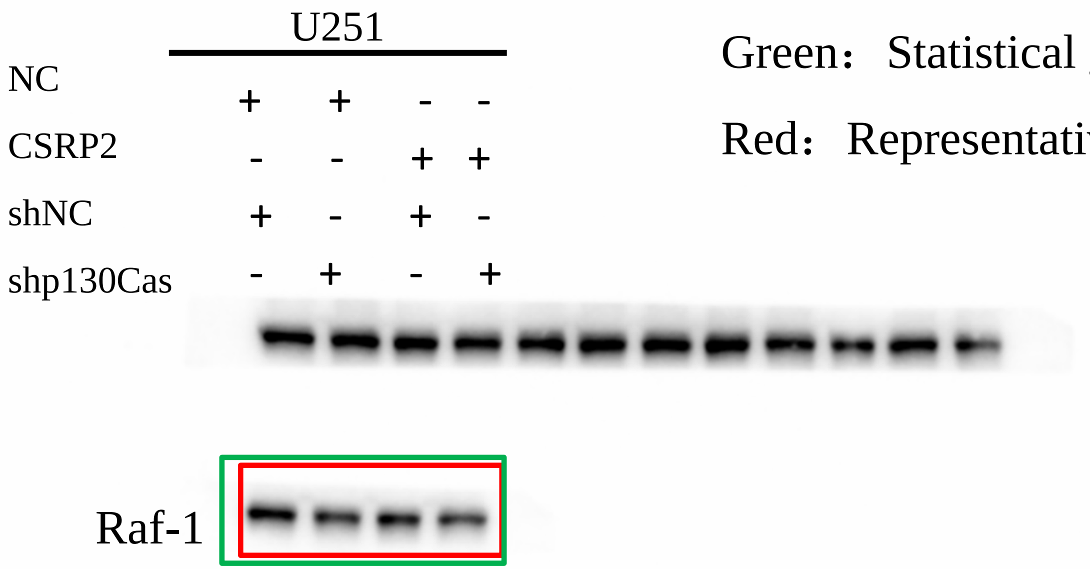

The membrane was imaged with Azure Biosystems 300

Full unedited gel for Figure 6I

Green： Statistical graph

Red： Representative graph

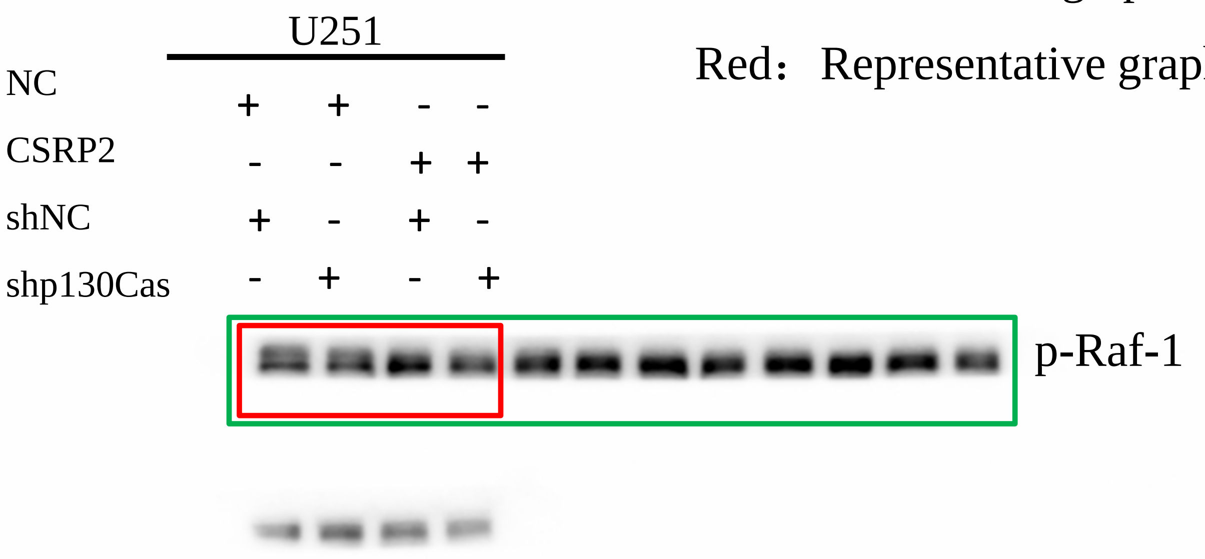

The membrane was imaged with Azure Biosystems 300

Full unedited gel for Figure 6I

Green：Statistical graph

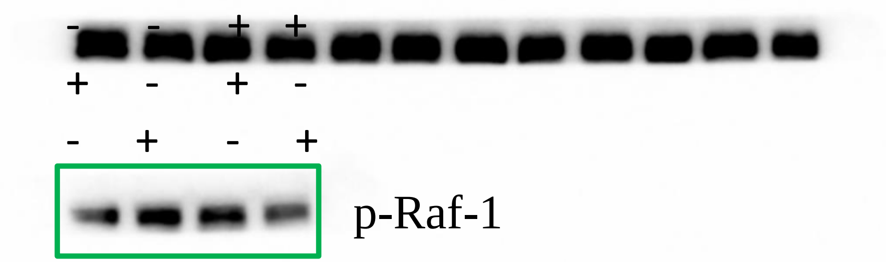

The membrane was imaged with Azure Biosystems 300

Full unedited gel for Figure 6I

Green： Statistical graph

Red： Representative graph

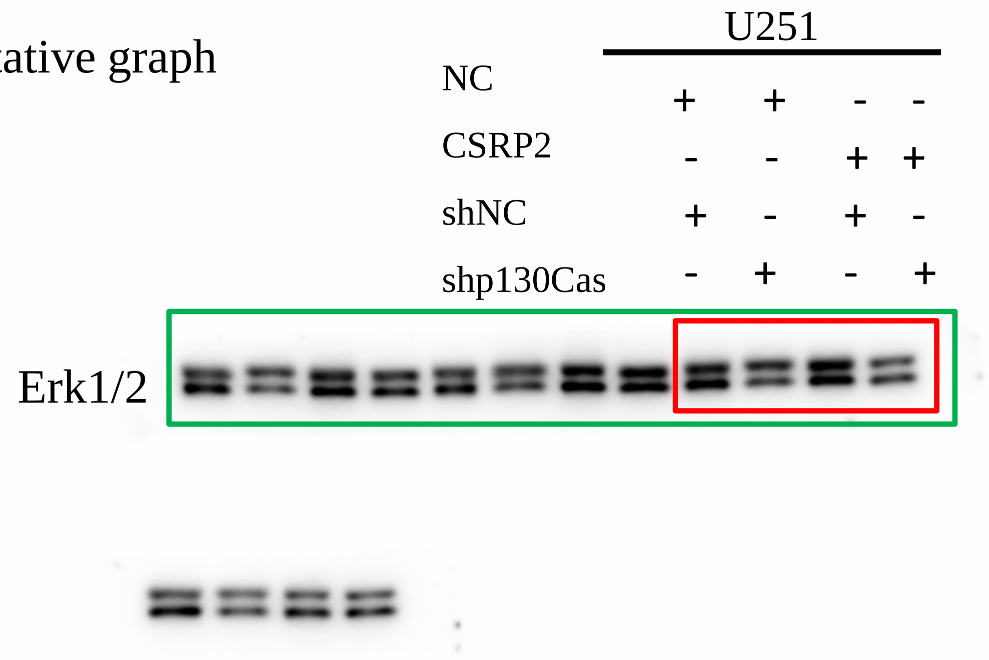

The membrane was imaged with Azure Biosystems 300

Full unedited gel for Figure 6I

Green： Statistical graph

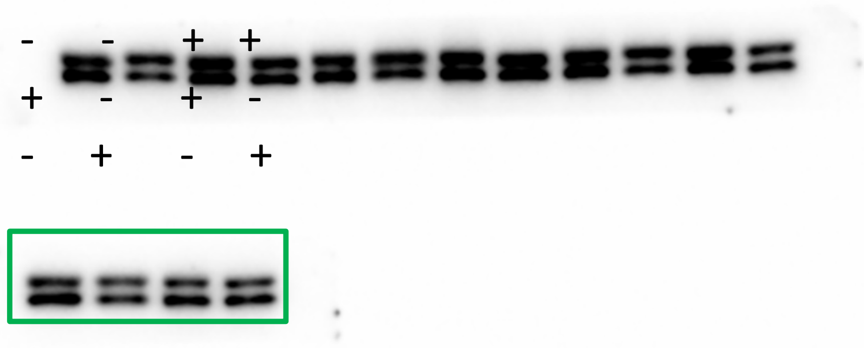

The membrane was imaged with Azure Biosystems 300

Full unedited gel for Figure 6I

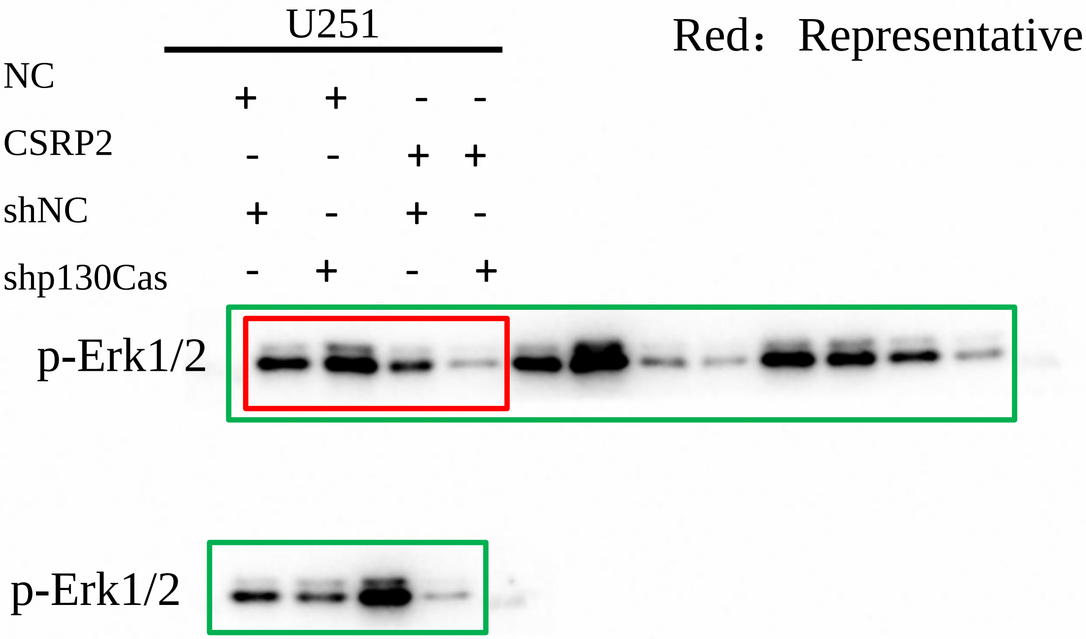

Green: Statistical graph

Red: Representative graph

The membrane was imaged with Azure Biosystems 300

Full unedited gel for Figure 6I

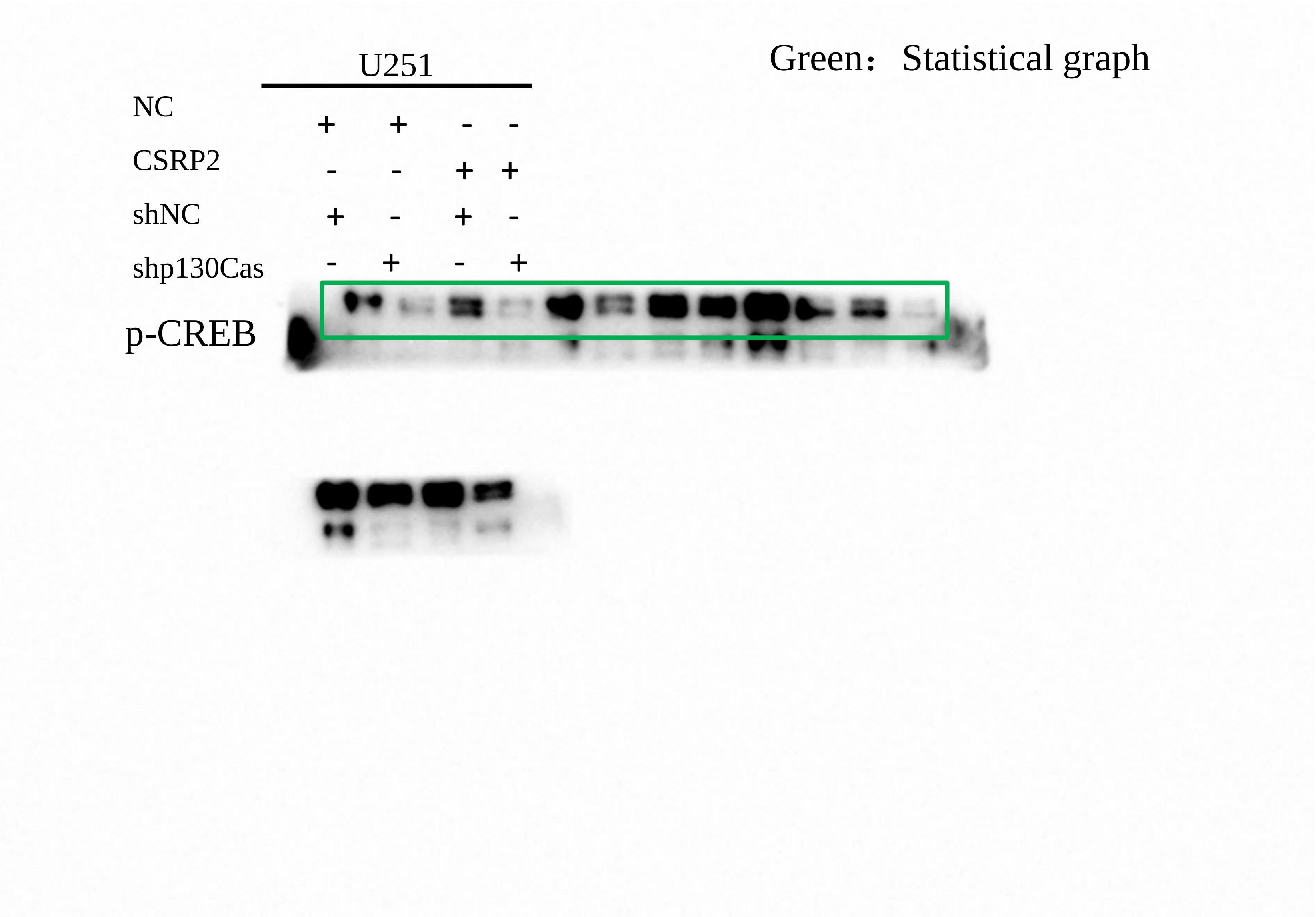

The membrane was imaged with Azure Biosystems 300

Full unedited gel for Figure 6I

Green： Statistical graph

Red： Representative graph

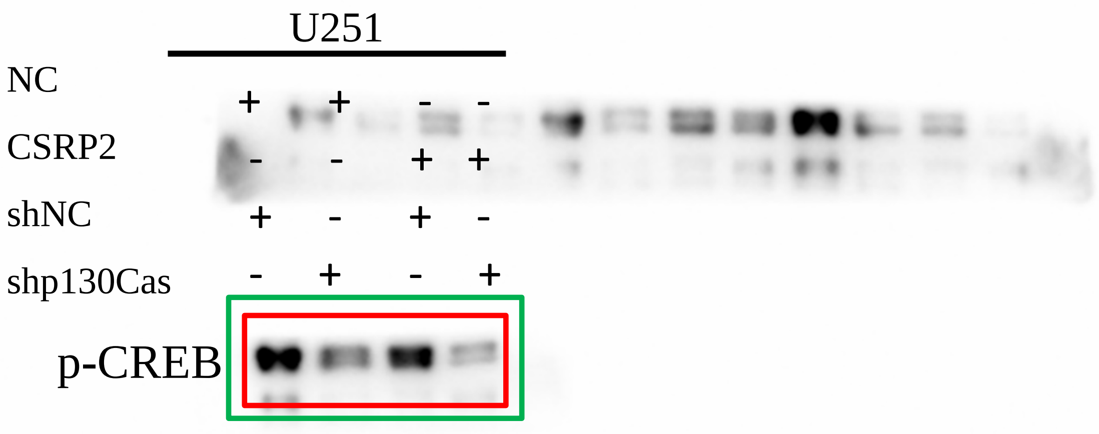

The membrane was imaged with Azure Biosystems 300

Full unedited gel for Figure 6I

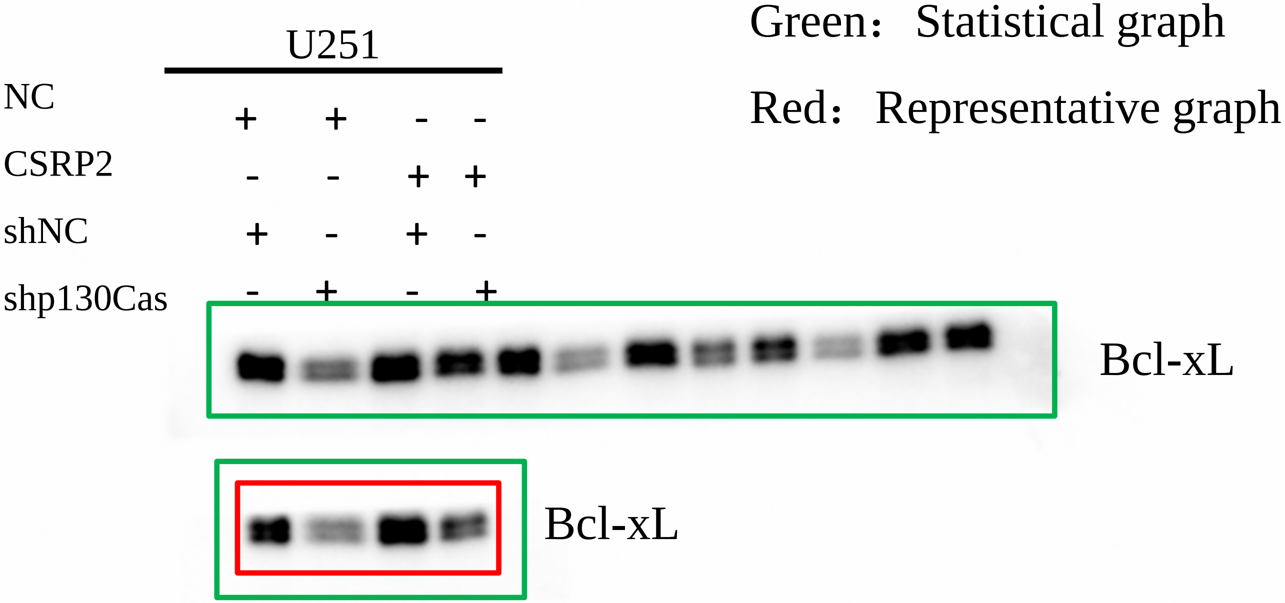

The membrane was imaged with Azure Biosystems 300

Full unedited gel for Figure 6I

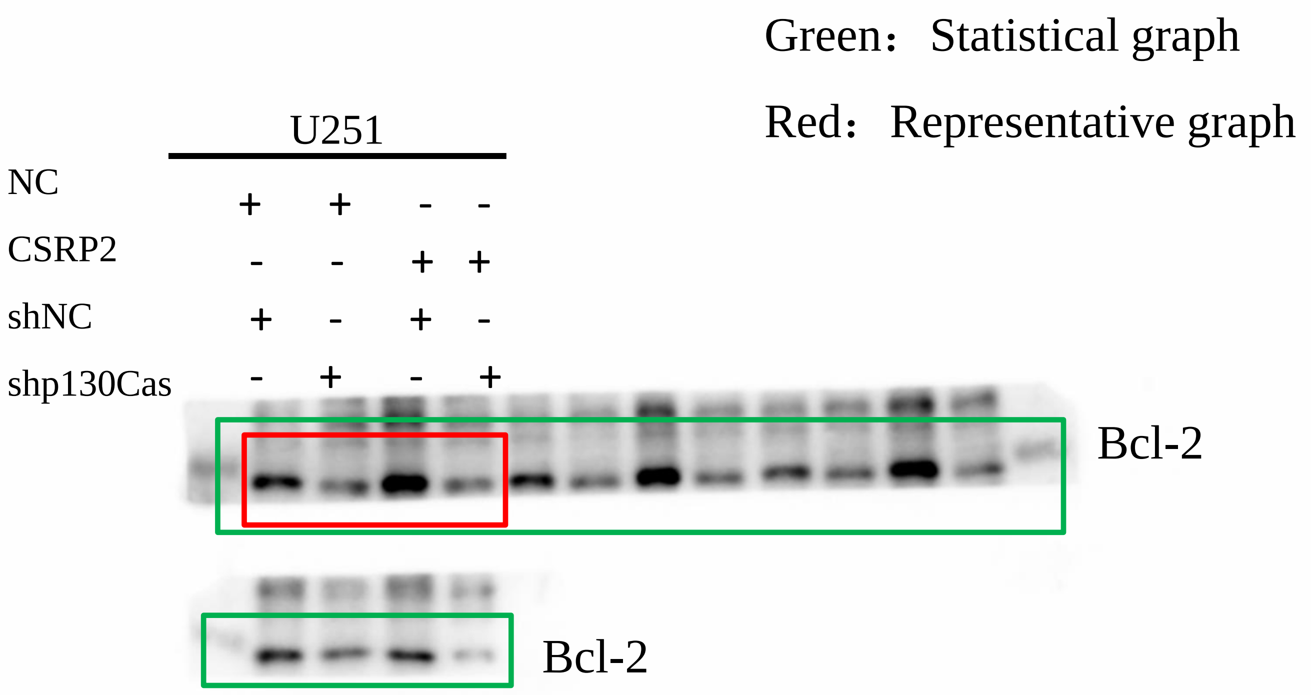

The membrane was imaged with Azure Biosystems 300

Full unedited gel for Figure 6I

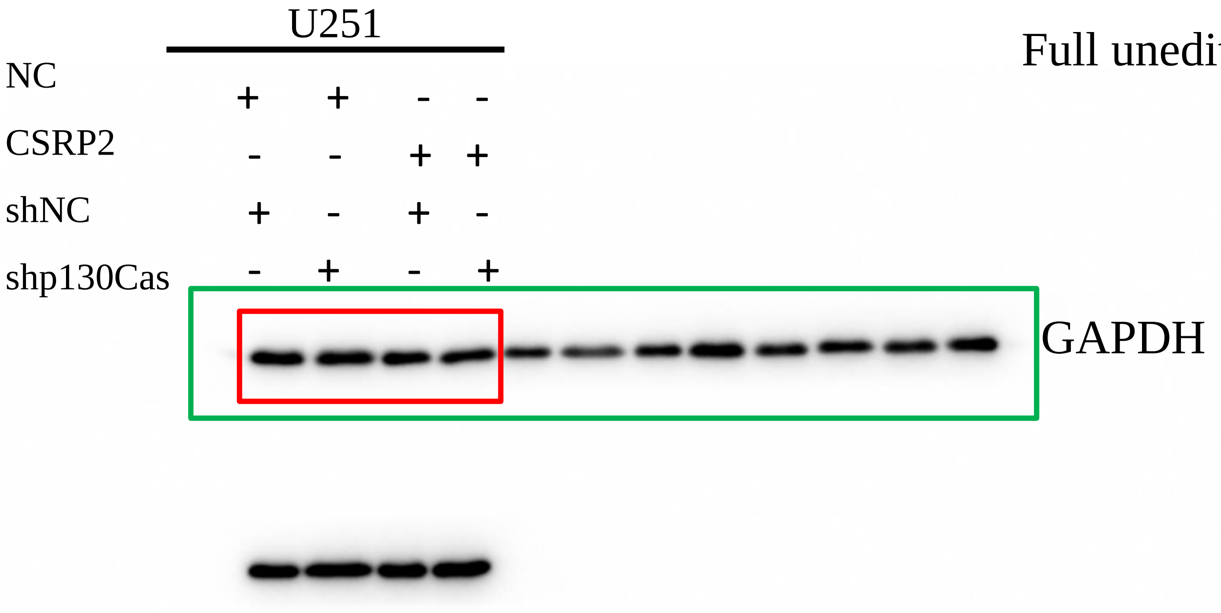

Green: Statistical graph

Red: Representative graph

The membrane was imaged with Azure Biosystems 300

Full unedited gel for Figure 6I

Green： Statistical graph

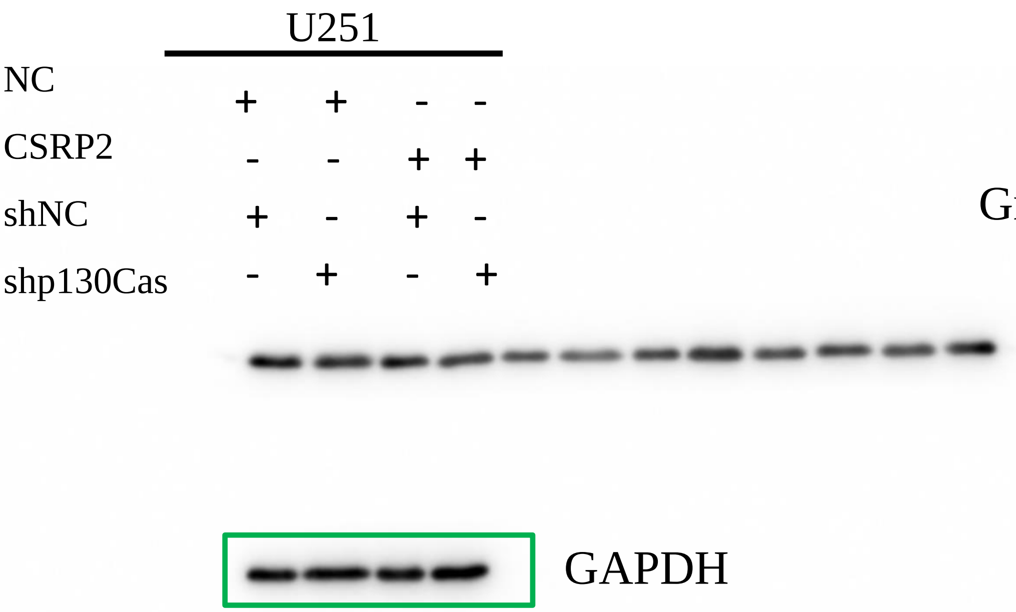

The membrane was imaged with Azure Biosystems 300

Full unedited gel for Figure 8L

Red： Representative graph

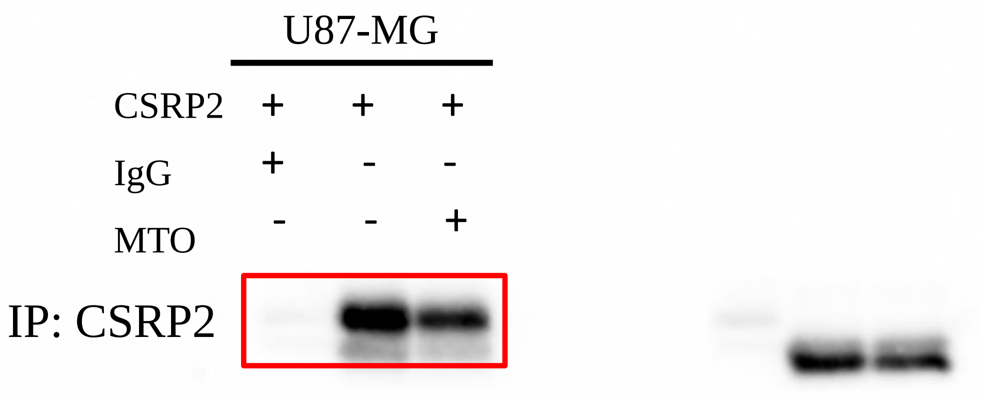

The membrane was imaged with Azure Biosystems 300

Full unedited gel for Figure 8L

Red： Representative graph

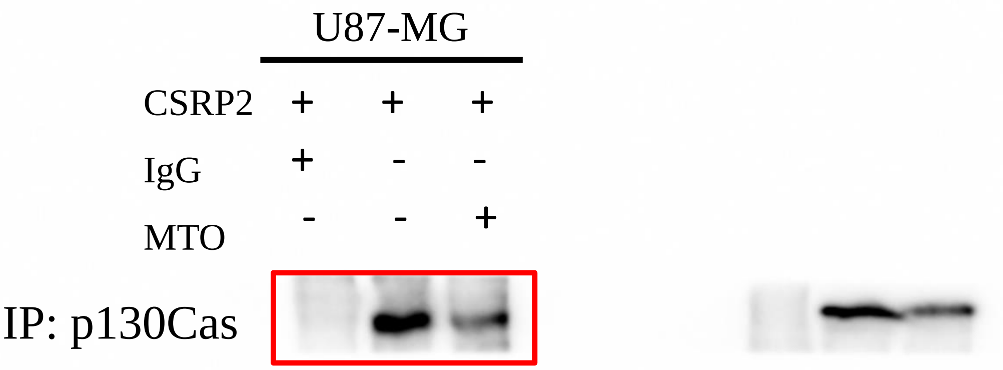

The membrane was imaged with Azure Biosystems 300

Full unedited gel for Figure 8L

Red： Representative graph

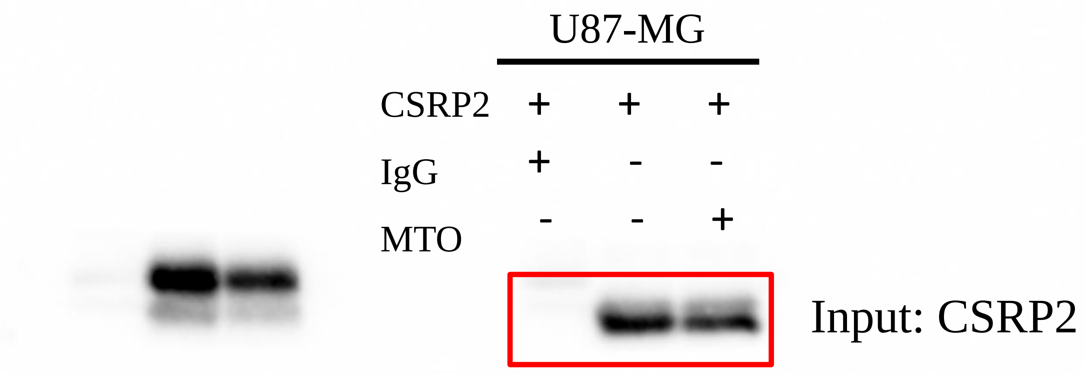

The membrane was imaged with Azure Biosystems 300

Full unedited gel for Figure 8L

Red： Representative graph

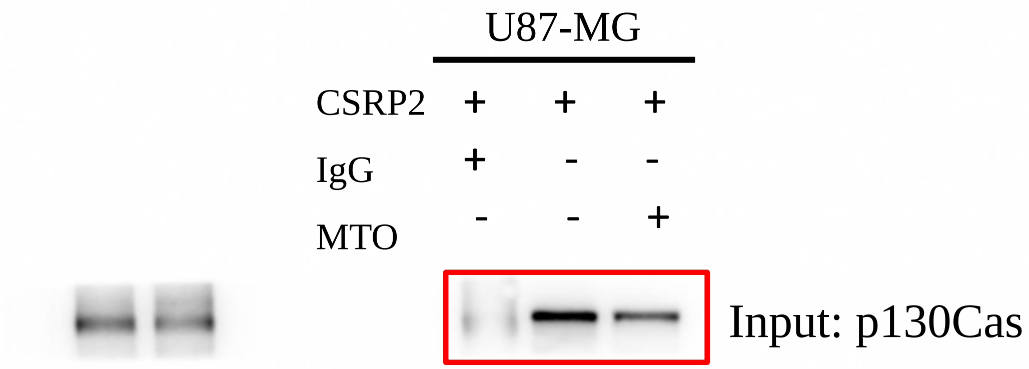

The membrane was imaged with Azure Biosystems 300

Full unedited gel for Figure 8L

Red： Representative graph

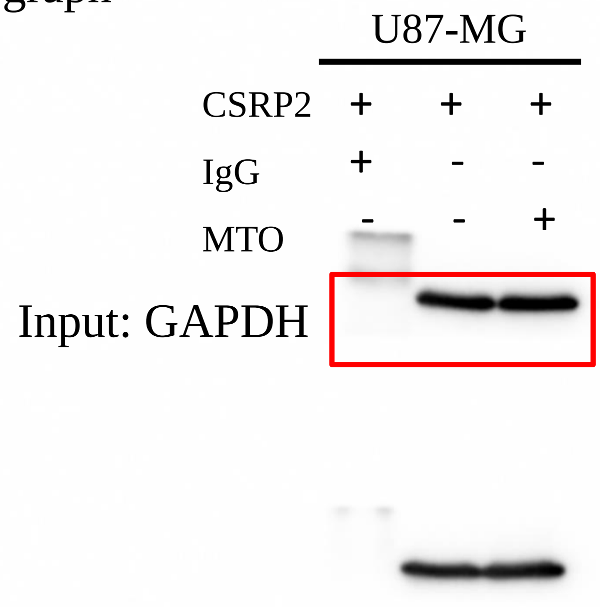

The membrane was imaged with Azure Biosystems 300

Full unedited gel for Figure 8L

Red： Representative graph

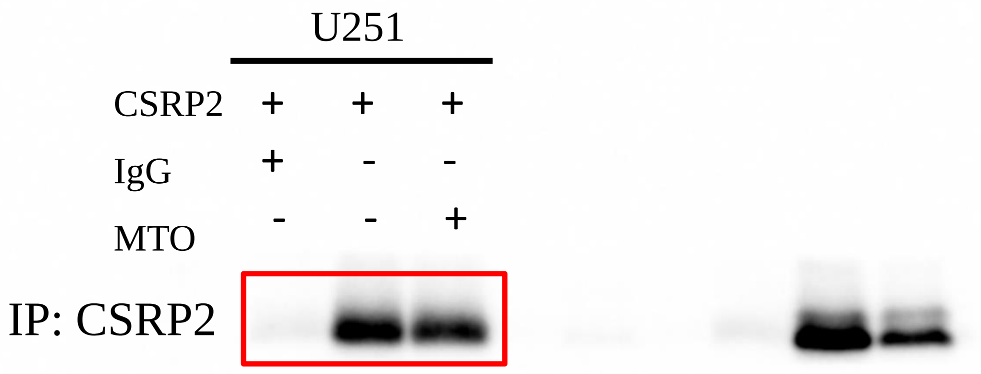

The membrane was imaged with Azure Biosystems 300

Full unedited gel for Figure 8L

Red： Representative graph

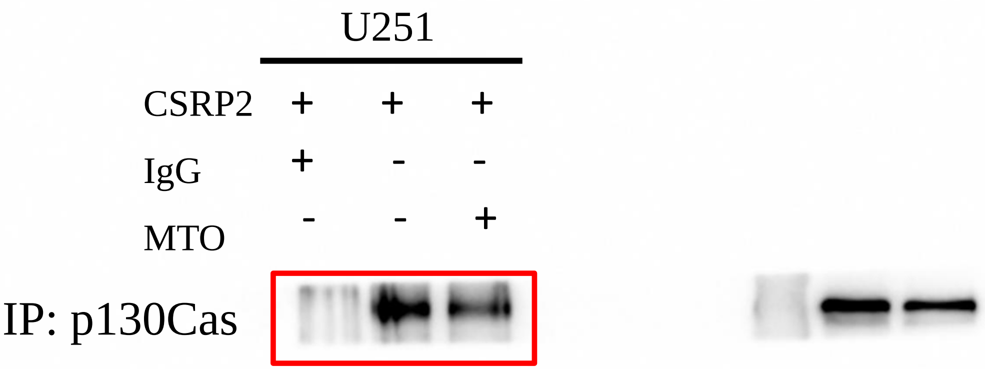

The membrane was imaged with Azure Biosystems 300

Full unedited gel for Figure 8L

Red： Representative graph

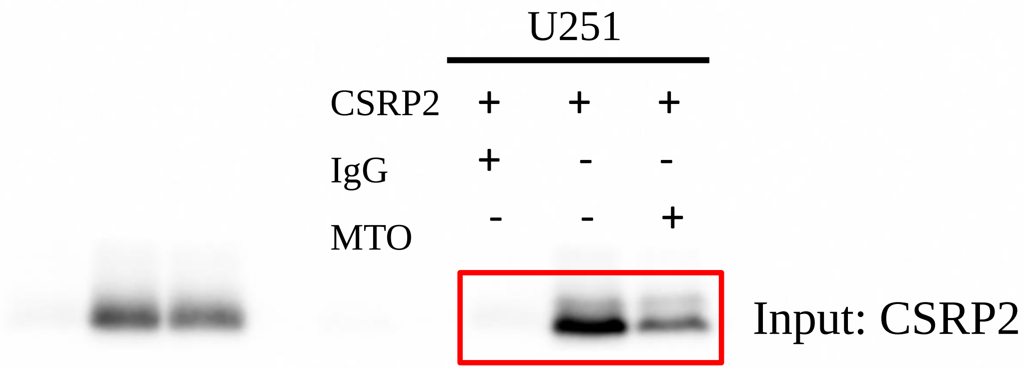

The membrane was imaged with Azure Biosystems 300

Full unedited gel for Figure 8L

Red：Representative graph

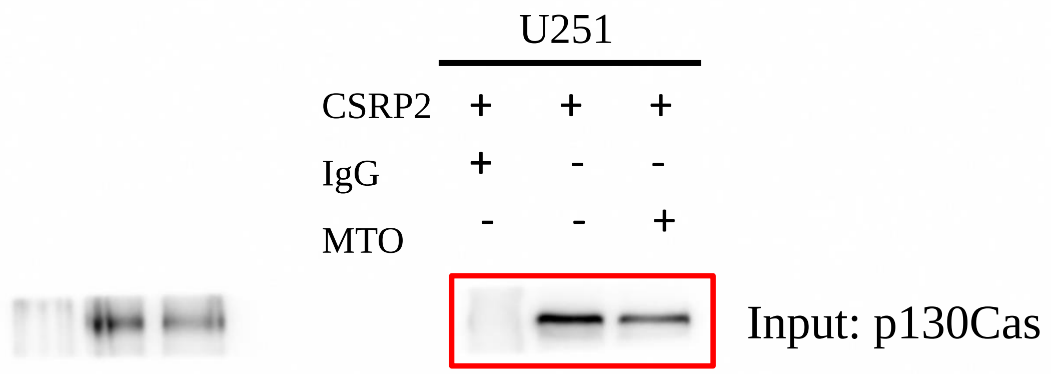

The membrane was imaged with Azure Biosystems 300

Full unedited gel for Figure 8L

Red： Representative graph

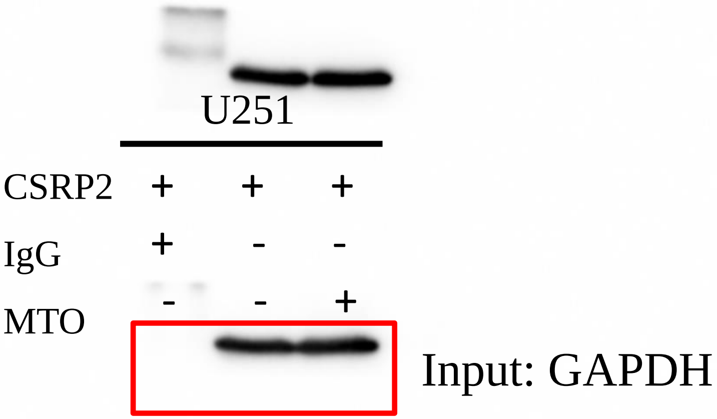

The membrane was imaged with Azure Biosystems 300

Full unedited gel for Figure 8M

Green： Statistical graph

Red： Representative graph

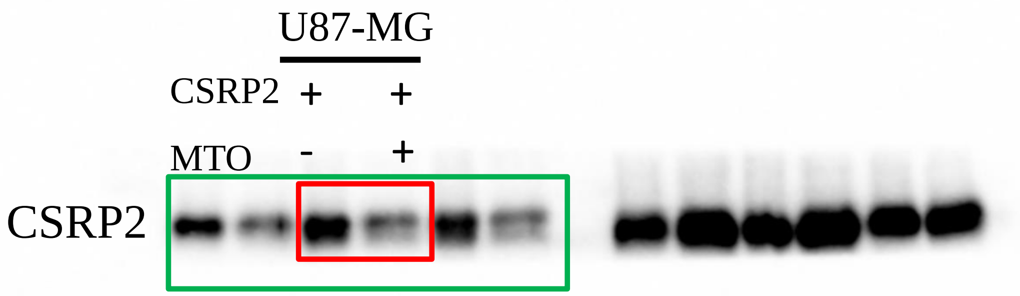

The membrane was imaged with Azure Biosystems 300

Full unedited gel for Figure 8M

Green： Statistical graph

Red： Representative graph

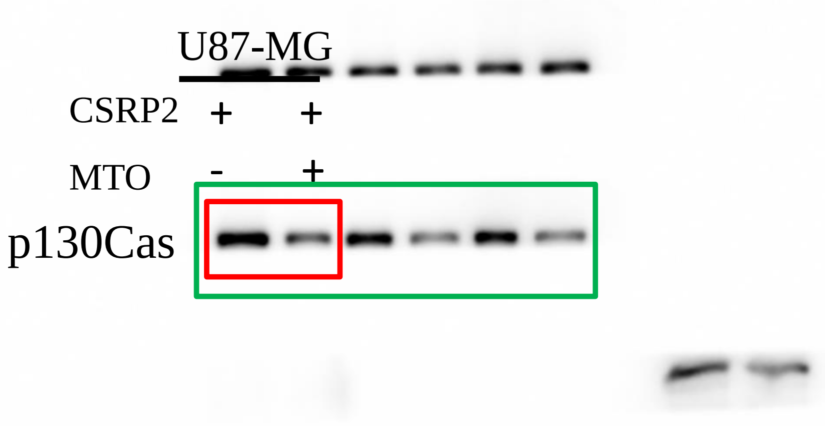

The membrane was imaged with Azure Biosystems 300

Full unedited gel for Figure 8M

Green： Statistical graph

Red： Representative graph

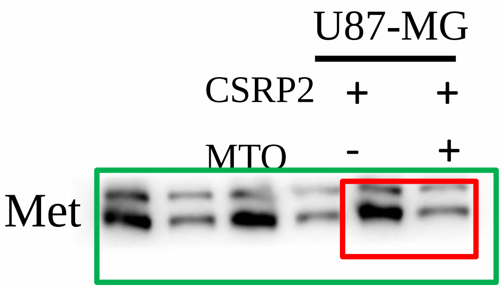

The membrane was imaged with Azure Biosystems 300

Full unedited gel for Figure 8M

Green： Statistical graph

Red： Representative graph

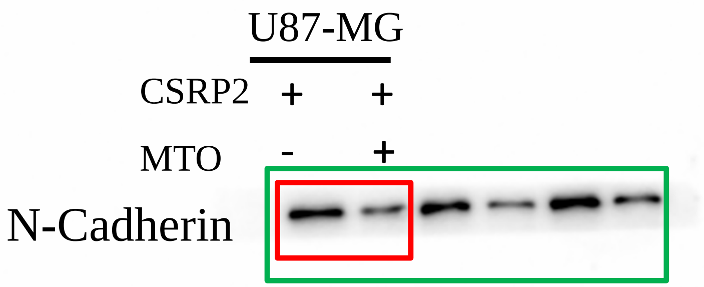

The membrane was imaged with Azure Biosystems 300

Full unedited gel for Figure 8M

Green： Statistical graph

Red： Representative graph

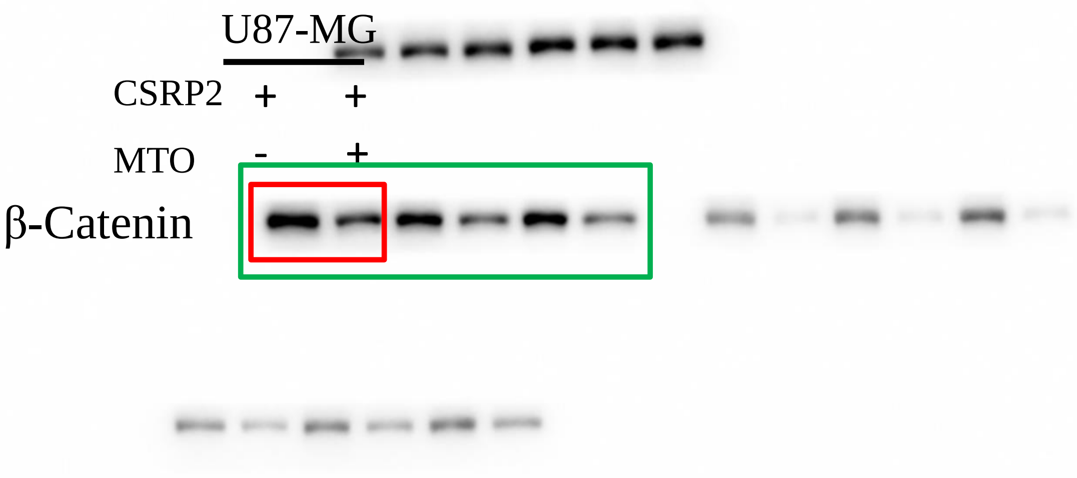

The membrane was imaged with Azure Biosystems 300

Full unedited gel for Figure 8M

Green：Statistical graph

Red：Representative graph

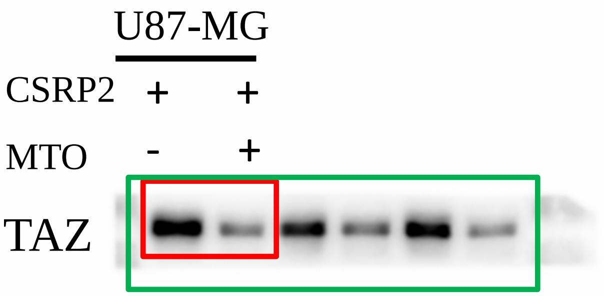

The membrane was imaged with Azure Biosystems 300

Full unedited gel for Figure 8M

Green： Statistical graph

Red： Representative graph

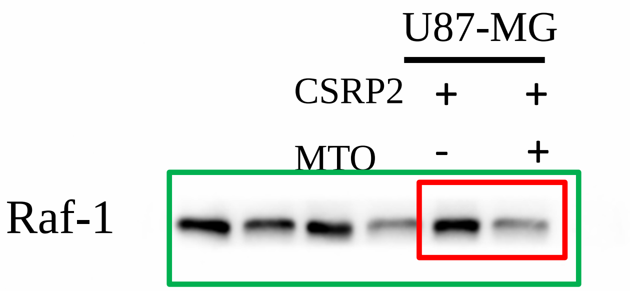

The membrane was imaged with Azure Biosystems 300

Full unedited gel for Figure 8M

Green： Statistical graph

Red： Representative graph

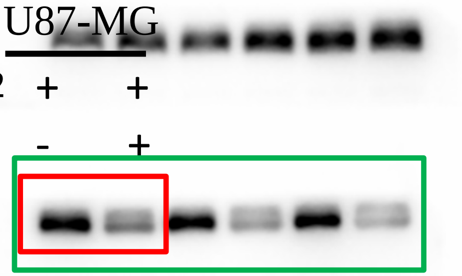

The membrane was imaged with Azure Biosystems 300

Full unedited gel for Figure 8M

Green： Statistical graph

Red： Representative graph

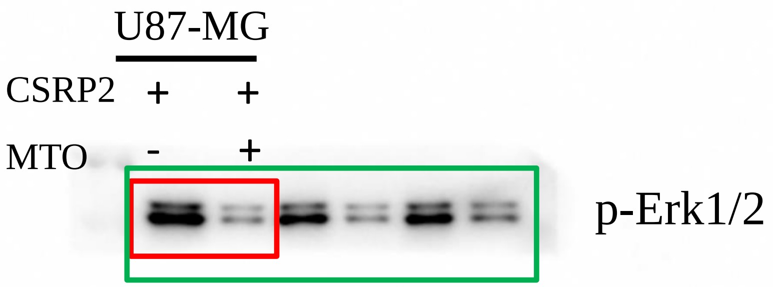

The membrane was imaged with Azure Biosystems 300

Full unedited gel for Figure 8M

Green： Statistical graph

Red： Representative graph

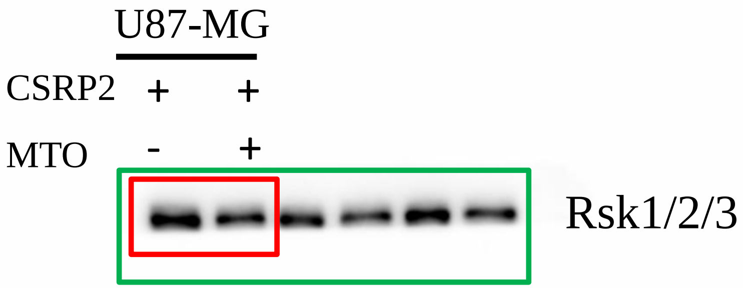

The membrane was imaged with Azure Biosystems 300

Full unedited gel for Figure 8M

Green： Statistical graph

Red： Representative graph

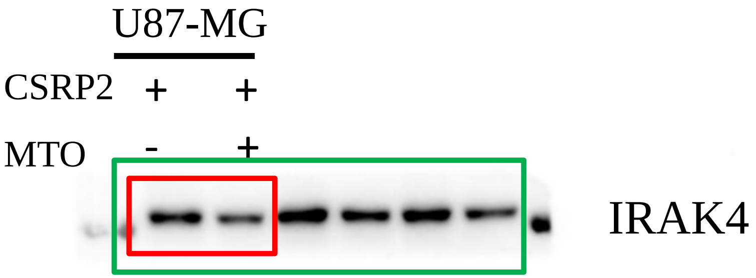

The membrane was imaged with Azure Biosystems 300

Full unedited gel for Figure 8M

Green： Statistical graph

Red： Representative graph

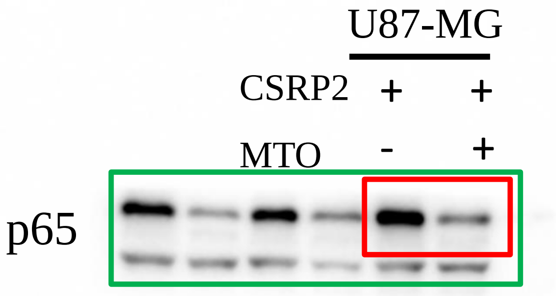

The membrane was imaged with Azure Biosystems 300

Full unedited gel for Figure 8M

Green： Statistical graph

Red： Representative graph

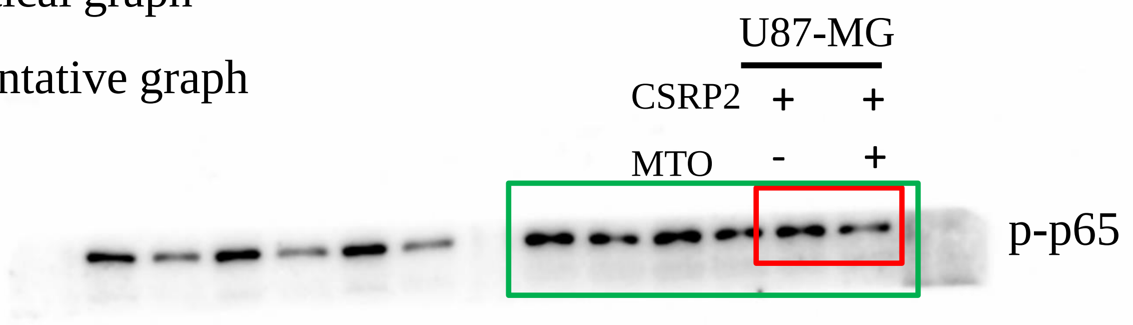

The membrane was imaged with Azure Biosystems 300

Full unedited gel for Figure 8M

Green：Statistical graph

Red：Representative graph

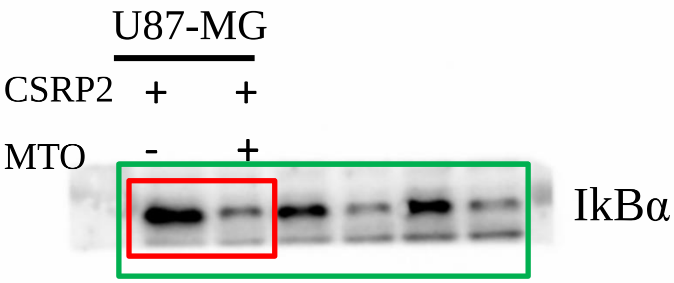

The membrane was imaged with Azure Biosystems 300

Full unedited gel for Figure 8M

Green： Statistical graph

Red： Representative graph

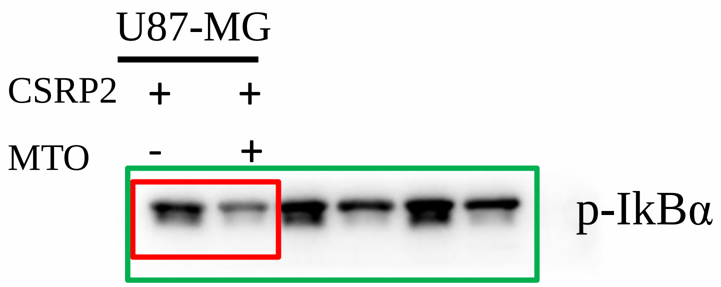

The membrane was imaged with Azure Biosystems 300

Full unedited gel for Figure 8M

Green: Statistical graph

Red: Representative graph

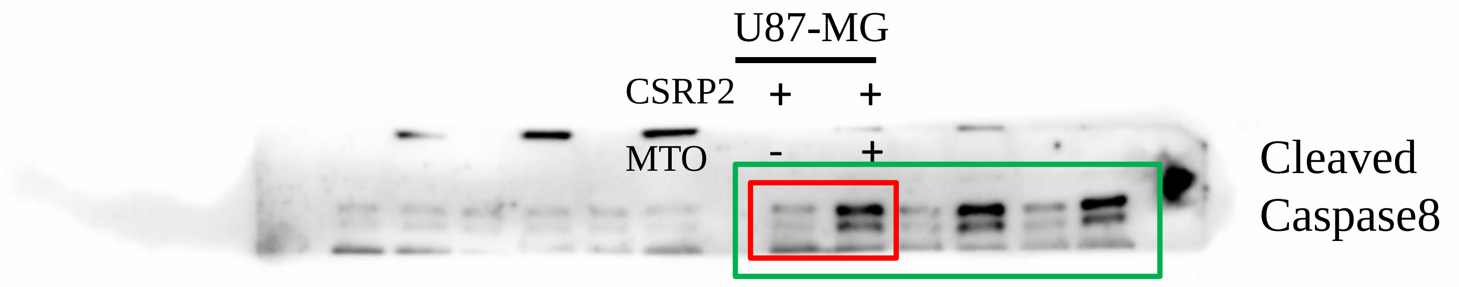

The membrane was imaged with Azure Biosystems 300

Full unedited gel for Figure 8M

Green： Statistical graph

Red： Representative graph

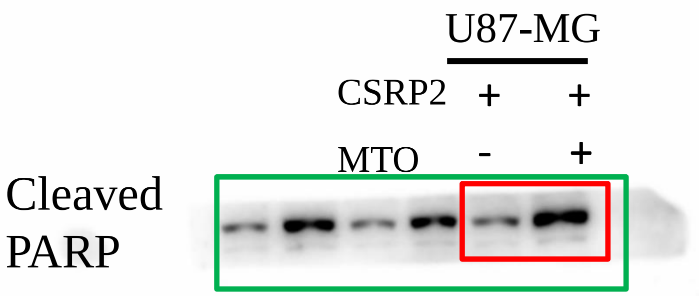

The membrane was imaged with Azure Biosystems 300

Full unedited gel for Figure 8M

Green： Statistical graph

Red： Representative graph

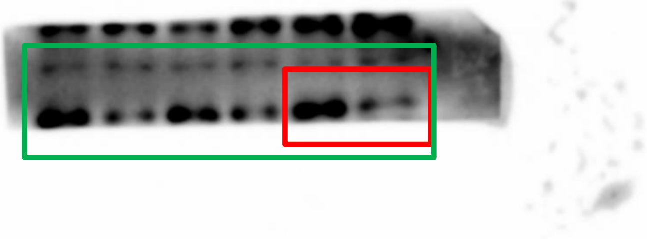

The membrane was imaged with Azure Biosystems 300

Full unedited gel for Figure 8M

Green： Statistical graph

Red： Representative graph

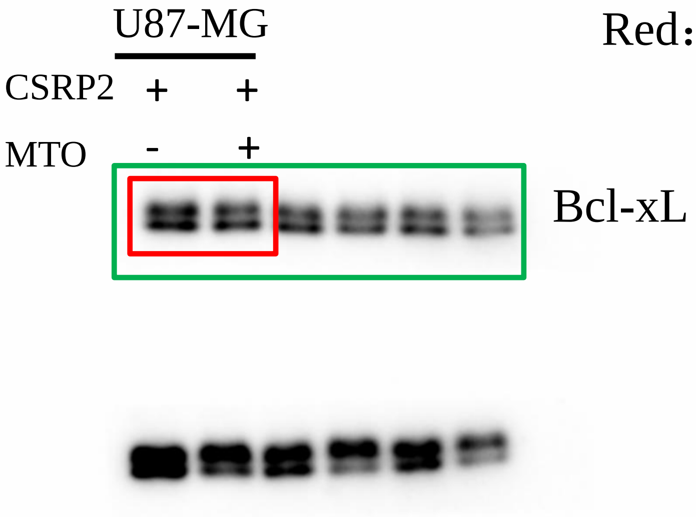

The membrane was imaged with Azure Biosystems 300

Full unedited gel for Figure 8M

Green: Statistical graph

Red: Representative graph

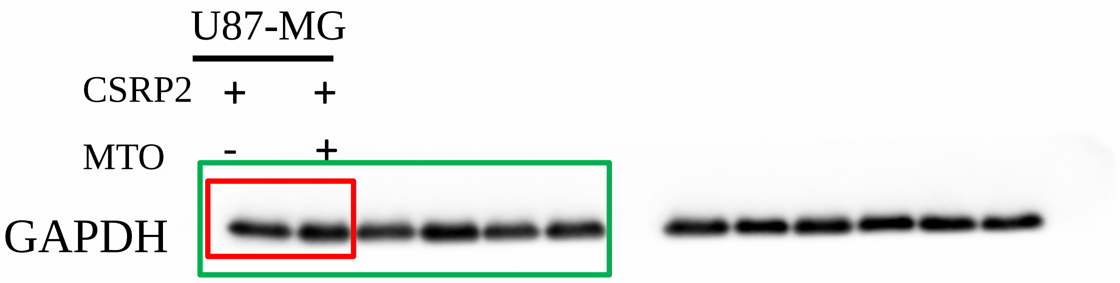

The membrane was imaged with Azure Biosystems 300

Full unedited gel for Figure 8M

Green： Statistical graph

Red： Representative graph

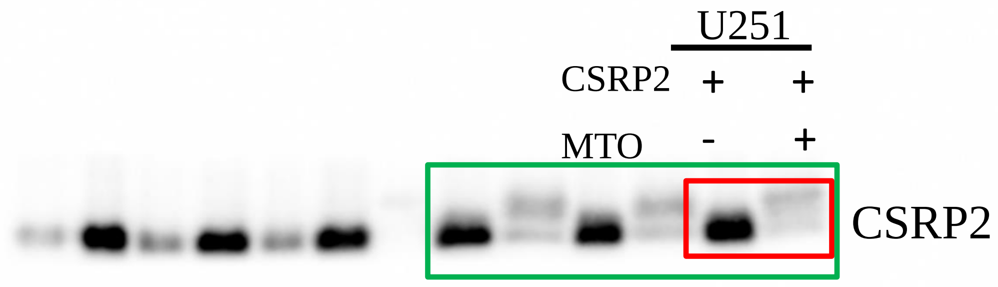

The membrane was imaged with Azure Biosystems 300

Full unedited gel for Figure 8M

Green： Statistical graph

Red： Representative graph

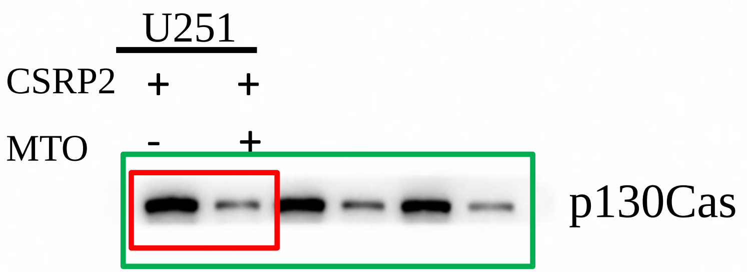

The membrane was imaged with Azure Biosystems 300

Full unedited gel for Figure 8M

Green： Statistical graph

Red： Representative graph

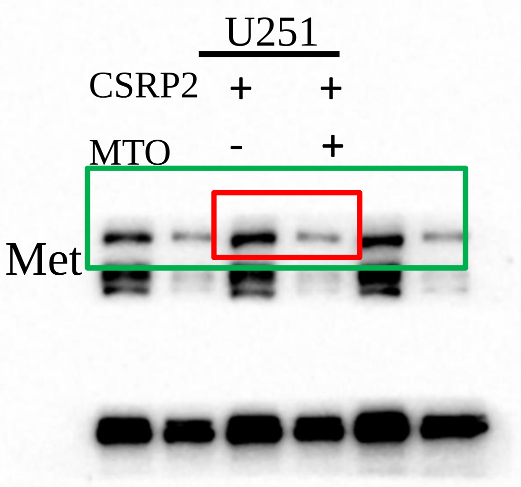

The membrane was imaged with Azure Biosystems 300

Full unedited gel for Figure 8M

Green： Statistical graph

Red： Representative graph

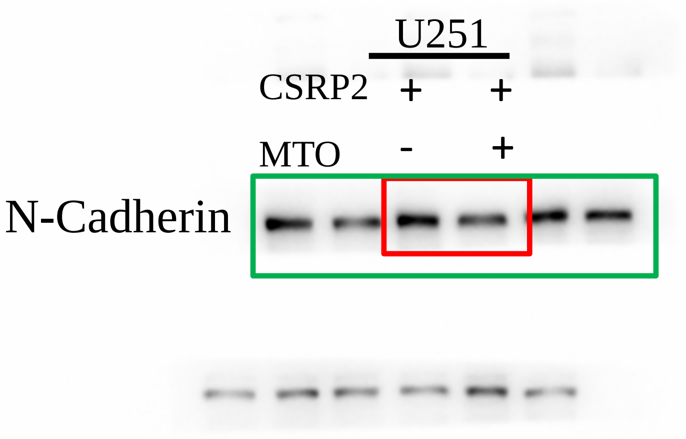

The membrane was imaged with Azure Biosystems 300

Full unedited gel for Figure 8M

Green： Statistical graph

Red： Representative graph

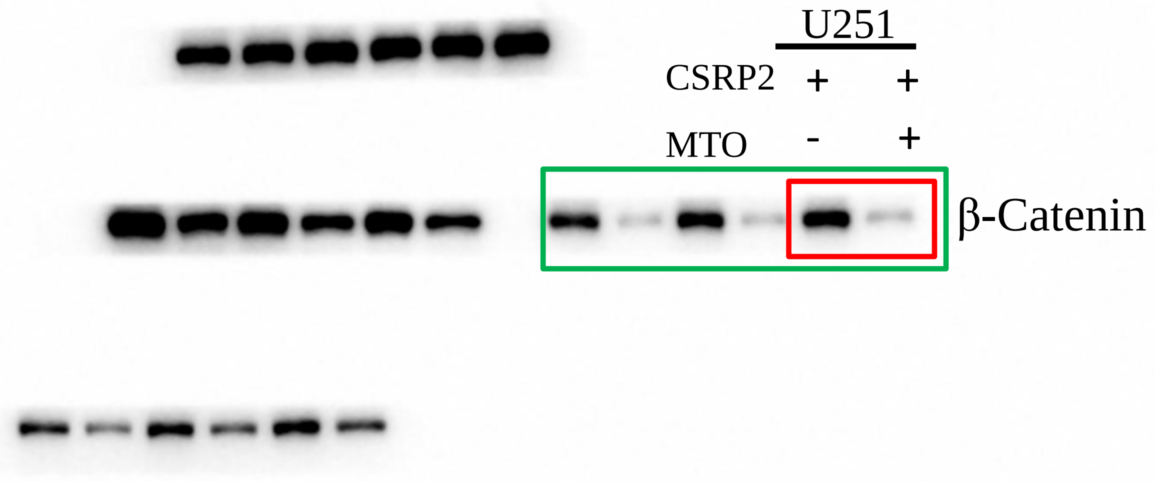

The membrane was imaged with Azure Biosystems 300

Full unedited gel for Figure 8M

Green: Statistical graph

Red: Representative graph

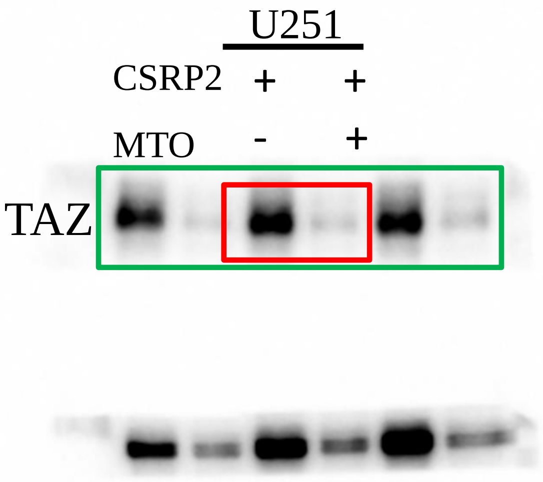

The membrane was imaged with Azure Biosystems 300

Full unedited gel for Figure 8M

Green： Statistical graph

Red： Representative graph

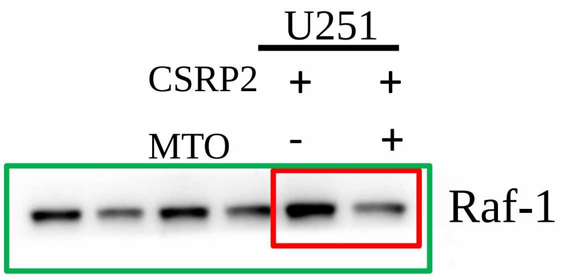

The membrane was imaged with Azure Biosystems 300

Full unedited gel for Figure 8M

Green： Statistical graph

Red： Representative graph

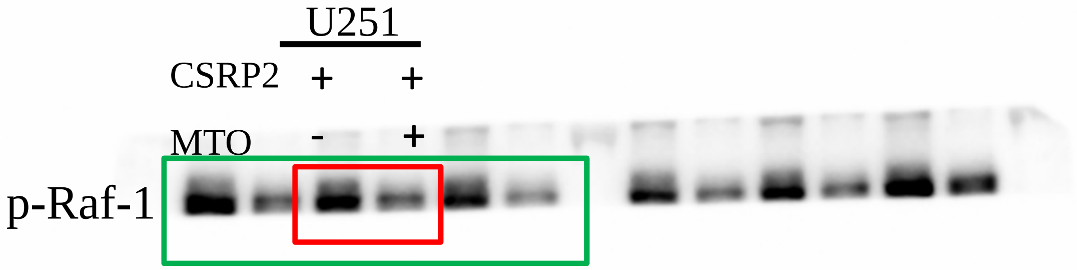

The membrane was imaged with Azure Biosystems 300

Full unedited gel for Figure 8M

Green: Statistical graph

Red: Representative graph

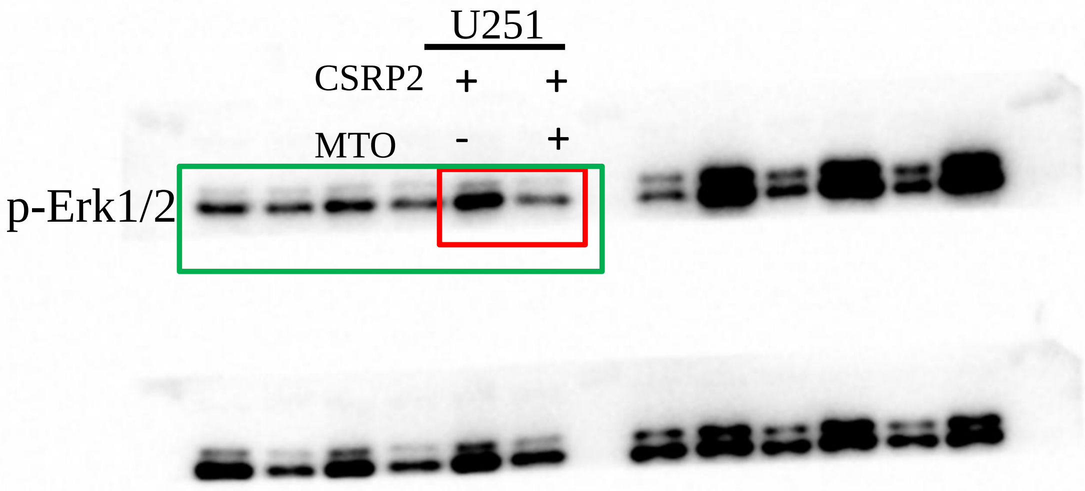

The membrane was imaged with Azure Biosystems 300

Full unedited gel for Figure 8M

Green： Statistical graph

Red： Representative graph

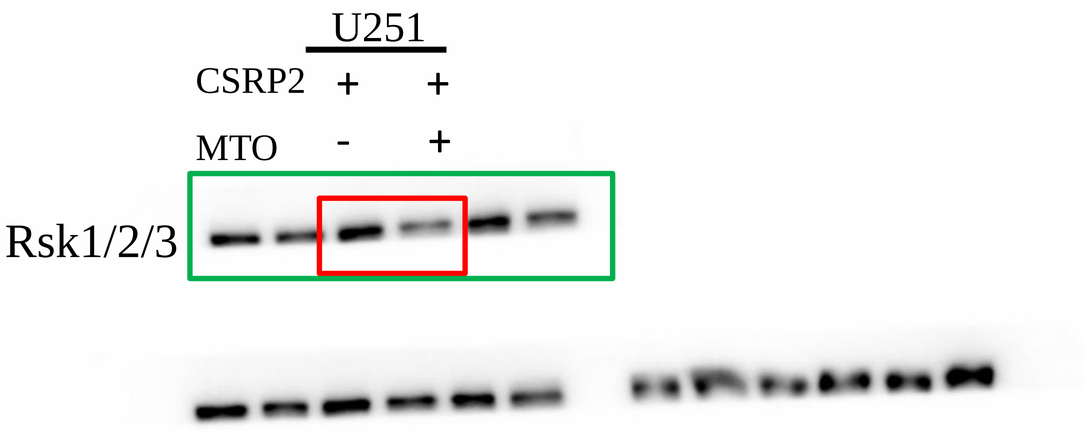

The membrane was imaged with Azure Biosystems 300

Full unedited gel for Figure 8M

Green： Statistical graph

Red： Representative graph

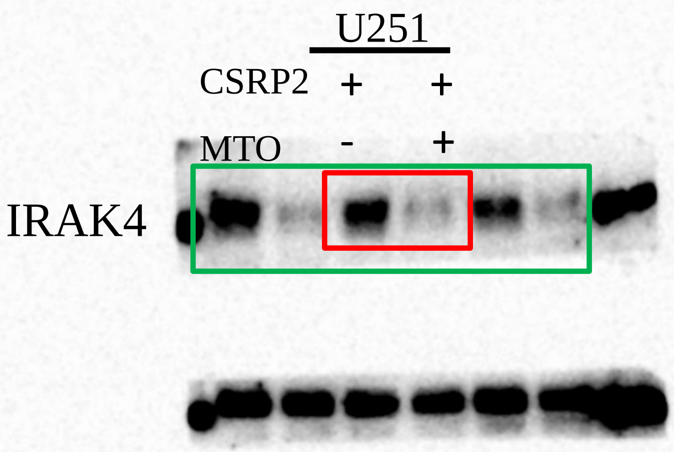

The membrane was imaged with Azure Biosystems 300

Full unedited gel for Figure 8M

Green： Statistical graph

Red： Representative graph

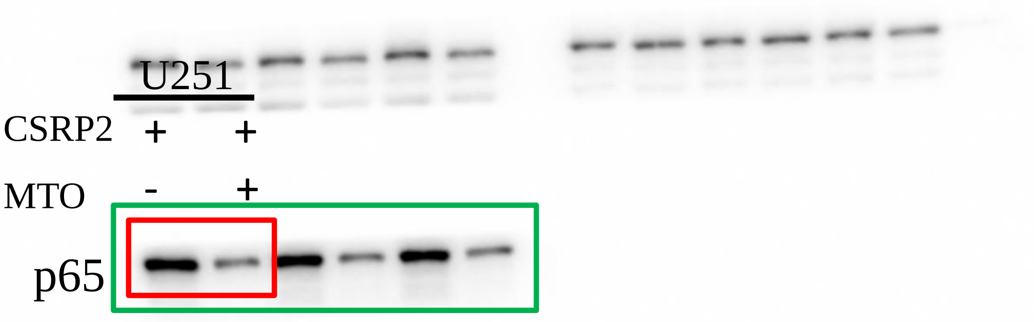

The membrane was imaged with Azure Biosystems 300

Full unedited gel for Figure 8M

Green： Statistical graph

Red： Representative graph

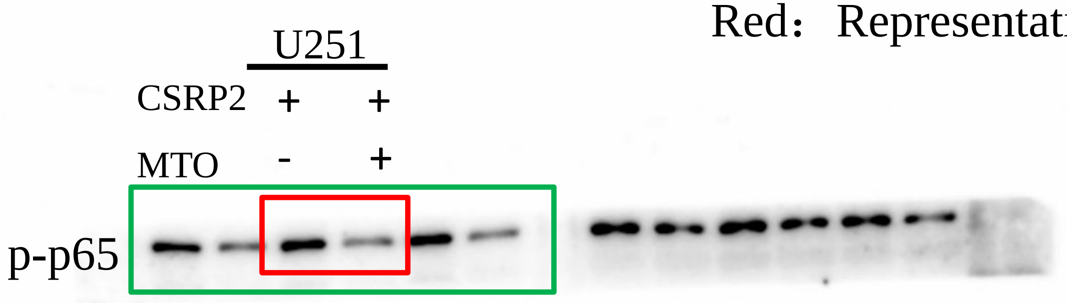

The membrane was imaged with Azure Biosystems 300

Full unedited gel for Figure 8M

Green： Statistical graph

Red： Representative graph

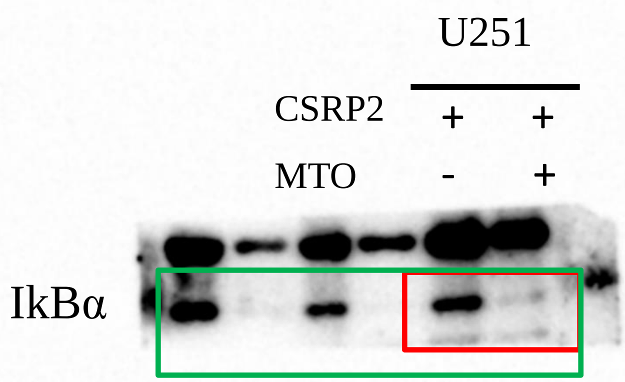

The membrane was imaged with Azure Biosystems 300

Full unedited gel for Figure 8M

Green: Statistical graph

Red: Representative graph

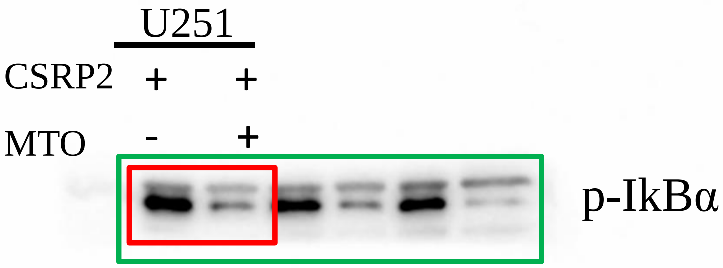

The membrane was imaged with Azure Biosystems 300

Full unedited gel for Figure 8M

Green： Statistical graph

Red： Representative graph

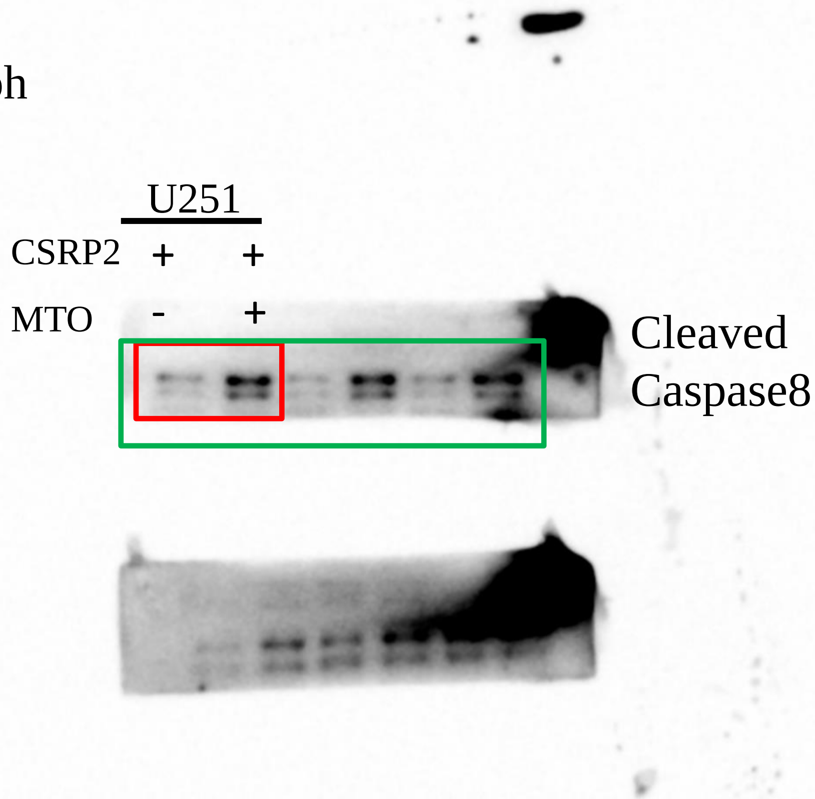

The membrane was imaged with Azure Biosystems 300

Full unedited gel for Figure 8M

Green： Statistical graph

Red： Representative graph

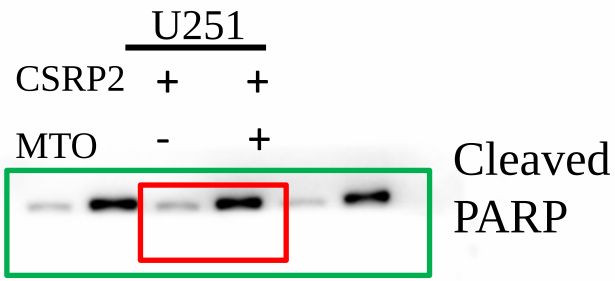

The membrane was imaged with Azure Biosystems 300

Full unedited gel for Figure 8M

Green: Statistical graph

Red: Representative graph

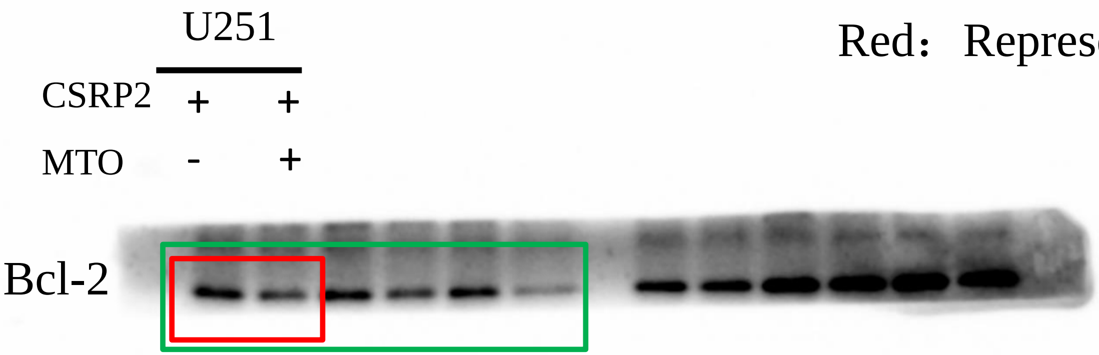

The membrane was imaged with Azure Biosystems 300

Full unedited gel for Figure 8M

Green： Statistical graph

Red： Representative graph

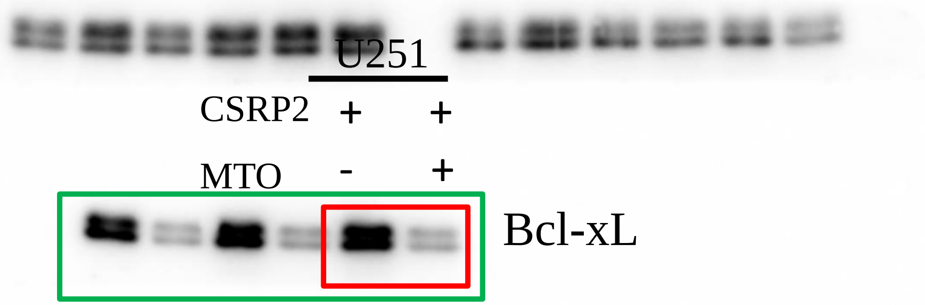

The membrane was imaged with Azure Biosystems 300

Full unedited gel for Figure 8M

Green： Statistical graph

Red： Representative graph

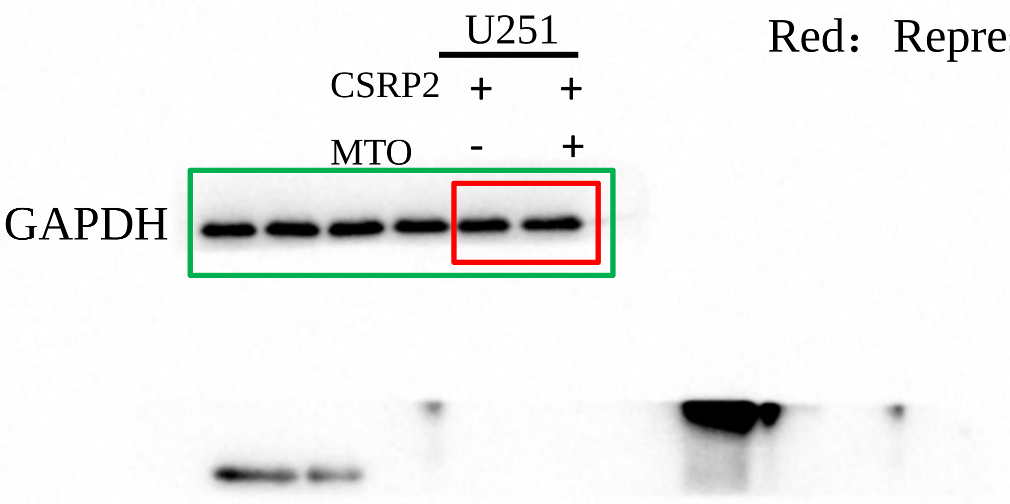

The membrane was imaged with Azure Biosystems 300

Full unedited gel for Figure Supplement 3C

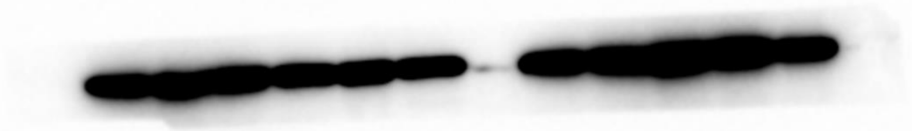

Red： Representative graph

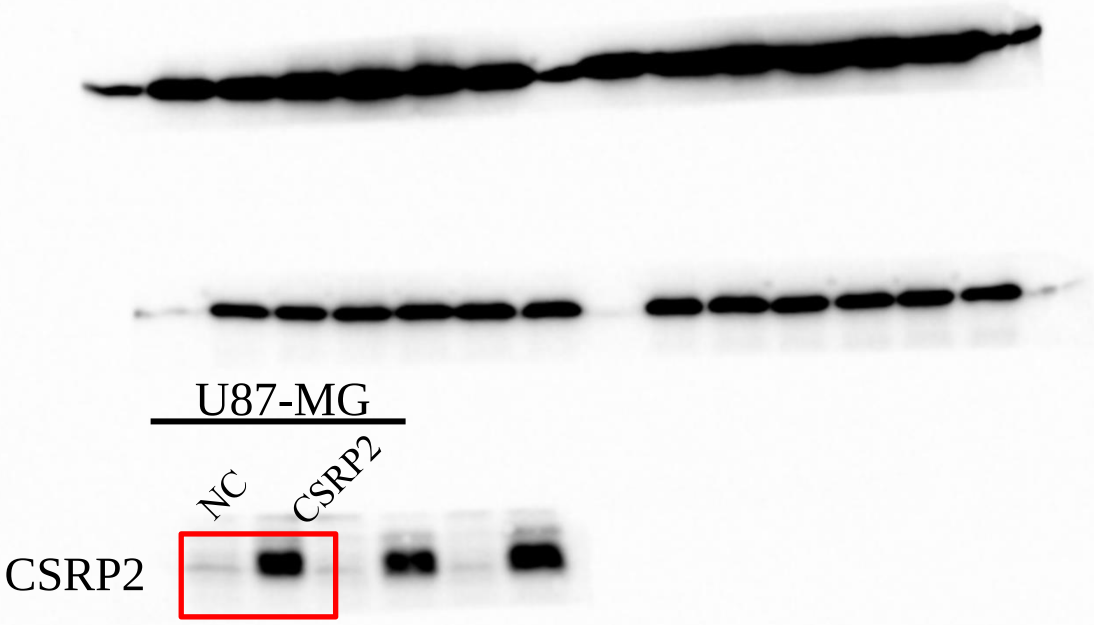

The membrane was imaged with Azure Biosystems 300

Full unedited gel for Figure Supplement 3C

Green： Statistical graph

Red： Representative graph

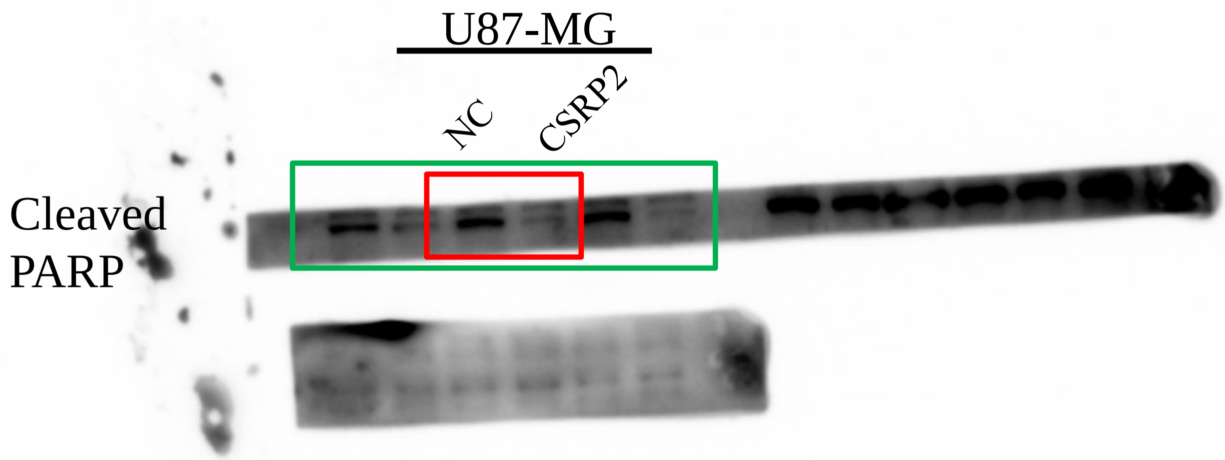

The membrane was imaged with Azure Biosystems 300

Full unedited gel for Figure Supplement 3C

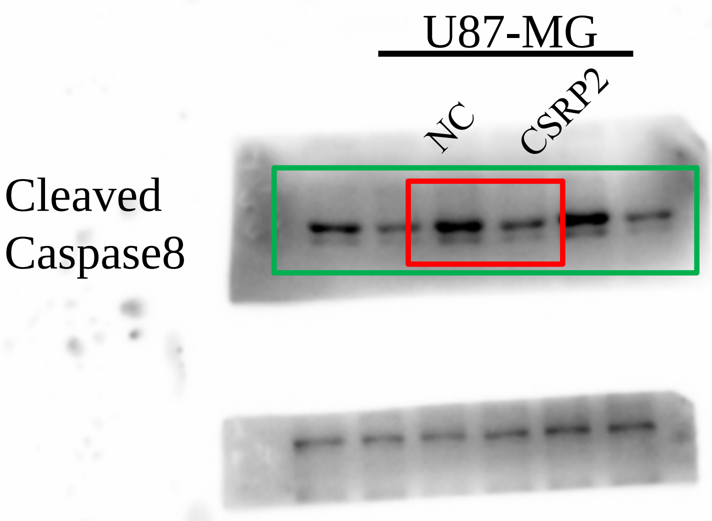

Green： Statistical graph

Red： Representative graph

The membrane was imaged with Azure Biosystems 300

Full unedited gel for Figure Supplement 3C

Green： Statistical graph

Red： Representative graph

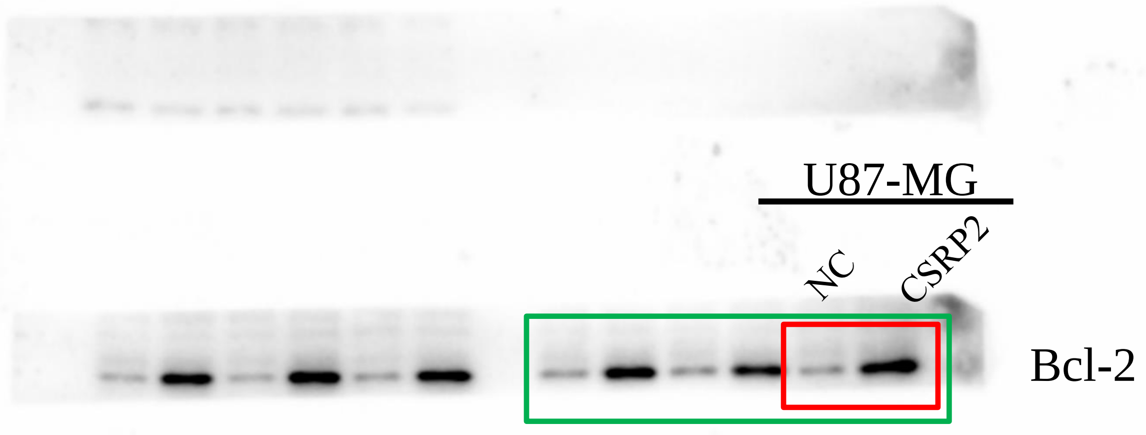

The membrane was imaged with Azure Biosystems 300

Full unedited gel for Figure Supplement 3C

Green： Statistical graph

Red： Representative graph

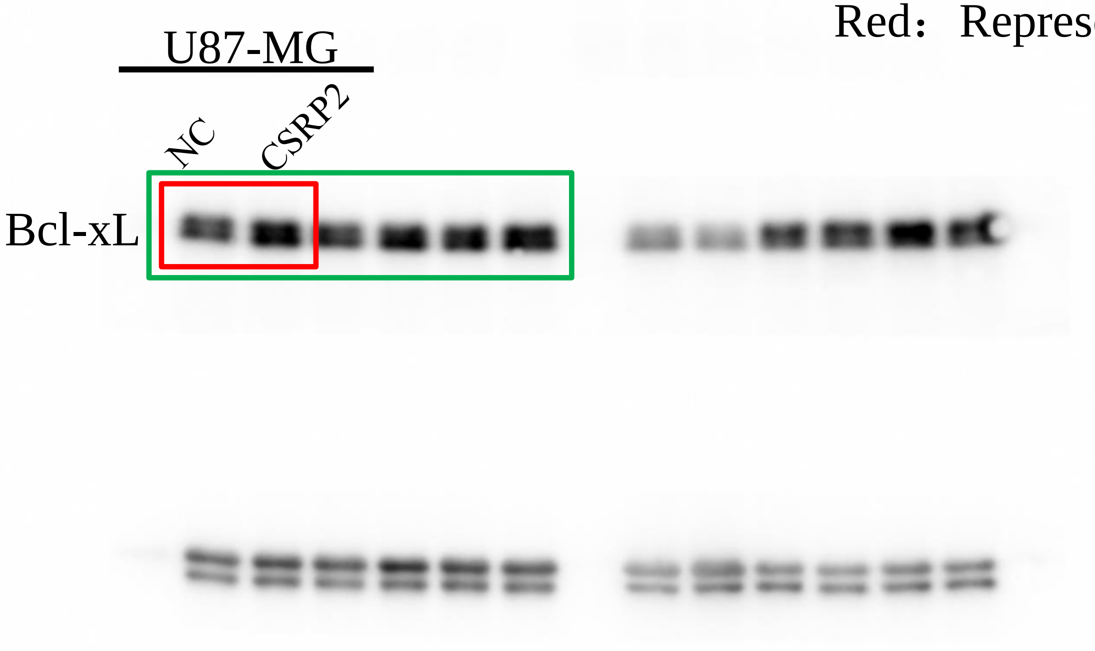

The membrane was imaged with Azure Biosystems 300

Full unedited gel for Figure Supplement 3C

Red： Representative graph

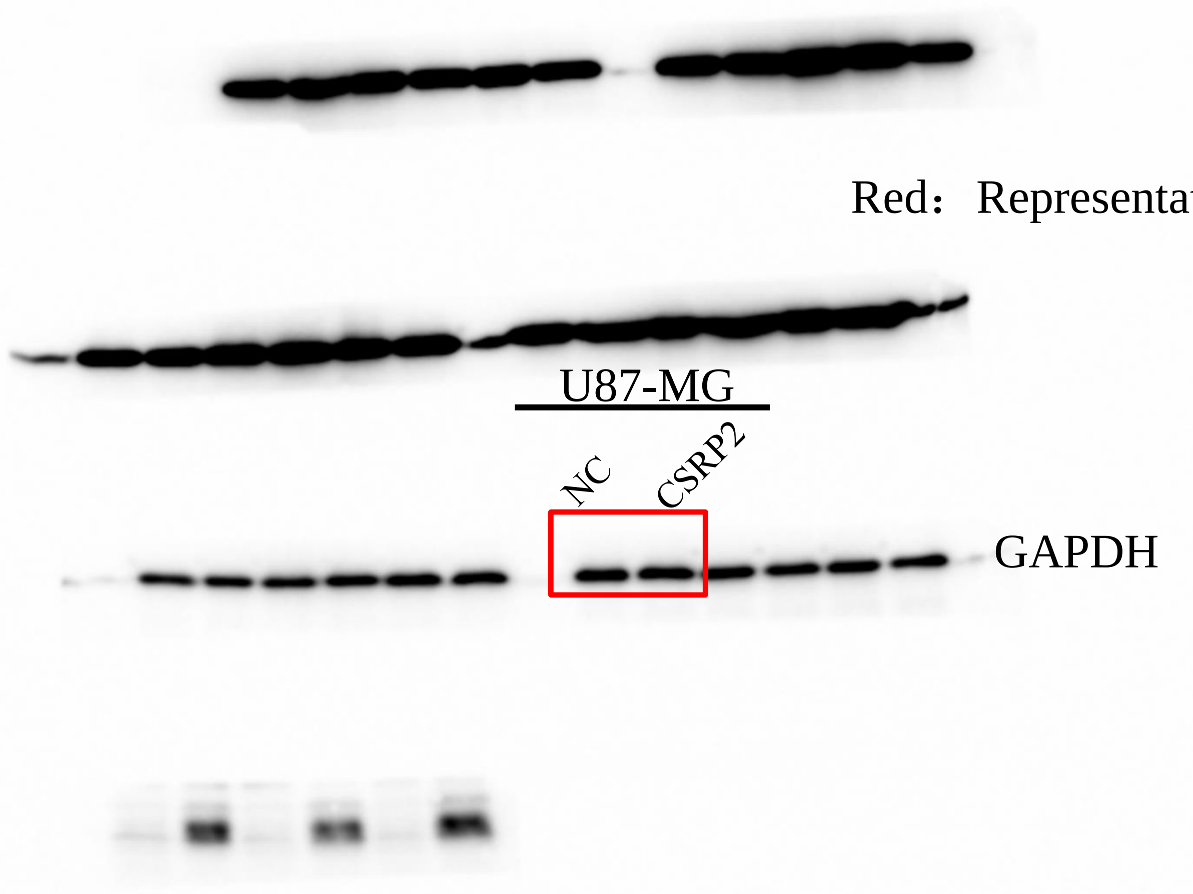

The membrane was imaged with Azure Biosystems 300

Full unedited gel for Figure Supplement 3C

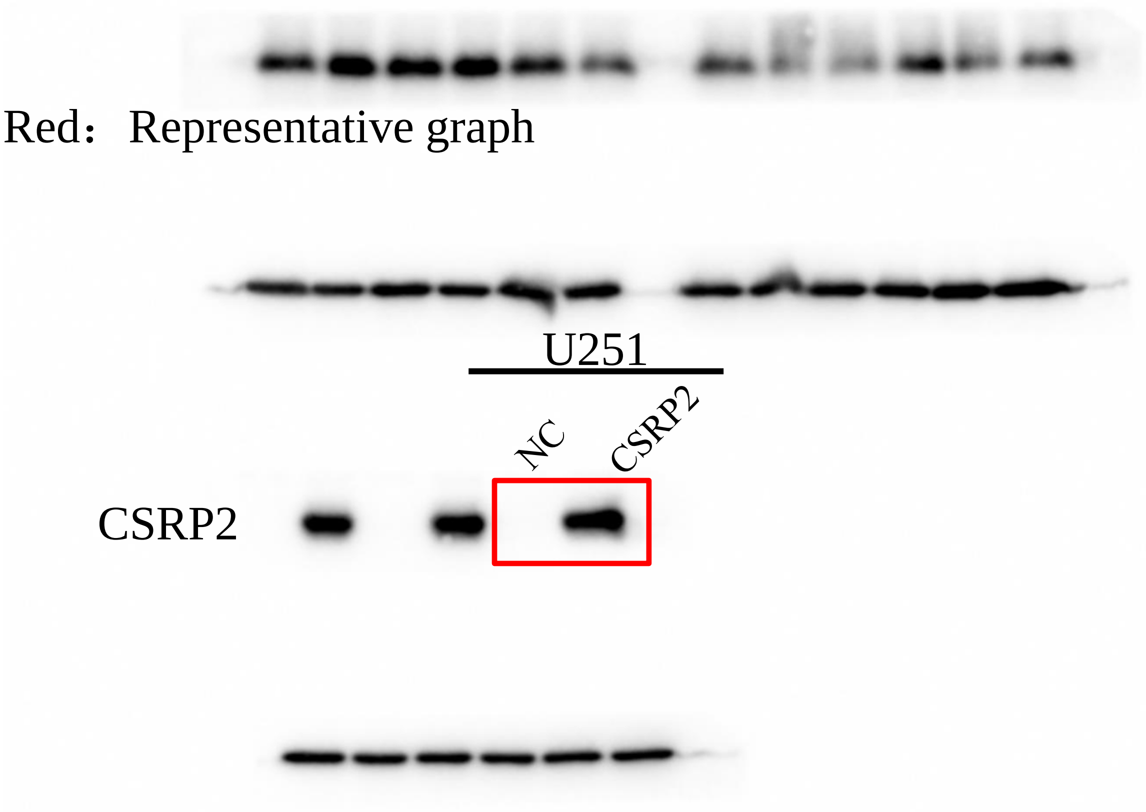

The membrane was imaged with Azure Biosystems 300

Full unedited gel for Figure Supplement 3C

Green： Statistical graph

Red： Representative graph

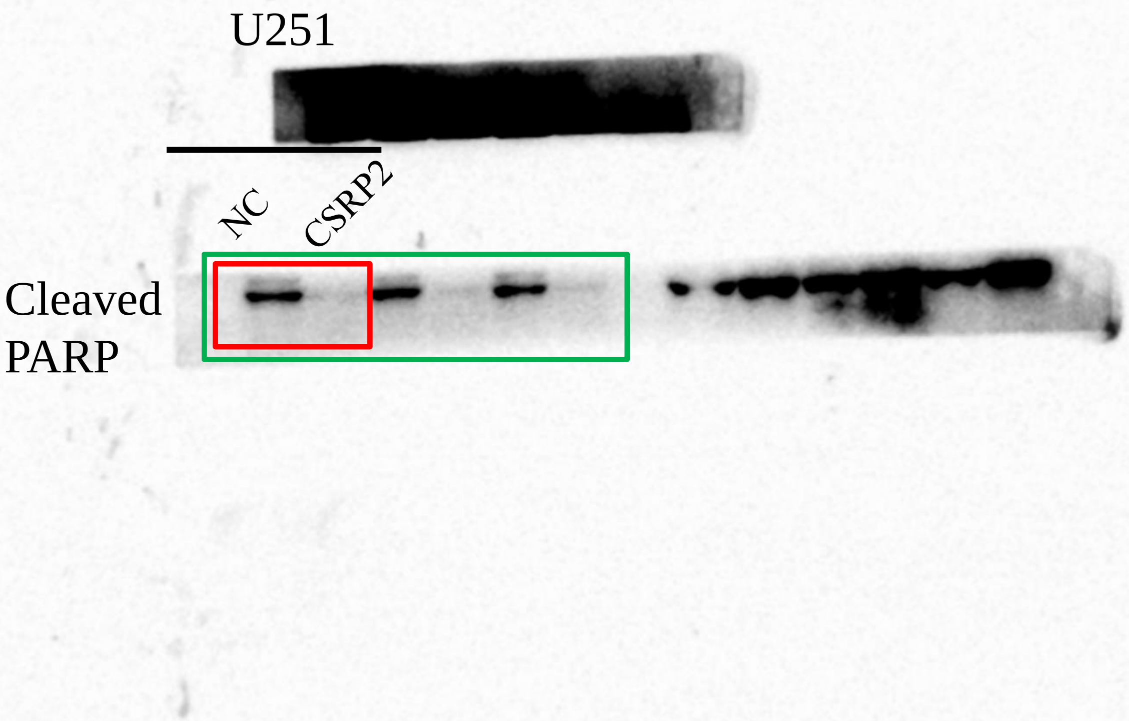

The membrane was imaged with Azure Biosystems 300

Full unedited gel for Figure Supplement 3C

Green： Statistical graph

Red： Representative graph

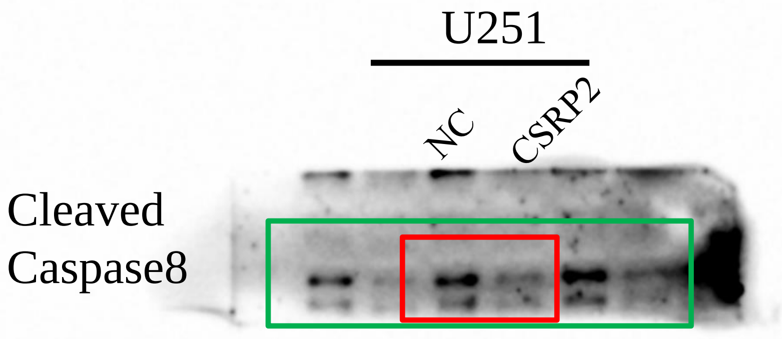

The membrane was imaged with Azure Biosystems 300

Full unedited gel for Figure Supplement 3C

Green： Statistical graph

Red： Representative graph

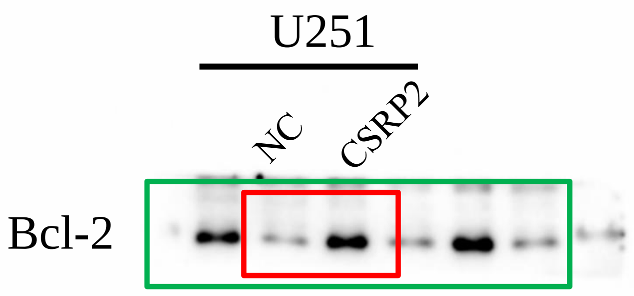

The membrane was imaged with Azure Biosystems 300

Full unedited gel for Figure Supplement 3C

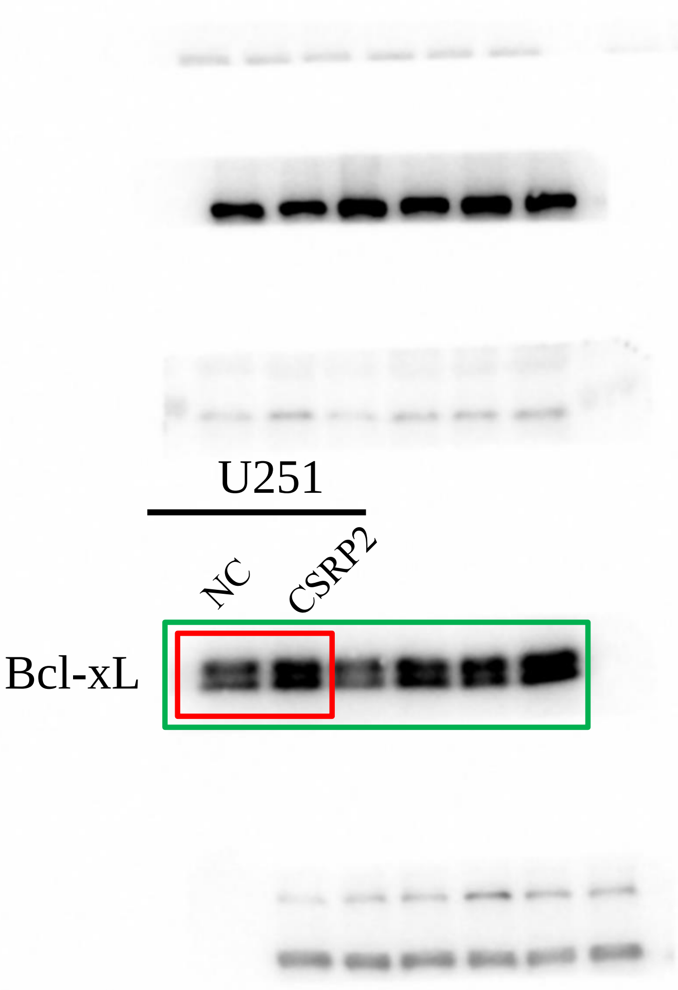

Green： Statistical graph

Red： Representative graph

The membrane was imaged with Azure Biosystems 300

Full unedited gel for Figure Supplement 3C

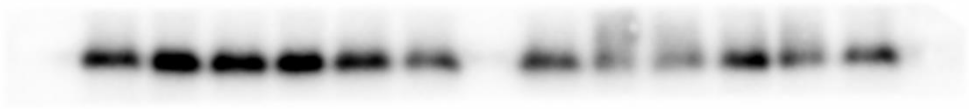

Red： Representative graph

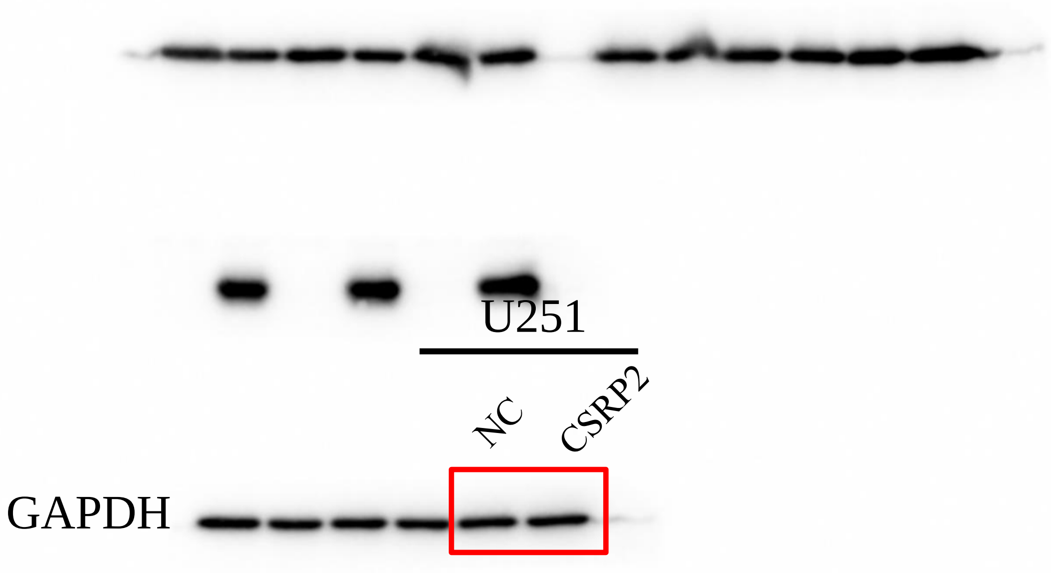

The membrane was imaged with Azure Biosystems 300

Full unedited gel for Figure Supplement 4C

Red： Representative graph

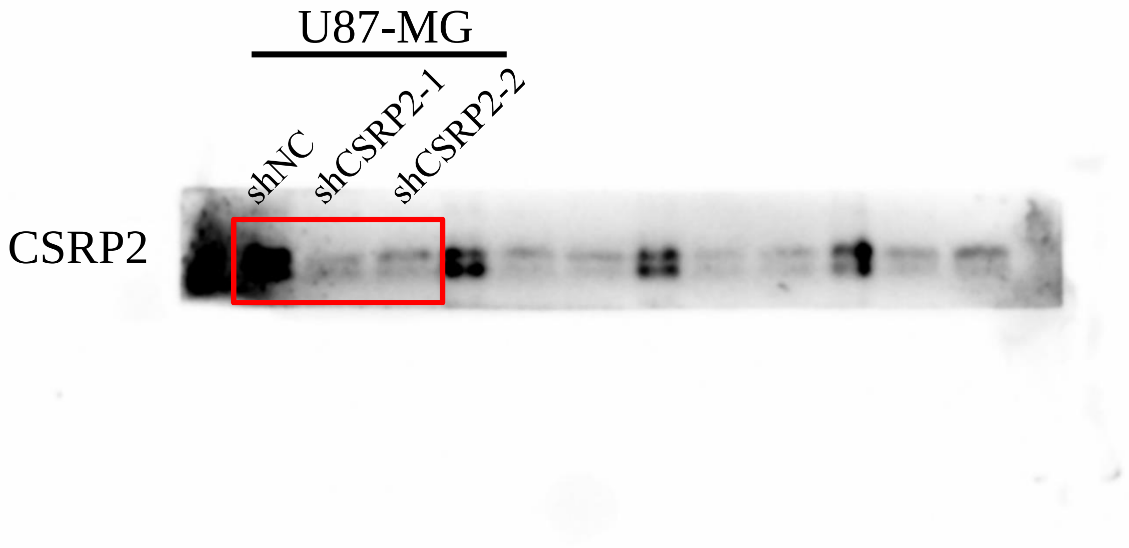

The membrane was imaged with Azure Biosystems 300

Full unedited gel for Figure Supplement 4C

Green： Statistical graph

Red： Representative graph

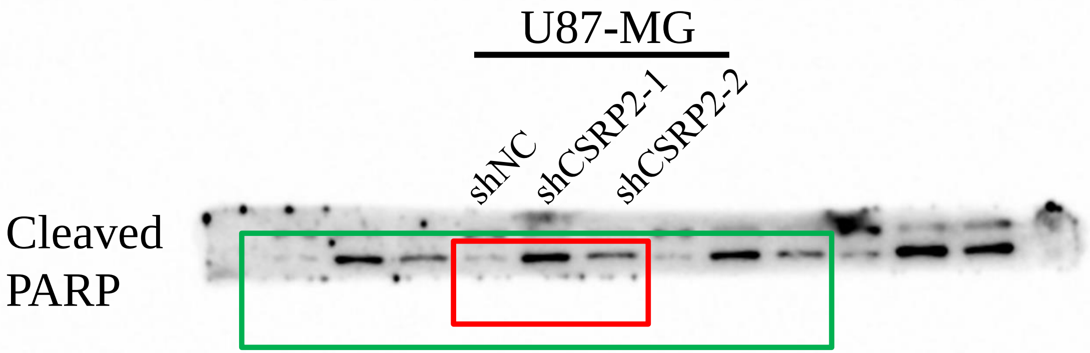

The membrane was imaged with Azure Biosystems 300

Full unedited gel for Figure Supplement 4C

Green： Statistical graph

Red： Representative graph

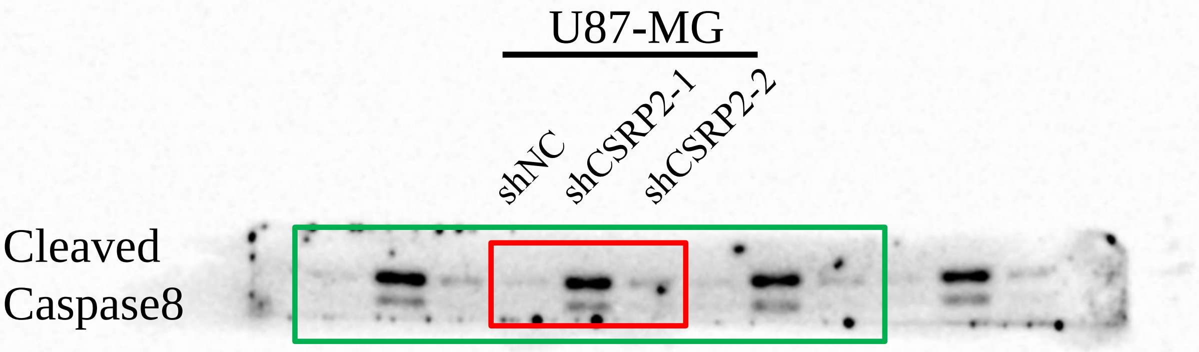

The membrane was imaged with Azure Biosystems 300

Full unedited gel for Figure Supplement 4C

Green： Statistical graph

Red： Representative graph

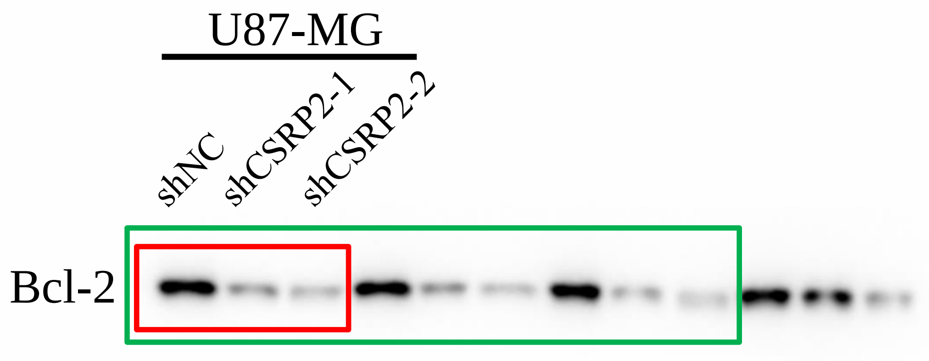

The membrane was imaged with Azure Biosystems 300

Full unedited gel for Figure Supplement 4C

Green： Statistical graph

Red： Representative graph

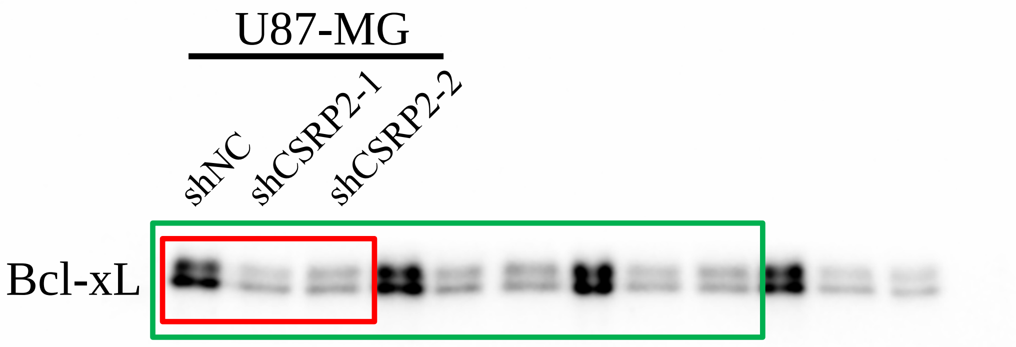

The membrane was imaged with Azure Biosystems 300

Full unedited gel for Figure Supplement 4C

Green： Statistical graph

Red： Representative graph

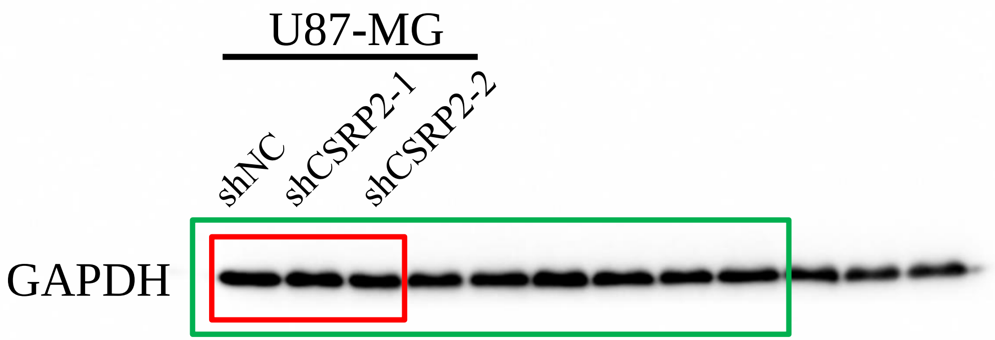

The membrane was imaged with Azure Biosystems 300

Full unedited gel for Figure Supplement 4C

Red： Representative graph

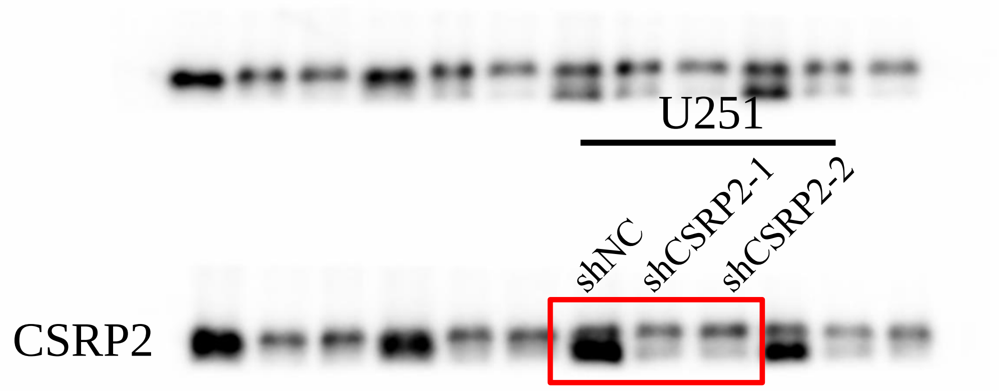

The membrane was imaged with Azure Biosystems 300

Full unedited gel for Figure Supplement 4C

Green: Statistical graph

Red: Representative graph

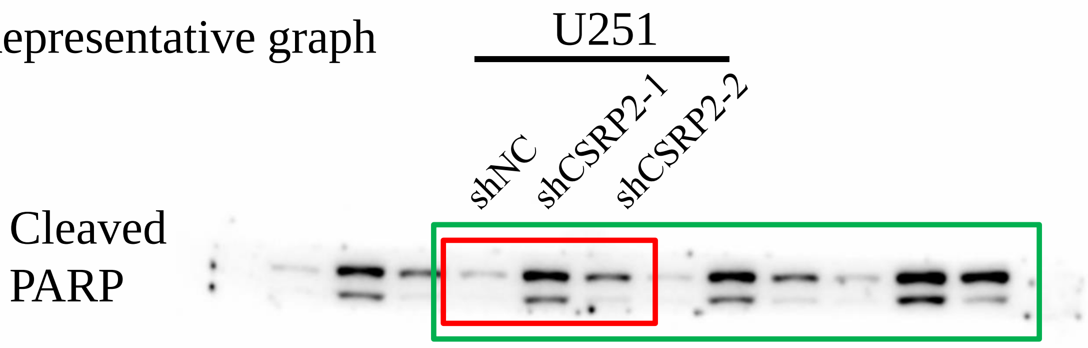

The membrane was imaged with Azure Biosystems 300

Full unedited gel for Figure Supplement 4C

Green： Statistical graph

Red： Representative graph

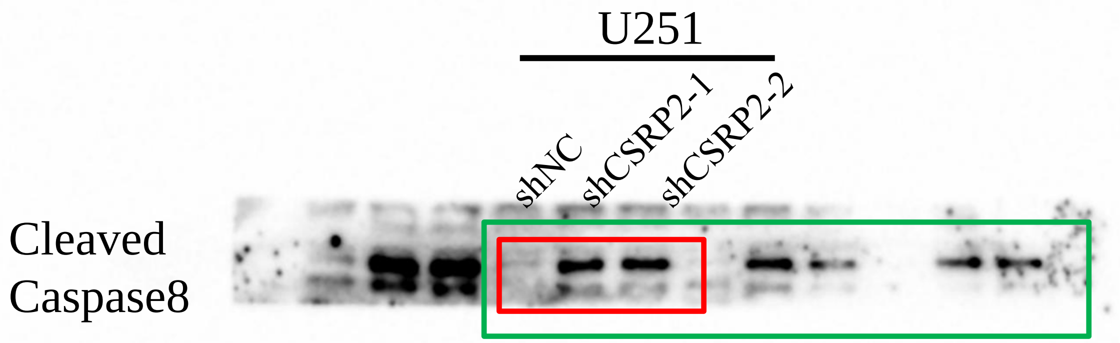

The membrane was imaged with Azure Biosystems 300

Full unedited gel for Figure Supplement 4C

Green： Statistical graph

Red： Representative graph

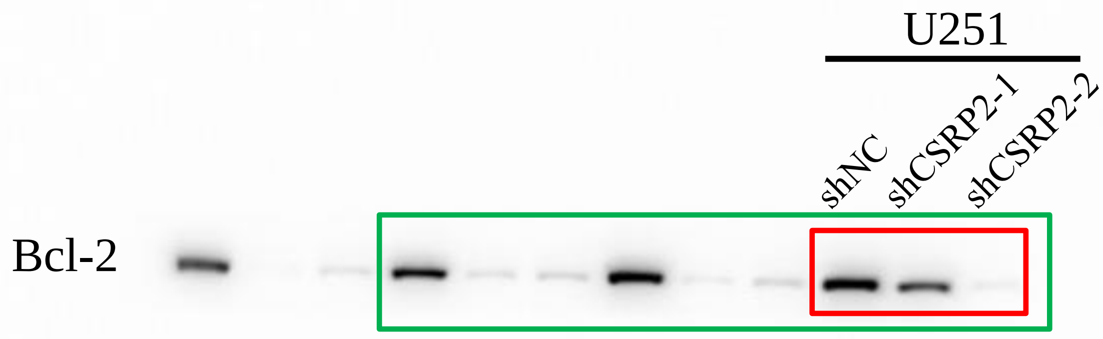

The membrane was imaged with Azure Biosystems 300

Full unedited gel for Figure Supplement 4C

Green: Statistical graph

Red: Representative graph

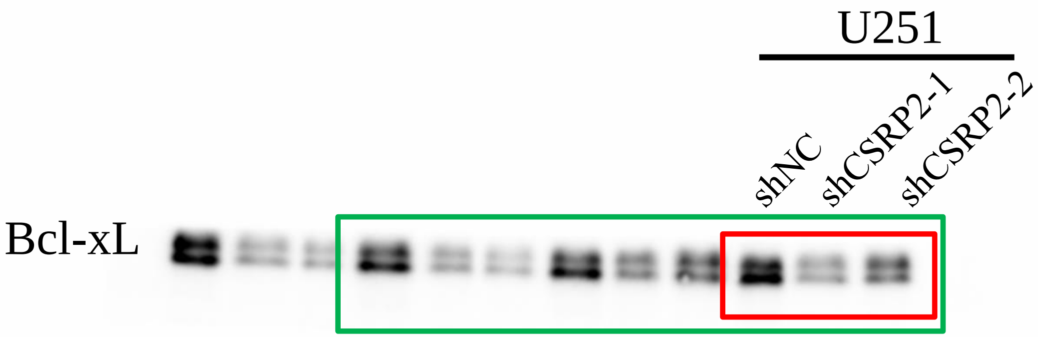

The membrane was imaged with Azure Biosystems 300

Full unedited gel for Figure Supplement 4C

Green： Statistical graph

Red： Representative graph

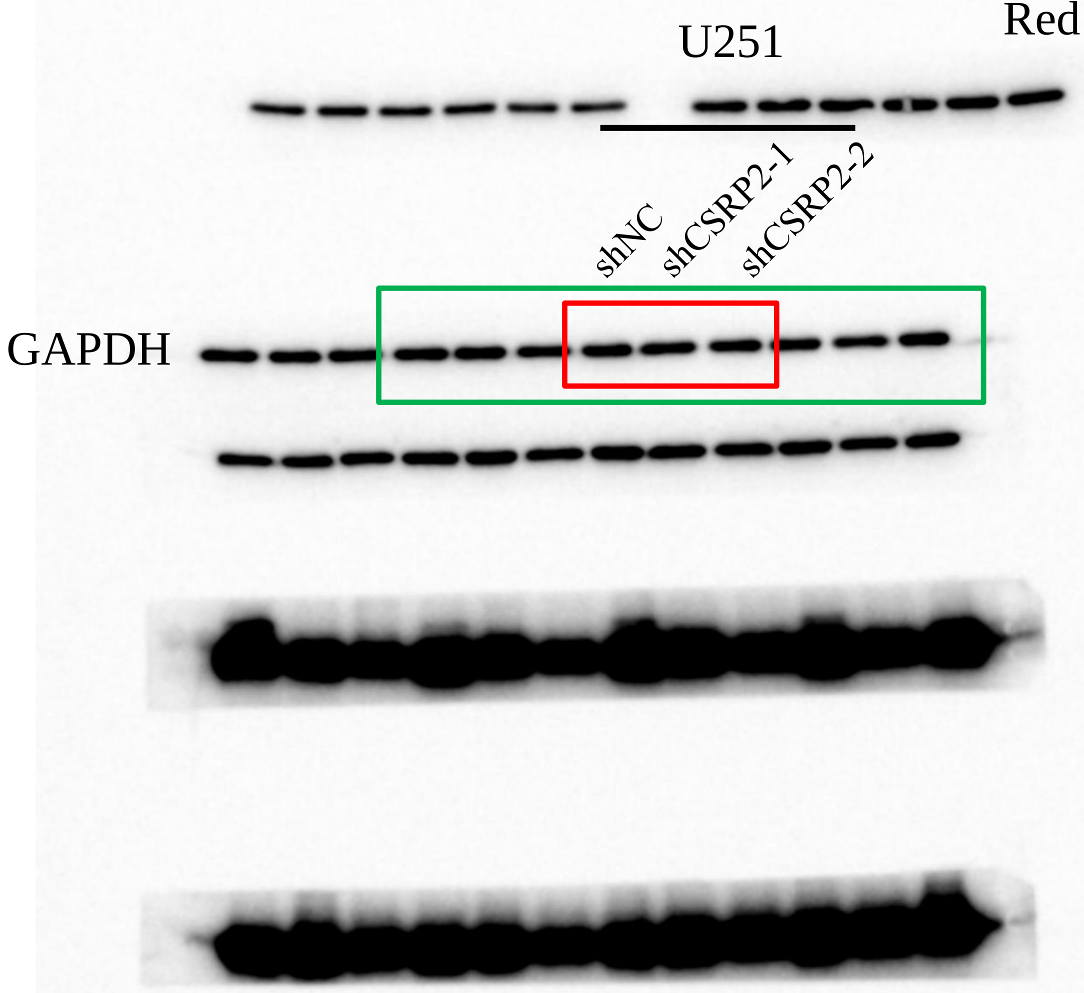

The membrane was imaged with Azure Biosystems 300

Full unedited gel for Figure Supplement 5B

Green： Statistical graph

Red： Representative graph

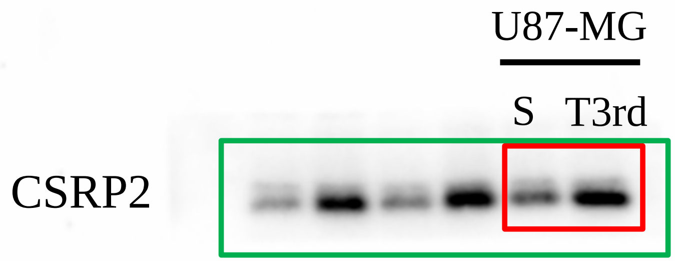

The membrane was imaged with Azure Biosystems 300

Full unedited gel for Figure Supplement 5B

Green： Statistical graph

Red： Representative graph

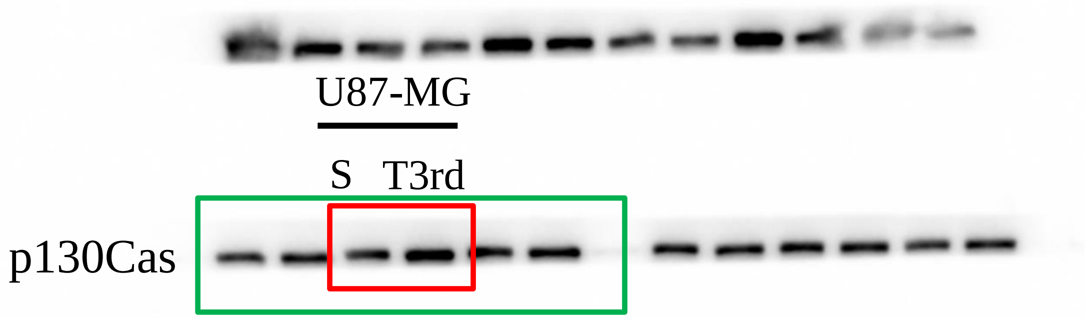

The membrane was imaged with Azure Biosystems 300

Full unedited gel for Figure Supplement 5B

Green： Statistical graph

Red： Representative graph

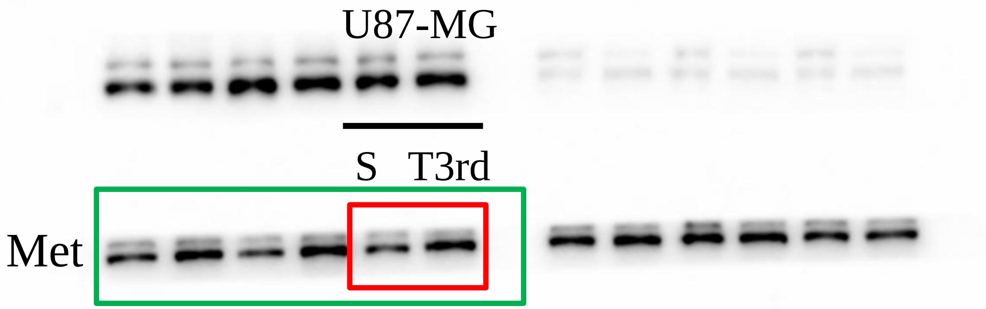

The membrane was imaged with Azure Biosystems 300

Full unedited gel for Figure Supplement 5B

Green： Statistical graph

Red： Representative graph

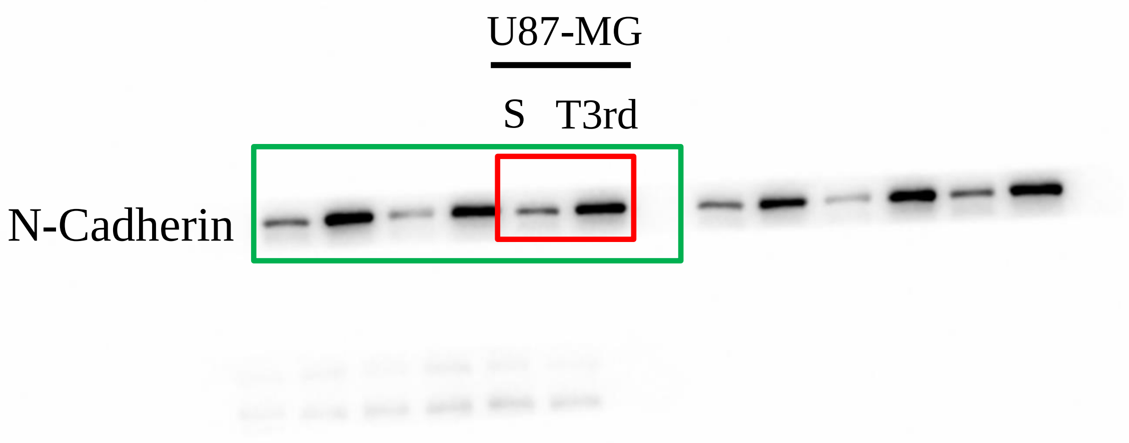

The membrane was imaged with Azure Biosystems 300

Full unedited gel for Figure Supplement 5B

Green: Statistical graph

Red: Representative graph

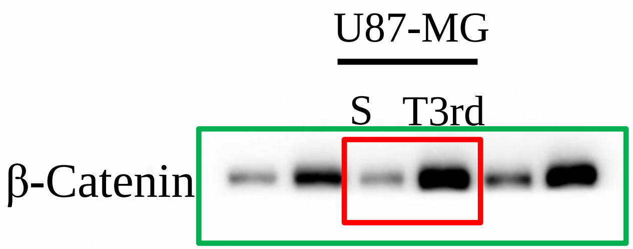

The membrane was imaged with Azure Biosystems 300

Full unedited gel for Figure Supplement 5B

Green： Statistical graph

Red： Representative graph

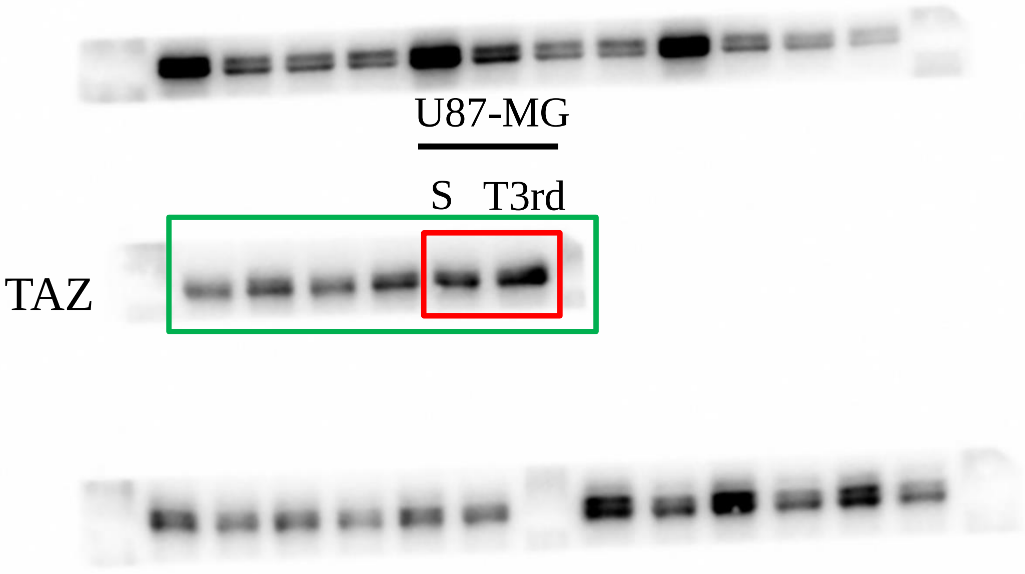

The membrane was imaged with Azure Biosystems 300

Full unedited gel for Figure Supplement 5B

Green： Statistical graph

Red： Representative graph

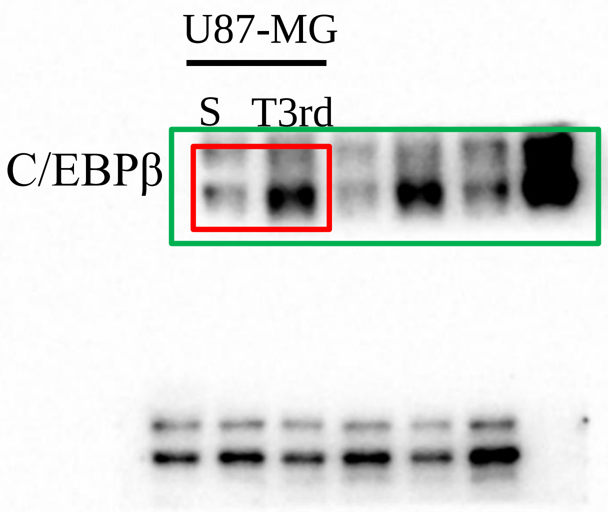

The membrane was imaged with Azure Biosystems 300

Full unedited gel for Figure Supplement 5B

Green： Statistical graph

Red： Representative graph

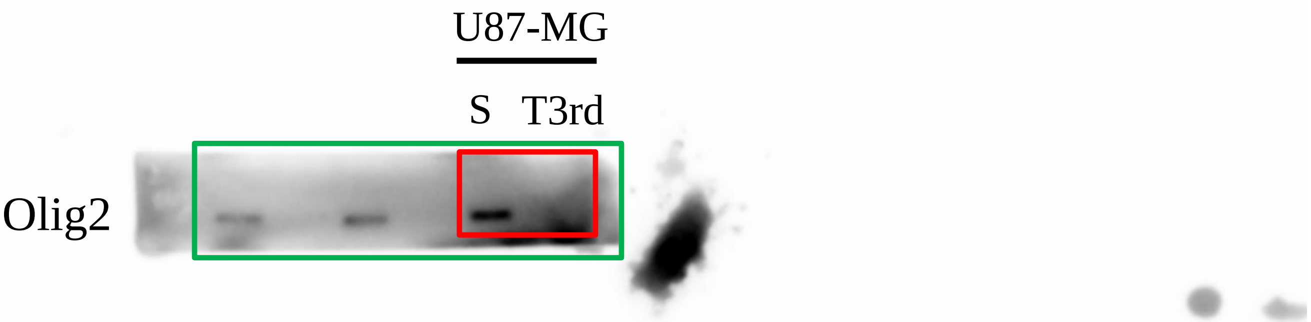

The membrane was imaged with Azure Biosystems 300

Full unedited gel for Figure Supplement 5B

Green： Statistical graph

Red： Representative graph

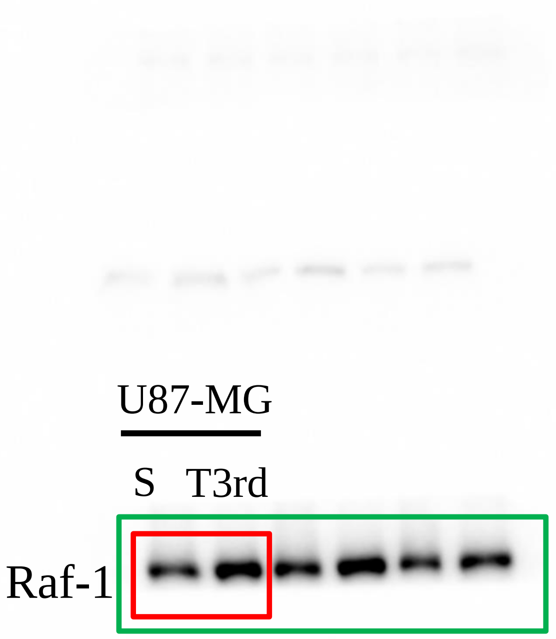

The membrane was imaged with Azure Biosystems 300

Full unedited gel for Figure Supplement 5B

Green： Statistical graph

Red： Representative graph

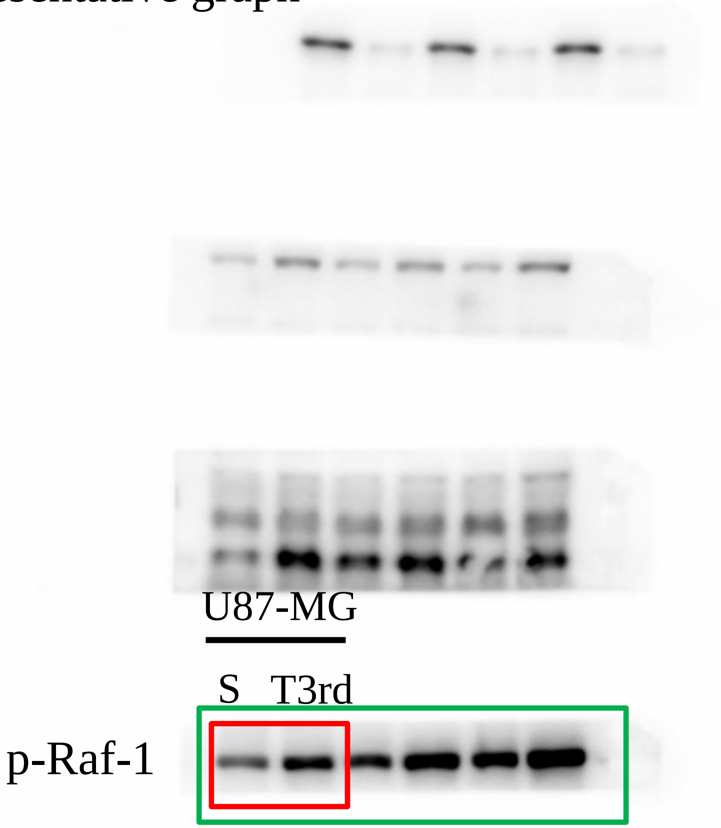

The membrane was imaged with Azure Biosystems 300

Full unedited gel for Figure Supplement 5B

Green： Statistical graph

Red： Representative graph

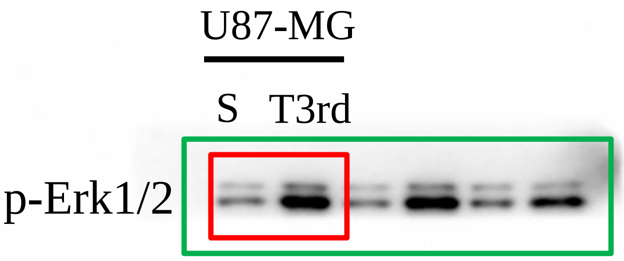

The membrane was imaged with Azure Biosystems 300

Full unedited gel for Figure Supplement 5B

Green： Statistical graph

Red： Representative graph

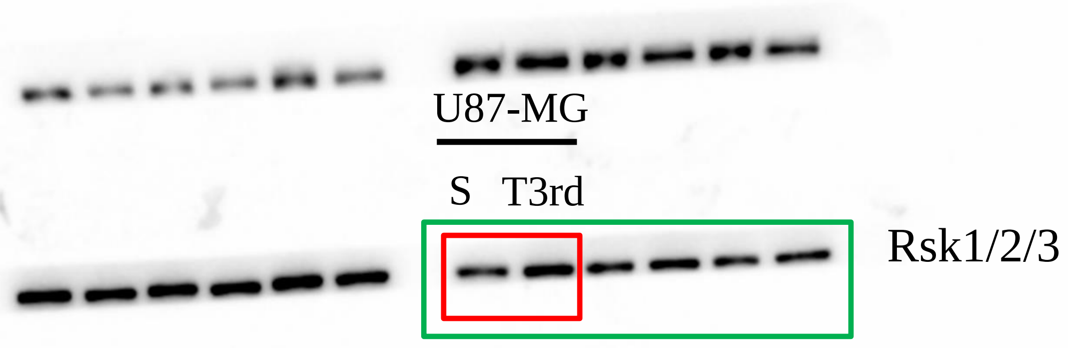

The membrane was imaged with Azure Biosystems 300

Full unedited gel for Figure Supplement 5B

Green： Statistical graph

Red： Representative graph

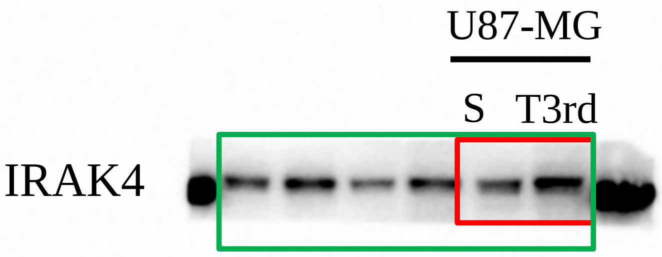

The membrane was imaged with Azure Biosystems 300

Full unedited gel for Figure Supplement 5B

Green： Statistical graph

Red： Representative graph

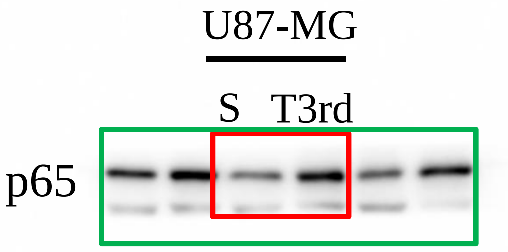

The membrane was imaged with Azure Biosystems 300

Full unedited gel for Figure Supplement 5B

Green： Statistical graph

Red： Representative graph

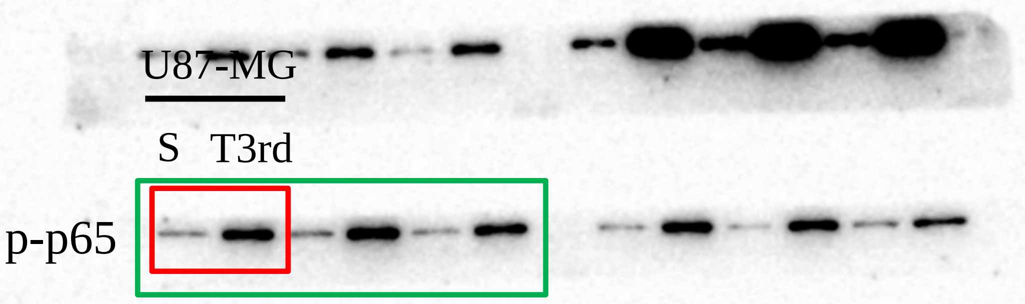

The membrane was imaged with Azure Biosystems 300

Full unedited gel for Figure Supplement 5B

Green: Statistical graph

Red: Representative graph

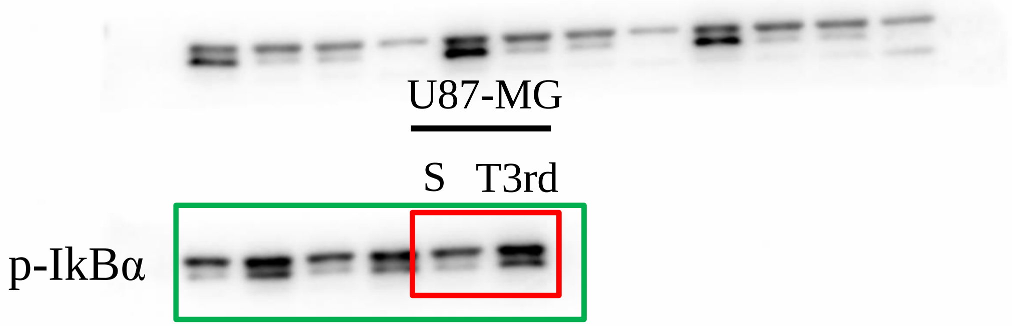

The membrane was imaged with Azure Biosystems 300

Full unedited gel for Figure Supplement 5B

Green： Statistical graph

Red： Representative graph

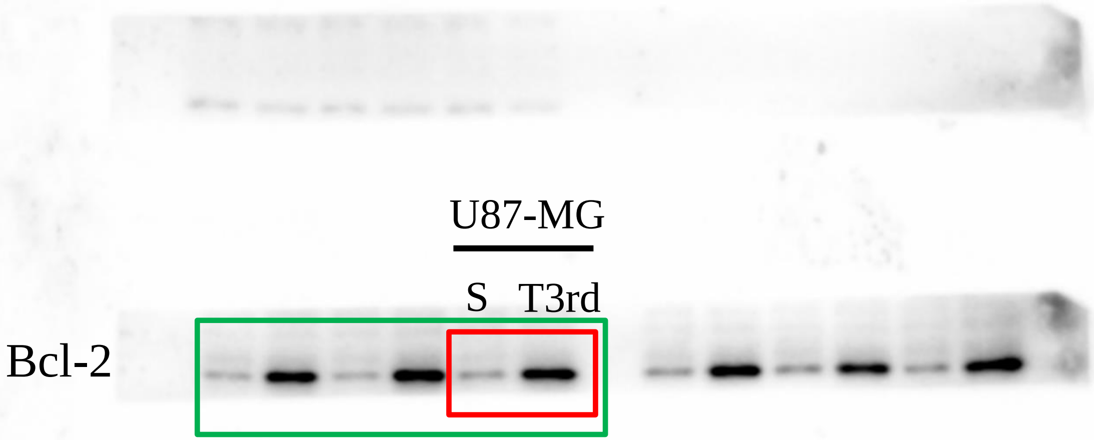

The membrane was imaged with Azure Biosystems 300

Full unedited gel for Figure Supplement 5B

Green： Statistical graph

Red： Representative graph

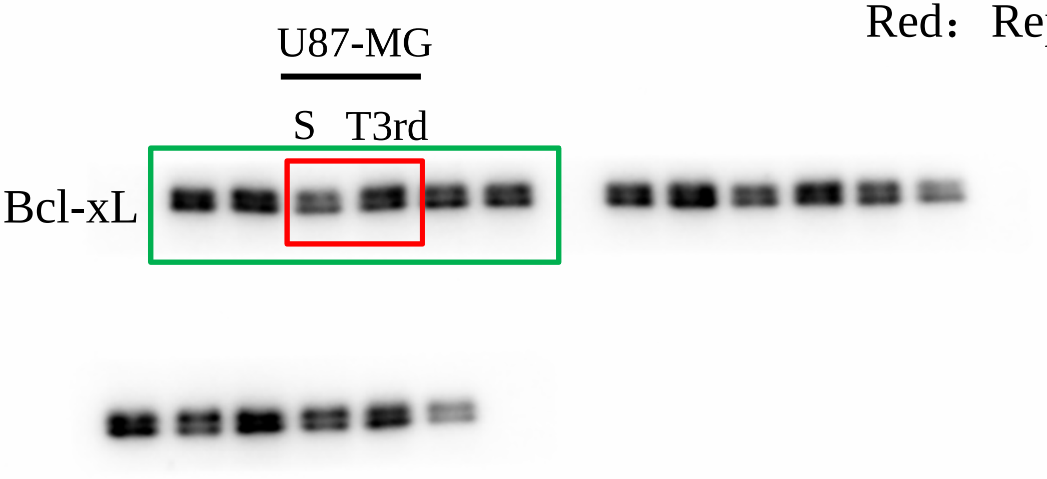

The membrane was imaged with Azure Biosystems 300

Full unedited gel for Figure Supplement 5B

Green: Statistical graph

Red: Representative graph

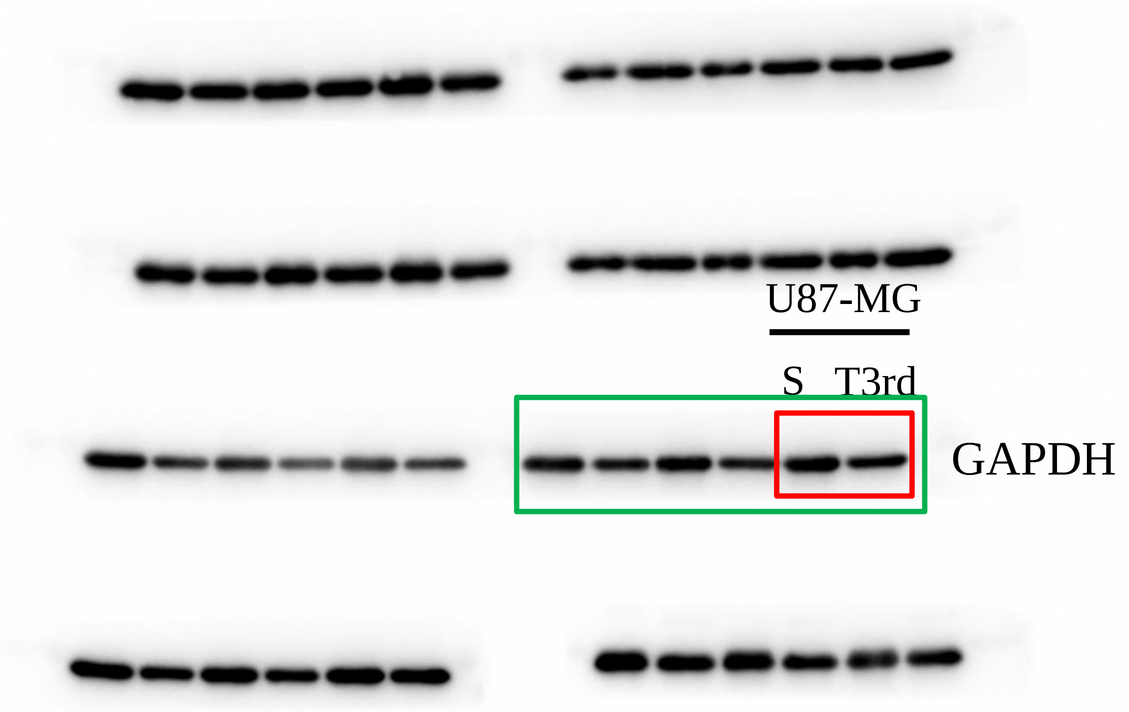

The membrane was imaged with Azure Biosystems 300

Full unedited gel for Figure Supplement 6A

Green： Statistical graph

Red： Representative graph

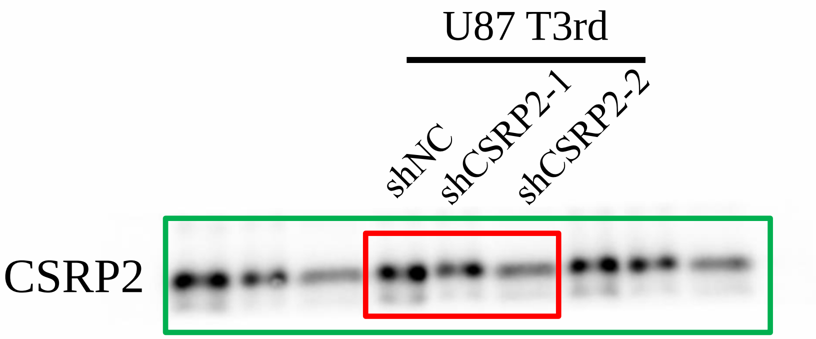

The membrane was imaged with Azure Biosystems 300

Full unedited gel for Figure Supplement 6A

Green： Statistical graph

Red： Representative graph

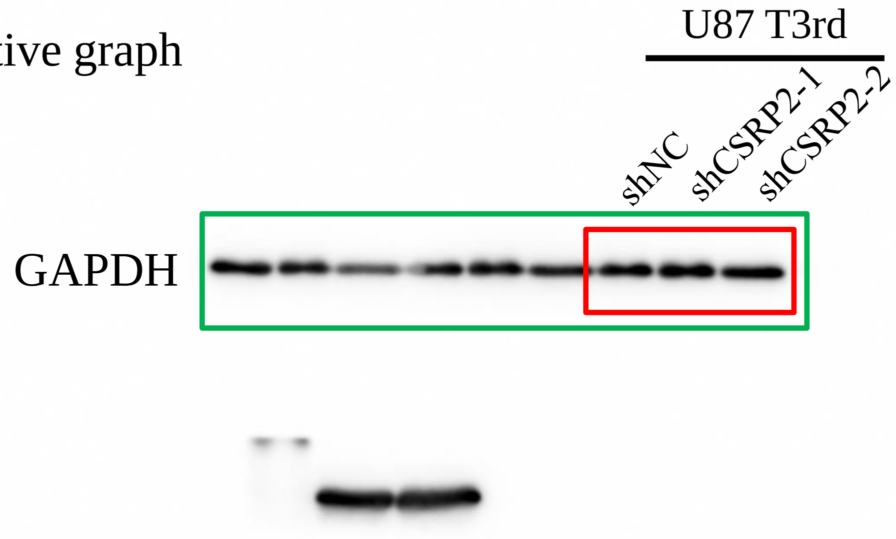

The membrane was imaged with Azure Biosystems 300

Full unedited gel for Figure Supplement 6I

Green： Statistical graph

Red： Representative graph

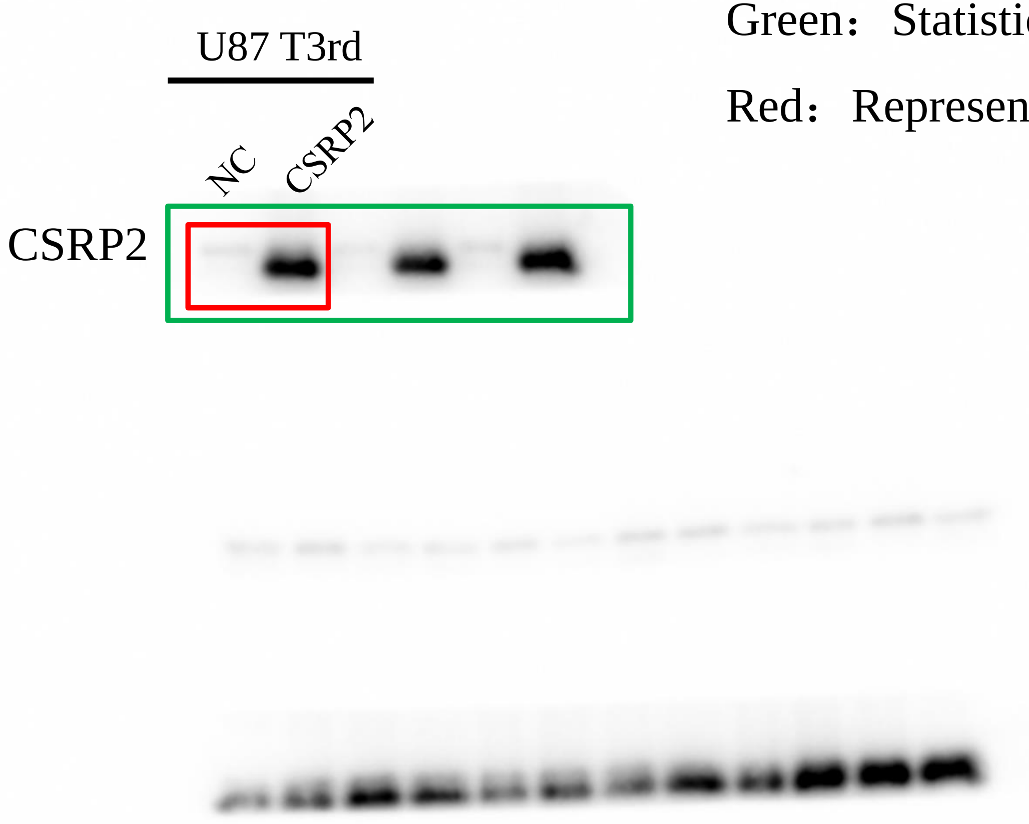

The membrane was imaged with Azure Biosystems 300

Full unedited gel for Figure Supplement 6I

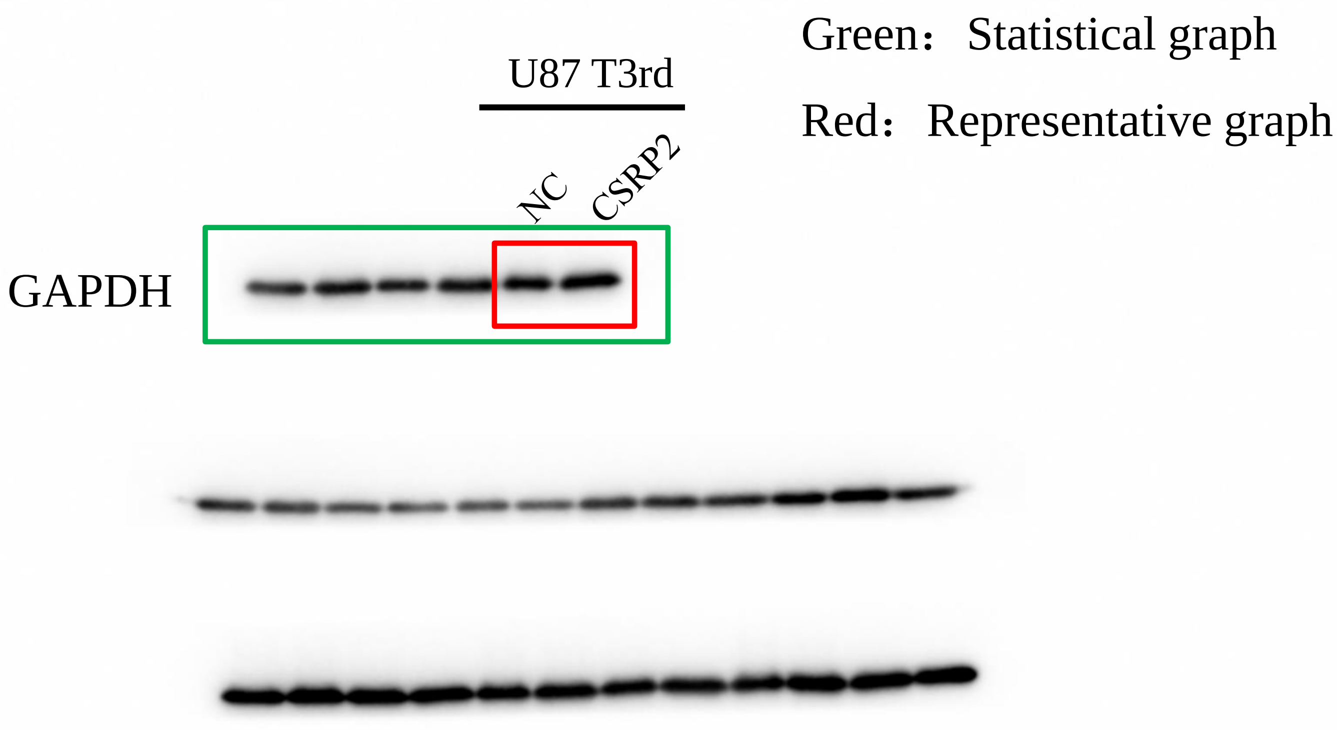

The membrane was imaged with Azure Biosystems 300
