# Supplementary material for: CSRP2 promotes the glioblastoma mesenchymal phenotype via p130Cas-mediated NF-κB and MAPK pathways
Source: J Exp Clin Cancer Res. 2025 Aug 5;44:228. doi: 10.1186/s13046-025-03484-7 (PMC12323131; doi:10.1186/s13046-025-03484-7)
Supplement: Supplementary file 2 — Supplementary Material 2 [file 13046_2025_3484_MOESM2_ESM.pdf]

CSRP2 promotes the glioblastoma mesenchymal phenotype via p130Cas-mediated NF-κB and MAPK pathways

Jiawei He et al.

Supplementary Figures S2-S6

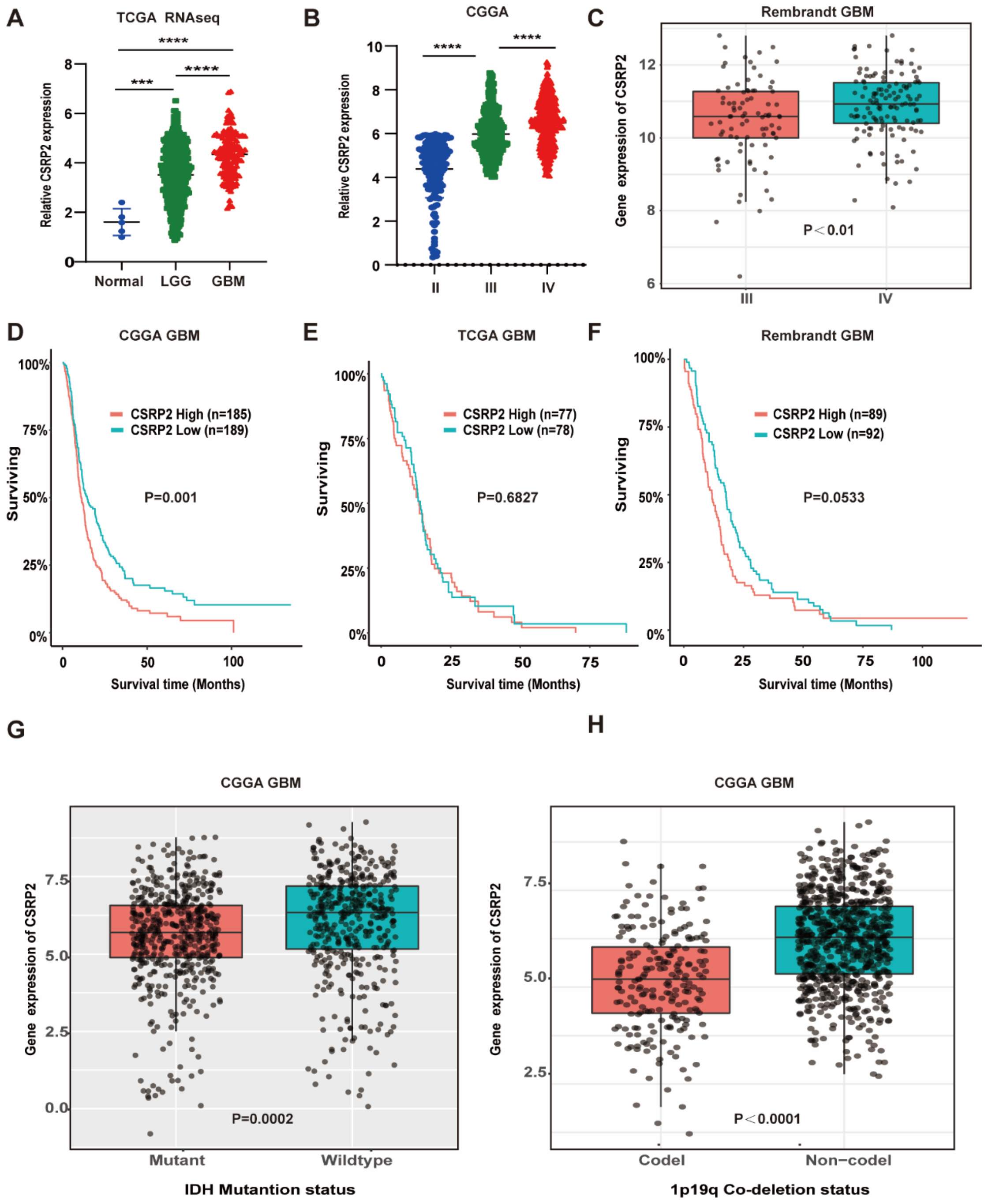

Supplementary Figure S2. CSRP2 expression is significantly upregulated in GBM patients and downregulated in GBM samples with IDH mutations and 1/19q codeletions. (A) Comparison of CSRP2 expression in normal, low-grade glioma (LGG), and GBM using the TCGA database. One-way ANOVA with Tukey's post hoc test, n = 5 for Normal, n = 500 for LGG, and n = 144 for GBM. (B) Comparison of CSRP2 expression between different WHO grades of glioma using the

CGGA database. One-way ANOVA with Tukey's post hoc test, n = 291 for grade II, n = 333 for grade III, and n = 388 for grade IV. (C) Comparison of CSRP2 expression between different WHO grades (III and IV) of glioma using the Rembrandt database. Unpaired t test, n = 85 for grade III and n = 183 for grade IV. (D) Kaplan–Meier analysis of progression-free survival using data from the CGGA database. Log-rank test. (E) Kaplan–Meier analysis of progression-free survival using data from the TCGA database. Log-rank test. (F) Kaplan–Meier analysis of progression-free survival using data from the Rembrandt database. Log-rank test. (G) Comparison of CSRP2 expression between GBM samples with and without IDH mutations. Unpaired t test, n = 531 for Mutant and n=435 for Wildtype. (H) Comparison of CSRP2 expression between GBM samples with and without 1/19q codeletions (Codel). Unpaired t test, n = 212 for Codel and n=728 for Non-codel. Data represent mean ± SEM. ***$p < 0.001$, ****$p < 0.0001$.

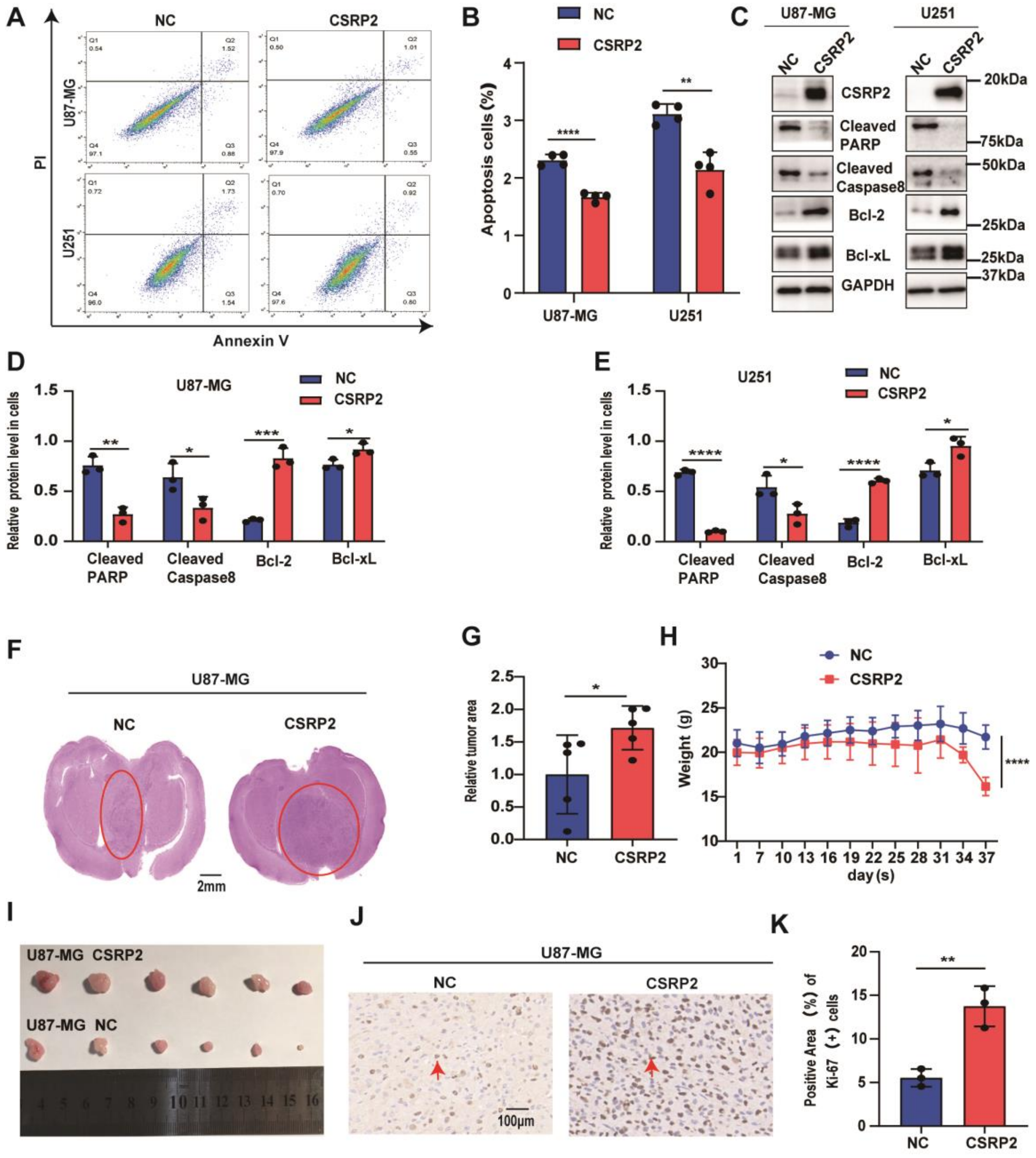

Supplementary Figure S3. CSRP2 overexpression reduces cell apoptosis and promotes tumor formation. (A, B) U87-MG and U251 cells with stable CSRP2 overexpression (CSRP2) and controls (NC) were measured by flow cytometry (A) for apoptosis ratio comparison (B). Unpaired t test, n = 4 per group. (C-E) Equal amounts of cell lysates of U87-MG and U251 cells with CSRP2 overexpression were subjected to western blotting (C) and quantification analysis (D, E) for apoptosis-related proteins. Unpaired t test, n = 3 per group. (F, G) Representative H&E staining

showing tumor regions (in red circles）in mice xenografted with U87-MG cells stably overexpressing CSRP2 or control cells (F). Scale bars: 2 mm. Tumor regions were subjected to comparison (G). Unpaired t test, n = 5 per group. (H) Body weight change of mice xenografted with U87-MG cells stably overexpressing CSRP2 or control cells. Two-way ANOVA with Sidak's post hoc test, n = 6 per group. (I) Brain tumor images of mice xenografted with U87-MG cells stably overexpressing CSRP2 or control cells, n = 6 per group. (J, K) Representative Ki-67 staining in mice xenografted with U87-MG cells stably overexpressing CSRP2 or control cells (J). Scale bars: 100 μm. Ki-67 positive cells were quantified for comparison (K). Unpaired t test, n = 3 per group. Data represent mean ± SEM. *$p < 0.05$, **$p < 0.01$, ***$p <0.001$, ****$p <0.0001$.

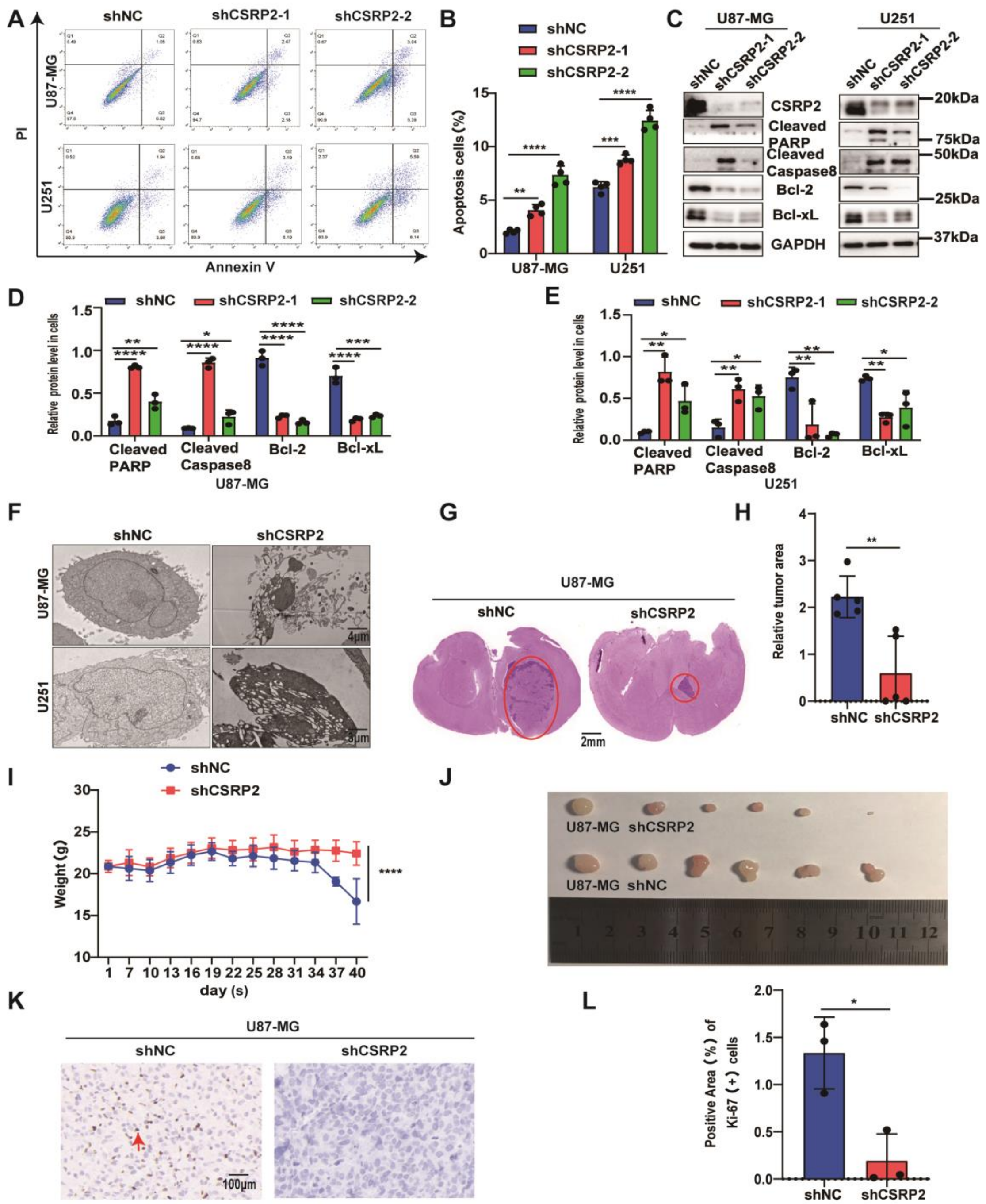

Supplementary Figure S4. CSRP2 knockdown promotes cell apoptosis and reduces tumor formation. (A, B) U87-MG and U251 cells with stable CSRP2 knockdown (shCSRP2-1 and shCSRP2-2) and control cells (shNC) were measured by flow cytometry (A) for apoptosis ratio comparison (B). One-way ANOVA with Tukey's post hoc test, n = 4 per group. (C-E) Equal amounts of cell lysates of U87-MG and U251 cells with CSRP2 knockdown and control cells were subjected to

western blotting (C) and quantification analysis (D, E) for apoptosis-related proteins. One-way ANOVA with Tukey's post hoc test, n = 3 per group. (F) Representative electron microscopy images showing apoptotic cell bodies with CSRP2 knockdown. U87-MG: 8K, scale bars: 4 μm. U251: 10K, scale bars: 3 μm. (G, H) Representative H&E staining showing tumor regions (in red circles) in mice xenografted with U87-MG cells with CSRP2 knockdown or control cells (G). Scale bars: 2 mm. Tumor regions were compared (H). Unpaired t test, n = 5 per group. (I) Body weight change of mice xenografted with U87-MG cells with CSRP2 knockdown or control cells. Two-way ANOVA with Sidak's post hoc test, n = 5 per group. (J) Brain tumor images of mice xenografted with U87-MG cells with CSRP2 knockdown or control cells, n = 6 per group. (K, L) Representative Ki-67 staining in mice xenografted with U87-MG cells with CSRP2 knockdown or control cells (K). Scale bars: 100 μm. Ki-67 positive cells were quantified for comparison (L). Unpaired t test, n = 3 per group. Data represent mean ± SEM. *p < 0.05, **p < 0.01, ***p < 0.001,****p <0.0001.

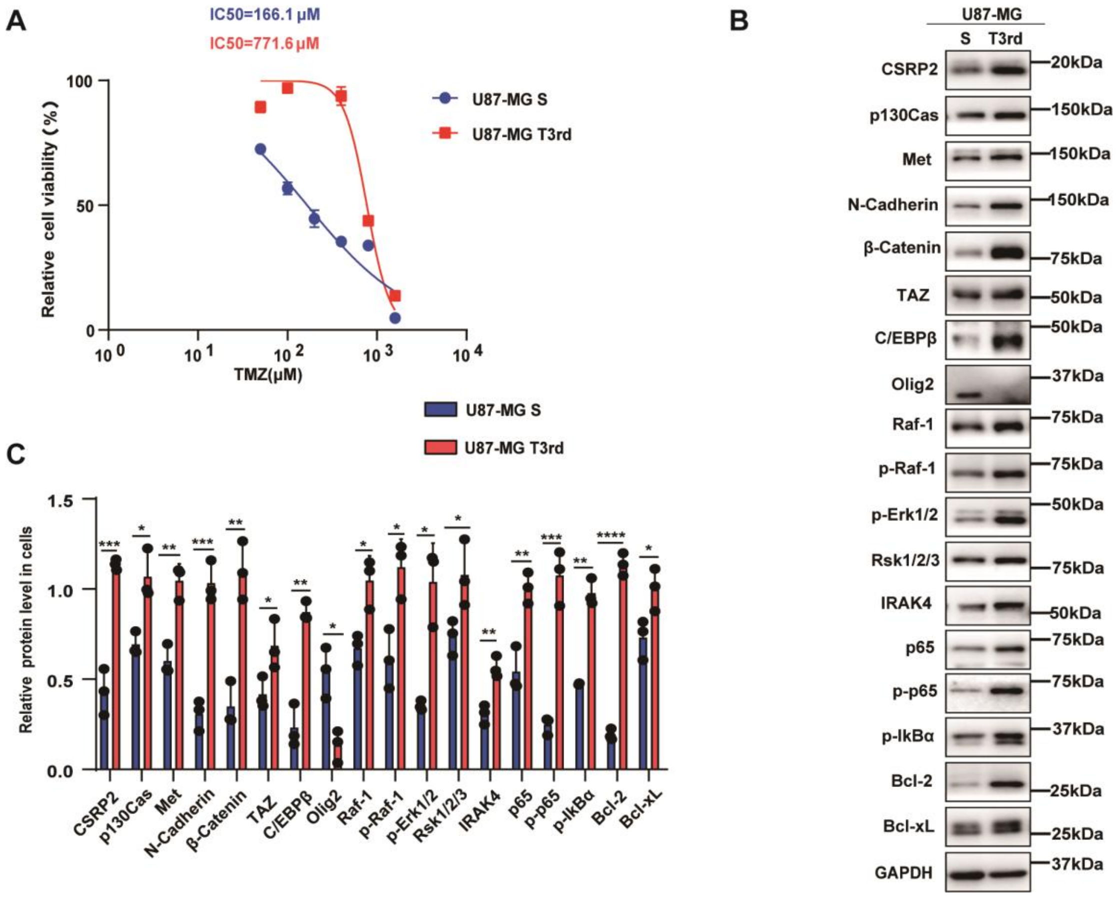

Supplementary Figure S5. TMZ resistance results in elevated CSRP2 expression, PMT, and the NF-κB and the MAPK signaling activities. (A) Dose-response curves of TMZ treatment in TMZ-resistant (U87-MG T3rd) and TMZ-sensitive (U87-MG S) cell lines. IC50 of TMZ was measured using nonlinear regression analysis of the dose-response curves. n = 4 per group. (B, C) Levels of CSRP2, as well as PMT-, apoptosis-, and NF-κB and MAPK signaling-related proteins in U87-MG T3rd and U87-MG S cell lines were analyzed by western blotting (B) and densitometry quantification comparison (C). Unpaired t test, n = 3 per group. Data represent mean ± SEM. *$p < 0.05$, **$p < 0.01$, ***$p < 0.001$, ****$p < 0.0001$.

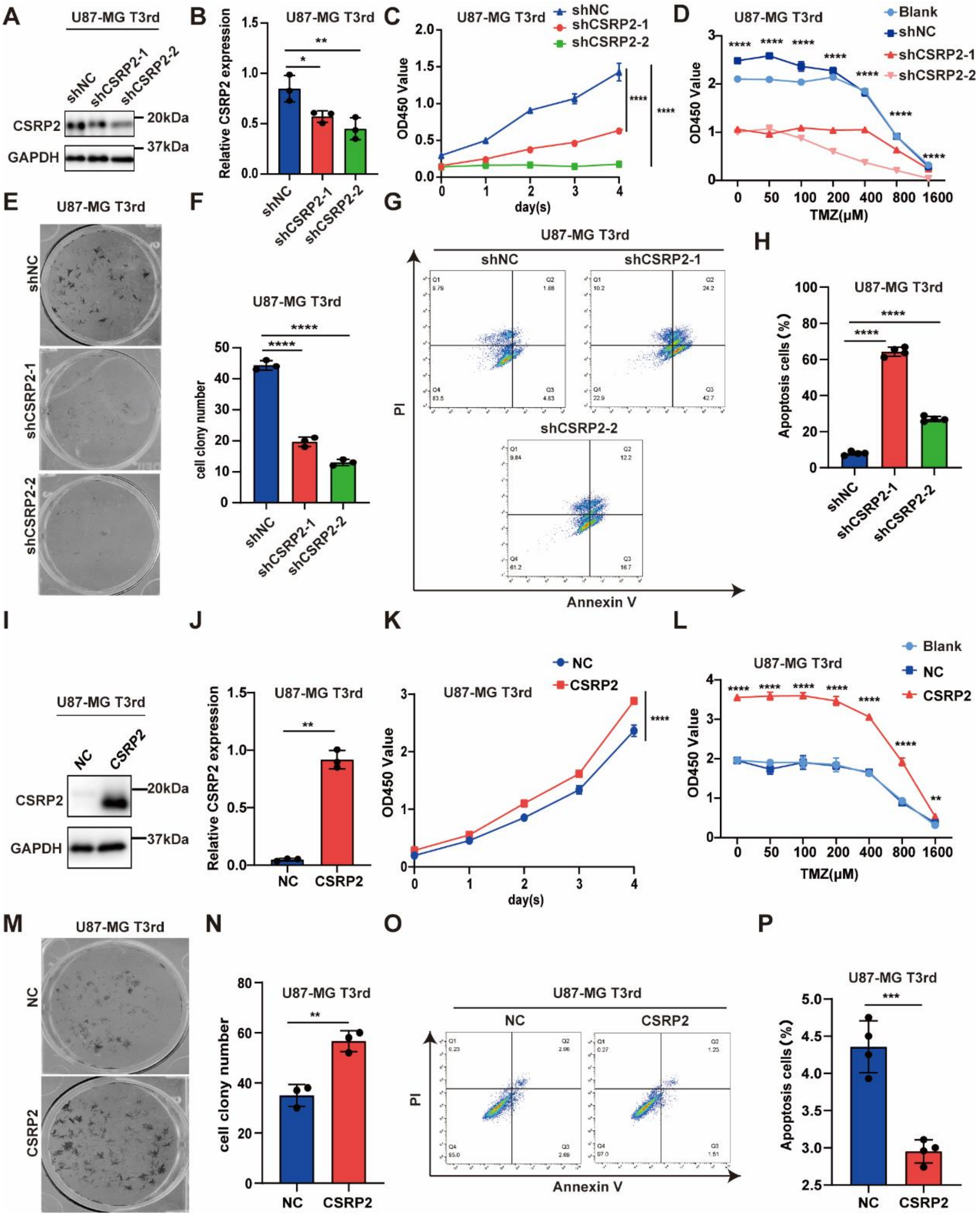

Supplementary Figure S6. CSRP2 knockdown restores TMZ sensitivity while CSRP2 overexpression enhances TMZ resistance in TMZ-resistant cells.

(A, B) U87-MG T3rd cells were stably transduced with two CSRP2 shRNA lentiviruses (shCSRP2-1 and shCSRP2-2) or control shRNA lentiviruses (shNC). CSRP2 protein levels in these cells were analyzed by western blotting (A) and

densitometry quantification for comparison (B). One-way ANOVA with Tukey's post hoc test, n = 3 per group. (C) U87-MG T3rd cells with CSRP2 knockdown and controls were assayed for their proliferation. Two-way ANOVA with Sidak's post hoc test, n = 6 per group. (D) U87-MG T3rd cells with CSRP2 knockdown and controls were treated with indicated amounts of TMZ for 48 h, and then assayed for their proliferation. Two-way ANOVA with Sidak's post hoc test, n = 5 per group. (E, F) U87-MG T3rd cells with CSRP2 knockdown and controls were assayed for their colony formation (E) and the colony numbers were compared (F). One-way ANOVA with Tukey's post hoc test, n = 3 per group. (G, H) Apoptosis of U87-MG T3rd cells with CSRP2 knockdown and controls was measured by flow cytometry (G) for apoptosis ratio comparison (H). One-way ANOVA with Tukey's post hoc test, n = 4 per group. (I, J) U87-MG T3rd cells were stably transduced with lentiviruses expressing CSRP2 or control (NC). CSRP2 protein levels in these cells were analyzed by western blotting (I) and densitometry quantification for comparison (J). Unpaired t test, n = 3 per group. (K) U87-MG T3rd cells with CSRP2 overexpression and controls were assayed for their proliferation. Two-way ANOVA with Sidak's post hoc test, n = 6 per group. (L) U87-MG T3rd cells with CSRP2 overexpression and controls were treated with indicated amounts of TMZ for 48 h and then assayed for their proliferation. Two-way ANOVA with Sidak's post hoc test, n = 5 per group. (M, N) U87-MG T3rd cells with CSRP2 overexpression and controls were assayed for their colony formation (M) and the colony numbers were compared (N). Unpaired t test, n = 3 per group. (O, P) Apoptosis of U87-MG T3rd cells with CSRP2

overexpression and controls was measured by flow cytometry (O) for apoptosis ratio comparison (P). Unpaired t test, n = 4 per group. Data represent mean ± SEM. *p < 0.05, **p < 0.01, ***p < 0.001, ****p <0.0001.
